# Genome-wide analysis of heat shock proteins in C_4_ model, foxtail millet identifies potential candidates for crop improvement under abiotic stress

**DOI:** 10.1038/srep32641

**Published:** 2016-09-02

**Authors:** Roshan Kumar Singh, Jananee Jaishankar, Mehanathan Muthamilarasan, Shweta Shweta, Anand Dangi, Manoj Prasad

**Affiliations:** 1 National Institute of Plant Genome Research, Aruna Asaf Ali Marg, New Delhi – 110067, India

## Abstract

Heat shock proteins (HSPs) perform significant roles in conferring abiotic stress tolerance to crop plants. In view of this, HSPs and their encoding genes were extensively characterized in several plant species; however, understanding their structure, organization, evolution and expression profiling in a naturally stress tolerant crop is necessary to delineate their precise roles in stress-responsive molecular machinery. In this context, the present study has been performed in C_4_ panicoid model, foxtail millet, which resulted in identification of 20, 9, 27, 20 and 37 genes belonging to *SiHSP100*, *SiHSP90*, *SiHSP70*, *SiHSP60* and *SisHSP* families, respectively. Comprehensive *in silico* characterization of these genes followed by their expression profiling in response to dehydration, heat, salinity and cold stresses in foxtail millet cultivars contrastingly differing in stress tolerance revealed significant upregulation of several genes in tolerant cultivar. *SisHSP-27* showed substantial higher expression in response to heat stress in tolerant cultivar, and its over-expression in yeast system conferred tolerance to several abiotic stresses. Methylation analysis of *SiHSP* genes suggested that, in susceptible cultivar, higher levels of methylation might be the reason for reduced expression of these genes during stress. Altogether, the study provides novel clues on the role of HSPs in conferring stress tolerance.

Plants in the environment are exposed to several abiotic and biotic stresses which pose serious threat to their survival and productivity; however, plants are evolved with sophisticated molecular machinery to sense and circumvent the stresses. In response to abiotic stresses, plants produce several biomolecules called molecular chaperones, which function in protecting the cells from the adverse impact of stresses. A class of such molecular chaperones are called heat shock proteins (HSP), which are synthesized in response to several stresses including low temperature, osmotic, salinity, oxidative, desiccation, high intensity irradiations, wounding, and heavy metals stresses^1^,^2^,^3^. The role of HSPs during stress and unstressed conditions is regulation of protein folding and accumulation along with their localization and degradation^4^,^5^,^6^,^7^. Nevertheless, the precise role of HSPs in regulating the molecular mechanisms responsible for normal growth and development, and stress response remains elusive^1^.

In plants, HSPs are classified into five principal classes, namely, HSP100, HSP90, HSP70/DnaK, HSP60/GroE and small heat shock proteins (sHSP) based on their molecular weight^8^. In order to delineate the molecular roles of these HSPs, several studies on identification and characterization of HSPs and their corresponding genes were performed in plant species such as Arabidopsis, tomato and rice^6^,^9^,^10^,^11^. In rice, 10, 9, 26 and 29 HSPs were identified belonging to HSP100, HSP90, HSP70, and sHSPs, respectively. Expression profiling of these HSP encoding genes in response to heat, cold, drought and salt stresses showed their differential expression with significant upregulation of *sHSP* genes during heat stress^6^. Identification and expression profiling of *sHSP* genes in barley during drought stress was reported by Reddy *et al*.^12^. The study identified 20 *sHSP*s, which are shown to be differentially regulated in response to drought stress. A candidate sHSP protein, Hsp17.5-CI was expressed in *E. coli,* which showed *in vitro* chaperone activity^12^. Among poaceae members, analysis of HSPs was performed only in rice and barley; however, no comprehensive investigation has been conducted in the C_4_ panicoid model species, foxtail millet (*Setaria italica* L.).

Foxtail millet is a naturally abiotic stress tolerant crop, which is predominantly cultivated for food and fodder in arid and semi-arid regions of the world^13^. In consequence of its C_4_ photosynthetic trait, remarkable tolerance to drought, heat and salinity, and genetic close-relatedness to several bioenergy grasses, foxtail millet is considered as a model species for studying C_4_ photosynthesis, abiotic stress tolerance and biofuel traits, respectively^14^. Being a model crop, foxtail millet has gained popularity among millet research community and several comprehensive researches have been pursued to identify and characterize the role of important stress-responsive gene families including *NAC, WD40, AP2/ERF, C*_*2*_*H*_*2*_ *zinc finger, MYB, DCL, AGO, RDR, WRKY* and *ADP-ribosylation factors* in conferring abiotic stress tolerance^15^,^16^,^17^,^18^,^19^,^20^,^21^,^22^,^23^,^24^. But no reports are available till date on structure, organization, evolution and expression profiling of HSPs in response to abiotic stresses in this model crop. Therefore, the present study was conducted to identify the HSPs encoded in foxtail millet genome and characterize them using *in silico* tools, and analyze their expression patterns during stress treatments. Further, the study identified a potential candidate showing several fold upregulation in stress tolerant foxtail millet cultivar, and its heterologous over-expression in yeast system conferred tolerance to several abiotic stresses.

## Results and Discussion

### HSP gene families and their organization in foxtail millet

**Figure 1:**
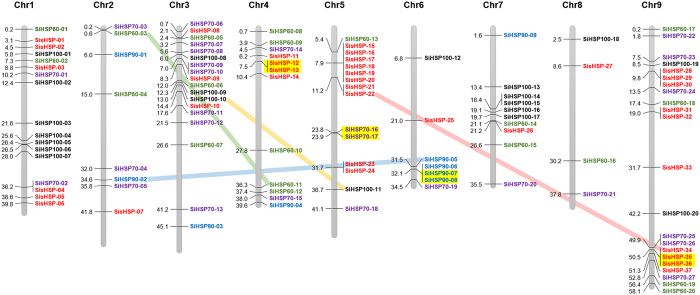
Physical map showing chromosomal location of *SiHSP* genes in foxtail millet. *SiHSP* genes were mapped onto nine chromosomes of foxtail millet and the physical map was generated. The vertical bars represent chromosomes with position of *SiHSP* genes on the left (in Mbp) and name of the gene on the right. Tandemly duplicated gene-pairs are highlighted in yellow and segmental duplications are shown by coloured lines.

The study identified 22, 11, 32, 24 and 41 non-redundant proteins belonging to HSP100 (SiHSP100), HSP90 (SiHSP90), HSP70 (SiHSP70), HSP60 (SiHSP60) and sHSP (SisHSP) classes, respectively in foxtail millet. Among these, three genes from *SiHSP100*, *SiHSP60* and *SisHSP* families produce splice variants. Further, alternate transcripts were produced by two genes from *SiHSP90* and *SiHSP70* families. Of these, maximum of three splice variants was produced by *SiHSP70-10*, whereas *SiHSP100-10*, *SiHSP70-01*, *SiHSP60-08* and *SisHSP-05* produced two alternate transcripts each (Supplementary Table 1). Removal of these alternate transcripts revealed the presence of 20, 9, 27, 20 and 37 primary transcripts belonging to SiHSP100, SiHSP90, SiHSP70, SiHSP60 and SisHSP families, respectively (Supplementary Table 1). In rice, 10, 9, 26 and 29 proteins of OsHSP100, OsHSP90, OsHSP70 and OssHSP families have been identified^6^, and in comparison to this, foxtail millet is found to encode more heat shock proteins than rice. Genes encoding these proteins were mapped onto nine chromosomes of foxtail millet to generate the physical map, which showed an uneven distribution of these genes in the genome (Fig. 1). Altogether, chromosome 9 was found to harbor a maximum of 22 genes (19.5%) and chromosome 8 had a minimum of 4 genes (3.5%). Examining the family-wise distribution of HSP genes showed that *SiHSP100* genes were present on chromosomes 1, 3, 5, 6, 7 and 9; *SiHSP90* on chromosomes 2, 3, 4, 6 and 7; and, *SiHSP60* on all the chromosomes except chromosome 8. *SiHSP70* and *SisHSP* genes were present on all the nine chromosomes. The expansion of *SiHSP* gene families could be attributable to duplication events that have occurred in the genome, which could be tandem and/or segmental duplications. Examining the occurrence of these duplications among *SiHSP* gene families showed that four genes underwent tandem and segmental duplications (Supplementary Table 2). *SisHSP12:SisHSP13, SiHSP70-16:SiHSP70-17, SiHSP90-07:SiHSP90-08,* and *SisHSP-35:SisHSP-36* were tandemly duplicated gene-pairs present on chromosomes 4, 5, 6 and 9, respectively; whereas *SisHSP-22:SisHSP34, SiHSP60-03:SiHSP60-11, SiHSP90-02:SiHSP90-05,* and *SiHSP100-10:SiHSP100-11* were found to be segmentally duplicated gene-pairs (Fig. 1). The distance between tandemly duplicated genes ranged from ~0.4 kb (SisHSP-35: SisHSP-36) to ~101.4 kb (SiHSP70-16: SiHSP70-17) with an average of 30.6 kb.

Different classes of HSPs and their corresponding genes identified in foxtail millet were analyzed for their properties, which showed diverse variations that exists within the gene families. Variations in terms of gene length, number of introns and exons, protein length and their physico-chemical properties were evidenced in different classes of heat shock proteins (Supplementary Table 1; Supplementary Fig. 1). Among *SiHSP100* family, the lengths of genes varied from 2692 bp (*SiHSP100-03*) to 7773 bp (*SiHSP100-02*), though the difference in gene length was not necessarily reflected in protein length as *SiHSP100-13* was found to encode the largest protein (3491 amino acids; 30.95 kDa), but its gene length was 4290 bp. *SiHSP100-19* encodes for the smallest protein of 1054 amino acids (100.56 kDa). SiHSP100-07 was found to encompass a maximum of 14 introns, whereas SiHSP100-03 and SiHSP100-13 possessed 2 introns only. In case of *SiHSP90*, the gene lengths varied between 2284 (*SiHSP90-01*) and 6896 (*SiHSP90-03*), with highest number of 19 introns in *SiHSP90-03*. Length of the proteins also varied with gene length, as *SiHSP90-01* and *SiHSP90-03* encode for smallest (414 amino acids; 47.28 kDa) and largest proteins (817 amino acids; 91.6 kDa), respectively.

Similarly, in *SiHSP70* class, the smallest gene was *SiHSP70-06* (1121 bp) and the largest was *SiHSP70-02* (6104 bp). Distribution of introns and exons varied significantly with the variations in gene length, and the largest gene, *SiHSP70-02*, encompasses maximum of 13 introns, whereas eleven genes possess only one intron and *SiHSP70-17* was intronless (Supplementary Fig. 1). The smallest protein of this class was *SiHSP70-06* (295 amino acids; 32.3 kDa) and the largest protein was *SiHSP70-02* (890 amino acids; 98.23 kDa). In case of *SiHSP60* class genes, *SiHSP60-17* was found to be smallest gene (3286 bp) and *SiHSP60-05* was the largest (6713 bp). Compared to other HSP family members, *SiHSP70* genes possess more introns, ranging from 0 (intron-less; *SiHSP70-17*) to 13 (*SiHSP70-02*) (Supplementary Fig. 1). Comparing the protein lengths of SiHSP60 proteins showed that SiHSP60-13 was the smallest (525 amino acids; 57.4 kDa), whereas SiHSP60-10 was the largest (655 amino acids; 70.94 kDa). Similarly, *SisHSP* class genes varied in length from 494 bp (*SisHSP-21*) to 3748 bp (*SisHSP-28*), with a variation in protein length ranging from 147 amino acids (SisHSP-14; 15.95 kDa) to 591 amino acids (SisHSP-28; 63.2 kDa). Contrasting to other *SiHSP* class genes, *SisHSP* genes possess a maximum of 2 introns, and a total of 13 genes were intronless (Supplementary Fig. 1). Compared to the data of homologs from other members of Poaceae, SiHSP members show a wide variation in length of gene and protein, intron-exon distribution, pI and molecular weight, which suggests the presence of putative novel variants among SiHSP members.

### Phylogenetic classification and domain architecture of SiHSPs

**Figure 2:**
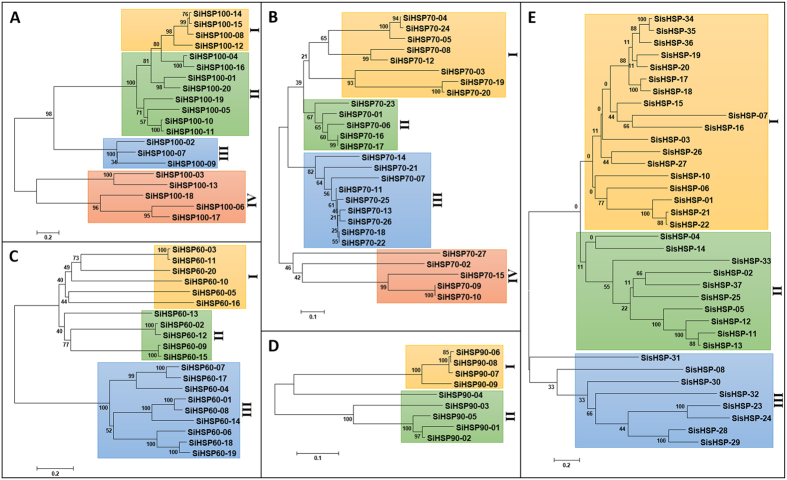
Phylogenetic relationship of SiHSP proteins. Unrooted phylogenetic tree deduced by neighbor-joining method showing the phylogenetic relationship and classification of **(A)** HSP100, **(B)** HSP70, **(C)** HSP60, **(D)** HSP90, and **(E)** sHSP proteins. Sub-classes are shaded in different colours.

Phylogenetic analysis followed by identification of different functional domains in SiHSPs enabled the classification of these proteins into different sub-classes (Fig. 2; Supplementary Table 3). SiHSP100 class proteins were classified into four sub-classes (I to IV). Sub-classes II and III possess one or more domains belonging to AAA (ATPase family associated with various cellular activities), AAA_2 (Cdc48 subfamily), AAA_5 (dynein-related subfamily), Sigma54_activat (Sigma-54 interaction domain), Clp_N (Clp amino terminal domain, pathogenicity island component) and ClpB_D2-small (C-terminal, D2-small domain, of ClpB protein) domain. Proteins belonging to sub-class I possess an additional UVR (UvrB/uvrC motif) domain, whereas sub-class IV proteins have only Clp_N and ClpB_D2-small domains (Fig. 2A; Supplementary Table 3). Similarly, SiHSP70 proteins were classified into four sub-classes (I to IV), and all the members of SiHSP70 possess the conserved HSP70 domain (PF00012.17) and MreB_Mbl (MreB/Mbl protein) domain. However, MreB_Mbl domain was absent in SiHSP70-06 and SiHSP70-07 proteins, and interestingly, SiHSP70-07 has three HSP70 domains (Fig. 2B; Supplementary Table 3). The phylogenetic analysis classified SiHSP60 proteins into three sub-classes (I to III), and all the proteins of this family were found to possess the conserved Cpn60_TCP1 (TCP-1/cpn60 chaperonin family) domain (Fig. 2C; Supplementary Table 3). In case of SiHSP90s, the phylogenetic tree classified the proteins into two sub-classes (I and II) with 4 and 5 proteins, respectively, in each sub-class. Domain analysis revealed the presence of two domains, namely, HSP90 and HATPase_c (Histidine kinase-, DNA gyrase B-, and HSP90-like ATPase). Although all the SiHSP90 proteins possess the conserved HSP90 domain, HATPase_c domain is absent in SiHSP90-01 and SiHSP90-06. Noteworthy, SiHSP90-01 has two HSP90 domains (Fig. 2D; Supplementary Table 3). SisHSP family proteins were classified into three major sub-classes with 18, 10 and 8 proteins, respectively, in each sub-class. All the proteins of this class have the conserved HSP20 (Hsp20/alpha crystallin family) domain. As exceptions, SisHSP-28 has two HSP20 domains, and SiHSP-27 has an additional CS domain (Fig. 2E; Supplementary Table 3). The phylogenetic trees also showed that duplicated gene-pairs were grouped together into single clade with strong bootstrap support (Fig. 2).

### *Cis-*regulatory elements in promoter region of *SiHSP* genes

*In silico* analysis of *cis-*regulatory elements in the promoter region of all *SiHSP* genes revealed the presence of 293 different *cis-*elements in the upstream region of these genes (Table 1; Supplementary Table 4). In *SiHSP100* genes, a total of 220 *cis-*elements were detected, of which many were present in all the 20 *SiHSP100* genes and a few were uniquely present in any one gene. *Cis-*elements including ABRE3HVA22 (*SiHSP100-13*), ANAERO5CONSENSUS (*SiHSP100-14*), EMBP1TAEM (*SiHSP100-07*) and OPAQUE2ZMB32 (*SiHSP100-18*) were unique to *SiHSP100* genes. In case of *SiHSP90* genes, 190 *cis-*elements were identified to be present in the promoter region, of which, AGCBOXNPGLB, ANAERO4CONSENSUS, GADOWNAT, LTRE1HVBLT49 and TRANSINITDICOTS were present only in *SiHSP90-02, SiHSP90-07, SiHSP90-04, SiHSP90-05* and *SiHSP90-09*, respectively (Table 1). Similarly, in *SiHSP70* genes, a total of 237 *cis-*elements were detected, of which, ABREDISTBBNNAPA, ACGTABOX, AUXREPSIAA4, CACGCAATGMGH3, E2FAT, GCBP2ZMGAPC4 and SURE2STPAT21 were uniquely present in SiHSP70-18, *SiHSP70-26, SiHSP70-22, SiHSP70-10, SiHSP70-08, SiHSP70-01* and *SiHSP70-12* genes, respectively.

In case of *SiHSP60* family genes, 218 *cis-*elements were present in the promoter region. In this family, ABREMOTIFAOSOSEM (*SiHSP60-04*), ACIPVPAL2 (*SiHSP60-13*), AMMORESIIUDCRNIA1 (*SiHSP60-12*), ARE1 (*SiHSP60-09*), MNF1ZMPPC1 (*SiHSP60-17*), OCTAMOTIF2 (*SiHSP60-10*), RGATAOS (*SiHSP60-11*) and SITEIOSPCNA (*SiHSP60-05*) are uniquely present in *SiHSP60* genes. A total of 248 *cis-*elements were detected in the upstream region of *SisHSP* genes. Among these, ABREBZMRAB28, ANAERO5CONSENSUS, CEREGLUBOX1PSLEGA, HY5AT, OCTAMOTIF2, PIATGAPB and VOZATVPP were present only in *SisHSP-10, SisHSP-34, SisHSP-21, SisHSP-24, SisHSP-11, SisHSP-17* and *SisHSP-28* genes, respectively (Table 1; Supplementary Table 4).

### Comparative mapping of *SiHSP* genes in sequenced grass genomes

**Figure 3:**
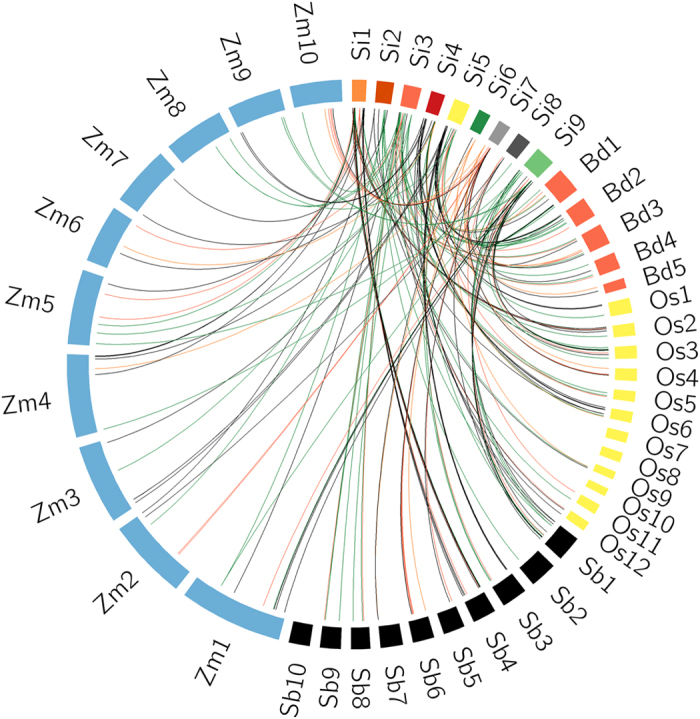
Comparative physical mapping of *SiHSP* genes. Orthologous relationship of foxtail millet *SiHSP* genes distributed on nine chromosomes (Si) with the genes of sorghum (Sb), maize (Zm), rice (Os) and *Brachypodium* (Bd). The coloured blocks represent the chromosomes.

In order to derive the orthologous relationship between *HSP* genes of foxtail millet and other sequenced grass genomes including sorghum, maize, rice and *Brachypodium*, comparative genome mapping was performed (Fig. 3). A total of 86 *SiHSP* genes (~76%) belonging to different families showed orthologous relationships with other crops, where maximum synteny was observed between foxtail millet and sorghum (84 genes; ~98%), followed by maize (76 genes; ~88%), rice (65 genes; ~75.5%) and *Brachypodium* (47 genes; ~54.6%) (Supplementary Table 5). In case of foxtail millet-sorghum synteny, all the genes present in chromosome 1 of foxtail millet showed 100% orthology with sorghum chromosome 4. Similarly, genes present in foxtail millet chromosomes 5 and 9 showed 100% orthology and synteny with sorghum chromosomes 3 and 1, respectively. Similar observation was reported by Puranik *et al*.^15^ where *SiNAC* genes on foxtail millet chromosome 6 showed 100% collinearity to sorghum chromosome 7. Though similar observations could not be made in foxtail millet-maize synteny, *SiHSP* genes on chromosome 1 of foxtail millet showed 100% synteny with rice chromosome 2. However, difference in the orientation of genes was observed in the present study, which may be attributed to nested chromosomal fusion that has frequently occurred in these genomes during the course of evolution. Further, decrease in number of orthologous genes between foxtail millet-sorghum (~98%), -maize (~88%), -rice (~75.5%) and -*Brachypodium* (~54.6%) reveals the close-evolutionary relationship of foxtail millet with sorghum and maize, followed by rice and *Brachypodium.* This is in agreement with the comparative maps developed using *NAC*^15^, *WD40*^16^, *AP2/ERF*^17^, *C*_*2*_*H*_*2*_ *zinc finger* and *MYB* transcription factors^18^,^19^, *DCL, AGO* and *RDR*^20^, *14-3-3*^21^, secondary cell wall genes and WRKY transcription factors^22^,^23^, and ADP-ribosylation factor^24^ gene families of foxtail millet.

### Duplication and divergence rates of paralogous and orthologous genes

To deduce the effect of Darwinian selection in duplication and divergence of *HSP* genes, the ratios of rate of non-synonymous substitution (Ka) to synonymous substitution (Ks) for paralogous as well as orthologous gene-pairs were estimated (Supplementary Tables 2 and 5). The ratios of Ka/Ks for tandemly duplicated gene-pairs ranged from 0.09 to 0.1 with an average of 0.1, and segmentally duplicated gene-pairs ranged from 0.06 to 0.1 (with an average of 0.1), which demonstrated that the genes were under strong purifying selection pressure (Ka/Ks < 1). The estimated time of divergence for tandemly and segmentally duplicated genes were ~26 and ~23 million years ago (mya) (Supplementary Table 2). These concords to the whole genome tandem and segmental duplications which were estimated to have occurred around 25–27 and 18–22 mya^25^. Similarly, average Ka/Ks ratios for orthologous gene-pairs between foxtail millet-sorghum, -maize, -rice and -*Brachypodium* were estimated to be 0.2, 0.3, 0.6 and 0.4, respectively (Supplementary Table 5). Relatively higher Ka/Ks ratio between foxtail millet and rice could be due to the occurrence of synonymous substitutions at higher rate, whereas Ka/Ks ratio was minimum between foxtail millet and sorghum. This showed that the gene-pairs between foxtail millet and sorghum were under intense purifying selection when compared to foxtail millet-maize, -rice and -*Brachypodium*. The estimated divergence time of foxtail millet-sorghum gene-pairs was ~18 mya, whereas the time of divergence for foxtail millet-maize, -rice and -*Brachypodium* were ~20, ~37 and ~57 mya. These results are comparable to genome-wide evolutionary studies performed with several stress-responsive gene families, where the time of divergence between foxtail millet-sorghum, -maize, -rice and -*Brachypodium* were estimated as ~27, ~34 and ~55 mya^15^,^16^,^17^,^18^,^19^,^20^,^21^,^22^,^23^,^24^.

### Expression profiles of *SiHSP* genes

**Figure 4:**
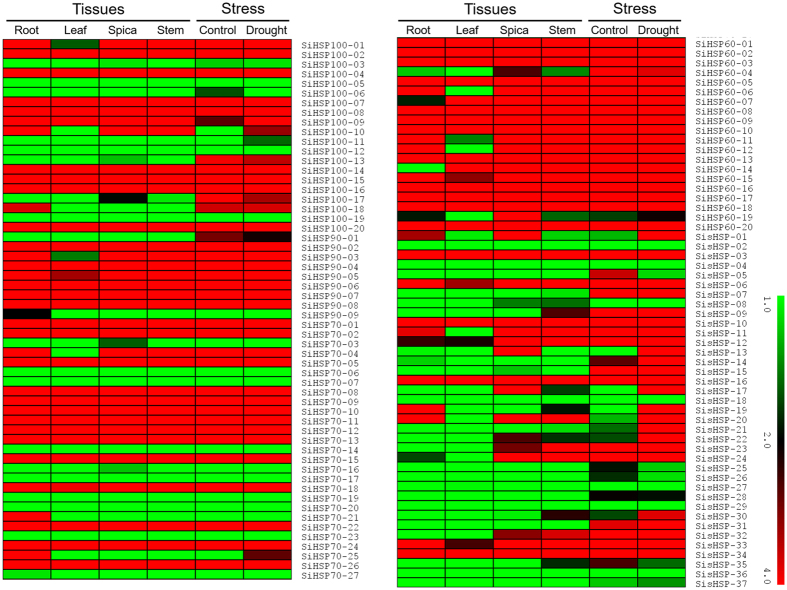
Heat map showing the expression pattern of *SiHSP* genes. Expression pattern of all the 113 *SiHSP* genes in four tissues namely, root, leaf, spica and stem, and drought stress library of foxtail millet is shown. The coloured bar at bottom right represents relative expression value, where 1.0, 2.0 and 4.0 denotes low, medium and high expression, respectively.

RNA-seq derived expression level of all the 113 *SiHSP* genes in four tissues namely, leaf, stem, root and spica and drought stress library was investigated (Fig. 4). The heat map revealed differential expression pattern of the genes in tissues and in response to drought stress. Many genes were observed to be highly expressed in all the tissues and stress, particularly, the members of *SiHSP60* family showed several fold higher expression in all the four tissues as well as in response to drought stress. Their upregulated expression in these samples suggests their role as chaperones which participate in the folding and aggregation of proteins that are mobilized to organelles including chloroplasts and mitochondria^26^,^27^. Similarly, significant number of genes belonging to *SiHSP90* and *SiHSP70* have also showed up-regulation in all the tissues as well as in stress. Stress-specific upregulation of *SiHSP60-04*, *SisHSP-07*, *SisHSP-07*, *SisHSP-21*, *SisHSP-22* and *SisHSP-30* was also observed, which suggests the putative involvement of these genes in stress-responsive molecular processes. Few genes did not show any expression in tissues as well as drought stress, which may mean that these genes might have roles in response to other stresses. Nevertheless, further functional characterization is necessary to conclude the putative involvement of these genes in stress-regulatory machinery.

**Figure 5:**
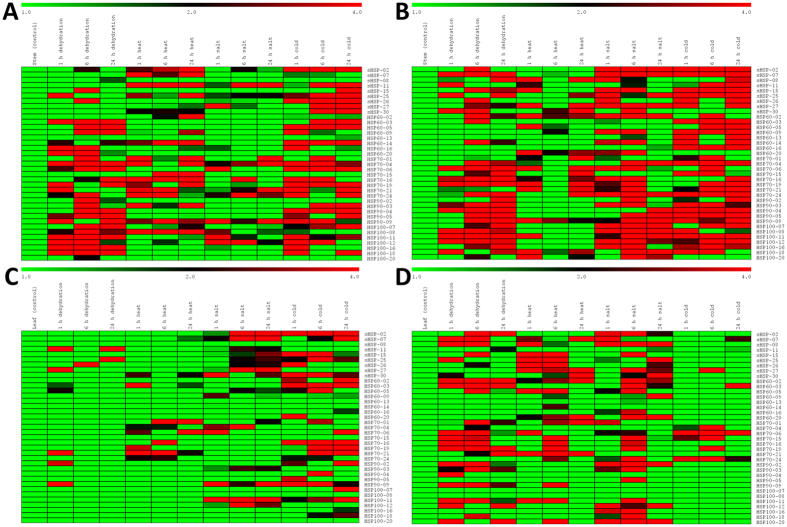
Expression profile of candidate *SiHSP* genes in response to abiotic stress treatments. Heat map showing differential gene expression in response four different stresses (dehydration, heat, salinity and cold) at three time-points (1 h, 6 h and 24 h) in two tissues (stem and leaf) of contrasting foxtail millet cultivars (IC-4 – stress tolerant, and IC-41 – susceptible). **(A)** Susceptible stem, **(B)** Tolerant stem, **(C)** Susceptible leaf, and **(D)** Tolerant leaf. The heat-map has been generated based on the fold-change values in the treated sample when compared with its treated control sample. The color scale for fold-change values is shown at the top, where 1.0, 2.0 and 4.0 denotes low, medium and high expression, respectively.

Based on the RNA-seq derived expression profiles, thirty-seven candidate genes showing stress-specific expression, tissue-specific expression, higher expression in all the four tissues, and/or no expression in all the four tissues were chosen for qRT-PCR analysis. These genes also represent all the five classes of HSP and nine chromosomes of foxtail millet. Expression pattern of these genes in response to four different stresses (dehydration, heat, salinity and cold) at three time-points (1 h, 6 h and 24 h) in two tissues (stem and leaf) of contrasting cultivars (IC-4, stress tolerant; IC-41, susceptible) was examined using qRT-PCR (Fig. 5; Supplementary Table 6). The results showed the differential expression of these genes in response to the stresses. Most of the genes were late responsive to dehydration stress as their expression level reached maximum at 24 h of dehydration treatment. There was distinct differential expression of *SisHSP-02*, *SisHSP-07*, *SisHSP-08* and *SiHSP70-15* gene in leaf as well as stem of susceptible and tolerant cultivars. The study showed that maximum of genes was induced in stem as compared to leaf in response to dehydration treatment in both the cultivars. *SisHSP-27, SisHSP-30, SiHSP60-14, SiHSP70-24, SiHSP90-05, SiHSP100-07, SiHSP100-08* and *SiHSP100-16* were highly in stem compared to leaf, suggesting that *HSP* genes have prominent roles in stem than leaf during dehydration stress.

During heat stress, many genes were observed to be upregulated in both stem and leaf of tolerant cultivar of foxtail millet (Fig. 5). Most of the small heat shock proteins showed higher expression in leaf tissue at early stage of heat stress. *SisHSP-15* (~9–200 fold)*, SisHSP-25* (~28–86 fold)*, SisHSP-26* (~6–20 fold) and *SisHSP-27* (~3–31 fold) were upregulated to several folds after 1 h and 6 h of heat treatment in leaf of tolerant cultivar. Other genes including *SiHSP60-05* (~5–8 fold)*, SiHSP60-20* (~3–17 fold)*, SiHSP70-06* (~4–10 fold)*, SiHSP100-11* (~7–35 fold) and *SiHSP100-20* (~5–6 fold) showed higher expression at early induction of heat in both the tissues of tolerant cultivar. *SisHSP-30* appeared to be a late responsive gene as it is highly expressed (~17–48 fold) after 24 h of heat stress in both leaf and stem of IC-4 cultivar. Although few heat induced genes such as *SiHSP70-06* (up to 19 fold) and *SiHSP100-18* (up to 9 fold) were highly expressed only in stem, the genes *SiHSP70-01, SiHSP70-16, SiHSP70-19, SiHSP70-21* and *SiHSP70-24* were expressed uniformly after heat induction in leaf and stem of both tolerant and susceptible cultivars (Fig. 5).

Expression profiling of *SiHSP* genes in response to salinity stress indicated that most of the genes were induced uniformly in both leaf and stem of tolerant cultivar compared to susceptible cultivar. Interestingly, *SisHSP-02* (5 fold)*, SiHSP60-02* (380 fold)*, SiHSP60-20* (26 fold)*, SiHSP70-16* (87 fold)*, SiHSP70-24* (148 fold)*, SiHSP90-02* (62 fold)*, SiHSP90-03* (57 fold)*, SiHSP100-16* (102 fold)*, SiHSP100-18* (77 fold) and *SiHSP100-20* (164 fold) were highly expressed in both the tissues of tolerant cultivar at all the three time-points (Fig. 5). This is in contrast to the results observed in response to heat and dehydration stresses where most of the genes were upregulated in leaf and stem, respectively. Few genes including *SiHSP60-14* (124 fold)*, SiHSP70-04* (48 fold)*, SiHSP90-09* (55 fold)*, SiHSP100-07* (158 fold) and *SiHSP100-08* (115 fold) were upregulated in stem of IC-4, whereas, *SisHSP-08* (13 fold) and *SiHSP70-15* (14 fold) were highly expressed in leaf of this cultivar.

In response to cold stress, most of the *SiHSP* genes were found to be upregulated in both tolerant and susceptible cultivars, and only a lesser number of genes showed differential expression pattern (Fig. 5). Further, the number of genes induced upon cold treatment in stem is higher than the leaf in tolerant cultivar. Similar pattern was observed in leaf tissues of susceptible cultivar, where the number of upregulated genes were higher than tolerant cultivar. Several genes including *SisHSP-02* (1723 fold)*, SisHSP-07* (489 fold)*, SisHSP-15* (349 fold)*, SisHSP-25* (351 fold)*, SisHSP26* (54 fold)*, SiHSP60-03* (3353 fold)*, SiHSP60-05* (153.5 fold)*, SiHSP70-16* (222 fold)*, SiHSP70-19* (119 fold)*, SiHSP70-21* (55 fold)*, SiHSP70-24* (203 fold)*, SiHSP90-09* (184 fold)*, SiHSP100-07* (167 fold)*, SiHSP100-11* (106 fold) and *SiHSP100-18* (32 fold) were upregulated in both the cultivars in response to cold stress. Few genes including *SisHSP-08, SiHSP60-13* and *SiHSP100-08* were expressed solely in the stem of tolerant cultivar, whereas *SiHSP70-15* is the only gene which showed differential expression pattern in both the tissues of both the cultivars.

Taken together, several genes including *SisHSP-15, SisHSP-25, SisHSP-27, SiHSP60-02, SiHSP70-06, SiHSP70-16, SiHSP70-19, SiHSP70-24, SiHSP90-09, SiHSP100-11, SiHSP100-12* and *SiHSP100-18* showed upregulation in response to abiotic stresses either in leaf or stem or in both tissues. The study suggests that these genes might have the potential to play an important role in abiotic stress-responsive molecular machinery. Importantly, *SisHSP-27* has shown 35-fold upregulation in response to heat stress in tolerant cultivar after one hour of stress treatment (Supplementary Table 6). The relative fold of expression level of *SisHSP-27* in IC-4 heat-stressed leaf sample was ~31 fold, while it was only 0.04 fold in IC-41. In 24 h sample, the relative expression in IC-4 declined to 0.95 and 10.6 folds in stem and leaf samples, respectively, while in the case of IC-41, 1.54 and 0.004-fold expression was observed in stem and leaf, respectively. Based on the qRT-PCR results, *SisHSP-27* was chosen for over-expression and abiotic stress assay in yeast system.

### Heterologous expression of *SisHSP-27* in yeast and methylation status of *SiHSP* genes

**Figure 6:**
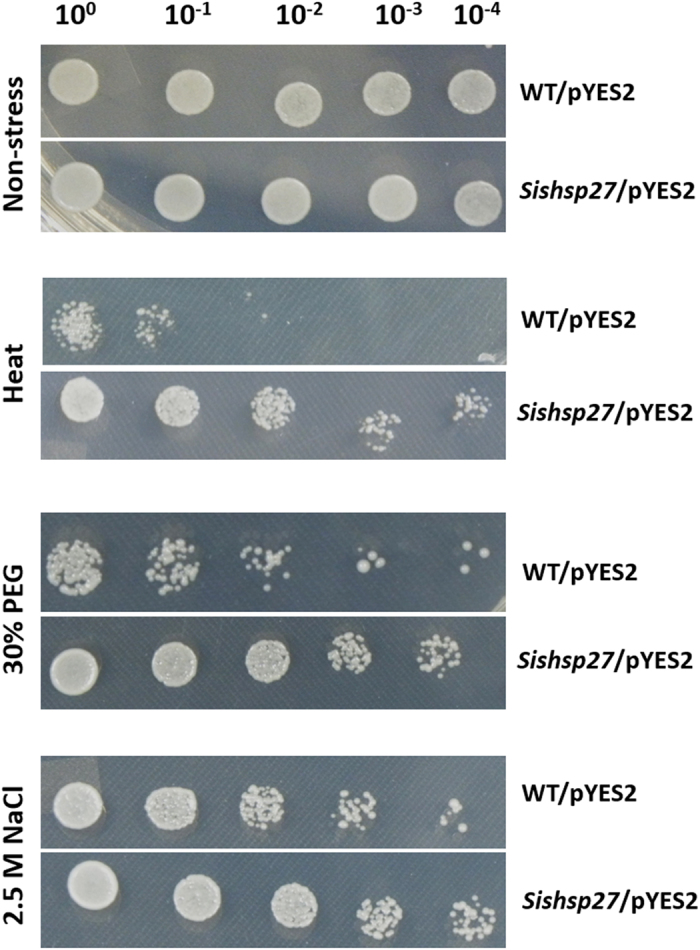
Spot assay of yeast (W303) cells on SD/-ura basal medium. Growth of control pYES2 and *Sishsp27*-pYES2 transformed yeast cells under different stress conditions.

The growth rate of *Sishsp27*-transformed (pYES2-*Sishsp27*) *S. cerevisiae* cells exposed to different abiotic stresses (heat, dehydration and salinity stress) was found to be superior to the growth rate of control (pYES2-0) transformed cells (Fig. 6). pYES2-*Sishsp27* recombinant *S. cerevisiae* cells were able to grow better at elevated temperature (50 °C), in the presence of 2.5 M NaCl and 30% PEG; however, the transformed cells did not show any significant growth in cold (−20 °C) stress. During heat and dehydration stresses, the differential growth rate between pYES2-*Sishsp27* transformed and only control pYES2 transformed yeast cells was significantly higher than salinity stress. These observations indicate that *Sishsp27* was induced upon galactose induction in functionally active yeast cells and improved tolerance to heat, salinity and dehydration. Earlier reports have shown that overexpression of *sHSP* genes enhances the tolerance of plants to abiotic stresses. A small heat shock protein, *limHSP16*.45 from David lily overexpressed in *Arabidopsis* confers tolerance to heat, salinity and oxidative stress^28^. *Arabidopsis* overexpressing *OsHSP18.2* demonstrated high seed vigor, and longevity by reducing ROS accumulation in seed and better performance of seed in germination under abiotic stresses^29^.

**Figure 7:**
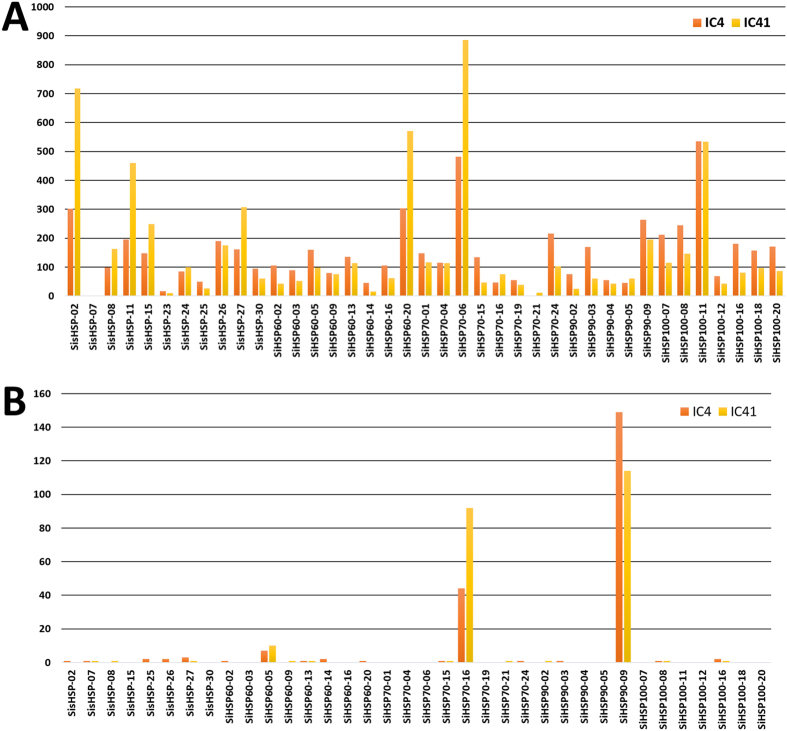
Number of cytosine methylation in *SiHSP* genes which showed differentially expression in response to abiotic stress in foxtail millet. (**A**) Number of cytosine methylation in gene body, (**B**) Number of cytosine methylation in TSS region.

Further, to understand the effect of methylation in regulation of gene expression, a genome-wide DNA methylation study was performed in both IC-4 and IC-41 cultivars (unpublished data). The methylation analysis of *SiHSP* genes showed that the extent of cytosine methylation in genic region was greater than promoter region in both the cultivars under non-stress condition. Further, CpG methylation is most abundant than CHG and CHH methylation in both the cultivars. DNA methylation is an epigenetic mechanism equipped by cells to control gene expression in specific conditions as hyper-methylated genes show lower expression than hypo-methylated genes^30^. Many of the abiotic stress induced *SiHSP* genes showed lesser genic methylation in the tolerant cultivar than the susceptible cultivar (Fig. 7; Supplementary Table S7). For example, *SisHSP-27* was upregulated in response to heat, salinity, cold and dehydration stress in tolerant cultivar. The DNA methylation level in genic region of tolerant cultivar is comparatively less than susceptible cultivar in non-stress conditions. Though the results suggest that the higher methylation in *SiHSP-27* gene in susceptible cultivar might be the reason for their reduced expression in stress conditions, further functional characterization is required to validate this hypothesis.

## Conclusion

The increasing threat of global warming poses serious threat to survival and productivity of crop plants, and therefore, framing appropriate strategies to circumvent these challenges to ensure yield is necessary. Heat and drought are the immediate outcomes of global warming, and plants naturally produce heat shock proteins to perform various molecular and physiological functions in order to withstand the stresses. In view of these, several heat shock protein families have been characterized in many crop plants; however, understanding the structure, organization, evolution and expression pattern of these proteins in a naturally stress tolerant crop would be rewarding. Therefore, the present study was conducted in the C_4_ panicoid model crop, foxtail millet and HSP100, HSP90, HSP70, HSP60 and sHSP proteins and their encoding genes were identified. In addition to several *in silico* analyses, expression profiling of these genes in response to abiotic stresses provided novel clues on putative role of these genes in stress-responsive molecular machinery. Several fold up-regulation of *SisHSP-27* in response to heat and salinity stress in stress tolerant cultivar hinted the role of this gene in conferring stress tolerance. Therefore, the gene was over-expressed in yeast, and interestingly, yeast cells transformed with *SisHSP-27* demonstrated tolerance to several abiotic stresses. Presently, over-expression of this gene in foxtail millet and rice systems is in progress, and if successful, the study will delineate the role of this novel gene in conferring durable stress tolerance.

## Materials and Methods

### Plant materials and stress treatments

Seeds of salt and dehydration tolerant foxtail millet cultivar ‘IC-403579’ (IC-4) and susceptible cultivar ‘IC-480117’ (IC-41) were used in the present study^17^,^20^,^22^. The seeds were grown in a plant growth chamber (PGC-6L; Percival Scientific Inc., USA) under following conditions; 28 ± 1 °C day/23 ± 1 °C night/70 ± 5% relative humidity with a photoperiod of 14 h and a photosynthetic photon flux density of 500 μmol m^−2^ s^−1^. The plants were watered daily with one-third strength Hoagland’s solution^17^. For abiotic stress treatments, 21-day-old seedlings were exposed 250 mM NaCl (salt), 20% PEG 6000 (dehydration), 4 °C (cold) and 45 °C (heat). Stem and leaf tissues were collected at 1 h, 6 h and 24 h post-stress treatments. Untreated tissues were maintained as controls. All the tissues were immediately frozen in liquid nitrogen after harvesting and stored at −80 °C until RNA isolation.

### RNA isolation, cDNA synthesis and qRT-PCR analysis

Total RNA was isolated using Trizol reagent as described by the procedure of Longeman *et al*.^31^, and treated with RNase-free DNase I (50 U/μl; Fermentas, USA). Quality and purity of isolated RNA was checked using NanoDrop 1000 Spectrophotometer (Thermo Scientific, USA) [OD_260_:OD_280_ nm absorption ratio (1.8–2.0)] and the integrity was ascertained by resolving on 1.5% agarose gel containing 18% formaldehyde. One microgram (1 μg) of total RNA was reverse transcribed to first strand cDNA by anchored oligo dT priming and random priming using Thermo Scientific Verso cDNA synthesis kit following manufacturer’s instructions. qRT- PCR analysis was performed in StepOne™ Real-Time PCR Systems (Applied Biosystems, USA) following the reaction profile of Kumar *et al*.^32^ using the primers mentioned in Supplementary Table 8. The experiment was performed in three technical replicates for each biological duplicate. The amount of transcripts accumulated for *SiHSP* genes normalized to the internal control *Act2* was analysed using 2^−ΔΔCt^ method^32^. The PCR efficiency was calculated as: Efficiency = 10^(−1/slope)^ −1 by the default software (Applied Biosystems, USA). Final heat map was generated representing log Ct values for respective gene and tissue using MeV4 software^33^.

### Heterologous expression of *SisHSP-27* in yeast and stress tolerance assay

Full length coding region of *SisHSP-27* gene (640 bp) was PCR amplified from foxtail millet cDNA library using forward (5′-CGGGATCCATGGCCACTGCGTCTAGG; flanked by Bam HI site) and reverse primers (5′-CGGAATTCTCACATCTCGGCTTTGGACG; flanked by Eco RI site), and cloned into pYES2 (Invitrogen, USA). Both pYES2-*SisHSP-27* and pYES2 alone (without insert) were individually transformed into *Saccharomyces cerevisiae* W303 using Yeastmaker Yeast Transformation System (Clontech). Transformants were screened by growth of colonies on SD/-ura medium with 2% (w/v) dextrose at 30 °C for 3 days.

Abiotic stress tolerance studies in transformed yeast cells were performed as described by Li *et al*.^30^ with minor modifications. Yeast cells harboring pYES2-*SisHSP-27* and empty pYES2 vector were incubated in SD/-ura broth medium containing 2% dextrose for 24 h at 30 °C. After incubation, OD_600_ of cultured cells was adjusted to 0.4 to contain an equal number of cells. Around 500 μL of the culture were resuspended in 10 mL of induction medium (SD/-ura broth supplemented with 2% galactose w/v) and incubated at 30 °C for 36 h to promote the expression of *SisHSP-27* gene. After incubation, OD_600_ of the cultures were adjusted to 0.6 and 500 μL of culture was added to 10 mL of SD/-ura medium and incubated at 50 °C in water bath (heat stress) and −20 °C alcohol bath (cold stress) for 24 h. For salinity and dehydration stresses, yeast cells were grown in SD/-ura medium supplemented with 2.5 M NaCl and 30% PEG, respectively, and incubated at 30 °C for 36 h. After stress treatments, cultures were serially diluted (10^0^, 10^−1^, 10^−2^, 10^−3^, 10^−4^) and 7 μL of each diluted cells were spotted on basal SD/-ura medium (supplemented with 2% w/v dextrose) and incubated at 30 °C for 3 days. For control, an equivalent number of unstressed cells suspended in SD/-ura broth were spotted on SD/-ura plates.

### Identification and analysis of genes encoding HSPs (*SiHSPs*) and phylogenetic analysis

Protein sequences of HSPs reported in *Oryza sativa*^6^, *Arabidopsis thaliana*^9^ and *Hordeum vulgare*^12^ were retrieved and HMM profile was prepared individually for each HSP class using HMMER suite^34^. The HMM profiles were then queried against foxtail millet protein database retrieved from Phytozome (https://phytozome.jgi.doe.gov/) with the inclusion threshold 0.01. The proteins falling within this threshold limit were considered as probable HSPs and redundant sequences removed. All predicted proteins were confirmed through HMMSCAN (https://www.ebi.ac.uk/Tools/hmmer/search/hmmscan) and CDD search (http://www.ncbi.nlm.nih.gov/Structure/cdd/wrpsb.cgi). Information regarding gene, transcript, CDS and amino acid sequence of identified HSPs along with their chromosomal locations were retrieved from Phytozome.

All the identified HSPs except small HSPs were annotated with prefix ‘Si’ (*Setaria italica*), suffix ‘100, 90, 70 or 60’ based on their type and numbered according to the ascending order of chromosomal location ranging from short-arm telomere to long-arm telomere. For small HSPs (sHSP), the prefix ‘Si’ and suffix denoting their number was given. The physico-chemical properties of each HSP was performed using ExPASy - ProtParam tool (http://web.expasy.org/protparam/). Gene structure of HSP encoding genes (*SiHSPs*) was predicted using Gene Structure Display Server v2.0 (http://gsds.cbi.pku.edu.cn/). Physical map showing the chromosomal location of *SiHSPs* was constructed using MapChart v2.3^35^.

For phylogenetic analysis, the protein sequences of each class were individually imported into MEGA v6.06^36^, and multiple sequence alignment was performed using ClustalW under default parameters. The alignment file was then used for constructing phylogenetic tree following Neighbor-Joining method using default parameters with 1000 bootstrap iterations.

### Promoter analysis, comparative genome mapping, and duplication and divergence analysis

Two kilobase nucleotide sequences upstream to each *SiHSP* gene were retrieved from Phytozome and analyzed for the presence of *cis*-regulatory elements using PlantCARE database (http://bioinformatics.psb.ugent.be/webtools/plantcare/html/). The gene sequences of physically mapped *SiHSP*s were BLASTN searched against the nucleotide database of sorghum, maize, rice and *Brachypodium* available at Phytozome under default parameters, and hits with 80% homology were chosen for reciprocal BLAST. Significant orthologs were selected for constructing comparative map using Circos v0.55 (http://circos.ca/).

Paralogous gene-pairs that have evolved due to segmental and tandem duplications were analyzed using MCScanX tool^37^. The ratios of non-synonymous (Ka) substitution to synonymous (Ks) substitution of paralogous and orthologous gene-pairs were calculated by PAL2NAL^38^, and time of duplication and divergence was estimated using a synonymous mutation rate of λ substitutions per synonymous site per year as T = Ks/2λ (λ = 6.5 × 10^−9^)^39^.

### RNA-seq derived expression profiling and methylation analysis of *SiHSP* genes

The RNA-seq data of four tissues namely, root (SRX128223), stem (SRX128225), leaf (SRX128224) and spica (SRX128226), and a drought stress library (SRR629694) as well as control (SRR629695) were retrieved from European Nucleotide Archive (http://www.ebi.ac.uk/ena). The reads were processed to generate RPKM following Mishra *et al*.^16^ and heat map was displayed using MeV v4.9^33^.

Total genomic DNA of foxtail millet cultivars ‘IC-403579’ and ‘IC-480117’ were sonicated and the fragmented DNA was end-repaired and ligated with adapters following manufacturer’s instructions (Illumina, San Diego, CA). Sodium bisulfite treatment was given to purified DNA fragments and the sample was PCR amplified using adapter specific primers. The amplified DNA was used to prepare library and sequenced by Illumina Genome Analyzer (GAIIx) according to manufacturer’s instructions. Raw reads were analysed using Bismark tool^40^.

## Additional Information

**How to cite this article**: Singh, R. K. *et al*. Genome-wide analysis of heat shock proteins in C_4_ model, foxtail millet identifies potential candidates for crop improvement under abiotic stress. *Sci. Rep.* **6**, 32641; doi: 10.1038/srep32641 (2016).

## Supplementary Material

Supplementary Information

## Figures and Tables

**Table 1:** Summary of major *cis-*regulatory elements present in promoter region of *SiHSP* genes.

Cis-element	Consensus sequence (5′-3′)	Function	Reference
ABRE3HVA22	GCCACGTACA	Abscisic acid-responsive element	41
ABREBZMRAB28	TCCACGTCTC	Abscisic acid-responsive element in embryos and vegetative tissues	42
ABREDISTBBNNAPA	GCCACTTGTC	Abscisic acid-responsive element and required for seed-specific expression	43
ABREMOTIFAOSOSEM	TACGTGTC	Abscisic acid-responsive element	44
ACGTABOX	TACGTA	Negative regulator of sugar signaling	45
ACGTATERD1	ACGT	A water-stress responsive element	46
ACIPVPAL2	CCCACCTACC	Required for vascular-specific gene expression	47
AGCBOXNPGLB	AGCCGCC	Stress-signaling responsive element	48
AMMORESIIUDCRNIA1	GGWAGGGT	Ammonium responsive and regulate expression of nitrate reductase	49
ANAERO4CONSENSUS	GTTTHGCAA	Involved in regulation of the fermentative pathway	50
ANAERO5CONSENSUS	TTCCCTGTT	Involved in regulation of the fermentative pathway	50
ARR1AT	NGATT	A cytokinin response regulator (RR) binding motif	51
ASF1MOTIFCAMV	TGACG	Auxin-and salicylic acid-responsive element	52
AUXREPSIAA4	KGTCCCAT	Auxine-responsive element	53
BIHD1OS	TGTCA	Binding site for BIHD1, a BELL class homeodomain transcriptional factor responsible for abiotic and biotic stress response	54
CAATBOX1	CAAT	Reported to regulate flowering in plants	55
CACGCAATGMGH3	CACGCAAT	Auxin-responsive element	56
CBFHV	RYCGAC	Dehydration-responsive element	57
CEREGLUBOX1PSLEGA	TGTTAAAGT	Homologous to the cereal glutenin gene control element	58
CURECORECR	GTAC	Regulate copper- and oxygen-responsive *Cyc6* and *Cpx1* expression	59
DOFCOREZM	AAAG	Binding site of Dof transcription factors, which are responsible for plant growth and development as well as stress response	60
E2FAT	TYTCCCGCC	E2F-binding site found in many potential E2F target genes regulating cell cycle	61
EBOXBNNAPA	CANNTG	An E-box sequence, responsible for light responsiveness and is controlled by bHLH and the MYB-transcription factor in regulating tissue-specific expression	62
EMBP1TAEM	CACGTGGC	Involved in ABA-mediated stress-signaling pathway	63
GADOWNAT	ACGTGTC	Gibberellic acid responsive element	64
GATABOX	GATA	Binding site for transcription factors with a zinc finger motif, which have been concerned in light and nitrate-dependent transcription control	65
GCBP2ZMGAPC4	GTGGGCCCG	Binding site of tobacco nuclear factor (GCBP-2) found in the maize (Z.m.) GapC4 (Glyceraldehyde-3-phosphate dehydrogenase 4) gene promoter	66
GT1CONSENSUS	GRWAAW	Recognizes GT-1 proteins, which have tri-helix DNA-binding domains, are conserved in plant nuclear genes and have diverse functions	67
GTGANTG10	GTGA	A pollen-specific *cis*-elements, identified in TCP-enriched genes	68
HY5AT	TGACACGTGGCA	Regulates stimulus-induced development of root and hypocotyl	69
IBOXCORE	GATAA	Light-responsive element	70
LTRE1HVBLT49	CCGAAA	Low temperature-responsive element	71
MNF1ZMPPC1	GTGCCCTT	Light-responsive element	72
MYB2CONSENSUSAT	YAACKG	Dehydration-responsive element	73
MYBCORE	CNGTTR	A binding site for plant MYB transcription factors, which play crucial roles in cell proliferation, differentiation and stress response	74
OCTAMOTIF2	CGCGGCAT	Found in histone-gene-specific consensus sequences; 200 base upstream from the initiation codon ATG	75
OPAQUE2ZMB32	GATGAYRTGG	Binding site of type I ribosome-inactivating protein gene and GARE form a gibberellin response complex	76
PIATGAPB	GTGATCAC	Light-responsive element	77
POLLEN1LELAT52	AGAAA	A regulatory element responsible for pollen-specific activation of gene expression	78
RAV1AAT	CAACA	Rosette leaves- and roots-specific element	79
RGATAOS	CAGAAGATA	Regulator of phloem-specific gene expression	80
RHERPATEXPA7	KCACGW	Root hair-specific cis-elements	81
RYREPEATBNNAPA	CATGCA	Required for seed specific expression	43
SITEIOSPCNA	CCAGGTGG	Regulatory region of PCNA (proliferating cell nuclear antigen)	82
SORLIP1AT	GCCAC	Light responsive element	83
SURE2STPAT21	AATACTAAT	Sucrose regulatory element	84
TRANSINITDICOTS	AMNAUGGC	Context sequence of translational initiation codon in dicots	85
VOZATVPP	GCGTNNNNNNNACGC	Regulate pollen development	86
WBOXNTERF3	TGACY	A W-box promoter motif, functions in response to wound signal	87
WRKY71OS	TGAC	A binding site of rice WRKY71, a transcriptional repressor of the gibberellin signaling pathway	88
