# Supplementary material for: Genome-wide analysis of heat shock proteins in C4 model, foxtail millet identifies potential candidates for crop improvement under abiotic stress
Source: Sci Rep. 2016 Sep 2;6:32641. doi: 10.1038/srep32641 (PMC5009299; doi:10.1038/srep32641)
Supplement: Supplementary Information [file srep32641-s1.pdf]

Genome-wide analysis of heat shock proteins in C_4 model, foxtail millet identifies potential candidates for crop improvement under abiotic stress

Roshan Kumar Singh[#], Jananee Jaishankar[#], Mehanathan Muthamilarasan, Shweta Shweta, Anand Dangi, Manoj Prasad*

National Institute of Plant Genome Research, Aruna Asaf Ali Marg, New Delhi – 110067, India

[#]*These authors contributed equally to this work*

***Corresponding author**

Manoj Prasad

National Institute of Plant Genome Research

Aruna Asaf Ali Marg

New Delhi 110067

India

E-mail: manoj_prasad@nipgr.ac.in

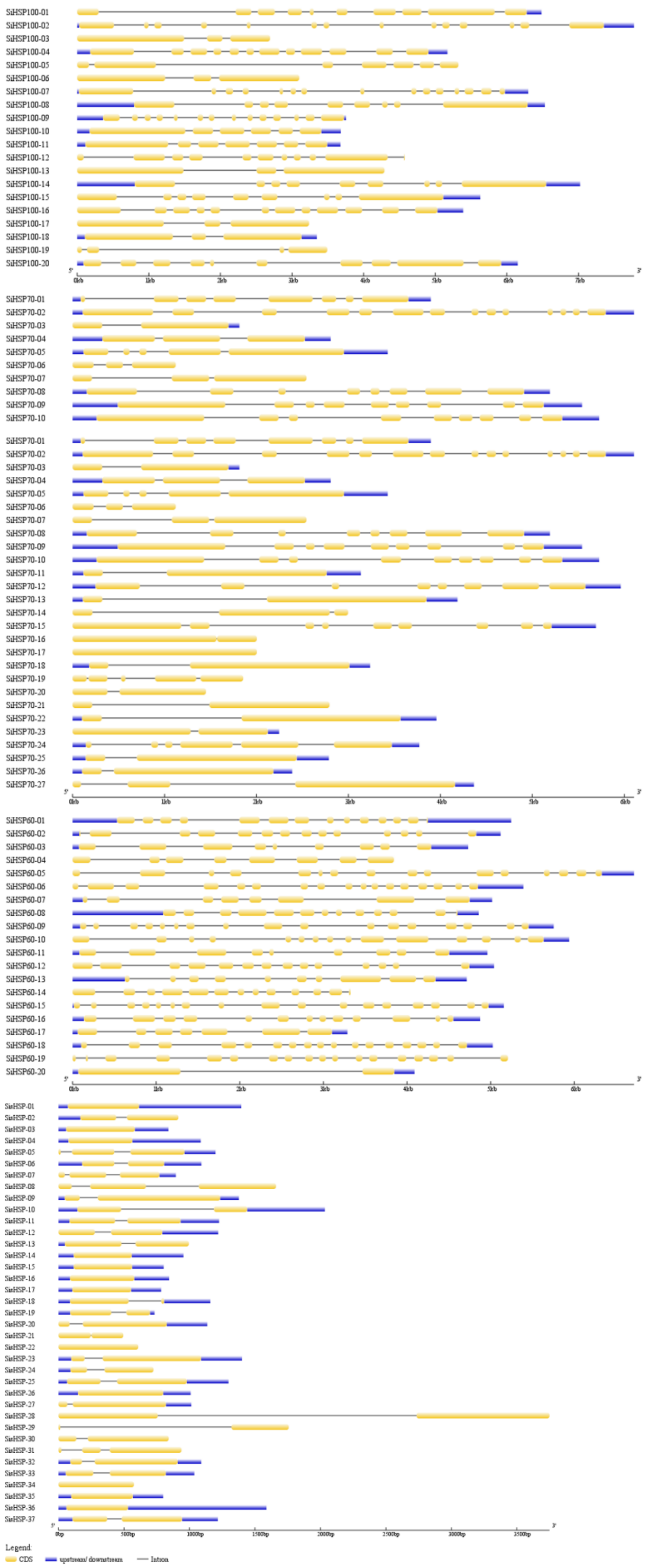

Supplementary Figure 1. Intron-exon positioning of all the 11 *SiHSP* genes.

Supplementary Table 1. Characteristic features of *SiHSP* genes of foxtail millet

SiHSP Class	SiHSP ID	Gene ID (Phytozome)	Alternate transcript ID	Chromosome number	Gene start (bp)	Gene end (bp)	Gene Length	Exons	Introns	Protein length	Mol. Wt (Da)	pI	negatively charged residues	positively charged residues	Instability index	Stability	Aliphatic index	GRAVITY
	SiHSP100-01	Si016214m	Si016215m	1	5798068	5804551	6483	10	9	989	109.4201	6.26	144	138	41.76	unstable	94.73	-0.31
	SiHSP100-02	Si016611m	Si016799m	1	12407911	12415684	7773	14	13	644	69.5513	6.15	77	72	45.46	unstable	90.47	-0.091
	SiHSP100-03	Si019885m		1	21562458	21565150	2692	3	2	753	79.933	5.84	86	75	57.84	unstable	80.21	-0.26
	SiHSP100-04	Si016236m		1	25559590	25564759	5169	12	11	942	102.3201	6.57	125	122	44.96	unstable	94.59	-0.171
	SiHSP100-05	Si016425m		1	26386951	26392274	5323	7	6	752	81.5585	7.19	90	90	41.54	unstable	95.74	-0.136
	SiHSP100-06	Si019205m		1	26480905	26484005	3100	3	2	864	91.8592	7.29	97	97	57.77	unstable	79.8	-0.249
	SiHSP100-07	Si016606m		1	28005200	28011497	6297	15	14	647	68.2841	6.35	78	74	44.34	unstable	82.53	-0.276
	SiHSP100-08	Si021139m		3	5977800	5984329	6529	9	8	921	102.0931	6.62	136	133	47.12	unstable	92.95	-0.317
	SiHSP100-09	Si021813m		3	12275929	12279682	3753	14	13	506	56.5451	8.52	58	63	44.98	unstable	96.36	-0.133
**HSP100**	SiHSP100-10	Si021144m	Si021149m, Si021272m	3	13016068	13019748	3680	6	5	913	101.0185	5.8	146	131	38.32	stable	99.79	-0.319
	SiHSP100-11	Si000232m		5	36658683	36662358	3675	7	6	914	101.0935	5.85	144	129	38.06	stable	99.97	-0.308
	SiHSP100-12	Si015500m		6	6768003	6772578	4575	11	10	781	85.2957	8.27	101	104	39.65	stable	92.57	-0.268
	SiHSP100-13	Si012582m		7	13430728	13435018	4290	3	2	1054	110.5586	5.95	117	99	60.3	unstable	83.31	-0.202
	SiHSP100-14	Si009304m		7	18353032	18360053	7021	9	8	921	102.2001	6.23	140	134	35.12	stable	95.19	-0.324
	SiHSP100-15	Si009306m		7	18363047	18368674	5627	9	8	918	101.7566	6.26	139	133	36.33	stable	95.38	-0.323
	SiHSP100-16	Si009283m		7	19133453	19138842	5389	12	11	942	102.4846	8.5	121	127	43.49	unstable	93.13	-0.176
	SiHSP100-17	Si009348m		7	19676298	19679535	3237	3	2	840	90.0083	8.19	90	95	56.25	unstable	78.81	-0.332
	SiHSP100-18	Si025977m		8	2488989	2492334	3345	3	2	838	90.3707	6.27	91	80	52.12	unstable	77.7	-0.318
	SiHSP100-19	Si039574m		9	8489563	8493054	3491	4	3	279	30.9524	5.93	43	39	35.97	stable	96.42	-0.291
	SiHSP100-20	Si034086m		9	42170787	42176938	6151	10	9	977	108.8432	6.67	147	145	40.27	unstable	90.77	-0.41
	SiHSP90-01	Si032932m		2	6007915	6010199	2284	11	10	414	47.2828	5.23	70	59	52.33	unstable	80.99	-0.483
	SiHSP90-02	Si028990m	Si029016m	2	34600248	34605291	5043	19	18	784	88.9235	4.95	141	109	47.6	unstable	80.1	-0.528
	SiHSP90-03	Si021237m		3	45081885	45088781	6896	20	19	817	91.6034	5.19	134	110	39.08	stable	78.8	-0.51
	SiHSP90-04	Si005877m	Si006128m	4	39628388	39633588	5200	15	14	807	92.7473	4.89	164	121	36.96	stable	79.14	-0.743
**HSP90**	SiHSP90-05	Si013283m		6	31466879	31471644	4765	19	18	788	88.7702	4.97	141	108	47.34	unstable	78.85	-0.529
	SiHSP90-06	Si013561m		6	32063209	32065743	2534	2	1	524	61.2758	5.05	115	90	44.04	unstable	79.96	-0.775
	SiHSP90-07	Si013347m		6	32076788	32080613	3825	3	2	698	80.187	4.96	139	105	39.61	stable	82.39	-0.607
	SiHSP90-08	Si013346m		6	32083265	32087190	3925	3	2	698	80.2581	4.95	139	104	40.22	unstable	81.83	-0.609
	SiHSP90-09	Si009497m		7	1556544	1559316	2772	2	1	709	81.2141	5.02	139	109	39.52	stable	83.22	-0.575
	SiHSP70-01	Si016582m	Si016819m, Si016822m	1	10246401	10250296	3895	8	7	665	73.3319	5.05	115	93	27.78	stable	86.21	-0.479
	SiHSP70-02	Si016270m		1	36152254	36158358	6104	14	13	890	98.2352	5.5	136	117	42.2	unstable	86	-0.422
	SiHSP70-03	Si032984m		2	227828	229641	1813	2	1	424	46.3728	5.68	61	48	30.71	stable	95.02	-0.093
	SiHSP70-04	Si029255m		2	32025020	32027826	2806	3	2	609	65.7844	5.33	88	76	34.46	stable	84.43	-0.387
	SiHSP70-05	Si029041m		2	35804609	35808035	3426	5	4	745	79.7562	8.89	89	96	38.98	stable	84.77	-0.306
	SiHSP70-06	Si022827m		3	650939	652060	1121	3	2	295	32.3299	4.7	59	41	24.88	stable	73.63	-0.678
	SiHSP70-07	Si025202m		3	3180564	3183107	2543	3	2	540	60.3836	5.59	80	67	46.54	unstable	82.5	-0.382
	SiHSP70-08	Si021359m		3	5573789	5578979	5190	8	7	702	74.3701	5.12	100	86	30.14	stable	86.14	-0.262
	SiHSP70-09	Si021202m		3	6988813	6994353	5540	9	8	850	93.8852	5.1	135	106	42.48	unstable	77	-0.487

HSP70	SiHSP70-10	Si021213m	Si021210m, Si021212m, Si021211m	3	7000063	7005788	5725	9	8	845	93.2205	4.98	137	104	38.6	stable	79.35	-0.451
	SiHSP70-11	Si021434m		3	17574222	17577356	3134	2	1	649	71.0124	5.13	100	82	33.49	stable	81.79	-0.409
	SiHSP70-12	Si021389m		3	21467161	21473122	5961	8	7	680	73.0294	5.11	101	85	31.24	stable	85.75	-0.331
	SiHSP70-13	Si021433m		3	41234666	41238851	4185	2	1	649	71.1205	5.13	100	82	34.98	stable	84.19	-0.398
	SiHSP70-14	Si008444m		4	4474303	4477299	2996	3	2	522	57.3394	5.79	71	66	36.65	stable	89.46	-0.22
	SiHSP70-15	Si005920m		4	37951108	37956799	5691	9	8	753	82.9024	5.46	113	91	46.27	unstable	85.55	-0.332
	SiHSP70-16	Si004555m		5	23768496	23770499	2003	2	1	665	73.3077	5.08	112	87	27.19	stable	84.3	-0.447
	SiHSP70-17	Si004526m		5	23871912	23873915	2003	1	0	667	73.4468	5.08	112	87	27.12	stable	83.75	-0.442
	SiHSP70-18	Si000619m		5	41050516	41053751	3235	2	1	648	70.9334	5.1	100	82	33.41	stable	82.65	-0.395
	SiHSP70-19	Si014889m		6	34467838	34469692	1854	5	4	439	47.8438	6.43	53	50	33.66	stable	92.23	-0.052
	SiHSP70-20	Si011909m		7	35472298	35473748	1450	2	1	439	48.0703	5.82	57	49	31.05	stable	94.44	0.013
	SiHSP70-21	Si028356m		8	37819280	37822074	2794	2	1	507	55.8357	5.43	66	56	31.42	stable	87.71	-0.203
	SiHSP70-22	Si034612m		9	1843820	1847776	3956	2	1	648	71.2848	5.06	100	81	35.09	stable	83.84	-0.415
	SiHSP70-23	Si034533m		9	7468266	7470511	2245	2	1	679	74.1269	5.34	112	96	29.76	stable	84.33	-0.405
	SiHSP70-24	Si034537m		9	13463310	13467080	3770	6	5	678	72.6423	5.62	90	83	40.19	unstable	86.37	-0.304
	SiHSP70-25	Si034600m		9	49893761	49896547	2786	2	1	650	71.4119	5.23	100	85	34.2	stable	81.8	-0.442
	SiHSP70-26	Si034604m		9	49939979	49942366	2387	2	1	649	71.1224	5.1	100	82	35.47	stable	81.48	-0.432
	SiHSP70-27	Si034355m		9	52763610	52767974	4364	3	2	763	82.7334	8.53	73	79	47.58	unstable	94.36	-0.031
HSP60	SiHSP60-01	Si016700m	Si016842m	1	179729	184974	5245	14	13	599	63.9223	5.92	78	74	31.05	stable	98.16	-0.126
	SiHSP60-02	Si016800m		1	7299768	7304886	5118	14	13	560	60.1577	6.45	69	67	31.96	stable	93.05	-0.1
	SiHSP60-03	Si029447m		2	589555	594286	4731	9	8	535	59.083	5.47	79	67	42.33	unstable	96.45	-0.208
	SiHSP60-04	Si029308m		2	14956816	14960659	3843	8	7	587	62.6366	6.55	69	67	30.93	stable	110.34	0.105
	SiHSP60-05	Si021480m		3	2403233	2409946	6713	16	15	620	68.3188	7.86	81	83	38.86	stable	94.52	-0.257
	SiHSP60-06	Si021450m		3	11971877	11977268	5391	17	16	637	67.4473	6.23	82	79	34.37	stable	90.71	-0.21
	SiHSP60-07	Si021598m		3	26620668	26625685	5017	8	7	577	61.0947	5.08	82	67	29	stable	104.37	-0.034
	SiHSP60-08	Si006135m	Si006134m, Si006136m	4	699908	704765	4857	13	12	579	61.9209	5.43	81	74	22.24	stable	101.9	-0.094
	SiHSP60-09	Si006193m		4	3925434	3931187	5753	17	16	545	59.1642	5.78	77	69	31.59	stable	103.45	-0.056
	SiHSP60-10	Si006022m		4	27834664	27840602	5938	15	14	655	70.9488	8.96	80	89	50.62	unstable	94.63	-0.209
	SiHSP60-11	Si006215m		4	36307046	36312007	4961	9	8	535	59.1242	5.64	79	68	44.1	unstable	96.8	-0.193
	SiHSP60-12	Si006049m		4	37441166	37446206	5040	14	13	635	68.7736	8.41	79	83	42.93	unstable	88.82	-0.241
	SiHSP60-13	Si001003m	Si001630m	5	5416922	5421635	4713	10	9	525	57.4071	5.57	80	68	33.69	stable	98.32	-0.125
	SiHSP60-14	Si012802m		7	21074009	21077334	3325	13	12	588	63.4638	8.77	73	80	32.36	stable	99.83	-0.078
	SiHSP60-15	Si009761m		7	26596337	26601494	5157	17	16	545	59.2653	5.72	77	69	31.58	stable	104.17	-0.061
	SiHSP60-16	Si026187m		8	30233760	30238633	4873	13	12	547	58.9075	5.29	77	62	37.12	stable	98.08	-0.036
	SiHSP60-17	Si034840m		9	187201	190487	3286	7	6	586	61.6463	5.31	80	69	32.71	stable	101.3	-0.043
	SiHSP60-18	Si034887m		9	17442926	17447951	5025	17	16	576	60.8081	5.84	77	73	26.49	stable	98.25	-0.04
	SiHSP60-19	Si034915m		9	56420402	56425609	5207	18	17	570	60.4656	5.62	76	71	24.17	stable	98.09	-0.066
	SiHSP60-20	Si035102m		9	58126455	58130544	4089	2	1	534	57.5826	6.78	66	65	39.44	stable	109.83	0.06
	SisHSP-01	Si018457m		1	3123887	3125282	1395	1	0	180	19.5903	6.63	26	26	47.02	unstable	71.5	-0.544
	SisHSP-02	Si018295m		1	4474502	4475416	914	2	1	221	23.5343	6.93	30	30	47.7	unstable	83.53	-0.35
	SisHSP-03	Si018487m		1	8792291	8793129	838	1	0	174	18.8414	6.85	23	23	43.94	unstable	82.99	-0.36

	SisHSP-04	Si018542m		1	36169044	36170129	1085	1	0	162	17.668	7.78	26	27	28.5	stable	65.68	-0.538
	SisHSP-05	Si018171m	Si018187m, Si018401m	1	38572788	38573986	1198	3	2	250	27.7195	9.61	35	43	48.01	unstable	73.32	-0.648
	SisHSP-06	Si018495m		1	39834413	39835503	1090	2	1	173	18.6349	6.71	26	25	42.44	unstable	77.75	-0.565
	SisHSP-07	Si031104m		2	41831224	41832119	895	3	2	209	23.032	8.15	22	23	54.09	unstable	78.09	-0.39
	SisHSP-08	Si024825m		3	2125364	2127024	1660	3	2	371	40.144	10.17	38	61	73.29	unstable	56.66	-0.824
	SisHSP-09	Si022520m		3	8276444	8277820	1376	2	1	349	37.5112	9.14	56	62	49.67	unstable	59.83	-0.887
	SisHSP-10	Si023317m		3	14350334	14352367	2033	2	1	195	21.5692	4.9	32	24	49.84	unstable	73.23	-0.292
	SisHSP-11	Si007151m		4	6236824	6238049	1225	2	1	251	26.9538	6.35	34	33	37.19	stable	84.7	-0.271
	SisHSP-12	Si007239m		4	7456674	7457892	1218	2	1	222	24.3394	4.85	42	32	47.8	unstable	74.82	-0.44
	SisHSP-13	Si007053m		4	7475921	7476915	994	2	1	279	29.3757	5.43	36	31	48.63	unstable	93.23	-0.134
	SisHSP-14	Si007440m		4	10433864	10434817	953	1	0	147	15.9531	8.13	20	21	36.55	stable	75.58	-0.322
	SisHSP-15	Si003245m		5	7897990	7898792	802	1	0	148	16.2334	5.95	24	23	37.59	stable	85.54	-0.359
	SisHSP-16	Si003160m		5	7908007	7908850	843	1	0	163	17.1862	4.74	24	15	42.84	unstable	86.38	0.022
	SisHSP-17	Si003242m		5	7909852	7910635	783	1	0	149	16.748	6.77	27	27	51.56	unstable	75.84	-0.572
	SisHSP-18	Si003202m		5	7912355	7913513	1158	2	1	156	17.4867	5.81	29	27	51.41	unstable	73.65	-0.593
**sHSP**	SisHSP-19	Si003151m	Si003231m	5	7923272	7924004	732	2	1	165	19.207	8.7	23	26	57.03	unstable	80.79	-0.37
	SisHSP-20	Si002731m		5	7924595	7925730	1135	2	1	241	27.1106	9.12	32	36	63.9	unstable	69.96	-0.67
	SisHSP-21	Si005062m		5	11220808	11221302	494	2	1	162	17.5582	5.95	27	26	40.32	unstable	77.1	-0.372
	SisHSP-22	Si004066m		5	11223563	11224171	608	1	0	202	22.2164	7.98	34	35	44.84	unstable	71.49	-0.644
	SisHSP-23	Si002482m		5	31718600	31720000	1400	2	1	284	30.5762	8.79	46	49	40.1	unstable	66.55	-0.792
	SisHSP-24	Si003145m		5	31740859	31741583	724	2	1	166	17.4429	8.64	17	19	15.99	stable	80.72	-0.075
	SisHSP-25	Si014289m	Si014309m	6	20972495	20973792	1297	2	1	262	28.8012	8.99	35	37	41.78	unstable	67.75	-0.621
	SisHSP-26	Si011036m		7	21161295	21162303	1008	1	0	216	23.5896	5.52	37	32	43.31	unstable	80.88	-0.376
	SisHSP-27	Si026764m		8	8605725	8606760	1035	1	0	213	22.715	6.35	31	30	45.15	unstable	82.91	-0.228
	SisHSP-28	Si039412m		9	9816080	9819828	3748	2	1	591	63.2028	5.41	90	74	76.71	unstable	57.77	-0.804
	SisHSP-29	Si039906m		9	9820171	9821928	1757	2	1	149	16.6546	4.93	26	19	61.43	unstable	73.22	-0.654
	SisHSP-30	Si038571m		9	9826133	9826973	840	2	1	250	27.251	9.11	34	39	57.99	unstable	73.76	-0.637
	SisHSP-31	Si038857m		9	19017777	19018715	938	3	2	236	25.3025	5.6	34	26	54.69	unstable	83.81	-0.313
	SisHSP-32	Si037243m		9	19020419	19021508	1089	2	1	241	25.899	6.61	33	32	49.78	unstable	73.28	-0.514
	SisHSP-33	Si037486m		9	31685191	31686227	1036	2	1	212	22.725	9.51	24	31	56.02	unstable	77.83	-0.423
	SisHSP-34	Si038751m		9	50454063	50454638	575	1	0	191	21.4991	8.18	27	28	61.86	unstable	65.29	-0.771
	SisHSP-35	Si037867m		9	50484374	50485172	798	1	0	156	17.4925	5.53	25	22	50.2	unstable	65.58	-0.644
	SisHSP-36	Si037871m		9	50485555	50487142	1587	1	0	156	17.5868	5.82	26	24	57.91	unstable	68.65	-0.678
	SisHSP-37	Si037240m		9	51325204	51326419	1215	2	1	241	26.6954	6.22	38	37	53.74	unstable	71.66	-0.538

Supplementary Table 2. The Ka/Ks ratios and estimated divergence time for tandemly and segmentally duplicated *SiHSP* genes.

Duplication type	Gene 1	Chr	Start	End	Gene 2	Chr	Start	End	Distance (bp)	Ka	Ks	Ka/Ks	Time of divergence (MYA)
**Tandem**	SisHSP-12	4	7456674	7457892	SisHSP-13	4	7475921	7476915	18029	0.06	0.34	0.18	26.2
	SisHSP-35	9	50484374	50485172	SisHSP-36	9	50485555	50487142	383	0.03	0.32	0.09	24.6
	SiHSP70-16	5	23768496	23770499	SiHSP70-17	5	23871912	23873915	101413	0.04	0.33	0.12	25.4
	SiHSP90-07	6	32076788	32080613	SiHSP90-08	6	32083265	32087190	2652	0.05	0.34	0.15	26.2
								**Mean**	**30619.25**	**0.05**	**0.33**	**0.14**	**25.60**

Duplication type	Gene 1	Chr	Start	End	Gene 2	Chr	Start	End	Ka	Ks	Ka/Ks	Time of divergence (MYA)
**Segmental**	SisHSP-22	5	11223563	11224171	SisHSP-34	9	50454063	50454638	0.03	0.3	0.1	23.1
	SiHSP60-03	2	589555	594286	SiHSP60-11	4	36307046	36312007	0.02	0.31	0.06	23.9
	SiHSP90-02	2	34600248	34605291	SiHSP90-05	6	31466879	31471644	0.04	0.29	0.14	22.3
	SiHSP100-10	3	13016068	13019748	SiHSP100-11	5	36658683	36662358	0.04	0.31	0.13	23.9
								**Mean**	**0.03**	**0.30**	**0.11**	**23.30**

Supplementary Table 3. Summary of domains present in SiHSP proteins

SiHSP Class	SiHSP ID	AAA (PF00004.26)		AAA_2 (PF07724.11)		AAA_5 (PF07728.11)		Sigma54_activat (PF00158.23)		Clp_N (PF02861.17)		ClpB_D2-small (PF10431.6)		UVR (PF02151.16)		HSP90 (PF00183.15)		HATPase_c (PF02518.23)		HATPase_c_3 (PF13589.3)		HSP70 (PF00012.17)		MreB_Mbl (PF06723.10)		Cpn60_TCP1 (PF00118.21)		HSP20 (PF00011.18)		CS (PF04969.13)	
		Start	End	Start	End	Start	End	Start	End	Start	End	Start	End	Start	End	Start	End	Start	End	Start	End	Start	End	Start	End	Start	End	Start	End	Start	End	Start	End
HSP100	SiHSP100-01	302	439	700	870	704	844	674	836	117	169	876	956	-	-	-	-	-	-	-	-	-	-	-	-	-	-	-	-	-	-	-	-
		705	844	-	-	-	-	-	-	194	245	-	-	-	-	-	-	-	-	-	-	-	-	-	-	-	-	-	-	-	-	-	-
	SiHSP100-02	208	360	203	409	-	-	-	-	-	-	415	500	-	-	-	-	-	-	-	-	-	-	-	-	-	-	-	-	-	-	-	-
	SiHSP100-03	-	-	-	-	-	-	-	-	23	64	-	-	-	-	-	-	-	-	-	-	-	-	-	-	-	-	-	-	-	-	-	-
		-	-	-	-	-	-	-	-	188	227	-	-	-	-	-	-	-	-	-	-	-	-	-	-	-	-	-	-	-	-	-	-
	SiHSP100-04	169	293	451	616	455	586	-	-	-	-	622	702	-	-	-	-	-	-	-	-	-	-	-	-	-	-	-	-	-	-	-	-
		456	594	-	-	-	-	-	-	-	-	-	-	-	-	-	-	-	-	-	-	-	-	-	-	-	-	-	-	-	-	-	-
	SiHSP100-05	306	447	655	829	659	791	629	789	85	129	835	915	-	-	-	-	-	-	-	-	-	-	-	-	-	-	-	-	-	-	-	-
		660	790	-	-	-	-	-	-	169	220	-	-	-	-	-	-	-	-	-	-	-	-	-	-	-	-	-	-	-	-	-	-
	SiHSP100-06	-	-	-	-	-	-	-	-	23	64	-	-	-	-	-	-	-	-	-	-	-	-	-	-	-	-	-	-	-	-	-	-
		-	-	-	-	-	-	-	-	188	227	-	-	-	-	-	-	-	-	-	-	-	-	-	-	-	-	-	-	-	-	-	-
	SiHSP100-07	299	454	294	500	298	381	-	-	-	-	506	591	-	-	-	-	-	-	-	-	-	-	-	-	-	-	-	-	-	-	-	-
	SiHSP100-08	295	429	633	808	294	402	607	765	105	157	814	894	508	543	-	-	-	-	-	-	-	-	-	-	-	-	-	-	-	-	-	-
		638	768	-	-	637	773	-	-	180	232	-	-	-	-	-	-	-	-	-	-	-	-	-	-	-	-	-	-	-	-	-	-
	SiHSP100-09	125	260	120	325	124	204	-	-	-	-	331	414	-	-	-	-	-	-	-	-	-	-	-	-	-	-	-	-	-	-	-	-
	SiHSP100-10	205	343	598	763	602	738	576	735	17	68	769	849	-	-	-	-	-	-	-	-	-	-	-	-	-	-	-	-	-	-	-	-
		603	742	-	-	-	-	-	-	98	148	-	-	-	-	-	-	-	-	-	-	-	-	-	-	-	-	-	-	-	-	-	-
	SiHSP100-11	205	343	598	763	602	740	573	734	17	62	769	849	-	-	-	-	-	-	-	-	-	-	-	-	-	-	-	-	-	-	-	-
		603	736	-	-	-	-	-	-	99	149	-	-	-	-	-	-	-	-	-	-	-	-	-	-	-	-	-	-	-	-	-	-
	SiHSP100-12	525	650	520	685	524	655	494	652	-	-	691	771	438	473	-	-	-	-	-	-	-	-	-	-	-	-	-	-	-	-	-	-
	SiHSP100-13	-	-	-	-	-	-	-	-	23	54	-	-	-	-	-	-	-	-	-	-	-	-	-	-	-	-	-	-	-	-	-	-
		-	-	-	-	-	-	-	-	188	227	-	-	-	-	-	-	-	-	-	-	-	-	-	-	-	-	-	-	-	-	-	-
	SiHSP100-14	295	429	-	-	294	425	607	766	105	157	814	894	508	543	-	-	-	-	-	-	-	-	-	-	-	-	-	-	-	-	-	-
		638	769	-	-	637	770	-	-	180	232	-	-	-	-	-	-	-	-	-	-	-	-	-	-	-	-	-	-	-	-	-	-
	SiHSP100-15	292	426	630	805	291	422	604	763	102	154	811	891	505	540	-	-	-	-	-	-	-	-	-	-	-	-	-	-	-	-	-	-
		635	766	-	-	634	767	-	-	177	229	-	-	-	-	-	-	-	-	-	-	-	-	-	-	-	-	-	-	-	-	-	-
	SiHSP100-16	305	447	654	829	658	791	628	787	90	140	835	915	-	-	-	-	-	-	-	-	-	-	-	-	-	-	-	-	-	-	-	-
		659	789	-	-	-	-	-	-	168	215	-	-	-	-	-	-	-	-	-	-	-	-	-	-	-	-	-	-	-	-	-	-
	SiHSP100-17	-	-	-	-	-	-	-	-	23	55	-	-	-	-	-	-	-	-	-	-	-	-	-	-	-	-	-	-	-	-	-	-
		-	-	-	-	-	-	-	-	137	175	-	-	-	-	-	-	-	-	-	-	-	-	-	-	-	-	-	-	-	-	-	-
	SiHSP100-18	-	-	-	-	-	-	-	-	23	54	-	-	-	-	-	-	-	-	-	-	-	-	-	-	-	-	-	-	-	-	-	-
		-	-	-	-	-	-	-	-	142	182	-	-	-	-	-	-	-	-	-	-	-	-	-	-	-	-	-	-	-	-	-	-
	SiHSP100-19	-	-	15	71	-	-	-	-	-	-	176	257	-	-	-	-	-	-	-	-	-	-	-	-	-	-	-	-	-	-	-	-
		-	-	83	164	-	-	-	-	-	-	-	-	-	-	-	-	-	-	-	-	-	-	-	-	-	-	-	-	-	-	-	-
	SiHSP100-20	285	422	683	852	687	821	657	816	100	152	858	938	-	-	-	-	-	-	-	-	-	-	-	-	-	-	-	-	-	-	-	-
		688	815	-	-	-	-	-	-	177	228	-	-	-	-	-	-	-	-	-	-	-	-	-	-	-	-	-	-	-	-	-	-
HSP90	SiHSP90-01	-	-	-	-	-	-	-	-	-	-	-	-	-	-	43	177	-	-	-	-	-	-	-	-	-	-	-	-	-	-	-	-
		-	-	-	-	-	-	-	-	-	-	-	-	-	-	169	396	-	-	-	-	-	-	-	-	-	-	-	-	-	-	-	-
	SiHSP90-02	-	-	-	-	-	-	-	-	-	-	-	-	-	-	260	765	95	257	93	250	-	-	-	-	-	-	-	-	-	-	-	-
	SiHSP90-03	-	-	-	-	-	-	-	-	-	-	-	-	-	-	286	784	122	284	120	272	-	-	-	-	-	-	-	-	-	-	-	-
	SiHSP90-04	-	-	-	-	-	-	-	-	-	-	-	-	-	-	259	796	100	257	98	254	-	-	-	-	-	-	-	-	-	-	-	-
	SiHSP90-05	-	-	-	-	-	-	-	-	-	-	-	-	-	-	265	769	100	262	98	255	-	-	-	-	-	-	-	-	-	-	-	-
	SiHSP90-06	-	-	-	-	-	-	-	-	-	-	-	-	-	-	11	522	-	-	-	-	-	-	-	-	-	-	-	-	-	-	-	-
	SiHSP90-07	-	-	-	-	-	-	-	-	-	-	-	-	-	-	185	696	28	183	26	161	-	-	-	-	-	-	-	-	-	-	-	-
	SiHSP90-08	-	-	-	-	-	-	-	-	-	-	-	-	-	-	185	696	28	183	26	158	-	-	-	-	-	-	-	-	-	-	-	-
	SiHSP90-09	-	-	-	-	-	-	-	-	-	-	-	-	-	-	195	707	37	193	35	165	-	-	-	-	-	-	-	-	-	-	-	-
HSP70	SiHSP70-01	-	-	-	-	-	-	-	-	-	-	-	-	-	-	-	-	-	-	-	-	35	642	135	408	-	-	-	-	-	-	-	-
	SiHSP70-02	-	-	-	-	-	-	-	-	-	-	-	-	-	-	-	-	-	-	-	-	28	740	128	421	-	-	-	-	-	-	-	-
	SiHSP70-03	-	-	-	-	-	-	-	-	-	-	-	-	-	-	-	-	-	-	-	-	39	420	132	421	-	-	-	-	-	-	-	-
	SiHSP70-04	-	-	-	-	-	-	-	-	-	-	-	-	-	-	-	-	-	-	-	-	1	581	97	357	-	-	-	-	-	-	-	-
	SiHSP70-05	-	-	-	-	-	-	-	-	-	-	-	-	-	-	-	-	-	-	-	-	121	715	231	491	-	-	-	-	-	-	-	-
	SiHSP70-06	-	-	-	-	-	-	-	-	-	-	-	-	-	-	-	-	-	-	-	-	1	280	-	-	-	-	-	-	-	-	-	-
	SiHSP70-07	-	-	-	-	-	-	-	-	-	-	-	-	-	-	-	-	-	-	-	-	8	198	-	-	-	-	-	-	-	-	-	-
		-	-	-	-	-	-	-	-	-	-	-	-	-	-	-	-	-	-	-	-	207	384	-	-	-	-	-	-	-	-	-	-
		-	-	-	-	-	-	-	-	-	-	-	-	-	-	-	-	-	-	-	-	381	540	-	-	-	-	-	-	-	-	-	-
	SiHSP70-08	-	-	-	-	-	-	-	-	-	-	-	-	-	-	-	-	-	-	-	-	68	663	123	440	-	-	-	-	-	-	-	-
	SiHSP70-09	-	-	-	-	-	-	-	-	-	-	-	-	-	-	-	-	-	-	-	-	3	692	113	381	-	-	-	-	-	-	-	-
	SiHSP70-10	-	-	-	-	-	-	-	-	-	-	-	-	-	-	-	-	-	-	-	-	3	690	113	381	-	-	-	-	-	-	-	-
	SiHSP70-11	-	-	-	-	-	-	-	-	-	-	-	-	-	-	-	-	-	-	-	-	8	617	117	386	-	-	-	-	-	-	-	-
	SiHSP70-12	-	-	-	-	-	-	-	-	-	-	-	-	-	-	-	-	-	-	-	-	47	642	131	421	-	-	-	-	-	-	-	-
	SiHSP70-13	-	-	-	-	-	-	-	-	-	-	-	-	-	-	-	-	-	-	-	-	9	618	118	387	-	-	-	-	-	-	-	-
	SiHSP70-14	-	-	-	-	-	-	-	-	-	-	-	-	-	-	-	-	-	-	-	-	10	522	119	395	-	-	-	-	-	-	-	-
	SiHSP70-15	-	-	-	-	-	-	-	-	-	-	-	-	-	-	-	-	-	-	-	-	3	647	117	386	-	-	-	-	-	-	-	-
	SiHSP70-16	-	-	-	-	-	-	-	-	-	-	-	-	-	-	-	-	-	-	-	-	40	646	134	416	-	-	-	-	-	-	-	-
	SiHSP70-17	-	-	-	-	-	-	-	-	-	-	-	-	-	-	-	-	-	-	-	-	40	648	134	416	-	-	-	-	-	-	-	-
	SiHSP70-18	-	-	-	-	-	-	-	-	-	-	-	-	-	-	-	-	-	-	-	-	8	617	118	386	-	-	-	-	-	-	-	-
	SiHSP70-19	-	-	-	-	-	-	-	-	-	-	-	-	-	-	-	-	-	-	-	-	52	437	155	438	-	-	-	-	-	-	-	-
	SiHSP70-20	-	-	-	-	-	-	-	-	-	-	-	-	-	-	-	-	-	-	-	-	58	436	138	438	-	-	-	-	-	-	-	-
	SiHSP70-21	-	-	-	-	-	-	-	-	-	-	-	-	-	-	-	-	-	-	-	-	9	507	111	392	-	-	-	-	-	-	-	-
	SiHSP70-22	-	-	-	-	-	-	-	-	-	-	-	-	-	-	-	-	-	-	-	-	10	619	120	388	-	-	-	-	-	-	-	-
	SiHSP70-23	-	-	-	-	-	-	-	-	-	-	-	-	-	-	-	-	-	-	-	-	44	658	139	419	-	-	-	-	-	-	-	-
	SiHSP70-24	-	-	-	-	-	-	-	-	-	-	-	-	-	-	-	-	-	-	-	-	54	649	165	425	-	-	-	-	-	-	-	-

	SiHSP70-25	-	-	-	-	-	-	-	-	-	-	-	-	-	-	-	-	-	-	-	-	9	618	118	387	-	-	-	-	-	-
	SiHSP70-26	-	-	-	-	-	-	-	-	-	-	-	-	-	-	-	-	-	-	-	-	9	618	118	387	-	-	-	-	-	-
	SiHSP70-27	-	-	-	-	-	-	-	-	-	-	-	-	-	-	-	-	-	-	-	-	213	718	314	600	-	-	-	-	-	-
**HSP60**	SiHSP60-01	-	-	-	-	-	-	-	-	-	-	-	-	-	-	-	-	-	-	-	-	-	-	-	-	76	580	-	-	-	-
	SiHSP60-02	-	-	-	-	-	-	-	-	-	-	-	-	-	-	-	-	-	-	-	-	-	-	-	-	35	528	-	-	-	-
	SiHSP60-03	-	-	-	-	-	-	-	-	-	-	-	-	-	-	-	-	-	-	-	-	-	-	-	-	40	534	-	-	-	-
	SiHSP60-04	-	-	-	-	-	-	-	-	-	-	-	-	-	-	-	-	-	-	-	-	-	-	-	-	60	563	-	-	-	-
	SiHSP60-05	-	-	-	-	-	-	-	-	-	-	-	-	-	-	-	-	-	-	-	-	-	-	-	-	114	614	-	-	-	-
	SiHSP60-06	-	-	-	-	-	-	-	-	-	-	-	-	-	-	-	-	-	-	-	-	-	-	-	-	116	618	-	-	-	-
	SiHSP60-07	-	-	-	-	-	-	-	-	-	-	-	-	-	-	-	-	-	-	-	-	-	-	-	-	58	561	-	-	-	-
	SiHSP60-08	-	-	-	-	-	-	-	-	-	-	-	-	-	-	-	-	-	-	-	-	-	-	-	-	57	561	-	-	-	-
	SiHSP60-09	-	-	-	-	-	-	-	-	-	-	-	-	-	-	-	-	-	-	-	-	-	-	-	-	31	538	-	-	-	-
	SiHSP60-10	-	-	-	-	-	-	-	-	-	-	-	-	-	-	-	-	-	-	-	-	-	-	-	-	130	626	-	-	-	-
	SiHSP60-11	-	-	-	-	-	-	-	-	-	-	-	-	-	-	-	-	-	-	-	-	-	-	-	-	40	534	-	-	-	-
	SiHSP60-12	-	-	-	-	-	-	-	-	-	-	-	-	-	-	-	-	-	-	-	-	-	-	-	-	110	603	-	-	-	-
	SiHSP60-13	-	-	-	-	-	-	-	-	-	-	-	-	-	-	-	-	-	-	-	-	-	-	-	-	28	518	-	-	-	-
	SiHSP60-14	-	-	-	-	-	-	-	-	-	-	-	-	-	-	-	-	-	-	-	-	-	-	-	-	54	559	-	-	-	-
	SiHSP60-15	-	-	-	-	-	-	-	-	-	-	-	-	-	-	-	-	-	-	-	-	-	-	-	-	31	538	-	-	-	-
	SiHSP60-16	-	-	-	-	-	-	-	-	-	-	-	-	-	-	-	-	-	-	-	-	-	-	-	-	39	530	-	-	-	-
	SiHSP60-17	-	-	-	-	-	-	-	-	-	-	-	-	-	-	-	-	-	-	-	-	-	-	-	-	66	569	-	-	-	-
	SiHSP60-18	-	-	-	-	-	-	-	-	-	-	-	-	-	-	-	-	-	-	-	-	-	-	-	-	56	559	-	-	-	-
	SiHSP60-19	-	-	-	-	-	-	-	-	-	-	-	-	-	-	-	-	-	-	-	-	-	-	-	-	54	557	-	-	-	-
	SiHSP60-20	-	-	-	-	-	-	-	-	-	-	-	-	-	-	-	-	-	-	-	-	-	-	-	-	38	534	-	-	-	-
**sHSP**	SisHSP-01	-	-	-	-	-	-	-	-	-	-	-	-	-	-	-	-	-	-	-	-	-	-	-	-	-	-	52	173	-	-
	SisHSP-02	-	-	-	-	-	-	-	-	-	-	-	-	-	-	-	-	-	-	-	-	-	-	-	-	-	-	120	221	-	-
	SisHSP-03	-	-	-	-	-	-	-	-	-	-	-	-	-	-	-	-	-	-	-	-	-	-	-	-	-	-	70	173	-	-
	SisHSP-04	-	-	-	-	-	-	-	-	-	-	-	-	-	-	-	-	-	-	-	-	-	-	-	-	-	-	28	150	-	-
	SisHSP-05	-	-	-	-	-	-	-	-	-	-	-	-	-	-	-	-	-	-	-	-	-	-	-	-	-	-	152	250	-	-
	SisHSP-06	-	-	-	-	-	-	-	-	-	-	-	-	-	-	-	-	-	-	-	-	-	-	-	-	-	-	59	171	-	-
	SisHSP-07	-	-	-	-	-	-	-	-	-	-	-	-	-	-	-	-	-	-	-	-	-	-	-	-	-	-	94	193	-	-
	SisHSP-08	-	-	-	-	-	-	-	-	-	-	-	-	-	-	-	-	-	-	-	-	-	-	-	-	-	-	69	152	-	-
	SisHSP-09	-	-	-	-	-	-	-	-	-	-	-	-	-	-	-	-	-	-	-	-	-	-	-	-	-	-	23	112	-	-
	SisHSP-10	-	-	-	-	-	-	-	-	-	-	-	-	-	-	-	-	-	-	-	-	-	-	-	-	-	-	96	194	-	-
	SisHSP-11	-	-	-	-	-	-	-	-	-	-	-	-	-	-	-	-	-	-	-	-	-	-	-	-	-	-	153	251	-	-
	SisHSP-12	-	-	-	-	-	-	-	-	-	-	-	-	-	-	-	-	-	-	-	-	-	-	-	-	-	-	125	221	-	-
	SisHSP-13	-	-	-	-	-	-	-	-	-	-	-	-	-	-	-	-	-	-	-	-	-	-	-	-	-	-	181	279	-	-
	SisHSP-14	-	-	-	-	-	-	-	-	-	-	-	-	-	-	-	-	-	-	-	-	-	-	-	-	-	-	34	144	-	-
	SisHSP-15	-	-	-	-	-	-	-	-	-	-	-	-	-	-	-	-	-	-	-	-	-	-	-	-	-	-	44	147	-	-
	SisHSP-16	-	-	-	-	-	-	-	-	-	-	-	-	-	-	-	-	-	-	-	-	-	-	-	-	-	-	45	145	-	-
	SisHSP-17	-	-	-	-	-	-	-	-	-	-	-	-	-	-	-	-	-	-	-	-	-	-	-	-	-	-	45	148	-	-
	SisHSP-18	-	-	-	-	-	-	-	-	-	-	-	-	-	-	-	-	-	-	-	-	-	-	-	-	-	-	46	149	-	-
	SisHSP-19	-	-	-	-	-	-	-	-	-	-	-	-	-	-	-	-	-	-	-	-	-	-	-	-	-	-	47	112	-	-
	SisHSP-20	-	-	-	-	-	-	-	-	-	-	-	-	-	-	-	-	-	-	-	-	-	-	-	-	-	-	137	240	-	-
	SisHSP-21	-	-	-	-	-	-	-	-	-	-	-	-	-	-	-	-	-	-	-	-	-	-	-	-	-	-	59	160	-	-
	SisHSP-22	-	-	-	-	-	-	-	-	-	-	-	-	-	-	-	-	-	-	-	-	-	-	-	-	-	-	97	200	-	-
	SisHSP-23	-	-	-	-	-	-	-	-	-	-	-	-	-	-	-	-	-	-	-	-	-	-	-	-	-	-	19	113	-	-
	SisHSP-24	-	-	-	-	-	-	-	-	-	-	-	-	-	-	-	-	-	-	-	-	-	-	-	-	-	-	27	111	-	-
	SisHSP-25	-	-	-	-	-	-	-	-	-	-	-	-	-	-	-	-	-	-	-	-	-	-	-	-	-	-	152	262	-	-
	SisHSP-26	-	-	-	-	-	-	-	-	-	-	-	-	-	-	-	-	-	-	-	-	-	-	-	-	-	-	82	186	-	-
	SisHSP-27	-	-	-	-	-	-	-	-	-	-	-	-	-	-	-	-	-	-	-	-	-	-	-	-	-	-	88	193	85	178
	SisHSP-28	-	-	-	-	-	-	-	-	-	-	-	-	-	-	-	-	-	-	-	-	-	-	-	-	-	-	1	79	-	-
		-	-	-	-	-	-	-	-	-	-	-	-	-	-	-	-	-	-	-	-	-	-	-	-	-	-	288	350	-	-
	SisHSP-29	-	-	-	-	-	-	-	-	-	-	-	-	-	-	-	-	-	-	-	-	-	-	-	-	-	-	1	78	-	-
	SisHSP-30	-	-	-	-	-	-	-	-	-	-	-	-	-	-	-	-	-	-	-	-	-	-	-	-	-	-	30	123	-	-
	SisHSP-31	-	-	-	-	-	-	-	-	-	-	-	-	-	-	-	-	-	-	-	-	-	-	-	-	-	-	62	145	-	-
	SisHSP-32	-	-	-	-	-	-	-	-	-	-	-	-	-	-	-	-	-	-	-	-	-	-	-	-	-	-	16	107	-	-
	SisHSP-33	-	-	-	-	-	-	-	-	-	-	-	-	-	-	-	-	-	-	-	-	-	-	-	-	-	-	86	181	-	-
	SisHSP-34	-	-	-	-	-	-	-	-	-	-	-	-	-	-	-	-	-	-	-	-	-	-	-	-	-	-	87	190	-	-
	SisHSP-35	-	-	-	-	-	-	-	-	-	-	-	-	-	-	-	-	-	-	-	-	-	-	-	-	-	-	52	155	-	-
	SisHSP-36	-	-	-	-	-	-	-	-	-	-	-	-	-	-	-	-	-	-	-	-	-	-	-	-	-	-	52	155	-	-
	SisHSP-37	-	-	-	-	-	-	-	-	-	-	-	-	-	-	-	-	-	-	-	-	-	-	-	-	-	-	136	241	-	-

- Not present

Supplementary Table 4. Details of *cis*-regulatory elements present in *SiHSP* genes

Supplementary Table 4. Details of *Cis*-regulatory elements present in the promoter region of *SiHSP* genes

[illegible]	[illegible]	[illegible]	[illegible]	[illegible]	[illegible]	[illegible]	[illegible]	[illegible]	[illegible]	[illegible]	[illegible]	[illegible]	[illegible]	[illegible]	[illegible]	[illegible]	[illegible]	[illegible]	[illegible]	[illegible]
-10PEHVPSBD	-	161 (+), 612 (-),	433 (+),	194 (-),	1213 (+), 1342 (-), 1863 (-),	947 (+), 1282 (+),	-	36 (+), 158 (+),	659 (+),	729 (-),	-	1781 (-),	418 (+),	1358 (+),	1452 (+),	893 (-),	-	-	17 (+),	45 (+), 97 (+), 382 (+), [illegible]
2SSEEDPROTBANAPA	222 (+), 869 (-),	959 (+), 1222 (+), 628 (-),	-	567 (+),	-	1620 (+),	598 (+), 1734 (+), 153 (-),	1732 (+),	84 (+),	-	-	-	430 (-), 750 (-),	-	360 (-),	-	-	1634 (+),	1137 (-),	-
[illegible]	[illegible]	[illegible]	[illegible]	[illegible]	[illegible]	[illegible]	[illegible]	[illegible]	[illegible]	[illegible]	[illegible]	[illegible]	[illegible]	[illegible]	[illegible]	[illegible]	[illegible]	[illegible]	[illegible]	[illegible]
-300ELEMENT	257 (-),	187 (+), 441 (-),	398 (-), 1781 (-), 389 (-), 588 (-), 1415 (-),	-	993 (+),	765 (+), 174 (-), 1542 (-),	-	1214 (+),	-	469 (+), 827 (+), 1013 (+), 684 (-),	-	925 (+), 1013 (+), 1910 (-), 475 (-),	213 (+), 504 (+), 1241 (+), 1493 (+),	29 (-),	1682 (+), 1814 (-),	1071 (+), 1902 (-),	541 (+), 1433 (+),	-	1805 (+),	215 (+), 651 (+), 746 (-), [illegible]
[illegible]	[illegible]	[illegible]	[illegible]	[illegible]	[illegible]	[illegible]	[illegible]	[illegible]	[illegible]	[illegible]	[illegible]	[illegible]	[illegible]	[illegible]	[illegible]	[illegible]	[illegible]	[illegible]	[illegible]	[illegible]
AACACOREOSGLUB1	-	1220 (+),	118 (+),	-	-	1357 (+),	-	298 (+), 1254 [illegible]	-	-	217 (-), 954 (-) [illegible]	-	-	1220 (+),	1283 (-),	1268 (+),	-	-	588 (+),	1631 (+), 523 [illegible]
ABREATCONSENSUS	-	-	-	-	-	-	805 (-),	-	-	-	-	-	1726 (-),	-	-	-	-	-	-	-
ABRELATERD1	955 (-),	1604 (+), 1531 (-),	988 (+), 4 (-), 10 (-), 143 (-), 174 (-), 328 (-),	746 (+), 1295 (+), 745 (-), 962 (-), 1204 (-), 1386 (-),	1537 (+),	-	808 (+), 126 (-), 161 (-), 807 (-), 1827 (-), 1844 (-),	-	361 (+), 815 (+), 1575 (+), 360 (-), 554 (-), 1138 (-),	1656 (+), 1655 (-),	1708 (+), 1768 (+), 337 (-), 1767 (-), 1812 (-),	-	1728 (-),	371 (+), 411 (+), 261 (-), 657 (-),	232 (+), 943 (+), 231 (-), 942 (-), 1343 (-),	627 (-),	436 (-),	174 (+), 1857 (+), 65 (-),	298 (+), 485 (+), 732 (+), 925 (+), 1535 (+), 1961 (+), 731 (-),	1019 (+), 1181 (-),
ABREOSRAB21	-	-	988 (+),	1387 (+), 1383 (-),	-	-	804 (-),	-	811 (-),	-	1768 (+),	-	72 (-),	-	1344 (+), 939 (-),	-	-	-	298 (+),	-
ABRERATCAL	521 (+),	1530 (-),	636 (+),	1294 (+), 745 (+), 1845 (+), 744 (-), 1789 (-),	-	-	807 (+), 806 (-), 1826 (-),	-	1574 (+), 360 (+), 359 (-), 498 (-),	1655 (+),	1767 (+), 1766 (-),	-	147 (+), 133 (-), 1451 (-),	1737 (-),	231 (+), 942 (+), 230 (-), 941 (-),	1529 (+),	-	-	297 (+), 924 (+), 731 (+),	1795 (+),
ABREZMRAB28	-	-	-	-	-	-	-	-	-	-	-	-	-	-	-	-	-	-	-	-
ACGTABOX	-	-	-	-	-	-	-	-	-	-	-	-	-	-	-	-	-	27 (+), 27 (-),	1516 (+), 1516 (-),	-

[illegible]

CURECORECR	242 (+), 375 (+), 1246 (+), 1598 (+), 242 (-), 375 (-), 1246 (-), 1598 (-),	147 (+), 311 (+), 1096 (+), 1262 (+), 1340 (+), 1602 (+), 1627 (+), 147 (-), 311 (-), 1096 (-), 1262 (-), 1340 (-), 1602 (-), 1627 [illegible]	34 (+), 168 (+), 386 (+), 515 (+), 1102 (+), 1433 (+), 34 (-), 168 (-), 386 (-), 515 (-), 1102 (-), 1433 (-),	100 (+), 109 (+), 611 (+), 678 (+), 819 (+), 965 (+), 1207 (+), 1236 (+), 100 (-), 109 (-), 611 (-), 678 (-), 819 (-), 965 (-), 1207 (-),	[illegible]
[illegible]					[illegible]
[illegible]					[illegible]
DOFCOREZM	213 (+), 231 (+), 1172 (+), 1308 (+), 1369 (+), 1390 (+), 68 (-), 94 (-), 107 (-), 258 (-), 267 (-), 590 (-), 599 (-), 1024 (-), 1210 (-), 1298 (-), 1381 (-), 1452 (-), 1818 (-), 1824 (-),	31 (+), 114 (+), 133 (+), 172 (+), 269 (+), 293 (+), 301 (+), 357 (+), 516 (+), 622 (+), 939 (+), 1059 (+), 1140 (+), 1144 (+), 1149 (+), 1157 (+), 1398 (+), 1516 (+), 1551 (+), 1827 (+), 250 (-), 255 (-), 263 (-), 650 (-), 1202 (-), 1254 (-), 1691 (-), 1906 (-),	319 (+), 374 (+), 545 (+), 718 (+), 731 (+), 905 (+), 961 (+), 1427 (+), 1490 (+), 1496 (+), 1519 (+), 1528 (+), 1532 (+), 1552 (+), 1605 (+), 1654 (+), 1685 (+), 1705 (+), 1732 (+), 1747 (+), 1795 (+), 1839 (+), 1870 (+), 218 (-), 314 (-),	202 (+), 211 (+), 550 (+), 844 (+), 869 (+), 885 (+), 1006 (+), 1308 (+), 1315 (+), 1501 (+), 1822 (+), 444 (-), 649 (-), 854 (-), 929 (-),	[illegible]
DPBFCOREDCDC3	1275 (+), 336 (-), 378 (-), 640 (-), 1116 (-),	329 (+), 638 (+), 1862 (+),	1660 (+), 1217 (-),	744 (+), 788 (+), 1082 (+), 1645 (+), 1410 (-), 1532 (-), 1865 (-),	[illegible]
DRE1COREZMRAB17	-	-	-	-	[illegible]
DRE2COREZMRAB17	757 (-),	-	-	-	[illegible]
DRECRTCOREAT	1682 (+), 757 (-), 1293 (-),	-	-	1417 (+), 1783 (+), 1703 (-),	[illegible]
[illegible]					[illegible]
E2F1OSPCNA	-	-	-	-	[illegible]
[illegible]					[illegible]
[illegible]					[illegible]
[illegible]					[illegible]
E2FCONSENSUS	1303 (-),	-	307 (+), 726 (-), 1026 (-),	1496 (-),	[illegible]
EBOXBNNAPA	640 (+), 1462 (+), 1515 (+), 1924 (+), 640 (-), 1462 (-), 1515 (-), 1924 (-),	65 (+), 314 (+), 377 (+), 639 (+), 656 (+), 727 (+), 772 (+), 804 (+), 1036 (+), 1391 (+), 1474 (+), 65 (-), 314 (-), 377 (-), 639 (-), 656 (-), 727 (-), 772 (-), 804 (-), 1036 (-), 1391 (-), 1474 (-),	135 (+), 1564 (+), 1670 (+), 1720 (+), 1990 (+), 135 (-), 1564 (-), 1670 (-), 1720 (-), 1990 (-),	7 (+), 179 (+), 345 (+), 745 (+), 1083 (+), 1178 (+), 1410 (+), 7 (-), 179 (-), 345 (-), 745 (-), 1083 (-), [illegible] (-), 1410 (-),	[illegible]
EECCRCAH1	1372 (+), 1456 (+), 307 (-), 1467 (-), 1634 (-),	465 (-), 1463 (-),	823 (+), 1017 (+), 1326 (+), 222 (-), 1016 (-),	120 (+), 1283 (+), 1500 (-),	[illegible]
ELRECOREPCRP1	435 (+),	1379 (-),	648 (-),	1730 (+),	[illegible]
[illegible]					[illegible]
EMBP1TAEM	-	-	-	-	[illegible]
[illegible]					[illegible]
ERELEE4	1453 (-),	-	-	-	[illegible]
EVENINGAT	-	-	-	-	[illegible]
GADOWNAT	-	1604 (+),	-	-	[illegible]
GAGA8HVBKN3	-	-	-	-	[illegible]
GAGAGMGSA1	-	-	-	-	[illegible]
GARE1OSREP1	-	164 (-),	-	-	[illegible]
GARE2OSREP1	-	-	-	-	[illegible]
GAREAT	1386 (+),	21 (+), 109 (+), 351 (+),	-	-	[illegible]
GATABOX	37 (+), 203 (+), 418 (+), 904 (+), 944 (+), 1270 (+), 1535 (+), 31 (-), 391 (-), 910 (-), 1422 (-), 1512 (-), 1537 (-),	141 (+), 536 (+), 1088 (+), 1832 (+), 1928 (+), 75 (-), 538 (-), 955 (-), 1018 (-), 1484 (-), 1688 (-), 1694 (-),	73 (+), 684 (+), 1237 (+), 1744 (+), 231 (-), 293 (-), 325 (-), 565 (-), 803 (-), 1318 (-), 1774 (-),	257 (+), 400 (+), 438 (+), 894 (+), 909 (+), 1011 (+), 563 (-), 658 (-), 759 (-), 1056 (-), [illegible] (-),	[illegible]
GBOXLERBCS	-	-	-	-	[illegible]
[illegible]					[illegible]
GCCCORE	1148 (+), 1884 (+), 1951 (+),	1814 (+),	642 (+), 722 (+), 1898 (-),	1626 (+),	[illegible]
[illegible]					[illegible]
[illegible]					[illegible]
GLMHVCHORD	-	-	-	-	[illegible]
[illegible]					[illegible]
[illegible]					[illegible]
GT1CONSENSUS	1547 (+), 74 (-), 188 (-), 998 (-), 999 (-), 1819 (-),	39 (+), 341 (+), 555 (+), 566 (+), 1012 (+), 1013 (+), 1268 (+), 1519 (+), 43 (-), 1386 (-), 1543 (-), 1931 (-), 56 (-), 98 (-), 258 (-), 442 (-), 1692 (-),	18 (+), 19 (+), 58 (+), 59 (+), 453 (+), 715 (+), 1602 (+), 1744 (+), 1193 (-), 1316 (-), 84 (-), 85 (-), 211 (-), 305 (-), 306 (-), 390 (-), 399 (-),	400 (+), 1311 (+), 561 (-), 1285 (-),	[illegible]
GT1CORE	349 (+),	195 (+),	-	1155 (+),	[illegible]
GT1GMSCAM4	998 (-), 1819 (-),	341 (+), 56 (-), 258 (-), 442 (-),	19 (+), 59 (+), 84 (-), 211 (-), 305 (-), 390 (-), 399 (-),	1311 (+),	[illegible]
[illegible]					[illegible]
GTGANTG10	35 (+), 477 (+), 495 (+), 706 (+), 797 (+), 892 (+), 1134 (+), 1313 (+), 1580 (+), 176 (-), 262 (-), 291 (-), 429 (-), 469 (-), 520 (-), 634 (-), 656 (-), 698 (-), 710 (-), 951 (-), 1048 (-), 1084 (-), 1435 (-), 1514 (-),	928 (+), 1041 (+), 1525 (+), 276 (-), 662 (-), 1349 (-), 1390 (-), 1444 (-), 1486 (-),	52 (+), 194 (+), 338 (+), 670 (+), 957 (+), 1010 (+), 1060 (+), 1144 (+), 1185 (+), 1938 (+), 3 (-), 9 (-), 103 (-), 109 (-), 142 (-), 173 (-), 203 (-), 233 (-), 238 (-), 295 (-), 327 (-), 462 (-), 572 (-), 1501 (-),	145 (+), 153 (+), 406 (+), 944 (+), [illegible] (+), 1357 (+), 280 (-), 772 (-), 1067 (-), 1169 (-), [illegible] (-), 1490 (-), 1635 (-), [illegible] (-),	[illegible]
HBOXCONSENSUSPVC[illegible]	-	-	-	-	[illegible]
[illegible]					[illegible]
[illegible]					[illegible]
HEXAMERATH4	-	-	677 (-), 1139 (-),	19 (+), 1450 (-), 1665 (-),	[illegible]
[illegible]					[illegible]
HEXMOTIFTAH3H4	-	-	1353 (+),	-	[illegible]
HSE	-	-	-	-	[illegible]
HSELIKENTACIDICPR1	-	-	-	-	[illegible]
[illegible]					[illegible]
IBOX	-	-	-	-	[illegible]
IBOXCORE	30 (-),	1693 (-),	1744 (+), 292 (-), 1317 (-),	400 (+), 562 (-),	[illegible]
IBOXCORENT	-	-	-	-	[illegible]
INRNTPSADB	-	1116 (-),	1088 (+), 1107 (+), 22 (-), 1843 (-), 1003 (-),	766 (+), 1097 (-),	[illegible]
INTRONLOWER	-	602 (-),	354 (+),	229 (-),	[illegible]
[illegible]					[illegible]
IRO2OS	-	-	-	745 (+),	[illegible]
L1BOXATPDF1	-	-	-	-	[illegible]
LEAFYATAG	-	923 (+), 664 (-	-	-	[illegible]
LECPLEACS2	-	-	-	-	[illegible]
LREBOXIIPCCHS1	-	-	-	-	[illegible]
[illegible]					[illegible]
LRENPCABE	-	-	-	-	[illegible]
LTRE1HVBLT49	1714 (-),	-	-	-	[illegible]
LTREATLTI78	-	-	-	-	[illegible]
LTRECOREATCOR15	1218 (+), 1683 (+), 757 (-), 1293 (-),	1405 (-),	676 (+), 1760 (-),	534 (+), 1418 (+), 1449 (+), 1664 (+), 1784 (+), 1695 (-), [illegible]	[illegible]
MARABOX1	-	-	-	-	[illegible]
MARARS	-	-	-	-	[illegible]
MARTBOX	1617 (-),	-	-	-	[illegible]
[illegible]					[illegible]
[illegible]					[illegible]
[illegible]					[illegible]
MYB1AT	397 (+), 27 (-), 48 (-), 348 (-),	1133 (+), 1271 (+), 800 (+), 52 (-), 194 (-),	1542 (+), 1547 (+),	1154 (-), [illegible] (-),	[illegible]
MYB1LEPR	-	1667 (-),	580 (-),	-	[illegible]
[illegible]					[illegible]
MYB2AT	21 (+),	320 (+),	-	-	[illegible]
MYB2CONSENSUSAT	811 (+), 21 (+), 1462 (-),	1474 (+), 320 (+), 377 (-),	-	1464 (+), 729 (-), 1252 (-),	[illegible]
MYBATRD22	-	-	-	-	[illegible]
MYBCORE	734 (+), 1054 (+), 1462 (+), 1717 (+), 811 (-), 21 (-), 1895 (-),	165 (+), 377 (+), 491 (-), 1305 (-), 1474 (-), 320 (-),	250 (-), 344 (-), 1982 (-),	729 (+), 1230 (+), 1252 (+), 1464 (-), [illegible] (-), 1158 (-),	[illegible]
MYBCOREATCYCB1	812 (+), 1551 (+), 1901 (+), 1665 (-),	411 (+),	1171 (+),	1465 (+), 1584 (+), 1799 (+), [illegible] (-), 250 (-), 729 (-), 1247 (-), 1252 (-), [illegible]	[illegible]
MYBGAHV	1386 (+),	21 (+), 109 (+), 351 (+),	-	-	[illegible]
MYBPLANT	398 (+),	-	1543 (+),	777 (-), 1151 (-),	[illegible]

MYBPZM	1774 (+), 1978 (+), 1055 (-), 1100 (-),	1368 (+), 1576 (+), 1630 (+), 1775 (+),	1571 (+), 1204 (-), 1878 (-),	1151 (-), 1257 (-),	1720 (+), 475 (-), 1294 (-), 1694 (-),	-	1062 (+), 1399 (+),	-	889 (-),	949 (+),	[illegible]
MYBST1	417 (+), 903 (+), 943 (+), 1422 (-), 1537 (-),	140 (+), 1831 (+), 538 (-), 955 (-), 1018 ( ),	803 (-),	256 (+), 437 (+), 893 (+), 1010 (+), 563 (-),	444 (+), 522 (+), 835 (+), 1150 (+), 542 (-), 1090 (-), 1798 (-),	5 (+),	1699 (-),	240 (+),	165 (+), 548 (+), 1961 (+), 13 (-),	134 (+), 488 (-),	[illegible]
MYCATERD1	-	65 (+), 639 (-), 772 (-),	135 (-),	1410 (+),	1249 (-),	1527 (-),	667 (+),	-	-	892 (-), 1883 ( ),	[illegible]
MYCATRD22	-	639 (+), 772 (+), 65 (-),	135 (+),	1410 (-),	1249 (+),	1527 (+),	667 (-),	-	-	892 (+), 1883 (+),	[illegible]
MYCCONSENSUSAT	640 (+), 1462 (+), 1515 (+), 1924 (+), 640 (-), 1462 (-), 1515 (-), 1924 (-),	65 (+), 314 (+), 377 (+), 639 (+), 656 (+), 727 (+), 772 (+), 804 (+), 1036 (+), 1391 (+), 1474 (+), 65 (-), 314 (-), 377 (-), 639 (-), 656 (-), 727 (-), 772 (-), 804 (-), 1036 (-), 1391 (-), 1474 (-),	135 (+), 1564 (+), 1670 (+), 1720 (+), 1990 (+), 135 (-), 1564 (-), 1670 (-), 1720 (-), 1990 (-),	7 (+), 179 (+), 345 (+), 745 (+), 1083 (+), 1178 (+), 1410 (+), 7 (-), 179 (-), 345 (-), 745 (-), 1083 (-), 1178 (-), 1410 (-),	273 (+), 746 (+), 874 (+), 934 (+), 1189 (+), 1249 (+), 1320 (+), 1334 (+), 1531 (+), 273 (-), 746 (-), 874 (-), 934 (-), 1189 (-), 1249 (-), 1320 (-), 1334 (-), 1531 (-),	150 (+), 326 (+), 415 (+), 636 (+), 1338 (+), 1527 (+), 1626 (+), 1704 (+), 1729 (+), 1793 (+), 1945 (+), 150 (-), 326 (-), 415 (-), 636 (-), 1338 (-), 1527 (-), 1626 (-), 1704 (-), 1729 (-), 1793 (-), 1945 (-),	194 (+), 667 (+), 807 (+), 1046 (+), 1603 (+), 194 (-), 667 (-), 807 (-), 1046 (-), 1603 (-),	167 (+), 177 (+), 460 (+), 471 (+), 496 (+), 734 (+), 887 (+), 901 (+), 908 (+), 1022 (+), 1359 (+), 1584 (+), 1721 (+), 167 (-), 177 (-), 460 (-), 471 (-), 496 (-), 734 (-), 887 (-), 901 (-), 908 (-), 1022 (-), 1359 (-), 1584 (-), 1721 (-),	326 (+), 339 (+), 360 (+), 416 (+), 425 (+), 634 (+), 1332 (+), 1540 (+), 1591 (+), 1621 (+), 1740 (+), 326 (-), 339 (-), 360 (-), 416 (-), 425 (-), 634 (-), 1332 (-), 1540 (-), 1591 (-), 1621 (-), 1740 (-),	518 (+), 528 (+), 552 (+), 735 (+), 892 (+), 911 (+), 1418 (+), 1596 (+), 1655 (+), 1883 (+), 518 (-), 528 (-), 552 (-), 735 (-), 892 (-), 911 (-), 1418 (-), 1596 (-), 1655 (-), 1883 (-),	[illegible]
NAPINMOTIFBN	-	-	-	-	1247 (+),	-	1379 (-),	1235 (-),	-	-	[illegible]
NODCON1GM	1369 (+), 588 (-), 1379 (-),	1689 (-),	374 (+), 961 (+), 1427 (+), 1496 (+), 1685 (+),	-	1438 (+), 1656 (+),	447 (+), 1738 (+), 306 (-), 889 (-), 1157 ( ), 1880 (-),	-	1218 (+),	210 (-), 253 (-),	682 (-),	[illegible]
NODCON2GM	513 (+), 726 (+), 1022 (+), 1309 (-),	1571 (+), 270 (-), 517 (-), 526 (-), 1418 ( ),	216 (+), 1289 (+), 449 (-), 1606 (-), 1706 (-), 1733 (-),	647 (+), 188 (-), 870 (-),	153 (+), 166 (+), 800 (+), 1454 (+), 1588 (+), 850 (-), 911 (-), 1226 (-),	67 (+), 725 (+), 606 (-), 779 (-), 1988 ( ),	1959 (+), 1352 (-), 1362 (-),	554 (+), 1081 (+), 1325 (+), 1969 (+), 319 (-), 560 (-), 601 (-), 614 (-), 882 (-), [illegible]	479 (+), 1199 (+), 1221 (+), 405 (-), 1972 ( ),	310 (+), 788 (+), 43 (-), 237 (-), 503 (-),	[illegible]
[illegible]											[illegible]
NONAMERMOTIFTAH3	-	-	-	-	-	-	-	-	-	-	[illegible]
NRRBNEXTA	-	-	-	-	-	-	-	-	-	-	[illegible]
NTBBF1ARROLB	-	649 (+), 300 (-), 938 (-),	1781 (+),	443 (+), 549 (-),	-	-	577 (-),	-	-	-	[illegible]
[illegible]											[illegible]
OCTAMERMOTIFTAH3	-	-	-	-	-	-	-	-	-	-	[illegible]
OCTAMOTIF2	-	-	-	-	-	-	-	-	-	1107 (+),	[illegible]
[illegible]											[illegible]
[illegible]											[illegible]
OSE1ROOTNODULE	1369 (+), 588 (-), 1379 (-),	1689 (-),	374 (+), 961 (+), 1427 (+), 1496 (+), 1685 (+),	-	1438 (+), 1656 (+),	447 (+), 1738 (+), 306 (-), 889 (-), 1157 ( ), 1880 (-),	-	1218 (+),	210 (-), 253 (-),	682 (-),	[illegible]
OSE2ROOTNODULE	513 (+), 726 (+), 1022 (+), 1309 (-),	1571 (+), 270 (-), 517 (-), 526 (-), 1418 ( ),	216 (+), 1289 (+), 449 (-), 1606 (-), 1706 (-), 1733 (-),	647 (+), 188 (-), 870 (-),	153 (+), 166 (+), 800 (+), 1454 (+), 1588 (+), 850 (-), 911 (-), 1226 (-),	67 (+), 725 (+), 606 (-), 779 (-), 1988 ( ),	1959 (+), 1352 (-), 1362 (-),	554 (+), 1081 (+), 1325 (+), 1969 (+), 319 (-), 560 (-), 601 (-), 614 (-), 882 (-), [illegible]	479 (+), 1199 (+), 1221 (+), 405 (-), 1972 ( ),	310 (+), 788 (+), 43 (-), 237 (-), 503 (-),	[illegible]
P1BS	-	-	-	-	-	944 (+), 944 (-),	1716 (+), 1716 (-),	1183 (+), 1183 (-),	1590 (+), 1590 (-),	551 (+), 551 (-),	[illegible]
PALBOXAPC	1731 (-),	-	-	1219 (+), 1525 (+), 1698 (-),	-	-	1305 (+), 418 (-),	-	862 (+), 1231 (+),	-	[illegible]
[illegible]											[illegible]
[illegible]											[illegible]
PALINDROMICCBOXG	-	-	-	-	-	-	-	-	-	-	[illegible]
[illegible]											[illegible]
PIATGAPB	706 (+), 706 (-	-	-	-	-	-	-	-	-	-	[illegible]
POLASIG1	1169 (+),	298 (+), 390 (+), 614 (+), 1427 (+),	407 (+), 413 (+), 423 (+), 431 (-),	199 (+),	422 (-), 813 (-), 972 (-), 1128 (-), 1310 (-), 1470 (-), 1844 (-),	770 (+),	1880 (+),	1653 (+), 1415 (-),	-	474 (+),	[illegible]
POLASIG2	-	1003 (-),	64 (+),	-	807 (-),	599 (+), 1113 (+), 75 (-),	683 (-),	1647 (+),	1989 (-),	-	[illegible]
POLASIG3	-	1708 (+),	188 (+), 456 (+), 378 (-),	196 (+), 557 (+),	-	48 (+), 1189 (+), 859 (-),	-	-	716 (+),	928 (-),	[illegible]
POLLEN1LELAT52	615 (+), 991 (+), 1367 (+), 269 (-), 596 (-), 1412 (-), 1821 (-),	340 (+), 359 (+), 1061 (+), 1138 (+), 1142 (+), 1146 (+), 1155 (+), 1249 (+), 1455 (+), [illegible]	372 (+), 1152 (+), 1530 (+), 1652 (+), 1699 (+), 1730 (+), 1797 (+), 1841 (+), 213 (-), 534 (-),	209 (+), 237 (+), 1310 (+), 1796 (+),	156 (-), 163 (-), 614 (-), 792 (-), 899 (-), 1457 (-), 1647 (-), 1840 (-),	390 (+), 520 (+), 609 (+), 1354 (+), 1487 (+), 1575 (+), 1613 (+), 1646 (+), 138 (-), 240 (-), [illegible]	17 (+), 43 (+), 1355 (+), 1962 (-), 1981 (-),	229 (+), 1092 (+), 1225 (+), 1231 (+), 1298 (+), 1643 (+), 1859 (+), 1867 (+), 1881 (+), [illegible]	54 (-), 606 (-), 1128 (-),	46 (+), 234 (+), 645 (+), 1682 (+), 68 (-), 92 (-), 125 (-), 153 (-), 653 (-), 817 (-),	[illegible]
[illegible]											[illegible]
PREATPRODH	-	1466 (+), 1796 (+),	1006 (-),	-	-	829 (+), 1821 (+),	1516 (+), 1677 (+),	1444 (+), 632 (-),	-	812 (+), 1708 (+), 400 (-),	[illegible]
PRECONSCRHSP70A	1218 (+), 1683 (+),	1386 (-),	1190 (+), 1313 (+),	1449 (+), 1652 (+), 1676 (-), 1636 (-), 1684 (-), 818 (-),	1267 (+), 535 (-),	525 (-),	1216 (+), 1797 (-),	1029 (-), 1785 (-),	1085 (+), 1395 (+), 1441 (+), 1569 (+), 761 (-),	1546 (+), 1915 (-), 1605 (-),	[illegible]
PROLAMINBOXOSGLU	-	-	589 (-), 1416 (	-	-	-	-	857 (+),	-	827 (+), 1013	[illegible]
PROXBBNNAPA	868 (-),	959 (+), 1222	-	567 (+),	-	-	598 (+), 152 (-	-	-	-	[illegible]
PYRIMIDINEBOXHVEP	997 (+),	-	304 (+), 18 (-),	-	-	-	-	1771 (-),	-	-	[illegible]
PYRIMIDINEBOXOSRAMY1A	1817 (+), 212 (-),	113 (-),	531 (+), 717 (-),	853 (+), 884 (-), 1314 (-),	996 (-),	131 (+),	-	1666 (+), 360 (-), 1901 (-),	586 (+),	65 (+), 89 (+), 102 (+),	[illegible]
QARBNEXTA	-	-	-	1294 (+),	-	-	-	-	-	-	[illegible]
QELEMENTZMZM13	-	-	-	1804 (+), 1485 (-),	1700 (+),	-	-	-	1056 (+), 1615 (-),	506 (+),	[illegible]
RAV1AAT	790 (-),	491 (+), 1219 (+), 1305 (+), 1373 (+), 1556 (+), 1559 (+),	124 (+), 1511 (+), 1982 (+), 1221 (-),	1516 (+), 932 (-), 935 (-),	549 (+), 704 (+), 109 (-), 345 (-),	622 (+), 1753 (+), 1758 (+), 569 (-), 1137 ( ), 1450 (-),	943 (+), 1933 (+),	186 (+), 431 (+), 716 (+), 1150 (+), 1697 (+), 844 (-), 1977 (-),	-	377 (+), 380 (+), 515 (+), 1198 (+), 1719 (+), 179 (-), 671 (-), 903 (-), 906 (-),	[illegible]
RAV1BAT	1515 (+), 640 (-),	656 (+),	1990 (-),	-	273 (-), 934 (-),	1729 (+),	-	460 (-),	1332 (+), 1621 (+),	1596 (+), 528 (-),	[illegible]
[illegible]											[illegible]
RBCSCONSENSUS	-	-	-	-	1717 (+),	-	31 (+),	623 (-), 661 (-),	-	542 (-),	[illegible]
REALPHALGLHCB21	398 (+),	1302 (+), 1370 (+),	1543 (+), 1548 (+),	1153 (-), 1255 (-),	-	71 (-),	228 (-),	366 (+),	-	708 (+), 951 (+),	[illegible]
REBETALGLHCB21	902 (+),	1018 (-),	-	892 (+),	-	-	-	-	1960 (+),	-	[illegible]
[illegible]											[illegible]
RHERPATEXPA7	33 (-), 1122 (-), 1578 (-),	233 (-),	3 (+), 9 (+), 142 (+), 173 (+), 327 (+), 988 (-),	961 (+), 1001 (+), 1203 (+), 1067 (+), 1169 (+), 803 (-),	-	-	160 (+), 808 (-),	1817 (+),	361 (+), 815 (-), 1575 (-),	1724 (+), 1654 (+), 1656 (-),	[illegible]
ROOTMOTIFTAPOX1	72 (+), 389 (-),	91 (+), 142 (+), 160 (+), 287 (+), 1731 (+), 1929 (+), 90 (-), 159 (-), 199 (-), 394 (-), 698 (-), 1016 (-), 1730 (-),	396 (+), 428 (+), 427 (-),	-	1041 (+), 1074 (+), 1353 (+), 1413 (+), 1791 (+), 1142 (-), 1485 (-), 1790 (-), 1865 (-),	117 (+), 206 (+), 716 (+), 748 (+), 946 (+), 961 (+), 1179 (+), 643 (-), 945 (-), 953 (-), 960 (-), 1178 (-),	528 (+), 1471 (+), 1718 (+), 555 (-), 653 (-), 1470 (-), 1717 (-),	35 (+), 157 (+), 242 (+), 1185 (+), 156 (-), 1631 (-), 1797 (-),	134 (+), 167 (+), 240 (+), 304 (+), 658 (+), 931 (+), 118 (-), 930 (-), 1982 (-),	161 (+), 898 (+), 160 (-), 957 (-),	[illegible]
RYREPEATBNNAPA	1557 (+), 1343 (-),	1490 (+),	352 (+), 1455 (+), 256 (-), 350 (-), 1457 ( ), 1724 (-),	139 (+), 693 (+), 137 (-), 695 (-),	491 (+), 489 (-),	144 (+), 1086 (+),	52 (-),	1811 (-),	925 (+),	1011 (+), 1071 (+),	[illegible]
RYREPEATGMGY2	-	-	1455 (+), 1456 (-), 1723	693 (+), 694 (-),	-	1086 (+),	-	-	-	-	[illegible]
RYREPEATLEGUMINBOX	-	-	1455 (+), 1456 (-), 1723 (-), 255 (-),	693 (+), 694 (-),	488 (-),	144 (+), 1086 (+),	-	1810 (-),	-	-	[illegible]
RYREPEATVFLEB4	-	-	1455 (+), 1455 (-),	693 (+), 693 (-),	-	-	-	-	-	-	[illegible]
S1FBOXSORPS1L21	1545 (+),	1266 (+), 1632 (-),	-	-	-	100 (+), 1813 (-),	-	787 (-),	121 (+),	326 (+), 484 (+),	[illegible]
S1FSORPL21	-	-	-	-	-	-	-	-	-	326 (+),	[illegible]
SBOXATRBCS	-	-	-	-	1201 (+), 938	-	1149 (+),	-	-	-	[illegible]
SEBFCONSSTPR10A	631 (+), 1081 (+),	659 (+),	1910 (+), 1608 (-),	277 (+),	930 (-),	1461 (-),	185 (+), 590 (+), 273 (-), 565 (-), 1545 (	694 (+),	1794 (+),	531 (-),	[illegible]
SEF1MOTIF	-	390 (-), 694 (-	428 (+), 423 (-	-	1791 (+),	-	-	1627 (-),	-	-	[illegible]
SEF3MOTIFGM	218 (+), 1625 (+),	1671 (+),	1380 (-),	1979 (+),	1774 (-),	1657 (+),	-	1344 (-),	1005 (+),	-	[illegible]
SEF4MOTIFGM7S	882 (+), 250 (-),	1916 (-),	409 (-),	1942 (+),	325 (+), 469 (+),	833 (+), 210 (-), 772 (-), 1116 (-),	-	1169 (+), 583 (-),	752 (+),	405 (-),	[illegible]
[illegible]											[illegible]
[illegible]											[illegible]
SITEIIATCYTC	1258 (+), 1302 (+), 1691 (+), 1933 (-),	-	1765 (-),	879 (-), 1374 ( ), 1841 (-),	-	-	939 (-), 1224 ( ),	1588 (+),	969 (+), 1544 (+), 1810 (+), 962 (-), 1035 ( ),	195 (+), 1376 (+), 1713 (+), 1749 (+), 1964 (+), 1202 (-), 1715 (-), 1863 (-),	[illegible]
[illegible]											[illegible]
[illegible]											[illegible]
SORLIP1AT	1845 (+), 1887 (+), 1919 (+), 536 (-),	591 (+), 1074 (+), 1357 (+), 579 (-),	1666 (+), 1905 (-),	1384 (+), 1560 (+), 1825 (+), 1108 (-), 1548 (-), 1597 (-), 1761 (-),	483 (-), 666 (-), 870 (-), 1264 (-),	507 (-),	776 (+), 805 (+), 985 (+), 1014 (+), 1130 (+), 436 (-), 876 (-), 909 (-), 1011 (	793 (+), 1582 (+), 904 (-), 942 (-),	1241 (+), 1285 (+), 1869 (+), 502 (-), 894 (-), 950 (-),	198 (+), 843 (+), 1393 (+), 1390 (-),	[illegible]
SORLIP2AT	1259 (+), 1303 (+), 1692 (+), 504 (-), 1265 (-), 1693 (-),	-	1581 (-),	888 (+), 1047 (+), 1512 (+), 980 (-), 1617 ( ), 1669 (-), 1841 (-),	1477 (+),	1277 (-),	72 (+), 939 (-), 1224 (-),	-	1849 (+), 1035 (-), 1850 (-),	196 (+), 1714 (+), 1750 (+), 1878 (+), 1322 (-), 1715 (-), 1751 (-), 1863 (-), 1879 (-),	[illegible]
[illegible]											[illegible]
[illegible]											[illegible]
SORLIP5AT	-	-	1008 (+),	-	1975 (-),	-	-	-	1048 (-),	-	[illegible]
[illegible]											[illegible]
SORLREP3AT	-	-	-	-	-	-	-	-	-	-	[illegible]
SORLREP4AT	-	901 (+), 684 (-	-	302 (-),	-	-	1691 (+), 210	-	-	-	[illegible]
[illegible]											[illegible]
SP8BFIBSP8BIB	-	-	417 (-),	-	-	-	-	1312 (+),	-	-	[illegible]
SPHCOREZMC1	-	-	1456 (-),	-	-	-	-	-	-	-	[illegible]
SREATMSD	-	-	-	562 (+),	-	-	1698 (+),	-	12 (+),	-	[illegible]
[illegible]											[illegible]
[illegible]											[illegible]
SURECOREATSULTR11	460 (+), 1089 (-),	1741 (+), 1892 (+), 1988 (+), 842 (-), 1111 (-),	1608 (+), 1739 (+), 1086 (-), 1912 (-),	364 (+), 454 (-),	-	877 (+), 1150 (+), 1461 (+), 1849 (-),	273 (+), 1194 (+), 1545 (+), 479 (-), 592 (-), 699 (-), 1778 (-),	562 (+), 1243 (+), 696 (-),	-	872 (+), 1315 (+), 1736 (+), 1765 (+), 786 (-),	[illegible]
SV40COREENHAN	1024 (-),	-	812 (-),	-	1170 (+),	891 (-),	844 (+),	1513 (-),	-	-	[illegible]
TAAAGSTKST1	1171 (+), 68 (-),	300 (+), 621 (+), 938 (+), 1058 (+), 1515 (+), 650 (-), 1691 (-),	1746 (+), 1869 (+), 1782 (-),	201 (+), 549 (+), 444 (-),	127 (+), 682 (+), 1437 (+), 1843 (-), 1899 (-),	1779 (+), 308 (-), 1286 (-),	577 (+), 830 (-),	558 (+), 1452 (-),	24 (+),	171 (+), 747 (+), 806 (-),	[illegible]
[illegible]											[illegible]
TATABOX2	-	86 (+),	-	-	353 (-), 1070 ( ), 1409 (-),	-	-	1469 (+),	-	246 (-),	[illegible]
TATABOX3	-	-	-	-	415 (+),	-	-	-	-	-	[illegible]
TATABOX4	-	-	-	-	381 (+),	264 (+), 263 (-),	-	-	-	-	[illegible]
TATABOX5	1892 (-),	389 (-),	406 (-), 412 (-),	-	973 (+), 1129 (+), 1311 (+),	769 (-),	1879 (-),	1416 (+), 1429 (-), 1652 (-),	1904 (-),	473 (-),	[illegible]
TATABOXOSPAL	-	695 (-),	-	-	-	-	-	1628 (-),	-	-	[illegible]
TATAPVTRNALEU	-	-	-	-	-	-	-	-	-	-	[illegible]
TATCCACHVAL21	-	-	-	-	-	-	-	-	-	-	[illegible]
TATCCAOSAMY	1422 (+), 1537 (+),	538 (+), 955 (+),	-	563 (+), 255 (-), 436 (-),	1798 (+),	-	-	239 (-),	13 (+), 164 (-),	488 (+),	[illegible]
TATCCAYMOTIFOSRA	-	-	-	435 (-),	1798 (+),	-	-	238 (-),	13 (+), 163 (-),	-	[illegible]
TBOXATGAPB	1451 (+),	249 (+), 132 (-),	-	-	177 (+),	936 (+), 1035 (+), 1143 (+),	-	533 (-),	451 (-),	19 (-),	[illegible]
[illegible]											[illegible]
[illegible]											[illegible]
[illegible]											[illegible]
TGACGTVMAMY	-	-	1353 (-),	-	1535 (+),	-	1640 (+), 127 (-),	-	1139 (-),	-	[illegible]
T/GBOXATPIN2	-	1531 (-),	4 (-), 143 (-),	1294 (+),	-	-	1827 (-),	-	1574 (+),	-	[illegible]
TGTCACACMCUCUMIS	-	660 (+), 927 (-	101 (+),	278 (+),	-	-	186 (+), 564 (-	-	1795 (+),	-	[illegible]
[illegible]											[illegible]

**TRANSINITDICOTS**	-	582 (-),	-	637 (-),	594 (-),	745 (-), 1008 (	-	-	-	290 (-), 1362 (	-	-	787 (-),	-	-	-	-	414 (-),	1489 (-),
**TRANSINITMONOCOT**	-	582 (-),	97 (-),	637 (-),	594 (-),	745 (-), 1008 (	-	-	1499 (-),	290 (-), 1362 (	-	1322 (-),	441 (-), 787 (-	176 (-),	-	857 (-),	-	414 (-),	1489 (-),
**UP1ATMSD**	1255 (-), 1299	-	-	-	-	-	-	-	-	-	-	-	-	-	-	-	-	-	-
**UP2ATMSD**	843 (-), 1188 (	-	-	-	727 (-),	-	-	-	-	-	-	-	-	-	-	-	-	1346 (-),	942 (+),
**UPRMOTIFIIAT**	-	1807 (+),	1563 (+),	-	-	-	1298 (+), 1843 (+),	-	-	-	-	-	-	-	-	1509 (+),	-	-	1536 (-),
**VOZATVPP**	-	-	-	-	-	-	-	-	-	-	-	-	-	-	-	-	-	-	-
**WBBOXPCWRKY1**	-	1379 (-),	-	181 (+), 1729 (+),	-	-	-	-	-	-	-	-	-	-	363 (+),	1263 (-),	-	-	-
**WBOXATNPR1**	435 (+), 683 (+), 717 (-), 751 (-), 928 (-),	780 (+), 1380 (-), 1782 (-),	1371 (+), 486 (-), 649 (-), 781 (-),	182 (+), 826 (+), 1730 (+), 579 (-),	138 (+), 1702 (-),	418 (+), 1038 (+), 96 (-), 1175 (-),	1579 (+), 1639 (+), 1752 (+), 129 (-),	200 (+), 1126 (-), 1163 (-), 1686 (-), 1695 (-), 1839 (-),	201 (+),	15 (+), 1592 (+), 322 (-),	1185 (+), 193 (-), 859 (-), 1752 (-),	177 (-), 339 (-),	959 (+), 978 (+), 1049 (+),	1047 (+), 1340 (+), 1801 (+), 1964 (+), 844 (-), 1772 (-),	364 (+),	9 (+), 1264 (-),	-	853 (+), 920 (+), 1985 (+),	1592 (-),
**WBOXHVISO1**	496 (+), 684 (+), 12 (-), 174 (-), 716 (-), 1046 (-),	-	1061 (+), 503 (-),	183 (+), 827 (+), 578 (-),	986 (+), 1873 (+), 1706 (-),	1342 (+), 275 (-), 661 (-), 1174 (-),	1460 (-),	48 (+), 1264 (+), 75 (-), 454 (-), 1685 (-),	189 (+), 197 (+), 848 (+), 1321 (-),	1706 (+), 321 (-), 1649 (-),	1134 (+), 654 (-), 685 (-), 858 (-), 871 (-),	1498 (-),	810 (+), 1050 (+), 1739 (-), 1845 (-),	775 (-), 792 (-),	365 (+),	350 (+), 1019 (+), 1076 (-),	-	1986 (+),	13 (+), 992 (+), 1197 (+), 1498 (-), 1591 (-),
**WBOXNTCHN48**	12 (-),	-	1258 (+), 1616 (+),	593 (+), 1484 (+),	-	1341 (+), 661 (-),	1137 (+),	47 (+), 75 (-), 522 (-),	847 (+),	-	484 (+), 1133 (+), 654 (-), 685 (-),	-	1739 (-),	-	1038 (+),	349 (+), 1076 (-),	-	-	1196 (+), 1498 (-),
**WBOXNTERF3**	436 (+), 496 (+), 684 (+), 12 (-), 174 (-), 716 (-), 1046 (-), 949 (-),	1718 (+), 478 (-), 1206 (-), 1379 (-),	1061 (+), 1145 (+), 1259 (+), 1617 (+), 1939 (+), 503 (-), 648 (-),	183 (+), 594 (+), 827 (+), 1358 (+), 1485 (+), 1731 (+), 578 (-), 1271 (-), 1805 (-)	986 (+), 1611 (+), 1873 (+), 1706 (-), 1701 (-),	1342 (+), 1831 (+), 1958 (+), 275 (-), 661 (-), 1174 (-),	1138 (+), 1808 (+), 1460 (-),	48 (+), 1264 (+), 75 (-), 454 (-), 1685 (-), 522 (-), 1566 (-),	189 (+), 197 (+), 630 (+), 848 (+), 1445 (+), 1615 (+), 1321 (-), 1057 (-),	1706 (+), 321 (-), 1649 (-), 507 (-),	485 (+), 1134 (+), 654 (-), 685 (-), 858 (-), 871 (-),	1498 (-), 338 (-), 662 (-),	810 (+), 960 (+), 979 (+), 1050 (+), 1739 (-), 1845 (-),	1965 (+), 775 (-), 792 (-), 1261 (-),	365 (+), 409 (+), 1039 (+),	10 (+), 350 (+), 1019 (+), 1076 (-), 1263 (-),	400 (-), 920 (-), 1405 (-), 1510 (-),	110 (+), 121 (+), 1986 (+), 1734 (-),	13 (+), 992 (+), 1197 (+), 1498 (-), 1591 (-),
**WRKY71OS**	247 (+), 436 (+), 496 (+), 684 (+), 13 (-), 175 (-), 341 (-), 383 (-), 633 (-), 717 (-), 739 (-), 751 (-), 928 (-), 950 (-), 1047 (-), 1083 (-),	781 (+), 929 (+), 1322 (+), 1433 (+), 1659 (+), 1718 (+), 479 (-), 661 (-), 1207 (-), 1380 (-), 1782 (-), 1789 (-), 1789 (-), 1789 (-), 1789 (-), 1789 (-), 1789 (-),	1061 (+), 1136 (+), 1145 (+), 1259 (+), 1372 (+), 1617 (+), 1939 (+), 102 (-), 202 (-), 486 (-), 504 (-), 649 (-), 781 (-), 1355 (-), 1397 (-), 1397 (-), 1397 (-), 1397	183 (+), 594 (+), 670 (+), 827 (+), 1358 (+), 1407 (+), 1485 (+), 1731 (+), 279 (-), 579 (-), 1272 (-), 1634 (-), 1806 (-), 1806 (-), 1806 (-), 1806 (-), 1806 (-), 1806 (-),	139 (+), 635 (+), 821 (+), 878 (+), 931 (+), 986 (+), 1193 (+), 1324 (+), 1535 (+), 1611 (+), 1751 (+), 1873 (+), 146 (-), 207 (-), 1199 (-), 1541 (-), 1702 (-), 1707 (-)	419 (+), 1039 (+), 1333 (+), 1342 (+), 1831 (+), 1958 (+), 96 (-), 276 (-), 662 (-), 1175 (-), 1522 (-), 1522 (-), 1522 (-), 1522 (-), 1522 (-), 1522 (-),	566 (+), 1138 (+), 1580 (+), 1640 (+), 1753 (+), 1808 (+), 129 (-), 187 (-), 672 (-), 1461 (-), 1461 (-), 1461 (-), 1461 (-), 1461 (-), 1461 (-),	48 (+), 201 (+), 1264 (+), 76 (-), 263 (-), 455 (-), 476 (-), 523 (-), 707 (-), 1027 (-), 1126 (-), 1163 (-), 1567 (-), 1686 (-), 1695 (-), 1839 (-), 1839 (-), 1839 (-), 1839 (-), 1839 (-), 1839 (-)	189 (+), 197 (+), 202 (+), 357 (+), 630 (+), 848 (+), 1445 (+), 1535 (+), 1615 (+), 1625 (+), 1013 (-), 1058 (-), 1141 (-), 1322 (-), 1796 (-), 1796 (-), 1796 (-), 1796 (-)	16 (+), 532 (+), 1161 (+), 1593 (+), 1706 (+), 322 (-), 508 (-), 974 (-), 1646 (-), 1650 (-), 1849 (-), 1888 (-), 1888 (-), 1888 (-), 1888 (-), 1888 (-),	420 (+), 485 (+), 537 (+), 1134 (+), 1186 (+), 1195 (+), 193 (-), 234 (-), 655 (-), 686 (-), 859 (-), 872 (-), 1752 (-), 1752 (-), 1752 (-), 1752 (-), 1752 (-), 1752 (-),	1760 (+), 177 (-), 197 (-), 295 (-), 339 (-), 663 (-), 1411 (-), 1499 (-), 1894 (-), 1894 (-), 1894 (-), 1894 (-), 1894 (-), 1894 (-), 1894 (-),	810 (+), 960 (+), 979 (+), 1050 (+), 1482 (+), 1638 (+), 1989 (+), 436 (-), 1065 (-), 1740 (-), 1846 (-),	569 (+), 913 (+), 1048 (+), 1341 (+), 1802 (+), 1897 (+), 1965 (+), 776 (-), 793 (-), 844 (-), 1101 (-), 1262 (-), 1772 (-), 1772 (-), 1772 (-), 1772 (-), 1772 (-), 1772 (-),	239 (+), 365 (+), 409 (+), 955 (+), 1039 (+), 29 (-), 80 (-), 261 (-), 527 (-), 1854 (-), 1854 (-), 1854 (-), 1854 (-), 1854 (-), 1854 (-),	10 (+), 225 (+), 350 (+), 372 (+), 808 (+), 1019 (+), 158 (-), 283 (-), 558 (-), 797 (-), 1026 (-), 1077 (-), 1264 (-),	156 (+), 401 (-), 921 (-), 979 (-), 1307 (-), 1406 (-), 1511 (-), 1621 (-), 1621 (-), 1621 (-), 1621 (-), 1621 (-), 1621 (-),	110 (+), 121 (+), 457 (+), 761 (+), 854 (+), 921 (+), 993 (+), 1807 (+), 1855 (+), 1986 (+), 188 (-), 907 (-), 1269 (-), 1735 (-), 1735 (-), 1735 (-), 1735 (-), 1735 (-), 1735 (-),	13 (+), 201 (+), 680 (+), 992 (+), 1197 (+), 426 (-), 881 (-), 1156 (-), 1499 (-), 1592 (-), 1592 (-), 1592 (-), 1592 (-), 1592 (-), 1592 (-),
**WUSATAg**	294 (-), 294 (-), 294 (-), 294 (-), 294 (-),	-	-	-	-	-	-	-	-	-	-	-	-	-	-	737 (-), 737 (-), 737 (-), 737 (-), 737 (-),	-	-	-
**XYLAT**	-	-	-	-	1458 (-), 1458 (-), 1458 (-), 1458 (-), 1458	-	-	-	-	-	-	-	673 (+), 673 (+), 673 (+), 673	-	-	-	-	-	-

**TRANSINITDICOTS**	1396 (-),	761 (-),	1673 (-),	-	-	-	-	-	-	1017 (-), 1611	-	268 (-), 554 (-	-	-	-	-	467 (-),	-	-
**TRANSINITMONOCOT**	1396 (-),	761 (-),	1673 (-),	-	1106 (-), 1449	-	-	133 (-),	2626 (-),	1017 (-), 1611	-	268 (-), 554 (-	-	-	-	-	146 (-), 467 (-	-	681 (-), 1003 (
**UP1ATMSD**	-	-	-	1962 (+),	-	-	-	-	445 (-),	1904 (+),	-	-	-	1816 (+),	-	-	-	-	-
**UP2ATMSD**	-	1401 (-),	-	-	-	-	-	-	-	-	-	1198 (+),	-	-	-	-	-	-	1882 (+),
**UPRMOTIFIIAT**	1846 (+),	1277 (-), 1419 (-),	-	-	-	-	-	-	2213 (-),	210 (-), 701 (-),	1637 (-),	-	-	-	-	-	324 (-),	-	-
**VOZATVPP**	-	-	-	-	-	-	-	-	-	-	-	-	-	-	-	-	-	-	-
**WBBOXPCWRKY1**	-	197 (-),	-	1256 (-),	-	-	675 (+),	-	1428 (+), 2972 (-),	1338 (+),	-	536 (+), 610 (+),	-	-	1405 (-),	-	790 (+),	-	-
**WBOXATNPR1**	263 (-),	3 (+), 181 (+), 198 (-), 999 (-), 1875 (-),	63 (+), 149 (+),	381 (-), 1257 (-),	1139 (+), 1146 (+), 1380 (-),	404 (+), 727 (-),	650 (+), 676 (+), 684 (+), 1558 (+),	373 (+), 1671 (+), 180 (-), 529 (-), 608 (-), 1635 (-),	1429 (+), 495 (-), 1405 (-), 1591 (-), 1839 (-), 2973 (-),	1246 (+), 1339 (+),	66 (+), 1503 (+), 1730 (+),	176 (+), 537 (+), 611 (+), 162 (-), 288 (-),	839 (+), 892 (-),	805 (-), 910 (-),	831 (+), 125 (-), 301 (-), 493 (-), 968 (-), 1406 (-),	1249 (+), 1285 (+), 366 (-),	199 (+), 791 (+), 1426 (-), 1669 (-), 1768 (-),	1341 (+), 224 (-), 970 (-), 1633 (-), 1866 (-),	375 (+), 1480 (+),
**WBOXHVISO1**	1241 (+), 1374 (+), 1504 (+), 1411 (-),	553 (+), 899 (+), 197 (-), 426 (-), 574 (-), 1870 (-), 1874 (-),	307 (-),	1196 (+), 1626 (+), 1256 (-),	1400 (+), 615 (-), 1431 (-),	1071 (+),	1554 (+), 1902 (+),	374 (+), 696 (+), 1448 (+), 1672 (+),	2521 (+), 1041 (-), 1404 (-), 1763 (-), 2972 (-),	1340 (+),	362 (+), 1504 (+), 499 (-), 1515 (-),	538 (+), 734 (+), 287 (-), 1038 (-), 1700 (-),	1442 (+), 677 (-),	366 (+), 931 (+), 1542 (+), 1177 (-),	595 (+), 745 (+), 792 (+), 967 (-), 1405 (-), 1437 (-),	941 (+), 1250 (+), 1352 (+),	1060 (+), 1425 (-), 1578 (-),	1 (+), 1342 (+), 1529 (+), 969 (-), 1865 (-),	376 (+),
**WBOXNTCHN48**	-	1870 (-),	1262 (+), 1768 (+), 307 (-),	1195 (+), 1625 (+),	1264 (+), 1399 (+), 615 (-),	218 (+), 952 (-),	1553 (+), 1870 (-),	-	2520 (+),	1269 (-),	-	-	1273 (+), 1441 (+), 677 (-), 1174 (-),	365 (+),	1217 (+),	940 (+), 256 (-), 609 (-),	-	1528 (+),	1342 (+),
**WBOXNTERF3**	1241 (+), 1374 (+), 1496 (+), 1504 (+), 1411 (-),	4 (+), 182 (+), 553 (+), 899 (+), 986 (+), 197 (-), 426 (-), 574 (-), 1870 (-), 1874 (-)	64 (+), 1263 (+), 1769 (+), 307 (-),	514 (+), 692 (+), 1196 (+), 1626 (+), 1256 (-), 17 (-), 380 (-),	1265 (+), 1400 (+), 615 (-), 1431 (-), 260 (-),	170 (+), 219 (+), 746 (+), 1071 (+), 952 (-),	677 (+), 685 (+), 1554 (+), 1902 (+), 141 (-), 1870 (-),	374 (+), 643 (+), 666 (+), 696 (+), 1448 (+), 1672 (+), 179 (-), 627 (-),	379 (+), 1430 (-), 2521 (+), 1041 (-), 1404 (-), 1763 (-), 2972 (-), 860 (-), 1590 (-),	333 (+), 1340 (+), 345 (-), 1269 (-),	67 (+), 362 (+), 1504 (+), 1731 (+), 499 (-), 1515 (-), 245 (-),	538 (+), 612 (+), 734 (+), 812 (+), 287 (-), 1038 (-), 1700 (-), 273 (-), 280 (-),	1070 (+), 1274 (+), 1442 (+), 677 (-), 70 (-), 1174 (-),	366 (+), 931 (+), 1542 (+), 1177 (-),	595 (+), 745 (+), 792 (+), 1218 (+), 967 (-), 1405 (-), 1437 (-), 234 (-), 300 (-),	941 (+), 1250 (+), 1352 (+), 256 (-), 609 (-),	200 (+), 269 (+), 359 (+), 792 (+), 1060 (+), 1425 (-), 1578 (-), 490 (-), 632 (-),	1 (+), 340 (+), 1342 (+), 1529 (+), 969 (-), 1865 (-), 1146 (-), 1632 (-),	376 (+), 1343 (+), 143 (-),
**WRKY71OS**	117 (+), 682 (+), 874 (+), 1241 (+), 1374 (+), 1496 (+), 1504 (+), 1557 (+), 28 (-), 263 (-), 644 (-), 775 (-), 1412 (-), 1412 (-), 1412 (-), 1412 (-), 1412 (-), 1412 (-),	4 (+), 182 (+), 553 (+), 899 (+), 986 (+), 22 (-), 198 (-), 427 (-), 575 (-), 999 (-), 1146 (-), 1871 (-), 1875 (-), 1875 (-), 1875 (-), 1875 (-), 1875 (-), 1875 (-),	64 (+), 150 (+), 735 (+), 1095 (+), 1263 (+), 1299 (+), 1329 (+), 1604 (+), 1617 (+), 1769 (+), 308 (-), 308 (-), 308 (-), 308 (-), 308 (-), 308 (-),	73 (+), 195 (+), 268 (+), 514 (+), 692 (+), 956 (+), 972 (+), 1196 (+), 1626 (+), 1787 (+), 18 (-), 381 (-), 833 (-), 1257 (-),	852 (+), 982 (+), 1060 (+), 1067 (+), 1140 (+), 1147 (+), 1265 (+), 1348 (+), 1400 (+), 261 (-), 616 (-), 1380 (-), 1432 (-), 1432 (-), 1432 (-), 1432 (-), 1432 (-), 1432 (-)	70 (+), 170 (+), 219 (+), 405 (+), 746 (+), 1071 (+), 163 (-), 537 (-), 670 (-), 727 (-), 953 (-), 1544 (-), 1624 (-), 1624 (-), 1624 (-), 1624 (-), 1624 (-), 1624 (-),	488 (+), 651 (+), 677 (+), 685 (+), 1023 (+), 1058 (+), 1327 (+), 1554 (+), 1559 (+), 1902 (+), 59 (-), 142 (-), 581 (-), 1871 (-),	374 (+), 643 (+), 666 (+), 696 (+), 1448 (+), 1526 (+), 1672 (+), 1741 (+), 1778 (+), 180 (-), 340 (-), 529 (-), 608 (-), 628 (-), 1434 (-), 1635 (-), 1642 (-), 1747 (-), 1747 (-), 1747 (-)	379 (+), 1430 (+), 2521 (+), 369 (-), 495 (-), 861 (-), 1042 (-), 1077 (-), 1405 (-), 1591 (-), 1655 (-), 1764 (-), 1839 (-), 2973 (-), 2973 (-), 2973 (-), 2973 (-), 2973 (-), 2973 (-),	333 (+), 784 (+), 807 (+), 843 (+), 1247 (+), 1340 (+), 152 (-), 194 (-), 346 (-), 800 (-), 1058 (-), 1270 (-), 1720 (-), 1720 (-), 1720 (-), 1720 (-), 1720 (-), 1720 (-),	67 (+), 230 (+), 259 (+), 326 (+), 362 (+), 1504 (+), 1673 (+), 1731 (+), 1975 (+), 246 (-), 322 (-), 500 (-), 1059 (-), 1158 (-), 1383 (-), 1516 (-), 1558 (-),	177 (+), 215 (+), 538 (+), 612 (+), 734 (+), 812 (+), 853 (+), 1045 (+), 1075 (+), 151 (-), 162 (-), 274 (-), 281 (-), 288 (-), 1039 (-), 1701 (-), 1701 (-), 1701 (-), 1701 (-), 1701 (-), 1701 (-)	840 (+), 1070 (+), 1274 (+), 1442 (+), 1513 (+), 1845 (+), 1898 (+), 71 (-), 678 (-), 711 (-), 892 (-), 1175 (-), 1311 (-), 1936 (-),	366 (+), 931 (+), 1008 (+), 1542 (+), 1730 (+), 166 (-), 571 (-), 657 (-), 805 (-), 910 (-), 1081 (-), 1162 (-), 1178 (-), 1178 (-), 1178 (-), 1178 (-), 1178 (-), 1178 (-),	82 (+), 246 (+), 367 (+), 595 (+), 745 (+), 792 (+), 832 (+), 1218 (+), 1811 (+), 125 (-), 235 (-), 301 (-), 493 (-), 859 (-), 968 (-), 1169 (-), 1370 (-), 1406 (-), 1438 (-), 1438 (-), 1438 (-), 1438	416 (+), 941 (+), 1250 (+), 1286 (+), 1319 (+), 1352 (+), 257 (-), 345 (-), 366 (-), 610 (-),	200 (+), 263 (+), 269 (+), 359 (+), 776 (+), 792 (+), 817 (+), 827 (+), 947 (+), 1060 (+), 1337 (+), 491 (-), 633 (-), 721 (-), 1426 (-), 1579 (-), 1669 (-), 1768 (-),	1 (+), 48 (+), 340 (+), 918 (+), 1342 (+), 1346 (+), 1529 (+), 14 (-), 94 (-), 224 (-), 301 (-), 970 (-), 1147 (-), 1534 (-), 1633 (-), 1761 (-), 1866 (-),	376 (+), 728 (+), 850 (+), 1343 (+), 1481 (+), 1725 (+), 144 (-), 952 (-), 1683 (-), 1683 (-), 1683 (-), 1683 (-), 1683 (-), 1683 (-),
**WUSATAg**	-	-	-	1380 (-), 1380 (-), 1380 (-), 1380 (-), 1380 (-),	-	-	346 (-), 346 (-), 346 (-), 346 (-), 346 (-),	-	-	-	-	-	1985 (-), 1985 (-), 1985 (-), 1985 (-), 1985 (-),	-	-	-	1619 (-), 1619 (-), 1619 (-), 1619 (-), 1619 (-),	-	-
**XYLAT**	-	-	-	-	-	-	-	-	-	-	4 (-), 4 (-), 4 (-), 4 (-), 4 (-), 4 (-),	-	-	-	-	1229 (+), 1229 (+), 1229 (+),	-	1477 (-), 1477 (-), 1477 (-), 1477	-

**TRANSINITDICOTS**	-	-	37 (-),	186 (-), 469 (-	-	-	-	-	-	-	-	20 (-),	-	652 (-),	-	1260 (-),	-	-	-
**TRANSINITMONOCOT**	-	-	37 (-),	186 (-), 469 (-	-	-	-	-	-	-	-	20 (-),	407 (-),	652 (-),	-	1260 (-),	-	-	-
**UP1ATMSD**	-	1740 (-), 1817	-	-	-	-	-	-	-	-	-	-	-	-	-	-	-	-	-
**UP2ATMSD**	1853 (+),	-	-	-	26 (-),	72 (+), 481	-	-	-	-	734 (+),	-	-	-	-	1086 (+),	-	-	-
**UPRMOTIFIIAT**	1825 (+),	-	-	1698 (-),	-	-	-	1302 (-),	-	-	-	60 (+), 1834 (-),	-	-	1785 (+), 1961 (+),	-	-	777 (+),	995 (-),
**VOZATVPP**	-	-	-	-	-	-	-	-	-	-	-	-	-	-	-	-	-	-	-
**WBBOXPCWRKY1**	-	1624 (-),	323 (-), 672 (-),	-	-	-	-	529 (-),	-	-	901 (-),	-	-	1185 (+), 1533 (-),	417 (+), 592 (+), 1437 (-),	1218 (+),	1036 (+), 1671 (+),	-	-
**WBOXATNPR1**	1164 (-), 1774 (-),	248 (+), 256 (+), 701 (+), 1625 (-),	1627 (+), 324 (-), 344 (-), 673 (-), 693 (-),	901 (+), 1500 (+), 1507 (+),	1885 (+), 820 (-), 1020 (-), 1497 (-),	188 (-),	-	1135 (+), 530 (-), 705 (-), 1023 (-), 1601 (-),	1475 (+),	902 (-),	1203 (+), 1576 (+), 700 (-),	563 (-),	71 (+), 1124 (+), 1186 (+), 343 (-), 1534 (-),	418 (+), 593 (+), 961 (+), 49 (-), 1346 (-), 1438 (-), 1500 (-), 1535 (-), 1594 (-)	166 (+), 390 (+), 657 (+), 854 (+), 1219 (+), 292 (-), 698 (-), 707 (-), 979 (-)	881 (+), 1037 (+), 1203 (+), 1672 (+), 13 (-), 901 (-), 914 (-), 1783 (-),	45 (-), 63 (-), 1278 (-), 1516 (-),	109 (+), 1476 (+), 1887 (+), 66 (-),	1372 (-),
**WBOXHVISO1**	429 (-),	1397 (+), 605 (-), 1624 (-), 1637 (-),	323 (-),	1383 (+), 1501 (+), 1587 (+), 1722 (+), 1044 (-),	935 (+), 762 (-),	637 (+), 799 (+), 1260 (-),	108 (-),	1706 (+), 704 (-), 1022 (-),	250 (-),	1372 (-),	1407 (+), 646 (-), 699 (-),	1462 (-),	451 (+), 1125 (+), 835 (-), 839 (-), 843 (-), 1533 (-),	594 (+), 1316 (-), 1437 (-), 1593 (-),	302 (+), 791 (+), 291 (-), 928 (-), 1155 (-),	1038 (+), 1217 (+), 1158 (-), 1434 (-), 1782 (-),	834 (+), 1685 (+), 44 (-), 394 (-), 1620 (-),	830 (+),	67 (+), 418 (-),
**WBOXNTCHN48**	-	605 (-),	-	1382 (+),	927 (+),	1748 (-),	499 (-),	1497 (+),	114 (-),	278 (+), 351 (-),	-	1738 (+),	835 (-), 839 (-), 843 (-),	-	301 (+),	1216 (+), 1434 (-),	266 (+), 833 (+), 1684 (+), 394 (-), 1032 (	-	418 (-),
**WBOXNTERF3**	429 (-), 58 (-), 1773 (-),	1397 (+), 1665 (+), 605 (-), 1624 (-), 1637 (-),	613 (+), 323 (-), 645 (-), 672 (-),	1383 (+), 1501 (+), 1587 (+), 1722 (+), 1044 (-), 1514 (-),	928 (+), 935 (+), 1886 (+), 762 (-), 1496 (-), 1768 (-),	236 (+), 376 (+), 637 (+), 799 (+), 1260 (-), 1748 (-),	108 (-), 286 (-), 499 (-), 1671 (-),	970 (+), 1498 (+), 1706 (+), 704 (-), 1022 (-), 529 (-), 1349 (-),	1476 (+), 250 (-), 114 (-),	191 (+), 279 (+), 1372 (-), 351 (-), 901 (-),	1407 (+), 646 (-), 699 (-), 549 (-),	1739 (+), 1886 (+), 1462 (-),	72 (+), 117 (+), 451 (+), 1125 (+), 1187 (+), 835 (-), 839 (-), 843 (-), 1533 (-)	419 (+), 594 (+), 962 (+), 1316 (-), 1437 (-), 1593 (-), 48 (-), 1345 (-), 1499 (-),	302 (+), 658 (+), 791 (+), 855 (+), 1075 (+), 1220 (+), 291 (-), 928 (-), 1155 (-), 706 (-)	842 (+), 1038 (+), 1673 (+), 1158 (-), 1434 (-), 1782 (-),	267 (+), 817 (+), 834 (+), 1685 (+), 44 (-), 394 (-), 1620 (-), 1032 (-),	110 (+), 830 (+),	67 (+), 418 (-), 1371 (-),
**WRKY71OS**	114 (+), 441 (+), 1321 (+), 59 (-), 430 (-), 1164 (-), 1774 (-), 1774 (-), 1774 (-), 1774 (-), 1774 (-), 1774 (-),	249 (+), 257 (+), 702 (+), 1397 (+), 1665 (+), 606 (-), 945 (-), 1625 (-), 1638 (-), 1638 (-), 1638 (-), 1638 (-), 1638 (-), 1638 (-),	522 (+), 613 (+), 1379 (+), 1628 (+), 1643 (+), 1895 (+), 324 (-), 344 (-), 646 (-), 673 (-), 693 (-), 816 (-), 1036 (-), 1664 (-), 1997 (-),	506 (+), 902 (+), 1383 (+), 1501 (+), 1508 (+), 1587 (+), 1722 (+), 725 (-), 1001 (-), 1045 (-), 1057 (-), 1515 (-), 1582 (-),	928 (+), 935 (+), 1886 (+), 763 (-), 820 (-), 897 (-), 1020 (-), 1227 (-), 1235 (-), 1497 (-), 1769 (-), 1778 (-),	236 (+), 376 (+), 637 (+), 746 (+), 799 (+), 188 (-), 673 (-), 764 (-), 871 (-), 1185 (-), 1261 (-), 1749 (-),	57 (+), 361 (+), 798 (+), 866 (+), 1286 (+), 100 (-), 109 (-), 287 (-), 335 (-), 397 (-), 500 (-), 509 (-), 1421 (-), 1672 (-), 1672 (-), 1672 (-), 1672 (-), 1672 (-), 1672 (-),	970 (+), 1136 (+), 1454 (+), 1498 (+), 1706 (+), 530 (-), 705 (-), 1023 (-), 1350 (-), 1601 (-), 1912 (-),	70 (+), 828 (+), 1400 (+), 1476 (+), 1713 (+), 24 (-), 115 (-), 251 (-), 846 (-), 846 (-), 846 (-), 846 (-), 846 (-), 846 (-),	191 (+), 279 (+), 684 (+), 1271 (+), 352 (-), 902 (-), 1024 (-), 1188 (-), 1373 (-), 1985 (-), 1985 (-), 1985 (-), 1985 (-), 1985 (-), 1985 (-),	1204 (+), 1407 (+), 1577 (+), 1761 (+), 550 (-), 647 (-), 700 (-), 1870 (-),	1408 (+), 1739 (+), 1886 (+), 563 (-), 1084 (-), 1463 (-), 1463 (-), 1463 (-), 1463 (-), 1463 (-), 1463 (-),	72 (+), 117 (+), 451 (+), 470 (+), 1125 (+), 1187 (+), 1320 (+), 1652 (+), 343 (-), 535 (-), 836 (-), 840 (-), 844 (-), 1534 (-), 1944 (-), 1944 (-), 1944 (-), 1944 (-), 1944 (-), 1944	419 (+), 594 (+), 962 (+), 49 (-), 1317 (-), 1346 (-), 1438 (-), 1500 (-), 1535 (-), 1594 (-),	167 (+), 302 (+), 391 (+), 658 (+), 791 (+), 855 (+), 1075 (+), 1220 (+), 1943 (+), 292 (-), 698 (-), 707 (-), 929 (-), 940 (-), 1156 (-), 1332 (-), 1754 (-), 1754 (-), 1754 (-), 1754 (-)	842 (+), 882 (+), 1038 (+), 1204 (+), 1217 (+), 1524 (+), 1673 (+), 1682 (+), 1741 (+), 13 (-), 901 (-), 914 (-), 1104 (-), 1159 (-), 1435 (-), 1783 (-), 1783 (-), 1783 (-), 1783	267 (+), 817 (+), 834 (+), 958 (+), 1685 (+), 41 (-), 45 (-), 63 (-), 395 (-), 591 (-), 828 (-), 1033 (-), 1278 (-), 1516 (-), 1621 (-),	110 (+), 830 (+), 1477 (+), 1644 (+), 1888 (+), 66 (-), 1032 (-), 1156 (-), 1311 (-), 1440 (-), 1760 (-), 1760 (-), 1760 (-), 1760 (-), 1760 (-), 1760 (-),	67 (+), 393 (-), 419 (-), 1372 (-), 1704 (-), 1760 (-),
**WUSATAg**	-	-	1040 (+), 1040 (+), 1040 (+), 1040 (+),	-	-	1837 (+), 1837 (+), 1837 (+), 1837 (+),	-	1087 (-), 1087 (-), 1087 (-), 1087 (-), 1087 (-), 1087 (-),	-	-	1083 (-), 1083 (-), 1083 (-), 1083 (-), 1083 (-),	-	-	-	-	-	570 (-),	-	-
**XYLAT**	-	-	-	1158 (-), 1158 (-), 1158 (-), 1158	754 (-), 754 (-), 754 (-), 754 (-), 754 (-),	-	-	-	-	-	-	-	-	-	395 (-), 395 (-), 395 (-), 395 (-), 395 (-),	-	-	-	1966 (+), 1966 (+), 1966 (+),

**TRANSINITDICOTS**	-	-	-	-	-	-	-	722 (-),	-	421 (-),	-	-	-	-	-	-	-	-	1039 (-),
**TRANSINITMONOCOT**	1088 (-),	-	-	-	1267 (-),	-	-	722 (-),	-	421 (-),	-	1608 (-),	-	-	-	864 (-),	-	-	1030 (-), 1039
**UP1ATMSD**	-	-	293 (+),	-	1862 (+),	1572 (+),	1665 (+),	-	-	-	1848 (+),	-	-	-	-	-	-	-	1692 (+),
**UP2ATMSD**	-	-	-	-	-	-	-	-	-	582 (+), 499 (-	-	-	1993 (+),	-	1972 (+),	1053 (+),	-	-	-
**UPRMOTIFIIAT**	569 (-),	-	-	-	-	1755 (-), 1782 (-),	-	836 (-),	-	549 (+),	-	275 (-),	1827 (+),	1608 (+), 1715 (+), 1810 (+),	-	-	-	-	-
**VOZATVPP**	-	-	-	-	-	-	-	-	700 (+), 700 (-	-	-	-	-	-	-	-	-	-	-
**WBBOXPCWRKY1**	112 (+),	-	-	-	-	-	1456 (+), 1439 (-), 1578	23 (+),	1160 (+), 1666 (-),	-	-	758 (+), 546 (-),	-	141 (-), 1065 (-),	-	568 (-),	247 (-),	170 (+), 831 (+),	-
**WBOXATNPR1**	113 (+), 908 (+), 232 (-), 450 (-), 606 (-), 1405 (-), 1706 (-),	115 (+), 1604 (+), 834 (-), 1587 (-),	1052 (+),	790 (+), 918 (+), 1161 (+), 1449 (+), 1631 (+), 1750 (+),	221 (+), 908 (+), 956 (+), 472 (-), 1768 (-),	937 (+), 1478 (+), 1700 (+),	623 (+), 1134 (+), 1457 (+), 525 (-), 1440 (-), 1579 (-), 1600 (-),	24 (+),	1062 (+), 1161 (+), 1667 (-),	1048 (+), 825 (-), 993 (-),	523 (-), 1288 (-),	759 (+), 1312 (+), 453 (-), 547 (-),	386 (+), 760 (-), 887 (-),	221 (+), 1567 (+), 1585 (+), 15 (-), 142 (-), 237 (-), 1066 (-), 1828 (-),	729 (+), 935 (+), 990 (+), 924 (-), 944 (-), 1504 (-),	418 (+), 1145 (+), 569 (-), 667 (-),	1185 (+), 1444 (+), 248 (-), 1386 (-), 1983 (-),	171 (+), 310 (+), 832 (+), 939 (+), 946 (-), 1934 (-),	852 (+), 14 (-), 196 (-),
**WBOXHVISO1**	114 (+), 909 (+), 1335 (-),	250 (+), 576 (+),	535 (+), 609 (+), 650 (+), 779 (+), 805 (+), 885 (+), 936 (+), 1053 (+), 1065 (-)	791 (+), 912 (+), 919 (+), 1249 (+), 1632 (+), 1884 (+),	4 (+), 222 (+), 260 (+), 957 (+), 1421 (+),	938 (+),	656 (+), 1458 (+), 1439 (-), 1937 (-),	809 (-),	1162 (+), 1733 (-),	608 (+), 1362 (-),	-	325 (+), 733 (+), 760 (+), 452 (-), 1735 (-),	306 (+), 929 (+), 1702 (+),	1586 (+), 1590 (+), 14 (-), 141 (-), 1031 (-),	523 (+), 966 (+), 1644 (+), 770 (-), 1503 (-),	1146 (+), 1306 (+), 568 (-), 660 (-),	43 (+), 1910 (+), 247 (-), 1978 (-), 1982 (-),	172 (+), 286 (+), 311 (+), 1879 (+), 945 (-), 1763 (-), 1929 (-), 1933 (-),	-
**WBOXNTCHN48**	1076 (+), 1335 (-), 1549 (-),	-	873 (+),	1780 (+), 1883 (+), 1890 (+),	263 (+), 528 (+), 546 (+), 1114 (-),	1729 (+),	655 (+), 1682 (+),	1156 (+),	-	1942 (+),	-	732 (+), 1136 (+), 1735 (-), 1562 (-),	305 (+), 1701 (+),	1589 (+),	965 (+), 770 (-), 1600 (-),	660 (-),	1909 (+), 1978 (-),	1878 (+), 1763 (-), 1929 (-),	-
**WBOXNTERF3**	114 (+), 489 (+), 909 (+), 1077 (+), 1335 (-), 605 (-), 1549 (-),	250 (+), 576 (+), 1605 (+), 682 (-), 1586 (-),	535 (+), 609 (+), 650 (+), 779 (+), 805 (+), 874 (+), 885 (+), 936 (+), 1053 (+), 1065 (-)	791 (+), 912 (+), 919 (+), 1162 (+), 1249 (+), 1632 (+), 1781 (+), 1884 (-)	4 (+), 222 (+), 260 (+), 264 (+), 529 (+), 547 (+), 909 (+), 957 (+), 1421 (+), 204 (-), 1114 (-)	377 (+), 938 (+), 1730 (+),	656 (+), 1458 (+), 1683 (+), 1439 (-), 1937 (-), 1578 (-), 1599 (-), 1788 (-),	25 (+), 1157 (+), 1181 (+), 809 (-),	20 (+), 1162 (+), 1733 (-), 1666 (-),	608 (+), 1878 (+), 1943 (+), 1362 (-),	393 (+), 1201 (-), 1287 (-),	325 (+), 733 (+), 760 (+), 1137 (+), 452 (-), 1735 (-), 546 (-), 1562 (-),	260 (+), 306 (+), 929 (+), 1702 (+), 1902 (+), 759 (-),	1568 (+), 1586 (+), 1590 (+), 14 (-), 141 (-), 1031 (-), 236 (-), 1065 (-), 1574 (-)	523 (+), 730 (+), 826 (+), 966 (+), 1644 (+), 770 (-), 1503 (-), 314 (-), 1600 (-),	419 (+), 1146 (+), 1306 (+), 568 (-), 660 (-),	43 (+), 1910 (+), 247 (-), 1978 (-), 1982 (-),	172 (+), 286 (+), 311 (+), 833 (+), 1879 (+), 945 (-), 1763 (-), 1929 (-), 1933 (-),	1208 (+), 13 (-), 264 (-),
**WRKY71OS**	114 (+), 489 (+), 909 (+), 1077 (+), 232 (-), 450 (-), 606 (-), 703 (-), 1336 (-), 1405 (-), 1550 (-), 1706 (-), 1893 (-), 1893 (-), 1893 (-), 1893 (-), 1893 (-), 1893 (-),	116 (+), 250 (+), 576 (+), 726 (+), 743 (+), 944 (+), 1605 (+), 1713 (+), 62 (-), 69 (-), 244 (-), 683 (-), 834 (-), 1499 (-), 1587 (-), 1587 (-), 1587 (-), 1587 (-), 1587 (-), 1587 (-),	489 (+), 535 (+), 572 (+), 609 (+), 623 (+), 650 (+), 681 (+), 779 (+), 790 (+), 805 (+), 834 (+), 874 (+), 885 (+), 909 (+), 936 (+), 967 (+), 1053 (+), 1065 (+), 1091 (+), 1170 (-)	501 (+), 791 (+), 912 (+), 919 (+), 1162 (+), 1249 (+), 1450 (+), 1632 (+), 1751 (+), 1781 (+), 1884 (+), 1891 (+), 763 (-), 1084 (-), 1128 (-), 1269 (-), 1676 (-),	4 (+), 222 (+), 260 (+), 264 (+), 529 (+), 547 (+), 661 (+), 867 (+), 909 (+), 957 (+), 1421 (+), 205 (-), 472 (-), 992 (-), 1115 (-), 1345 (-), 1560 (-), 1768 (-),	377 (+), 732 (+), 938 (+), 1479 (+), 1701 (+), 1730 (+), 1941 (+), 1300 (-), 1549 (-), 1549 (-), 1549 (-), 1549 (-), 1549 (-), 1549 (-),	624 (+), 656 (+), 763 (+), 1135 (+), 1458 (+), 1683 (+), 32 (-), 525 (-), 1210 (-), 1440 (-), 1579 (-), 1600 (-), 1789 (-), 1938 (-), 1938 (-), 1938 (-), 1938 (-), 1938 (-), 1938 (-),	25 (+), 1157 (+), 1181 (+), 810 (-), 891 (-), 1423 (-), 1423 (-), 1423 (-), 1423 (-), 1423 (-), 1423 (-),	20 (+), 335 (+), 1063 (+), 1162 (+), 240 (-), 252 (-), 969 (-), 1667 (-), 1734 (-), 1734 (-), 1734 (-), 1734 (-), 1734 (-), 1734 (-),	435 (+), 608 (+), 1049 (+), 1114 (+), 1170 (+), 1596 (+), 1878 (+), 1943 (+), 825 (-), 881 (-), 993 (-), 1363 (-), 1652 (-), 1652 (-), 1652 (-), 1652 (-), 1652 (-), 1652 (-)	206 (+), 594 (+), 1337 (+), 394 (-), 523 (-), 1202 (-), 1288 (-), 1973 (-), 1973 (-), 1973 (-), 1973 (-), 1973 (-), 1973 (-),	325 (+), 448 (+), 690 (+), 733 (+), 760 (+), 1137 (+), 1313 (+), 1720 (+), 453 (-), 547 (-), 1033 (-), 1563 (-), 1724 (-), 1736 (-), 1736 (-), 1736 (-), 1736 (-), 1736 (-), 1736 (-),	69 (+), 260 (+), 306 (+), 387 (+), 929 (+), 978 (+), 1593 (+), 1702 (+), 1865 (+), 1902 (+), 760 (-), 887 (-), 1259 (-), 1380 (-), 1611 (-), 1628 (-), 1749 (-), 1755 (-), 1897 (-), 1897	222 (+), 1442 (+), 1568 (+), 1586 (+), 1590 (+), 1851 (+), 15 (-), 142 (-), 237 (-), 295 (-), 912 (-), 1032 (-), 1066 (-), 1455 (-), 1474 (-), 1575 (-), 1828 (-),	523 (+), 730 (+), 826 (+), 936 (+), 966 (+), 991 (+), 1644 (+), 315 (-), 340 (-), 771 (-), 924 (-), 944 (-), 1382 (-), 1504 (-), 1601 (-),	419 (+), 1146 (+), 1306 (+), 1582 (+), 1619 (+), 267 (-), 569 (-), 661 (-), 667 (-), 667 (-), 667 (-), 667 (-), 667 (-), 667 (-),	43 (+), 1186 (+), 1445 (+), 1910 (+), 248 (-), 1386 (-), 1979 (-), 1983 (-), 1983 (-), 1983 (-), 1983 (-), 1983 (-), 1983 (-),	172 (+), 286 (+), 311 (+), 634 (+), 833 (+), 940 (+), 1550 (+), 1879 (+), 119 (-), 946 (-), 1180 (-), 1764 (-), 1930 (-), 1934 (-), 1934 (-), 1934 (-), 1934 (-), 1934 (-), 1934 (-),	52 (+), 853 (+), 1208 (+), 14 (-), 196 (-), 265 (-), 890 (-), 1588 (-), 1588 (-), 1588 (-), 1588 (-), 1588 (-), 1588 (-),
**WUSATAg**	-	-	1180 (+), 1180 (+), 1180 (+), 1180 (+),	1714 (-),	40 (-), 40 (-), 40 (-), 40 (-), 40 (-), 40 (-),	-	-	-	-	-	-	-	-	1740 (-), 1740 (-), 1740 (-), 1740 (-), 1740 (-),	1692 (+), 1692 (+), 1692 (+), 1692 (+),	-	-	-	-
**XYLAT**	-	-	-	1544 (-), 1544 (-), 1544 (-), 1544	-	-	-	-	-	-	-	-	-	-	-	-	-	-	-

**TRANSINITDICOTS**	-	-	-	294 (-), 315 (-	-	-	-	-	1064 (-),	-	872 (-),	599 (-),	-	-	-	-	1677 (-),	-	-
**TRANSINITMONOCOT**	162 (-),	-	-	294 (-), 315 (-	666 (-),	-	-	-	1064 (-),	-	872 (-),	599 (-),	-	312 (-), 1576 (	-	-	1677 (-),	-	-
**UP1ATMSD**	-	-	-	-	-	-	-	-	-	608 (+),	1910 (+),	1611 (+),	-	1551 (+),	-	-	-	-	-
**UP2ATMSD**	-	-	-	-	602 (-),	-	400 (-), 469 (-	-	-	-	-	-	1098 (-),	-	-	-	-	-	-
**UPRMOTIFIIAT**	-	-	-	-	-	1124 (+), 260 (-),	-	-	-	-	1761 (-),	-	-	-	-	-	1656 (+),	-	-
**VOZATVPP**	-	-	-	-	-	-	-	-	-	-	-	-	-	-	-	-	-	-	-
**WBBOXPCWRKY1**	-	-	-	1147 (+),	1399 (+),	1103 (-),	990 (+), 1805 (+), 786 (-),	-	-	-	86 (+), 1286 (-),	-	-	-	-	17 (+), 271 (-),	-	-	-
**WBOXATNPR1**	1114 (+), 1742 (-),	1129 (+), 1291 (+), 1324 (+), 1924 (-),	-	1148 (+), 1662 (+),	1400 (+), 1651 (+), 1867 (+), 1411 (-),	790 (+), 596 (-), 1104 (-), 1663 (-),	991 (+), 1806 (+), 652 (-), 787 (-),	1095 (+), 1482 (-), 1584 (-),	264 (+), 1093 (+), 1664 (-),	69 (+), 87 (+), 1173 (+), 1287 (-),	1047 (+), 1982 (-),	207 (+), 183 (-), 803 (-), 1910 (-),	1929 (-),	476 (+), 755 (+), 1013 (+), 1210 (+), 864 (-),	18 (+), 749 (+), 947 (+), 272 (-), 1159 (-),	299 (+), 628 (+), 1105 (+), 553 (-),	1053 (+), 1452 (+), 909 (-), 1561 (-),	613 (+), 1293 (+), 1377 (+), 339 (-), 655 (-), 839 (-), 1147 (-), 1514 (-), 1685 (-)	303 (+), 831 (+), 846 (+),
**WBOXHVISO1**	1660 (+), 1741 (-),	313 (+), 1130 (+), 1179 (+), 1325 (+), 1186 (-),	360 (-), 1410 (-),	1149 (+), 1024 (-), 1760 (-),	1395 (+), 1401 (+), 268 (-), 913 (-),	515 (+), 1940 (+), 458 (-), 595 (-), 807 (-),	1807 (+), 600 (-),	1096 (+), 191 (-), 1933 (-),	191 (+), 1663 (-), 1813 (-), 1817 (-),	70 (+), 88 (+), 1550 (+), 1839 (+),	1010 (+), 19 (-),	589 (+), 1717 (+),	1681 (+),	477 (+), 1599 (+), 362 (-), 505 (-), 805 (-), 1284 (-), 1869 (-),	19 (+), 1176 (+), 418 (-), 1765 (-),	424 (+), 629 (+), 552 (-), 1244 (-), 1861 (-),	199 (+), 1535 (+), 908 (-),	1294 (+), 338 (-), 1146 (-), 1513 (-),	304 (+), 1508 (+), 1483 (-),
**WBOXNTCHN48**	1336 (-),	1375 (-), 1678 (-),	962 (-),	1760 (-),	575 (-),	807 (-),	1597 (+),	-	190 (+), 1813 (-), 1817 (-),	892 (+), 1549 (+), 877 (-),	1009 (+),	1716 (+),	-	1598 (+), 505 (-), 805 (-),	1765 (-),	1861 (-), 1773 (-),	198 (+), 1534 (+),	-	1507 (+),
**WBOXNTERF3**	1660 (+), 1741 (-), 8 (-), 1336 (-),	313 (+), 1130 (+), 1179 (+), 1325 (+), 1186 (-), 278 (-), 1375 (-), 1678 (-),	1681 (+), 360 (-), 1410 (-), 962 (-),	1149 (+), 1663 (+), 1024 (-), 1760 (-), 1212 (-), 1781 (-),	1395 (+), 1401 (+), 1652 (+), 268 (-), 913 (-), 41 (-), 304 (-), 575 (-),	426 (+), 515 (+), 1940 (+), 458 (-), 595 (-), 807 (-), 1103 (-),	992 (+), 1598 (+), 1807 (+), 600 (-), 786 (-), 1313 (-),	1096 (+), 1397 (+), 191 (-), 1933 (-), 1481 (-),	191 (+), 265 (+), 1663 (-), 1813 (-), 1817 (-),	70 (+), 88 (+), 517 (+), 893 (+), 1550 (+), 1839 (+), 877 (-), 1286 (-),	1010 (+), 19 (-), 50 (-), 1238 (-),	589 (+), 1717 (+), 79 (-), 182 (-), 743 (-),	1681 (+), 1788 (+), 487 (-),	477 (+), 1014 (+), 1599 (+), 362 (-), 505 (-), 805 (-), 1284 (-), 1869 (-), 105 (-), 863 (-), 1032	19 (+), 948 (+), 418 (-), 1765 (-), 271 (-),	300 (+), 424 (+), 629 (+), 1889 (+), 552 (-), 1244 (-), 1861 (-), 240 (-), 1773 (-),	199 (+), 1321 (+), 1535 (+), 1881 (+), 908 (-), 540 (-),	1294 (+), 338 (-), 1146 (-), 1513 (-), 654 (-), 979 (-),	304 (+), 577 (+), 643 (+), 832 (+), 1508 (+), 1483 (-),
**WRKY71OS**	1115 (+), 1272 (+), 1660 (+), 1721 (+), 9 (-), 400 (-), 995 (-), 1337 (-), 1347 (-), 1725 (-), 1742 (-), 1742 (-), 1742 (-), 1742 (-), 1742 (-), 1742 (-),	271 (+), 313 (+), 416 (+), 1130 (+), 1179 (+), 1292 (+), 1325 (+), 1667 (+), 50 (-), 279 (-), 785 (-), 1187 (-), 1350 (-), 1376 (-), 1679 (-), 1924 (-),	293 (+), 635 (+), 813 (+), 1341 (+), 1681 (+), 9 (-), 357 (-), 361 (-), 963 (-), 1411 (-), 1880 (-), 1880 (-), 1880 (-), 1880 (-), 1880 (-), 1880 (-),	1121 (+), 1149 (+), 1663 (+), 1025 (-), 1213 (-), 1282 (-), 1761 (-), 1782 (-), 1782 (-), 1782 (-), 1782 (-), 1782 (-), 1782 (-),	794 (+), 918 (+), 1022 (+), 1059 (+), 1372 (+), 1395 (+), 1401 (+), 1652 (+), 1709 (+), 1868 (+), 1950 (+), 42 (-), 269 (-), 305 (-), 576 (-), 752 (-), 914 (-), 1036 (-)	426 (+), 515 (+), 603 (+), 791 (+), 1641 (+), 1678 (+), 1940 (+), 233 (-), 459 (-), 596 (-), 808 (-), 1104 (-), 1663 (-), 1663 (-), 1663 (-), 1663 (-), 1663 (-), 1663 (-),	34 (+), 823 (+), 992 (+), 1579 (+), 1598 (+), 1654 (+), 1807 (+), 601 (-), 652 (-), 787 (-), 1314 (-), 1658 (-),	1096 (+), 1397 (+), 192 (-), 1482 (-), 1584 (-), 1934 (-), 1934 (-), 1934 (-), 1934 (-), 1934 (-), 1934 (-),	191 (+), 265 (+), 563 (+), 1094 (+), 1156 (+), 242 (-), 1664 (-), 1814 (-), 1818 (-), 1984 (-), 1984 (-), 1984 (-), 1984 (-), 1984 (-), 1984 (-),	10 (+), 70 (+), 88 (+), 517 (+), 893 (+), 1174 (+), 1537 (+), 1550 (+), 1794 (+), 1839 (+), 242 (-), 878 (-), 1041 (-), 1287 (-), 1508 (-), 1508 (-), 1508 (-), 1508 (-), 1508 (-), 1508	98 (+), 1010 (+), 1048 (+), 1446 (+), 20 (-), 51 (-), 224 (-), 231 (-), 1239 (-), 1377 (-), 1384 (-), 1982 (-), 1982 (-), 1982 (-), 1982 (-), 1982 (-), 1982 (-),	208 (+), 589 (+), 1717 (+), 1927 (+), 80 (-), 95 (-), 183 (-), 744 (-), 803 (-), 1135 (-), 1910 (-),	63 (+), 544 (+), 1339 (+), 1681 (+), 1788 (+), 488 (-), 567 (-), 693 (-), 1929 (-), 1929 (-), 1929 (-), 1929 (-), 1929 (-), 1929 (-),	477 (+), 756 (+), 1014 (+), 1211 (+), 1599 (+), 1975 (+), 106 (-), 363 (-), 506 (-), 806 (-), 864 (-), 945 (-), 1033 (-), 1285 (-), 1642 (-), 1870 (-),	19 (+), 750 (+), 948 (+), 1176 (+), 1623 (+), 87 (-), 204 (-), 272 (-), 419 (-), 519 (-), 1159 (-), 1489 (-), 1627 (-), 1766 (-), 1766 (-), 1766 (-), 1766 (-), 1766 (-), 1766 (-),	300 (+), 424 (+), 629 (+), 1106 (+), 1143 (+), 1548 (+), 1693 (+), 1889 (+), 1939 (+), 241 (-), 292 (-), 533 (-), 553 (-), 1245 (-), 1350 (-), 1774 (-), 1862 (-),	130 (+), 199 (+), 1054 (+), 1321 (+), 1453 (+), 1535 (+), 1881 (+), 180 (-), 493 (-), 541 (-), 706 (-), 909 (-), 1561 (-),	614 (+), 1204 (+), 1274 (+), 1294 (+), 1378 (+), 339 (-), 655 (-), 839 (-), 896 (-), 980 (-), 1147 (-), 1514 (-), 1668 (-), 1685 (-), 1685 (-), 1685 (-), 1685 (-), 1685 (-), 1685 (-),	304 (+), 438 (+), 577 (+), 643 (+), 832 (+), 847 (+), 1150 (+), 1508 (+), 1727 (+), 1782 (+), 1954 (+), 168 (-), 1484 (-), 1484 (-), 1484 (-), 1484 (-), 1484 (-), 1484 (-),
**WUSATAg**	-	1416 (+), 1416 (+), 1416 (+), 1416 (+),	-	-	-	-	995 (-), 995 (-), 995 (-), 995 (-), 995 (-),	-	-	-	-	-	-	-	-	179 (-), 1706 (-), 1706 (-), 1706 (-), 1706 (-), 1706 (-),	414 (+), 414 (+), 414 (+), 414 (+), 414 (+),	-	-
**XYLAT**	-	-	-	-	-	-	-	-	-	-	-	-	-	761 (+), 761 (+), 761 (+), 761	-	-	-	-	-

**TRANSINITDICOTS**	-	-	-	-	-	-	-	790 (-),	-	-	-	-	-	-	-	-	-	-
**TRANSINITMONOCOT**	-	-	-	-	-	-	-	790 (-),	1130 (-),	-	-	-	-	-	1314 (-), 1351	-	1881 (-),	29 (-), 700 (-),
**UP1ATMSD**	-	-	-	-	-	96 (-),	-	1848 (+),	-	-	-	-	721 (-),	-	-	1870 (+),	1741 (-),	-
**UP2ATMSD**	-	-	-	-	-	-	-	-	-	-	-	-	-	-	-	-	-	-
**UPRMOTIFIIAT**	-	-	1863 (+),	-	-	521 (-),	-	-	-	-	999 (+),	-	-	1113 (-),	1307 (+), 1344 (+),	-	-	1726 (+),
**VOZATVPP**	-	-	-	-	-	-	-	-	1907 (+),	-	-	-	-	-	-	-	-	-
**WBBOXPCWRKY1**	-	898 (+),	87 (+),	1603 (-),	367 (+),	-	77 (+),	550 (+),	-	-	1219 (-),	1069 (+), 1447 (+),	223 (+), 107 (-),	-	-	-	-	-
**WBOXATNPR1**	188 (+), 1113 (+), 1128 (+),	20 (+), 187 (+), 899 (+), 506 (-), 1064 (-),	70 (+), 88 (+), 918 (-),	750 (+), 969 (+), 1604 (-),	368 (+), 459 (+), 1525 (+), 1609 (+), 1592 (-),	248 (+), 1535 (-),	78 (+), 953 (+), 1557 (+), 1816 (+), 1301 (-),	163 (+), 551 (+), 1474 (-),	502 (+), 920 (+), 1727 (+), 1940 (+),	336 (+),	1944 (+), 1220 (-), 1550 (-), 1976 (-),	564 (+), 1070 (+), 1220 (+), 1448 (+), 94 (-), 253 (-), 599 (-),	224 (+), 108 (-), 172 (-), 267 (-), 1892 (-),	990 (+), 224 (-), 476 (-), 584 (-), 1328 (-),	1038 (+), 1572 (+),	1110 (+), 1125 (+), 1779 (+), 1990 (-),	1073 (+), 1088 (+), 1625 (+), 1836 (-),	908 (+), 1211 (+), 268 (-),
**WBOXHVISO1**	1129 (+), 1924 (+), 1899 (-),	686 (+), 505 (-),	89 (+), 482 (-), 958 (-),	890 (-), 1603 (-),	-	249 (+),	79 (+), 248 (+), 990 (+), 1416 (+), 1558 (+), 1817 (+),	164 (+), 552 (+),	503 (+), 539 (-), 570 (-),	326 (+), 652 (+), 1733 (+), 1838 (-),	1282 (+), 1643 (+), 678 (-),	1071 (+), 1221 (+), 1449 (+), 598 (-),	413 (+), 107 (-), 1493 (-),	1006 (-),	1496 (-),	1628 (+), 1438 (-),	1987 (-),	1130 (+), 267 (-),
**WBOXNTCHN48**	1899 (-),	-	482 (-),	1642 (+),	-	653 (-), 1392 (-),	1390 (+),	1925 (+),	1737 (+), 539 (-),	651 (+), 1838 (-),	1642 (+), 50 (-), 111 (-), 859 (-),	718 (-),	-	424 (-), 1252 (-),	1273 (+), 389 (-), 856 (-),	1627 (+), 1438 (-),	1987 (-),	-
**WBOXNTERF3**	1114 (+), 1129 (+), 1924 (+), 1899 (-),	686 (+), 900 (+), 505 (-), 1063 (-),	71 (+), 89 (+), 352 (+), 482 (-), 958 (-),	970 (+), 1553 (+), 1643 (+), 890 (-), 1603 (-), 402 (-),	369 (+), 460 (+), 1303 (+), 1591 (-), 1677 (-),	249 (+), 1928 (+), 406 (-), 653 (-), 1392 (-), 1534 (-),	79 (+), 248 (+), 990 (+), 1391 (+), 1416 (+), 1558 (+), 1817 (+), 1300 (-)	164 (+), 552 (+), 1926 (+), 324 (-),	311 (+), 503 (+), 1738 (+), 539 (-), 570 (-), 1386 (-), 1607 (-),	326 (+), 652 (+), 1733 (+), 1838 (-),	1282 (+), 1643 (+), 678 (-), 50 (-), 111 (-), 664 (-), 859 (-), 1219 (-),	565 (+), 1071 (+), 1221 (+), 1449 (+), 598 (-), 718 (-),	225 (+), 413 (+), 107 (-), 1493 (-), 1968 (-),	1006 (-), 223 (-), 424 (-), 1252 (-), 1327 (-),	1039 (+), 1274 (+), 1496 (-), 389 (-), 856 (-),	1111 (+), 1126 (+), 1628 (+), 1780 (+), 1438 (-),	1089 (+), 1987 (-), 1835 (-),	1130 (+), 267 (-),
**WRKY71OS**	189 (+), 335 (+), 925 (+), 1081 (+), 1114 (+), 1129 (+), 1309 (+), 1924 (+), 1454 (-), 1626 (-), 1681 (-), 1900 (-), 1900 (-), 1900 (-), 1900 (-), 1900 (-), 1900 (-),	21 (+), 188 (+), 336 (+), 686 (+), 900 (+), 1687 (+), 68 (-), 506 (-), 677 (-), 1064 (-), 1671 (-), 1671 (-), 1671 (-), 1671 (-), 1671 (-), 1671 (-),	71 (+), 89 (+), 352 (+), 820 (+), 1777 (+), 78 (-), 348 (-), 483 (-), 576 (-), 714 (-), 834 (-), 918 (-), 959 (-), 1558 (-), 1985 (-), 1985 (-), 1985 (-), 1985 (-), 1985 (-),	751 (+), 970 (+), 1022 (+), 1553 (+), 1643 (+), 1723 (+), 99 (-), 403 (-), 500 (-), 586 (-), 891 (-), 1040 (-), 1604 (-), 1604 (-), 1604 (-), 1604 (-), 1604 (-), 1604 (-),	369 (+), 460 (+), 494 (+), 503 (+), 1303 (+), 1526 (+), 1610 (+), 35 (-), 256 (-), 511 (-), 527 (-), 1270 (-), 1550 (-), 1592 (-), 1678 (-), 1678 (-), 1678 (-), 1678 (-), 1678 (-), 1678 (-),	249 (+), 1804 (+), 1829 (+), 1928 (+), 407 (-), 654 (-), 1393 (-), 1535 (-), 1910 (-), 1910 (-), 1910 (-), 1910 (-), 1910 (-), 1910 (-),	79 (+), 248 (+), 954 (+), 990 (+), 1391 (+), 1416 (+), 1558 (+), 1817 (+), 1301 (-), 1323 (-), 1667 (-), 1987 (-), 1987 (-), 1987 (-), 1987 (-), 1987 (-), 1987 (-),	164 (+), 552 (+), 325 (-), 677 (-), 1474 (-),	311 (+), 356 (+), 503 (+), 767 (+), 921 (+), 1201 (+), 1210 (+), 1245 (+), 1728 (+), 1738 (+), 1941 (+), 1953 (+), 540 (-), 571 (-), 1387 (-), 1608 (-), 1909 (-), 1909 (-), 1909	326 (+), 337 (+), 652 (+), 989 (+), 1130 (+), 1733 (+), 564 (-), 633 (-), 671 (-), 1541 (-), 1839 (-), 1839 (-), 1839 (-), 1839 (-), 1839 (-), 1839 (-),	1282 (+), 1643 (+), 1945 (+), 51 (-), 112 (-), 154 (-), 167 (-), 665 (-), 679 (-), 860 (-), 1220 (-), 1372 (-), 1512 (-), 1550 (-), 1909 (-), 1976 (-), 1976 (-), 1976 (-), 1976	220 (+), 565 (+), 1071 (+), 1221 (+), 1289 (+), 1449 (+), 1469 (+), 94 (-), 253 (-), 599 (-), 719 (-), 719 (-), 719 (-), 719 (-), 719 (-), 719 (-),	225 (+), 413 (+), 1704 (+), 108 (-), 140 (-), 172 (-), 267 (-), 1494 (-), 1892 (-), 1969 (-),	843 (+), 991 (+), 91 (-), 224 (-), 425 (-), 476 (-), 584 (-), 794 (-), 1007 (-), 1253 (-), 1288 (-), 1328 (-),	1039 (+), 1274 (+), 1556 (+), 1573 (+), 390 (-), 398 (-), 857 (-), 1497 (-), 1497 (-), 1497 (-), 1497 (-), 1497 (-), 1497 (-),	545 (+), 556 (+), 631 (+), 732 (+), 1111 (+), 1126 (+), 1147 (+), 1628 (+), 1780 (+), 919 (-), 1439 (-), 1979 (-), 1990 (-), 1990 (-), 1990 (-), 1990 (-), 1990 (-), 1990 (-),	17 (+), 573 (+), 609 (+), 1074 (+), 1089 (+), 1110 (+), 1558 (+), 1626 (+), 1637 (+), 95 (-), 146 (-), 1836 (-), 1988 (-), 1988 (-), 1988 (-), 1988 (-), 1988 (-), 1988	132 (+), 283 (+), 730 (+), 909 (+), 1130 (+), 1212 (+), 1781 (+), 268 (-), 415 (-),
**WUSATAg**	-	-	-	-	-	-	-	1291 (+), 1291 (+), 1291 (+), 1291 (+),	-	-	-	-	697 (-), 697 (-), 697 (-), 697 (-), 697 (-),	-	-	-	-	1154 (+), 1154 (+), 1154 (+), 1154 (+),
**XYLAT**	-	-	-	-	-	-	-	-	-	-	-	-	-	-	-	-	-	-

Not Present

Supplementary Table 5. The Ka/Ks ratios and estimated divergence time for orthologous SiHSP proteins between foxtail millet, sorghum, maize, rice and *Brachypodium*.

FOXTAIL MILLET - SORGHUM													
**HSP ID**	**Chr.**	**Start**	**End**	**E value**	**Sorghum ID**	**Sorghum chr.**	**Start**	**End**	**% identity**	**Ks**	**Ka**	**Ka/Ks**	**Mya**
SiHSP100-01	1	5798068	5804551	0	Sobic.004G066500	4	5430379	5436780	97	0.26	0.05	0.19	20
SiHSP100-02	1	12407911	12415684	0	Sobic.004G131000	4	17668284	17679789	92	0.26	0.05	0.19	20
SiHSP100-04	1	25559590	25564759	0	Sobic.004G162400	4	50454184	50459051	90	0.21	0.04	0.19	20.8
SiHSP100-07	1	28005200	28011497	0	Sobic.004G183800	4	53005023	53011870	89	0.21	0.04	0.19	16.2
SiHSP100-08	3	5977800	5984329	0	Sobic.008G081900	8	14552810	14559299	99	0.25	0.07	0.28	20.8
SiHSP100-09	3	12275929	12279682	0	Sobic.009G210000	9	55650427	55654408	86	0.24	0.06	0.25	18.5
SiHSP100-10	3	13016068	13019748	0	Sobic.009G201500	9	55111029	55114832	98	0.26	0.06	0.23	20
SiHSP100-11	5	36658683	36662358	0	Sobic.003G293500	3	62620541	62624374	92	0.21	0.04	0.19	16.2
SiHSP100-14	7	18353032	18360053	0	Sobic.006G058100	6	40392554	40400023	98	0.22	0.04	0.18	16.9
SiHSP100-15	7	18363047	18368674	0	Sobic.006G058100	6	40392554	40400023	96	0.21	0.04	0.19	16.2
SiHSP100-16	7	19133453	19138842	0	Sobic.006G065100	6	42444632	42451255	93	0.25	0.07	0.28	19.2
SiHSP100-17	7	19676298	19679535	0	Sobic.006G074900	6	44663537	44667963	81	0.24	0.06	0.25	18.5
SiHSP100-20	9	42170787	42176938	0	Sobic.001G333500	1	55034049	55041488	94	0.26	0.06	0.23	20
SiHSP60-01	1	179729	184974	0	Sobic.004G002200	4	184552	189149	92	0.21	0.04	0.19	16.2
SiHSP60-02	1	7299768	7304886	0	Sobic.004G047200	4	3859080	3864307	98	0.23	0.04	0.17	17.7
SiHSP60-03	2	589555	594286	0	Sobic.002G014400	2	1274739	1280448	98	0.25	0.06	0.24	19.2
SiHSP60-04	2	14956816	14960659	0	Sobic.002G131000	2	18578655	18583228	96	0.23	0.06	0.26	17.7
SiHSP60-05	3	2403233	2409946	0	Sobic.002G423500	2	77029138	77035081	97	0.21	0.05	0.24	16.2
SiHSP60-06	3	11971877	11977268	0	Sobic.009G214100	9	55933261	55939565	93	0.21	0.04	0.19	20.8
SiHSP60-07	3	26620668	26625685	0	Sobic.009G098100	9	36116646	36121629	97	0.25	0.07	0.28	20.8
SiHSP60-08	4	699908	704765	0	Sobic.010G011200	10	976735	981963	98	0.24	0.06	0.25	18.5
SiHSP60-09	4	3925434	3931187	0	Sobic.004G326600	4	65463764	65470595	97	0.26	0.06	0.23	20
SiHSP60-10	4	27834664	27840602	0	Sobic.001G460500	1	66406534	66413074	98	0.26	0.05	0.19	20
SiHSP60-11	4	36307046	36312007	0	Sobic.010G169300	10	49733940	49738634	98	0.26	0.05	0.19	20
SiHSP60-12	4	37441166	37446206	0	Sobic.004G047200	4	3859080	3864307	96	0.21	0.05	0.24	16.2
SiHSP60-13	5	5416922	5421635	0	Sobic.003G112800	3	10146037	10150691	97	0.25	0.06	0.24	19.2
SiHSP60-14	7	21074009	21077334	0	Sobic.001G437900	1	64432239	64436570	92	0.24	0.05	0.21	18.5

SiHSP60-15	7	26596337	26601494	0	Sobic.004G326600	4	65463764	65470595	98	0.26	0.05	0.19	20
SiHSP60-16	8	30233760	30238633	0	Sobic.005G163700	5	54313383	54318528	97	0.21	0.06	0.29	16.2
SiHSP60-17	9	187201	190487	0	Sobic.001G003100	1	278105	282107	97	0.23	0.04	0.17	17.7
SiHSP60-18	9	17442926	17447951	0	Sobic.001G228200	1	21846065	21852784	97	0.24	0.04	0.17	18.5
SiHSP60-19	9	56420402	56425609	0	Sobic.001G228200	1	21846065	21852784	90	0.25	0.04	0.16	19.2
SiHSP60-20	9	58126455	58130544	0	Sobic.001G530800	1	72264916	72269622	96	0.22	0.06	0.27	20.8
SiHSP70-01	1	10246401	10250296	0	Sobic.004G011700	4	981250	985579	97	0.24	0.07	0.29	18.5
SiHSP70-02	1	36152254	36158358	0	Sobic.004G263500	4	60153717	60160279	89	0.24	0.07	0.29	18.5
SiHSP70-04	2	32025020	32027826	0	Sobic.001G193500	1	17198814	17203217	95	0.23	0.06	0.26	17.7
SiHSP70-05	2	35804609	35808035	0	Sobic.002G249800	2	63646363	63649703	96	0.25	0.04	0.16	19.2
SiHSP70-08	3	5573789	5578979	0	Sobic.008G088200	8	18700694	18705774	97	0.21	0.06	0.29	20.8
SiHSP70-09	3	6988813	6994353	0	Sobic.009G066900	9	7341192	7348274	94	0.23	0.04	0.17	17.7
SiHSP70-10	3	7000063	7005788	0	Sobic.009G067000	9	7374652	7381061	89	0.24	0.04	0.17	18.5
SiHSP70-11	3	17574222	17577356	0	Sobic.009G163900	9	52057973	52061547	99	0.25	0.04	0.16	19.2
SiHSP70-12	3	21467161	21473122	0	Sobic.001G129000	1	10115167	10123384	98	0.22	0.06	0.27	16.9
SiHSP70-13	3	41234666	41238851	0	Sobic.008G136000	8	49034254	49038609	99	0.24	0.07	0.29	18.5
SiHSP70-15	4	37951108	37956799	0	Sobic.010G230600	10	57097171	57104031	90	0.24	0.07	0.29	18.5
SiHSP70-16	5	23768496	23770499	0	Sobic.003G378700	3	69271261	69273529	85	0.23	0.06	0.26	17.7
SiHSP70-17	5	23871912	23873915	0	Sobic.003G378700	3	69271261	69273529	85	0.25	0.04	0.16	19.2
SiHSP70-18	5	41050516	41053751	0	Sobic.003G350700	3	67035595	67038868	99	0.21	0.04	0.19	20.8
SiHSP70-22	9	1843820	1847776	0	Sobic.008G136000	8	49034254	49038609	94	0.25	0.07	0.28	19.2
SiHSP70-23	9	7468266	7470511	0	Sobic.001G118600	1	9228011	9230460	93	0.24	0.06	0.25	18.5
SiHSP70-24	9	13463310	13467080	0	Sobic.001G193500	1	17198814	17203217	95	0.27	0.09	0.33	20.8
SiHSP70-25	9	49893761	49896547	0	Sobic.001G418600	1	62741658	62744909	98	0.21	0.06	0.29	16.2
SiHSP70-26	9	49939979	49942366	0	Sobic.001G420100	1	62869072	62871695	99	0.21	0.06	0.29	16.2
SiHSP70-27	9	52763610	52767974	0	Sobic.001G454000	1	65861178	65864823	90	0.23	0.04	0.17	17.7
SiHSP90-01	2	6007915	6010199	0	Sobic.007G224100	7	63928689	63933642	86	0.24	0.04	0.17	18.5
SiHSP90-02	2	34600248	34605291	0	Sobic.007G224100	7	63928689	63933642	89	0.25	0.04	0.16	19.2
SiHSP90-03	3	45081885	45088781	0	Sobic.008G111600	8	44284585	44292434	90	0.22	0.06	0.27	16.9
SiHSP90-04	4	39628388	39633588	0	Sobic.010G267400	10	60059350	60064968	96	0.24	0.07	0.29	18.5
SiHSP90-05	6	31466879	31471644	0	Sobic.007G224100	7	63928689	63933642	96	0.24	0.07	0.29	18.5

SiHSP90-06	6	32063209	32065743	0	Sobic.007G216300	7	63233778	63237824	98	0.23	0.06	0.26	17.7
SiHSP90-07	6	32076788	32080613	0	Sobic.007G216300	7	63233778	63237824	98	0.25	0.04	0.16	19.2
SiHSP90-08	6	32083265	32087190	0	Sobic.007G216300	7	63233778	63237824	98	0.22	0.04	0.18	16.9
SiHSP90-09	7	1556544	1559316	0	Sobic.006G005600	6	837870	840874	96	0.22	0.04	0.18	16.9
SisHSP-01	1	3123887	3125282	5.70E-85	Sobic.004G092800	4	7921132	7922072	86	0.21	0.05	0.24	20.8
SisHSP-02	1	4474502	4475416	6.80E-71	Sobic.004G083600	4	6917870	6919168	80	0.25	0.06	0.24	20.8
SisHSP-05	1	38572788	38573986	2.10E-111	Sobic.004G228900	4	57154672	57155971	86	0.24	0.05	0.21	18.5
SisHSP-06	1	39834413	39835503	1.20E-79	Sobic.004G321000	4	64875360	64876595	91	0.26	0.05	0.19	20
SisHSP-08	3	2125364	2127024	1.60E-44	Sobic.008G178900	8	53934795	53936714	80	0.26	0.05	0.19	20
SisHSP-10	3	14350334	14352367	2.80E-93	Sobic.009G187400	9	54001560	54003967	88	0.22	0.07	0.32	20.8
SisHSP-14	4	10433864	10434817	2.80E-55	Sobic.010G104300	10	9756647	9757919	82	0.21	0.06	0.29	20.8
SisHSP-15	5	7897990	7898792	2.00E-60	Sobic.003G082500	3	7081950	7082402	86	0.23	0.06	0.26	17.7
SisHSP-17	5	7909852	7910635	1.60E-46	Sobic.003G082000	3	7044736	7045581	88	0.25	0.04	0.16	19.2
SisHSP-18	5	7912355	7913513	1.30E-57	Sobic.003G082100	3	7048518	7049369	92	0.22	0.04	0.18	16.9
SisHSP-19	5	7923272	7924004	1.20E-60	Sobic.003G082000	3	7044736	7045581	92	0.25	0.06	0.24	19.2
SisHSP-20	5	7924595	7925730	5.60E-91	Sobic.003G082000	3	7044736	7045581	92	0.24	0.05	0.21	18.5
SisHSP-21	5	11220808	11221302	9.10E-65	Sobic.003G039400	3	3696466	3697630	100	0.23	0.06	0.26	17.7
SisHSP-22	5	11223563	11224171	3.40E-69	Sobic.003G039400	3	3696466	3697630	89	0.25	0.04	0.16	19.2
SisHSP-24	5	31740859	31741583	2.30E-64	Sobic.003G201200	3	53040468	53041626	86	0.22	0.04	0.18	16.9
SisHSP-26	7	21161295	21162303	1.80E-94	Sobic.006G093500	6	47249049	47250400	88	0.22	0.04	0.18	16.9
SisHSP-27	8	8605725	8606738	6.70E-65	Sobic.005G086400	5	12040101	12041114	81	0.21	0.05	0.24	16.2
SisHSP-34	9	50454063	50454638	2.80E-67	Sobic.001G426000	1	63385616	63387051	96	0.25	0.06	0.24	19.2
SisHSP-35	9	50484374	50485172	3.10E-66	Sobic.001G425600	1	63355114	63356147	93	0.24	0.05	0.21	18.5
SisHSP-36	9	50485555	50487142	4.40E-66	Sobic.001G425500	1	63353980	63354978	90	0.23	0.06	0.26	20.8
SisHSP-37	9	51325204	51326419	4.10E-130	Sobic.001G438000	1	64436899	64438301	89	0.25	0.04	0.16	19.2
									**MEAN**	**0.2**	**0.1**	**0.2**	**18.6**

**FOXTAIL MILLET - MAIZE**													
**HSP ID**	**Chr.**	**Start**	**End**	**E value**	**Sorghum ID**	**Maize chr.**	**Start**	**End**	**% identity**	**Ks**	**Ka**	**Ka/Ks**	**Mya**
SiHSP100-01	1	5798068	5804551	0	GRMZM2G060561	5	93230538	93236600	96	0.24	0.09	0.38	18.5
SiHSP100-02	1	12407911	12415684	0	GRMZM5G861603	5	1.3E+08	130122744	90	0.25	0.07	0.28	19.2

SiHSP100-04	1	25559590	25564759	0	GRMZM2G172230	5	167938142	167942807	91	0.26	0.07	0.27	20
SiHSP100-07	1	28005200	28011497	0.00E+00	GRMZM2G101791	5	174670519	174677666	91	0.26	0.09	0.35	20
SiHSP100-08	3	5977800	5984329	0	GRMZM2G009443	10	35583589	35590383	98	0.26	0.08	0.31	20
SiHSP100-10	3	13016068	13019748	0	GRMZM2G360681	6	160621048	160624717	97	0.29	0.09	0.31	22.3
SiHSP100-11	5	36658683	36662358	0.00E+00	GRMZM2G360681	6	160621048	160624717	90	0.28	0.07	0.25	21.5
SiHSP100-14	7	18353032	18360053	0	GRMZM2G123922	10	111239151	111246665	98	0.27	0.09	0.33	20.8
SiHSP100-15	7	18363047	18368674	0.00E+00	GRMZM2G123922	10	111239151	111246665	96	0.27	0.08	0.3	20.8
SiHSP100-16	7	19133453	19138842	0	GRMZM2G149567	2	59795473	59802602	92	0.25	0.08	0.32	19.2
SiHSP100-17	7	19676298	19679535	0.00E+00	GRMZM2G049681	10	114852674	114856305	82	0.24	0.08	0.33	18.5
SiHSP100-20	9	42170787	42176938	0	GRMZM2G162968	9	124034244	124040497	94	0.24	0.09	0.38	18.5
SiHSP60-01	1	179729	184974	0	GRMZM2G015989	4	2.41E+08	2.41E+08	91	0.26	0.07	0.27	22
SiHSP60-02	1	7299768	7304886	0	GRMZM2G009871	4	2.37E+08	2.37E+08	98	0.26	0.07	0.27	22
SiHSP60-03	2	589555	594286	0	GRMZM2G043383	7	2584958	2590700	97	0.28	0.07	0.25	21.5
SiHSP60-04	2	14956816	14960659	0	GRMZM2G321767	2	1.66E+08	1.66E+08	95	0.29	0.06	0.21	22.3
SiHSP60-05	3	2403233	2409946	0	GRMZM2G109425	2	2.18E+08	2.18E+08	96	0.24	0.08	0.33	18.5
SiHSP60-06	3	11971877	11977268	0	GRMZM2G074790	6	161725359	1.62E+08	92	0.24	0.09	0.38	18.5
SiHSP60-07	3	26620668	26625685	0	AC215201.3_FG005	6	1.42E+08	142086400	92	0.25	0.07	0.28	19.2
SiHSP60-08	4	699908	704765	0	GRMZM2G083716	9	25922353	25927460	98	0.26	0.07	0.27	20
SiHSP60-09	4	3925434	3931187	0	GRMZM2G110626	5	213171515	2.13E+08	97	0.24	0.09	0.38	18.5
SiHSP60-10	4	27834664	27840602	0	GRMZM2G175510	4	2.29E+08	228825532	98	0.24	0.09	0.38	18.5
SiHSP60-11	4	36307046	36312007	0	GRMZM2G070542	2	2.36E+08	2.36E+08	97	0.29	0.09	0.31	22.3
SiHSP60-12	4	37441166	37446206	0	GRMZM2G009871	4	2.37E+08	2.37E+08	96	0.28	0.07	0.25	21.5
SiHSP60-13	5	5416922	5421635	0	GRMZM2G058276	8	6280491	6291108	95	0.27	0.09	0.33	20.8
SiHSP60-14	7	21074009	21077334	0	GRMZM2G042253	1	33470702	33474591	92	0.27	0.08	0.3	20.8
SiHSP60-15	7	26596337	26601494	0	GRMZM2G110626	5	213171515	2.13E+08	97	0.24	0.08	0.33	18.5
SiHSP60-16	8	30233760	30238633	0	GRMZM2G381744	4	2.02E+08	2.02E+08	96	0.26	0.09	0.35	20
SiHSP60-17	9	187201	190487	0	GRMZM2G434173	1	3E+08	3E+08	95	0.26	0.08	0.31	20
SiHSP60-18	9	17442926	17447951	0	GRMZM2G416120	1	2.35E+08	2.35E+08	97	0.27	0.08	0.3	20.8
SiHSP60-19	9	56420402	56425609	0	GRMZM2G416120	1	2.35E+08	2.35E+08	89	0.24	0.09	0.38	24
SiHSP60-20	9	58126455	58130544	0	GRMZM2G085909	1	3830541	3834833	96	0.24	0.08	0.33	24
SiHSP70-01	1	10246401	10250296	0	GRMZM2G415007	4	2.4E+08	2.4E+08	97	0.26	0.07	0.27	20

SiHSP70-02	1	36152254	36158358	0	GRMZM2G020040	5	2.02E+08	2.02E+08	89	0.26	0.07	0.27	20
SiHSP70-04	2	32025020	32027826	0	GRMZM2G153815	5	21697782	21703415	94	0.26	0.07	0.27	20
SiHSP70-05	2	35804609	35808035	0	GRMZM2G365374	2	190158113	190161171	95	0.28	0.07	0.25	21.5
SiHSP70-08	3	5573789	5578979	0	GRMZM2G001500	10	21722658	21727770	96	0.29	0.06	0.21	22.3
SiHSP70-09	3	6988813	6994353	0	GRMZM2G063676	8	90052293	90060254	91	0.24	0.08	0.33	18.5
SiHSP70-10	3	7000063	7005788	0	GRMZM2G023232	6	130343970	130350095	87	0.24	0.09	0.38	18.5
SiHSP70-11	3	17574222	17577356	0	GRMZM5G802801	8	116617793	116620719	98	0.25	0.07	0.28	19.2
SiHSP70-12	3	21467161	21473122	0	GRMZM2G111475	5	12580030	12585678	98	0.26	0.07	0.27	20
SiHSP70-13	3	41234666	41238851	0	GRMZM2G340251	3	126460606	126465045	99	0.28	0.07	0.25	21.5
SiHSP70-14	4	4474303	4477299	0	GRMZM2G145275	1	41217232	41221207	81	0.27	0.09	0.33	20.8
SiHSP70-15	4	37951108	37956799	0	GRMZM2G056766	5	41360388	41366915	87	0.29	0.09	0.31	22.3
SiHSP70-17	5	23871912	23873915	0	GRMZM2G415007	4	240050091	240053865	80	0.28	0.07	0.25	21.5
SiHSP70-18	5	41050516	41053751	0	GRMZM2G310431	8	168955391	168958754	98	0.27	0.09	0.33	20.8
SiHSP70-22	9	1843820	1847776	0	GRMZM2G340251	3	126460606	126465045	95	0.27	0.08	0.3	20.8
SiHSP70-23	9	7468266	7470511	0	GRMZM2G471196	5	7356194	7358172	85	0.25	0.08	0.32	19.2
SiHSP70-24	9	13463310	13467080	0	GRMZM2G153815	5	21697782	21703415	98	0.29	0.08	0.28	22.3
SiHSP70-26	9	49939979	49942366	0	GRMZM2G428391	1	41101054	41103817	97	0.24	0.07	0.29	18.5
SiHSP70-27	9	52763610	52767974	0	GRMZM2G024718	9	146456351	146459543	94	0.25	0.07	0.28	19.2
SiHSP90-01	2	6007915	6010199	0	GRMZM5G813217	7	129966717	129971449	88	0.24	0.08	0.33	18.5
SiHSP90-02	2	34600248	34605291	0	GRMZM2G002220	4	197363864	197368446	88	0.26	0.09	0.35	20
SiHSP90-04	4	39628388	39633588	0	GRMZM2G399073	5	61527567	61532764	94	0.28	0.07	0.25	21.5
SiHSP90-05	6	31466879	31471644	0	GRMZM2G002220	4	197363864	197368446	96	0.27	0.09	0.33	20.8
SiHSP90-06	6	32063209	32065743	0	GRMZM2G012631	4	198369976	198374059	97	0.27	0.08	0.3	20.8
SiHSP90-07	6	32076788	32080613	0	GRMZM2G012631	4	198369976	198374059	97	0.25	0.08	0.32	19.2
SiHSP90-08	6	32083265	32087190	0	GRMZM2G012631	4	198369976	198374059	97	0.24	0.07	0.29	24
SiHSP90-09	7	1556544	1559316	0	GRMZM5G833699	10	93684085	93687059	96	0.25	0.07	0.28	19.2
SisHSP-02	1	4474502	4475416	8.80E-71	GRMZM5G803365	5	115589124	1.16E+08	81	0.24	0.08	0.33	24
SisHSP-05	1	38572788	38573986	########	GRMZM2G007729	4	179822113	179823382	86	0.26	0.09	0.35	24
SisHSP-06	1	39834413	39835503	2.20E-77	GRMZM2G098167	5	212248891	212249987	86	0.26	0.08	0.31	24
SisHSP-08	3	2125364	2127024	5.00E-45	GRMZM2G449274	1	176841045	176845475	88	0.27	0.08	0.3	20.8
SisHSP-10	3	14350334	14352367	4.20E-93	GRMZM2G013970	6	156748173	156750220	84	0.24	0.09	0.38	18.5

SisHSP-15	5	7897990	7898792	9.00E-54	GRMZM2G049767	3	20641924	20642867	85	0.28	0.07	0.25	21.5
SisHSP-17	5	7909852	7910635	8.30E-44	GRMZM2G158232	3	20534046	20534889	85	0.27	0.09	0.33	20.8
SisHSP-18	5	7912355	7913513	9.60E-54	GRMZM2G158232	3	20534046	20534889	84	0.27	0.08	0.3	20.8
SisHSP-19	5	7923272	7924004	2.70E-56	GRMZM2G158232	3	20534046	20534889	88	0.25	0.08	0.32	19.2
SisHSP-20	5	7924595	7925730	7.60E-85	GRMZM2G158232	3	20534046	20534889	88	0.28	0.07	0.25	21.5
SisHSP-21	5	11220808	11221302	1.40E-65	GRMZM2G083810	3	8884623	8885815	95	0.29	0.06	0.21	22.3
SisHSP-22	5	11223563	11224171	6.40E-70	GRMZM2G083810	3	8884623	8885815	94	0.26	0.09	0.35	22
SisHSP-26	7	21161295	21162303	8.50E-87	GRMZM2G331701	2	45966874	45967865	85	0.26	0.08	0.31	22
SisHSP-27	8	8605725	8606738	1.40E-70	GRMZM2G375517	2	2.2E+08	2.2E+08	83	0.25	0.07	0.28	19.2
SisHSP-34	9	50454063	50454638	3.00E-67	GRMZM2G046382	1	38719939	38720992	96	0.26	0.07	0.27	20
SisHSP-36	9	50485555	50487142	1.00E-62	GRMZM2G046382	1	38719939	38720992	85	0.24	0.09	0.38	18.5
SisHSP-37	9	51325204	51326419	########	GRMZM2G149647	1	33459256	33460463	87	0.24	0.09	0.38	18.5
									**MEAN**	**0.3**	**0.1**	**0.3**	**20.5**

**FOXTAIL MILLET - RICE**													
**HSP ID**	**Chr.**	**Start**	**End**	**E value**	**Sorghum ID**	**Rice chr.**	**Start**	**End**	**% identity**	**Ks**	**Ka**	**Ka/Ks**	**Mya**
SiHSP100-01	1	5798068	5804551	0	LOC_Os02g08490	2	4567617	4573049	92	0.45	0.26	0.58	34.6
SiHSP100-02	1	12407911	12415684	0	LOC_Os02g19150	2	11162263	11171493	85	0.44	0.26	0.59	33.8
SiHSP100-07	1	28005200	28011497	0	LOC_Os02g35630	2	21425656	21431714	86	0.46	0.3	0.65	35.4
SiHSP100-08	3	5977800	5984329	0	LOC_Os12g12850	12	7095797	7101612	95	0.46	0.28	0.61	35.4
SiHSP100-10	3	13016068	13019748	0	LOC_Os05g44340	5	25803530	25807160	96	0.45	0.3	0.67	34.6
SiHSP100-11	5	36658683	36662358	0	LOC_Os05g44340	5	25803530	25807160	90	0.44	0.26	0.59	33.8
SiHSP100-20	9	42170787	42176938	0.00E+00	LOC_Os03g31300	3	17832202	17839322	96	0.43	0.29	0.67	33.1
SiHSP60-01	1	179729	184974	0	LOC_Os02g01280	2	158662	163732	91	0.45	0.26	0.58	34.6
SiHSP60-02	1	7299768	7304886	0	LOC_Os06g47320	6	28682052	28687078	96	0.46	0.26	0.57	35.4
SiHSP60-03	2	589555	594286	0	LOC_Os06g36700	6	21598229	21602687	95	0.47	0.27	0.57	36.2
SiHSP60-04	2	14956816	14960659	0	LOC_Os09g38980	9	22382924	22387028	93	0.44	0.3	0.68	33.8
SiHSP60-05	3	2403233	2409946	0	LOC_Os05g05470	5	2718697	2723446	96	0.46	0.26	0.57	35.4
SiHSP60-06	3	11971877	11977268	0	LOC_Os05g46290	5	26835671	26840314	90	0.45	0.26	0.58	34.6
SiHSP60-07	3	26620668	26625685	0	LOC_Os12g17910	12	10293567	10297912	95	0.47	0.3	0.64	36.2
SiHSP60-08	4	699908	704765	0	LOC_Os06g02380	6	811034	816534	93	0.44	0.28	0.64	33.8

SiHSP60-09	4	3925434	3931187	0	LOC_Os04g46620	4	27638052	27643893	97	0.46	0.29	0.63	35.4
SiHSP60-10	4	27834664	27840602	0	LOC_Os06g34690	6	20158641	20165624	96	0.47	0.3	0.64	36.2
SiHSP60-11	4	36307046	36312007	0	LOC_Os06g36700	6	21598229	21602687	96	0.43	0.28	0.65	33.1
SiHSP60-12	4	37441166	37446206	0	LOC_Os06g47320	6	28682052	28687078	95	0.49	0.27	0.55	37.7
SiHSP60-13	5	5416922	5421635	0	LOC_Os03g42220	3	23485491	23490741	95	0.44	0.25	0.57	33.8
SiHSP60-14	7	21074009	21077334	0	ChrSy.fgenesh.gene.28	ChrSy	179075	182794	84	0.52	0.3	0.58	40
SiHSP60-15	7	26596337	26601494	0	LOC_Os04g46620	4	27638052	27643893	97	0.49	0.28	0.57	37.7
SiHSP60-16	8	30233760	30238633	0	LOC_Os03g59020	3	33592752	33598074	95	0.43	0.28	0.65	33.1
SiHSP60-17	9	187201	190487	0	LOC_Os03g64210	3	36281082	36284945	90	0.5	0.29	0.58	38.5
SiHSP60-18	9	17442926	17447951	0	LOC_Os10g32550	10	17048041	17053445	95	0.5	0.28	0.56	38.5
SiHSP60-19	9	56420402	56425609	0	LOC_Os10g32550	10	17048041	17053445	90	0.44	0.28	0.64	33.8
SiHSP60-20	9	58126455	58130544	0	LOC_Os02g22780	2	13560753	13565942	95	0.49	0.28	0.57	37.7
SiHSP70-01	1	10246401	10250296	0	LOC_Os02g02410	2	838717	842672	97	0.43	0.27	0.63	33.1
SiHSP70-02	1	36152254	36158358	0	LOC_Os02g48110	2	29451939	29457868	86	0.5	0.28	0.56	38.5
SiHSP70-04	2	32025020	32027826	0	LOC_Os03g02260	3	766648	771264	91	0.49	0.26	0.53	37.7
SiHSP70-05	2	35804609	35808035	0	LOC_Os09g31486	9	18987942	18991156	92	0.44	0.26	0.59	33.8
SiHSP70-08	3	5573789	5578979	0	LOC_Os12g14070	12	7986161	7991953	90	0.5	0.3	0.6	38.5
SiHSP70-09	3	6988813	6994353	0	LOC_Os01g08560	1	4253838	4259626	82	0.45	0.26	0.58	34.6
SiHSP70-10	3	7000063	7005788	0	LOC_Os01g08560	1	4253838	4259626	83	0.49	0.3	0.61	37.7
SiHSP70-11	3	17574222	17577356	0	LOC_Os05g38530	5	22599355	22602579	97	0.5	0.26	0.52	38.5
SiHSP70-12	3	21467161	21473122	0	LOC_Os05g23740	5	13621777	13627168	96	0.44	0.26	0.59	33.8
SiHSP70-13	3	41234666	41238851	0	LOC_Os11g47760	11	28804248	28808550	96	0.54	0.26	0.48	41.5
SiHSP70-15	4	37951108	37956799	0	LOC_Os06g46600	6	28286534	28293449	81	0.54	0.3	0.56	41.5
SiHSP70-16	5	23768496	23770499	0	LOC_Os05g35400	5	21033769	21036041	85	0.44	0.31	0.7	33.8
SiHSP70-17	5	23871912	23873915	0	LOC_Os05g35400	5	21033769	21036041	85	0.54	0.26	0.48	41.5
SiHSP70-18	5	41050516	41053751	0	LOC_Os01g62290	1	36039706	36043510	97	0.54	0.31	0.57	41.5
SiHSP70-22	9	1843820	1847776	0	LOC_Os03g60620	3	34446323	34450944	98	0.44	0.27	0.61	33.8
SiHSP70-23	9	7468266	7470511	0	LOC_Os03g50250	3	28647086	28649570	87	0.54	0.31	0.57	41.5
SiHSP70-24	9	13463310	13467080	0	LOC_Os02g53420	2	32688605	32693044	91	0.49	0.31	0.63	37.7
SiHSP70-25	9	49893761	49896547	0	LOC_Os03g16920	3	9411494	9416082	96	0.54	0.3	0.56	41.5
SiHSP70-26	9	49939979	49942366	0	LOC_Os03g16860	3	9370159	9373083	95	0.43	0.31	0.72	33.1

SiHSP90-01	2	6007915	6010199	0	LOC_Os09g29840	9	18150618	18155512	87	0.57	0.29	0.51	43.8
SiHSP90-02	2	34600248	34605291	0	LOC_Os09g29840	9	18150618	18155512	91	0.43	0.3	0.7	33.1
SiHSP90-03	3	45081885	45088781	0	LOC_Os12g32986	12	19921576	19927766	83	0.46	0.31	0.67	35.4
SiHSP90-04	4	39628388	39633588	0	LOC_Os06g50300	6	30444411	30450497	91	0.57	0.25	0.44	43.8
SiHSP90-05	6	31466879	31471644	0	LOC_Os08g38086	8	24124838	24129488	89	0.46	0.27	0.59	35.4
SiHSP90-06	6	32063209	32065743	0	LOC_Os09g30412	9	18514572	18518316	94	0.57	0.24	0.42	43.8
SiHSP90-07	6	32076788	32080613	0	LOC_Os09g30412	9	18514572	18518316	93	0.49	0.27	0.55	37.7
SiHSP90-08	6	32083265	32087190	0	LOC_Os09g30412	9	18514572	18518316	93	0.57	0.26	0.46	43.8
SiHSP90-09	7	1556544	1559316	0	LOC_Os04g01740	4	483241	486065	89	0.57	0.28	0.49	43.8
SisHSP-16	Si5	7908007	7908850	3.40E-58	LOC_Os01g04350	Os1	1941003	1941953	81	0.45	0.29	0.64	34.6
SisHSP-17	Si5	7909852	7910635	3.30E-44	LOC_Os01g04370	Os1	1948737	1949587	90	0.57	0.27	0.47	43.8
SisHSP-18	Si5	7912355	7913513	2.30E-55	LOC_Os01g04360	Os1	1944247	1945035	91	0.46	0.27	0.59	35.4
SisHSP-19	Si5	7923272	7924004	2.50E-56	LOC_Os01g04380	Os1	1951928	1952771	89	0.57	0.24	0.42	43.8
SisHSP-20	Si5	7924595	7925730	6.80E-86	LOC_Os01g04370	Os1	1948737	1949587	89	0.57	0.26	0.46	43.8
SisHSP-28	Si9	9816080	9819828	1.00E-31	LOC_Os03g45340	Os3	25599553	25600862	88	0.43	0.26	0.6	33.1
SisHSP-34	Si9	50454063	50454638	1.50E-64	LOC_Os03g15960	Os3	8805567	8806601	89	0.55	0.26	0.47	42.3
SisHSP-35	Si9	50484374	50485172	2.30E-69	LOC_Os03g15960	Os3	8805567	8806601	86	0.55	0.3	0.55	42.3
SisHSP-36	Si9	50485555	50487142	4.20E-65	LOC_Os03g16030	Os3	8834823	8835802	88	0.44	0.25	0.57	33.8
SisHSP-37	Si9	51325204	51326419	########	LOC_Os03g14180	Os3	7697015	7698284	84	0.55	0.27	0.49	42.3
									**MEAN**	**0.5**	**0.3**	**0.6**	**37.3**

**FOXTAIL MILLET - BRACHYPODIUM**										
**HSP ID**	**E value**	**Brachypodium ID**	**Brachypodium chromosome**	**Start**	**End**	**% identity**	**Ks**	**Ka**	**Ka/Ks**	**Mya**
SiHSP100-01	0	Bradi3g06107	3	4398051	4404080	91	0.26	0.63	0.4	45
SiHSP100-02	0	Bradi3g10660	3	8850843	8859522	84	0.34	0.72	0.5	51.4
SiHSP100-04	0	Bradi3g44640	3	46629458	46634463	86	0.26	0.83	0.3	59.3
SiHSP100-07	0	Bradi3g46170	3	48104359	48110592	88	0.31	0.87	0.4	62.1
SiHSP100-08	0	Bradi4g39880	4	44447338	44453462	94	0.28	0.71	0.4	50.7
SiHSP100-10	0	Bradi2g19540	2	17209321	17212448	95	0.26	0.83	0.3	59.3
SiHSP100-11	0	Bradi2g49660	2	49696910	49700438	92	0.26	0.83	0.3	59.3
SiHSP100-14	0	Bradi3g44340	3	46140435	46158800	95	0.31	0.87	0.4	62.1
SiHSP100-15	0	Bradi3g44340	3	46140435	46158800	99	0.26	0.83	0.3	59.3

SiHSP100-16	0	Bradi5g08920	5	12044633	12050329	86	0.34	0.72	0.5	51.4
SiHSP100-20	0	Bradi1g16190	1	13106112	13113550	92	0.26	0.83	0.3	59.3
SiHSP70-01	0	Bradi3g01477	3	930882	934983	96	0.31	0.87	0.4	62.1
SiHSP70-02	0	Bradi3g53100	3	53871677	53878144	84	0.28	0.71	0.4	50.7
SiHSP70-04	0	Bradi1g77637	1	73885217	73889364	90	0.26	0.83	0.3	59.3
SiHSP70-05	0	Bradi4g33878	4	39556892	39559789	91	0.26	0.83	0.3	59.3
SiHSP70-08	0	Bradi4g39470	4	44074559	44080339	89	0.31	0.87	0.4	62.1
SiHSP70-09	0	Bradi2g33682	2	33814330	33819102	83	0.26	0.83	0.3	59.3
SiHSP70-10	0	Bradi2g33682	2	33814330	33819102	86	0.31	0.87	0.4	62.1
SiHSP70-11	0	Bradi2g23250	2	20789638	20793184	96	0.26	0.83	0.3	59.3
SiHSP70-12	0	Bradi2g30560	2	30223015	30229068	94	0.34	0.72	0.5	51.4
SiHSP70-13	0	Bradi4g04220	4	3417110	3421010	95	0.26	0.83	0.3	59.3
SiHSP70-15	0	Bradi1g32770	1	28176647	28182076	83	0.31	0.87	0.4	62.1
SiHSP70-18	0	Bradi2g23250	2	20789638	20793184	96	0.28	0.71	0.4	50.7
SiHSP70-22	0	Bradi1g03720	1	2511355	2515521	96	0.26	0.83	0.3	59.3
SiHSP70-24	0	Bradi1g77637	1	73885217	73889364	89	0.28	0.71	0.4	50.7
SiHSP70-25	0	Bradi1g66470	1	65253599	65256102	93	0.26	0.83	0.3	59.3
SiHSP70-26	0	Bradi1g66590	1	65332123	65335039	94	0.26	0.83	0.3	59.3
SiHSP70-27	0	Bradi1g69700	1	68185327	68188756	89	0.31	0.87	0.4	62.1
SiHSP90-01	0	Bradi4g32941	4	38737602	38742043	86	0.26	0.83	0.3	59.3
SiHSP90-02	0	Bradi4g32941	4	38737602	38742043	88	0.31	0.87	0.4	62.1
SiHSP90-03	0	Bradi4g06370	4	5322450	5328583	84	0.28	0.71	0.4	50.7
SiHSP90-04	0	Bradi1g30130	1	25540745	25546115	92	0.26	0.83	0.3	59.3
SiHSP90-05	0	Bradi3g38897	3	41354946	41359904	91	0.26	0.83	0.3	59.3
SiHSP90-06	0	Bradi3g39590	3	41826150	41830502	91	0.31	0.87	0.4	62.1
SiHSP90-07	0	Bradi3g39590	3	41826150	41830502	92	0.26	0.83	0.3	59.3
SiHSP90-08	0	Bradi3g39590	3	41826150	41830502	92	0.34	0.72	0.5	51.4
SiHSP90-09	0	Bradi5g02037	5	2157500	2160247	92	0.26	0.83	0.3	59.3
SisHSP-17	2.20E-44	Bradi2g02410	2	1626109	1626863	85	0.31	0.87	0.4	62.1
SisHSP-18	2.20E-44	Bradi2g02410	2	1626109	1626863	85	0.28	0.71	0.4	50.7
SisHSP-19	6.20E-51	Bradi2g02410	2	1626109	1626863	81	0.26	0.83	0.3	59.3
SisHSP-20	4.30E-81	Bradi2g02410	2	1626109	1626863	84	0.26	0.83	0.3	59.3
SisHSP-21	6.70E-71	Bradi2g05380	2	3906496	3907279	81	0.31	0.87	0.4	62.1
SisHSP-22	1.20E-74	Bradi2g05380	2	3906496	3907279	81	0.26	0.83	0.3	59.3

SisHSP-34	1.30E-64	Bradi1g67080	1	65802387	65803316	91	0.31	0.87	0.4	62.1
SisHSP-35	4.80E-67	Bradi1g67040	1	65770535	65771305	82	0.26	0.83	0.3	59.3
SisHSP-36	4.20E-66	Bradi1g53850	1	52261638	52262497	85	0.34	0.72	0.5	51.4
SisHSP-37	#######	Bradi1g68440	1	66943477	66944663	81	0.26	0.83	0.3	59.3
						**MEAN**	**0.3**	**0.8**	**0.4**	**57.8**

Supplementary Table 6: Fold expression values of *SiHSP* genes in two contrasting cultivars in response to various stresses.

Tolerant cultivar (IC 4) in response to salinity stress											
**SAMPLE**	**DETECTOR**	**Ct1**	**Ct2**	**Ct3**	**sd**	**Avg Ct**	**End CTl**	**dCt**	**CTL0h**	**ddCt**	**Log Ct**
1hsalt_leaf	ACT	31.12611	30.57903	31.25997	0.322675	30.98837	30.98837	0	0	0	1
1hsalt_leaf_ST	ACT	30.59235	30.50901	30.68559	0.088339	30.59565	30.59565	0	0	0	1
1hsalt_stem	ACT	35.05992	30.50797	32.18615	2.058966	32.58468	32.58468	0	0	0	1
1hsalt_stem_ST	ACT	30.15948	30.83181	30.99775	0.443893	30.66301	30.66301	0	0	0	1
24hsalt_leaf	ACT	27.20658	27.21153	26.93239	0.142886	27.11683	27.11683	0	0	0	1
24hsalt_leaf_ST	ACT	26.74441	26.44323	26.17784	0.283472	26.45516	26.45516	0	0	0	1
24hsalt_stem	ACT	31.16855	32.03036	32.40502	0.567092	31.86798	31.86798	0	0	0	1
24hsalt_stem_ST	ACT	30.47015	30.92189	30.24818	0.343323	30.54674	30.54674	0	0	0	1
6hsalt_leaf	ACT	28.78678	28.71104	28.52616	0.119911	28.67466	28.67466	0	0	0	1
6hsalt_leaf_ST	ACT	27.82815	27.90591	27.46921	0.232951	27.73442	27.73442	0	0	0	1
6hsalt_stem	ACT	30.38717	29.87943	30.89936	0.456126	30.38865	30.38865	0	0	0	1
6hsalt_stem_ST	ACT	30.4885	30.49312	31.5566	0.615336	30.84607	25.88104	4.96503	0	4.96503	0.032017
1hsalt_leaf_ST	HSP100-07	30.87141	30.30106	30.39104	0.306638	30.52117	30.59565	-0.07448	-1.7011	1.626618	0.323846
1hsalt_stem_ST	HSP100-07	31.88505	31.8284	31.43773	0.243561	31.71706	30.66301	1.054048	1.011604	0.042444	0.971009
24hsalt_leaf_ST	HSP100-07	27.15124	26.97812	27.02377	0.089724	27.05104	26.45516	0.59588	-1.7011	2.296981	0.203488
24hsalt_stem_ST	HSP100-07	30.30299	31.70039	31.83141	0.847146	31.27826	30.54674	0.731527	1.011604	-0.28008	1.21426
6hsalt_leaf_ST	HSP100-07	27.59856	27.96668	27.98864	0.219148	27.85129	27.73442	0.116871	-1.7011	1.817972	0.283619
6hsalt_stem_ST	HSP100-07	29.96286	30.51229	30.21961	0.274907	30.23159	25.88104	4.350545	1.011604	3.338942	0.098828
1hsalt_leaf_ST	HSP100-08	26.93098	26.15485	26.97079	0.46002	26.68554	30.59565	-3.91011	-5.52741	1.617301	0.325945
1hsalt_stem_ST	HSP100-08	29.98253	29.19361	28.50124	0.74117	29.2258	30.66301	-1.43722	0.086964	-1.52418	2.876237
24hsalt_leaf_ST	HSP100-08	26.78601	25.70643	25.76537	0.606999	26.08593	26.45516	-0.36923	-5.52741	5.158183	0.028005
24hsalt_stem_ST	HSP100-08	29.91775	29.85362	29.98916	0.067801	29.92018	30.54674	-0.62656	0.086964	-0.71352	1.639804
6hsalt_leaf_ST	HSP100-08	26.8879	26.8054	26.85665	0.041655	26.84998	27.73442	-0.88444	-5.52741	4.64297	0.040025
6hsalt_stem_ST	HSP100-08	28.29796	28.42448	28.40741	0.068653	28.37662	25.88104	2.495578	0.086964	2.408614	0.188337
1hsalt_leaf_ST	HSP100-11	28.08393	28.58584	28.67753	0.31955	28.4491	30.59565	-2.14655	0.939984	-3.08653	8.494531
1hsalt_stem_ST	HSP100-11	31.73524	31.95582	31.37143	0.295108	31.6875	30.66301	1.024482	3.166584	-2.1421	4.414048
24hsalt_leaf_ST	HSP100-11	23.21539	23.84487	23.40509	0.322916	23.48845	26.45516	-2.96671	0.939984	-3.9067	14.99798

24hsalt_stem_ST	HSP100-11	33.57513	33.67522	33.69787	0.065315	33.6494	30.54674	3.102668	3.166584	-0.06392	1.0453
6hsalt_leaf_ST	HSP100-11	24.20753	24.29103	24.44807	0.122129	24.31554	27.73442	-3.41888	0.939984	-4.35886	20.51864
6hsalt_stem_ST	HSP100-11	28.17844	28.31796	27.93915	0.191582	28.14518	25.88104	2.264143	3.166584	-0.90244	1.869226
1hsalt_leaf_ST	HSP100-12	30.68306	30.26536	30.66324	0.235643	30.53722	30.59565	-0.05843	0.37819	-0.43662	1.353432
1hsalt_stem_ST	HSP100-12	33.31495	Undetermined	33.46542	0.106401	33.39019	30.66301	2.727174	4.931034	-2.20386	4.607105
24hsalt_leaf_ST	HSP100-12	25.34345	25.59441	24.9851	0.306231	25.30765	26.45516	-1.14751	0.37819	-1.5257	2.879262
24hsalt_stem_ST	HSP100-12	34.47568	34.07215	34.91973	0.423951	34.48918	30.54674	3.942447	4.931034	-0.98859	1.98424
6hsalt_leaf_ST	HSP100-12	26.07913	26.23302	26.00517	0.116239	26.10577	27.73442	-1.62865	0.37819	-2.00684	4.01901
6hsalt_stem_ST	HSP100-12	Undetermined	27.59643	27.00661	0.417066	27.30152	25.88104	1.420479	4.931034	-3.51055	11.39679
1hsalt_leaf_ST	HSP100-16	28.68059	29.13791	29.66096	0.490553	29.15982	30.59565	-1.43583	-3.05802	1.622183	0.324843
1hsalt_stem_ST	HSP100-16	29.91423	30.92076	30.13197	0.529578	30.32232	30.66301	-0.3407	0.107474	-0.44817	1.364308
24hsalt_leaf_ST	HSP100-16	25.78682	25.75318	25.89259	0.072747	25.81086	26.45516	-0.6443	-3.05802	2.413718	0.187672
24hsalt_stem_ST	HSP100-16	31.51789	32.80726	31.45136	0.764344	31.9255	30.54674	1.378766	0.107474	1.271292	0.414289
6hsalt_leaf_ST	HSP100-16	26.45857	26.42566	26.59039	0.087171	26.49154	27.73442	-1.24288	-3.05802	1.815133	0.284178
6hsalt_stem_ST	HSP100-16	29.77603	29.42248	29.96909	0.277204	29.72253	25.88104	3.841491	0.107474	3.734017	0.075153
1hsalt_leaf_ST	HSP100-18	Undetermined	Undetermined	34.9223	#DIV/0!	34.9223	30.59565	4.326649	-0.04711	4.373756	0.048236
1hsalt_stem_ST	HSP100-18	34.12444	Undetermined	34.20323	0.055716	34.16383	30.66301	3.500819	0.197874	3.302945	0.101325
24hsalt_leaf_ST	HSP100-18	31.45136	35.27097	Undetermined	2.700872	33.36117	26.45516	6.906007	-0.04711	6.953114	0.008071
24hsalt_stem_ST	HSP100-18	35.09171	31.51789	32.80726	1.809852	33.13895	30.54674	2.592215	0.197874	2.394341	0.190209
6hsalt_leaf_ST	HSP100-18	35.27097	34.9223	32.72495	1.380346	34.30608	27.73442	6.571654	-0.04711	6.618761	0.010175
6hsalt_stem_ST	HSP100-18	34.73101	33.23652	35.09171	0.983643	34.35308	25.88104	8.472041	0.197874	8.274167	0.00323
1hsalt_leaf_ST	HSP60-05	33.0382	33.11002	33.50274	0.223659	33.21699	30.98837	2.228615	2.073274	0.155341	0.89792
1hsalt_stem_ST	HSP60-05	35.60633	33.48606	32.51612	1.413541	33.8695	32.58468	1.284821	1.23342	0.051401	0.964999
24hsalt_leaf_ST	HSP60-05	29.70767	27.82071	27.70324	1.006127	28.41054	27.11683	1.293706	2.073274	-0.77957	1.716616
24hsalt_stem_ST	HSP60-05	33.52704	33.03802	32.9831	0.26784	33.18272	31.86798	1.314744	1.23342	0.081324	0.94519
6hsalt_leaf_ST	HSP60-05	29.5592	29.82302	29.5971	0.127578	29.65977	28.67466	0.985112	2.073274	-1.08816	2.12603
6hsalt_stem_ST	HSP60-05	32.15236	32.54024	31.91099	0.28394	32.2012	30.38865	1.812545	1.23342	0.579125	0.669369
1hsalt_leaf_ST	HSP60-09	31.02851	30.57032	26.66107	2.146832	29.41997	30.98837	-1.5684	-0.14832	-1.42008	2.676009
1hsalt_stem_ST	HSP60-09	30.92117	30.35887	32.60022	1.043012	31.29342	32.58468	-1.29126	-1.77485	0.483587	0.715197
24hsalt_leaf_ST	HSP60-09	26.563	26.7531	26.6668	0.085134	26.66097	27.11683	-0.45586	-0.14832	-0.30754	1.237599
24hsalt_stem_ST	HSP60-09	32.83416	31.90778	32.17202	0.426839	32.30465	31.86798	0.436677	-1.77485	2.211525	0.215906

6hsalt_leaf_ST	HSP60-09	28.0561	28.37367	29.25517	0.555657	28.56164	28.67466	-0.11302	-0.14832	0.035305	0.975825
6hsalt_stem_ST	HSP60-09	30.69705	31.02624	30.853	0.147288	30.85876	30.38865	0.470115	-1.77485	2.244964	0.210959
1hsalt_leaf_ST	HSP60-13	30.72092	31.02899	31.34821	0.280549	31.03271	30.98837	0.044335	-1.11553	1.159869	0.447553
1hsalt_stem_ST	HSP60-13	29.73103	30.95524	31.33834	0.750903	30.67487	32.58468	-1.90981	-3.58785	1.678043	0.312506
24hsalt_leaf_ST	HSP60-13	26.73174	26.95258	26.74114	0.11169	26.80849	27.11683	-0.30834	-1.11553	0.807191	0.571493
24hsalt_stem_ST	HSP60-13	31.77611	31.79769	31.62458	0.084379	31.73279	31.86798	-0.13518	-3.58785	3.452672	0.091336
6hsalt_leaf_ST	HSP60-13	28.03131	31.71158	29.5749	1.652976	29.77259	28.67466	1.097934	-1.11553	2.213468	0.215615
6hsalt_stem_ST	HSP60-13	30.85674	30.61984	31.05035	0.192856	30.84231	30.38865	0.453662	-3.58785	4.041517	0.060727
1hsalt_leaf_ST	HSP60-14	31.59664	32.31396	32.067	0.32594	31.99253	30.98837	1.004163	1.098698	-0.09454	1.067721
1hsalt_stem_ST	HSP60-14	Undetermined	35.01448	Undetermined	0	35.01448	32.58468	2.429804	1.994256	0.435548	0.739413
24hsalt_leaf_ST	HSP60-14	27.16178	32.0122	25.6173	2.984554	28.26376	27.11683	1.14693	1.098698	0.048232	0.967121
24hsalt_stem_ST	HSP60-14	35.70366	Undetermined	Undetermined	0	35.70366	31.86798	3.83568	1.994256	1.841424	0.279046
6hsalt_leaf_ST	HSP60-14	Undetermined	33.73105	35.99252	1.305659	34.86179	28.67466	6.187125	1.098698	5.088427	0.029392
6hsalt_stem_ST	HSP60-14	32.98121	33.89576	34.38222	0.636223	33.75306	30.38865	3.364412	1.994256	1.370156	0.386849
1hsalt_leaf_ST	HSP60-16	29.65759	29.92016	29.67707	0.146295	29.75161	30.59565	-0.84404	-1.82249	0.978447	0.507526
1hsalt_stem_ST	HSP60-16	29.01649	28.17854	28.60578	0.419002	28.60027	30.66301	-2.06275	-1.31644	-0.74631	1.677499
24hsalt_leaf_ST	HSP60-16	25.67391	25.98591	25.34668	0.319643	25.66883	26.45516	-0.78633	-1.82249	1.036163	0.487623
24hsalt_stem_ST	HSP60-16	30.87603	30.88447	30.53872	0.197228	30.7664	30.54674	0.219668	-1.31644	1.536103	0.344816
6hsalt_leaf_ST	HSP60-16	26.9416	26.69354	26.95652	0.147714	26.86388	27.73442	-0.87054	-1.82249	0.951955	0.516931
6hsalt_stem_ST	HSP60-16	28.73019	28.65694	29.00513	0.183569	28.79742	25.88104	2.916379	-1.31644	4.232814	0.053186
1hsalt_leaf_ST	HSP90-03	28.41702	28.94089	28.73528	0.263945	28.69773	30.59565	-1.89792	-1.37812	-0.5198	1.433754
1hsalt_stem_ST	HSP90-03	30.76478	30.5011	29.55434	0.636534	30.2734	30.66301	-0.38961	-1.60754	1.217931	0.429899
24hsalt_leaf_ST	HSP90-03	23.73397	23.96059	23.97176	0.13418	23.88877	26.45516	-2.56639	-1.37812	-1.18826	2.278783
24hsalt_stem_ST	HSP90-03	29.99067	30.30667	30.94248	0.484776	30.41327	30.54674	-0.13346	-1.60754	1.474079	0.359963
6hsalt_leaf_ST	HSP90-03	24.86796	24.99712	25.17829	0.155888	25.01446	27.73442	-2.71996	-1.37812	-1.34184	2.53474
6hsalt_stem_ST	HSP90-03	29.66367	29.09223	30.49269	0.704169	29.74953	25.88104	3.86849	-1.60754	5.476031	0.022467
1hsalt_leaf_ST	HSP90-04	27.83323	27.33722	27.99813	0.344004	27.72286	30.59565	-2.87279	-4.34938	1.476594	0.359336
1hsalt_stem_ST	HSP90-04	30.56523	30.72263		0.111299	30.64393	30.66301	-0.01909	-0.82806	0.808975	0.570787
24hsalt_leaf_ST	HSP90-04	25.90134	25.69761	25.28577	0.313594	25.62824	26.45516	-0.82692	-4.34938	3.52246	0.087023
24hsalt_stem_ST	HSP90-04	33.05872	32.93028	34.34561	0.782704	33.44487	30.54674	2.89813	-0.82806	3.726192	0.075562
6hsalt_leaf_ST	HSP90-04	25.46611	25.38219	25.70358	0.166695	25.51729	27.73442	-2.21713	-4.34938	2.132257	0.228101

6hsalt_stem_ST	HSP90-04	29.64027	29.74284	29.91677	0.139773	29.76663	25.88104	3.885585	-0.82806	4.713648	0.038111
1hsalt_leaf_ST	HSP90-05	28.95674	29.58689	29.54631	0.352689	29.36331	30.59565	-1.23234	-2.88662	1.654279	0.317697
1hsalt_stem_ST	HSP90-05	31.65352	31.43608	29.8681	0.974128	30.9859	30.66301	0.322885	-0.85395	1.176838	0.44232
24hsalt_leaf_ST	HSP90-05	26.97051	26.00229	26.23774	0.504949	26.40351	26.45516	-0.05165	-2.88662	2.834968	0.140149
24hsalt_stem_ST	HSP90-05	32.11224	32.04483	32.09435	0.034916	32.08381	30.54674	1.53707	-0.85395	2.391023	0.190647
6hsalt_leaf_ST	HSP90-05	26.84052	26.97051	27.13714	0.148689	26.98273	27.73442	-0.7517	-2.88662	2.13492	0.22768
6hsalt_stem_ST	HSP90-05	30.5365	31.12898	31.2872	0.395733	30.98423	25.88104	5.103185	-0.85395	5.957138	0.016096
1hsalt_leaf_ST	HSP90-09	29.16493	29.08585	28.52606	0.348273	28.92561	30.59565	-1.67004	1.241222	-2.91126	7.522742
1hsalt_stem_ST	HSP90-09	31.33447	31.35628	31.88191	0.30996	31.52422	30.66301	0.861204	5.641408	-4.7802	27.47798
24hsalt_leaf_ST	HSP90-09	25.01671	25.42921	25.7022	0.345101	25.38271	26.45516	-1.07245	1.241222	-2.31368	4.971481
24hsalt_stem_ST	HSP90-09	32.54982	32.31093	34.19218	1.024166	33.01764	30.54674	2.470905	5.641408	-3.1705	9.003606
6hsalt_leaf_ST	HSP90-09	25.78981	25.78692	26.06639	0.160519	25.88104	25.88104	0	1.241222	-1.24122	2.363987
6hsalt_stem_ST	HSP90-09	29.92593	30.22651	30.61878	0.347435	30.25707	25.88104	4.376031	5.641408	-1.26538	2.403901
1hsalt_leaf_ST	sHSP-02	32.81208	32.90866	31.69047	0.605674	32.4704	30.98837	1.482033	1.994441	-0.51241	1.42643
1hsalt_stem_ST	sHSP-02	34.50449	34.58097	Undetermined	0.044152	34.54273	32.58468	1.958051	2.032746	-0.07469	1.053138
24hsalt_leaf_ST	sHSP-02	26.63829	26.7759	26.93845	0.134392	26.78421	27.11683	-0.33262	1.994441	-2.32706	5.017819
24hsalt_stem_ST	sHSP-02	34.33418	34.43386	31.82508	1.322184	33.53104	31.86798	1.663065	2.032746	-0.36968	1.292066
6hsalt_leaf_ST	sHSP-02	28.70565	28.34646	28.73429	0.193303	28.59547	28.67466	-0.07919	1.994441	-2.07363	4.209456
6hsalt_stem_ST	sHSP-02	32.24707	29.50997	32.25597	1.415737	31.33767	30.38865	0.949022	2.032746	-1.08372	2.1195
1hsalt_leaf_ST	sHSP-07	33.46199	33.80585	32.64458	0.533536	33.30414	30.98837	2.315769	3.379002	-1.06323	2.089608
1hsalt_stem_ST	sHSP-07	Undetermined	Undetermined	36.3201	0	36.3201	32.58468	3.735423	2.032746	1.702678	0.307215
24hsalt_leaf_ST	sHSP-07	26.87953	27.17064	28.12113	0.580772	27.39043	27.11683	0.273602	3.379002	-3.1054	8.606339
24hsalt_stem_ST	sHSP-07	35.73283	Undetermined	36.92983	0.691087	36.33133	31.86798	4.463358	2.032746	2.430612	0.185487
6hsalt_leaf_ST	sHSP-07	28.67901	28.65925	29.00215	0.172197	28.78013	28.67466	0.105474	3.379002	-3.27353	9.670081
6hsalt_stem_ST	sHSP-07	36.66811	Undetermined	Undetermined	0	36.66811	30.38865	6.279456	2.032746	4.246711	0.052676
1hsalt_leaf_ST	sHSP-11	31.40094	31.89075	31.58426	0.221346	31.62532	30.98837	0.636945	0.844496	-0.20755	1.154726
1hsalt_stem_ST	sHSP-11	34.33791	33.70746	35.97046	1.044594	34.67194	32.58468	2.087261	2.81045	-0.72319	1.650827
24hsalt_leaf_ST	sHSP-11	26.94121	26.8633	26.81724	0.056047	26.87391	27.11683	-0.24292	0.844496	-1.08741	2.124927
24hsalt_stem_ST	sHSP-11	31.57988	31.67618	31.57555	0.050884	31.61054	31.86798	-0.25744	2.81045	-3.06789	8.385445
6hsalt_leaf_ST	sHSP-11	28.50778	28.03451	28.1737	0.217553	28.23866	28.67466	-0.436	0.844496	-1.28049	2.429217
6hsalt_stem_ST	sHSP-11	30.34909	30.40367	30.66465	0.15085	30.47247	30.38865	0.083818	2.81045	-2.72663	6.619084

1hsalt_leaf_ST	sHSP-15	30.99021	32.65377	31.08171	0.836437	31.57523	30.98837	0.586861	0.777854	-0.19099	1.141549
1hsalt_stem_ST	sHSP-15	33.97892	35.78746	33.977	0.934422	34.58112	32.58468	1.996445	1.623717	0.372728	0.772321
24hsalt_leaf_ST	sHSP-15	26.33299	26.2733	26.37564	0.045981	26.32731	27.11683	-0.78952	0.777854	-1.56738	2.963652
24hsalt_stem_ST	sHSP-15	Undetermined	35.62562	35.51838	0.061912	35.572	31.86798	3.704025	1.623717	2.080308	0.236464
6hsalt_leaf_ST	sHSP-15	28.85445	28.45657	28.58456	0.181669	28.63186	28.67466	-0.0428	0.777854	-0.82066	1.766209
6hsalt_stem_ST	sHSP-15	33.39828	33.531	33.84618	0.205774	33.59182	30.38865	3.203172	1.623717	1.579455	0.334608
1hsalt_leaf_ST	sHSP-25	32.38744	32.76196	32.38599	0.193774	32.5118	30.98837	1.523425	0.658062	0.865363	0.548908
1hsalt_stem_ST	sHSP-25	35.12326	Undetermined	35.22429	0.058327	35.17377	32.58468	2.589093	3.365561	-0.77647	1.712932
24hsalt_leaf_ST	sHSP-25	26.44326	26.51584	26.40086	0.052008	26.45332	27.11683	-0.66351	0.658062	-1.32157	2.499384
24hsalt_stem_ST	sHSP-25	Undetermined	35.31896	33.04511	1.312808	34.18204	31.86798	2.31406	3.365561	-1.0515	2.072686
6hsalt_leaf_ST	sHSP-25	28.24348	28.31382	28.23427	0.038921	28.26386	28.67466	-0.4108	0.658062	-1.06887	2.097783
6hsalt_stem_ST	sHSP-25	32.63204	32.75892	32.79751	0.077433	32.72949	30.38865	2.340841	3.365561	-1.02472	2.034564
1hsalt_leaf_ST	sHSP-26	32.41454	31.7491	31.58443	0.393107	31.91603	30.98837	0.927655	0.788017	0.139638	0.907747
1hsalt_stem_ST	sHSP-26	Undetermined	35.5214	Undetermined	0	35.5214	32.58468	2.936717	3.274928	-0.33821	1.264188
24hsalt_leaf_ST	sHSP-26	26.31472	27.16257	26.77635	0.379669	26.75122	27.11683	-0.36561	0.788017	-1.15363	2.224731
24hsalt_stem_ST	sHSP-26	36.59771	36.18228	35.79289	0.359987	36.19096	31.86798	4.322984	3.274928	1.048056	0.483619
6hsalt_leaf_ST	sHSP-26	28.40483	28.39793	28.52541	0.064121	28.44272	28.67466	-0.23194	0.788017	-1.01996	2.027856
6hsalt_stem_ST	sHSP-26	33.05307	34.4377	32.96672	0.738327	33.48583	30.38865	3.097181	3.274928	-0.17775	1.131116
1hsalt_leaf_ST	sHSP-27	34.52968	31.08104	32.40417	1.556132	32.67163	30.98837	1.683261	2.00304	-0.31978	1.24814
1hsalt_stem_ST	sHSP-27	33.17757	Undetermined	Undetermined	0	33.17757	32.58468	0.592887	2.979305	-2.38642	5.228574
24hsalt_leaf_ST	sHSP-27	25.87383	25.72547	26.19626	0.215289	25.93186	27.11683	-1.18498	2.00304	-3.18802	9.113566
24hsalt_stem_ST	sHSP-27	34.95469	34.9197	35.90403	0.499516	35.25947	31.86798	3.391495	2.979305	0.41219	0.751482
6hsalt_leaf_ST	sHSP-27	27.7945	27.72374	28.42882	0.347276	27.98235	28.67466	-0.69231	2.00304	-2.69535	6.477103
6hsalt_stem_ST	sHSP-27	32.43135	32.31139	33.42099	0.544669	32.72124	30.38865	2.332595	2.979305	-0.64671	1.565594
1hsalt_leaf_ST	sHSP-30	31.6191	32.21506	33.17901	0.704056	32.33772	31.86798	0.469745	3.274928	-2.80518	6.989468
1hsalt_stem_ST	sHSP-30	29.35702	30.4772	30.10041	0.509823	29.97821	28.67466	1.303546	0.788017	0.515529	0.699536
24hsalt_leaf_ST	sHSP-30	31.97957	32.11514	32.6424	0.313203	32.2457	30.38865	1.857049	3.274928	-1.41788	2.671923
24hsalt_stem_ST	sHSP-30	34.18128	34.34284	34.57661	0.177777	34.36691	30.98837	3.37854	0.788017	2.590523	0.166026
6hsalt_leaf_ST	sHSP-30	31.63097	32.11416	31.79771	0.219519	31.84761	32.58468	-0.73707	-0.66351	-0.07356	1.052307
6hsalt_stem_ST	sHSP-30	32.67087	33.16291	32.64088	0.262176	32.82489	28.67466	4.150224	2.31406	1.836165	0.280065

Susceptible cultivar (IC 41) in response to salinity stress											
**SAMPLE**	**DETECTOR**	**Ct1**	**Ct2**	**Ct3**	**sd**	**Avg Ct**	**End ctl**	**dCt**	**CTL 0h**	**ddCt**	**log Ct**
1hsalt_leaf_SS	ACT	21.82279	21.82279	22.36743	0.31445	22.00433	22.00433	0		0	1
1hsalt_stem_SS	ACT	24.90869	24.90869	22.88948	1.165792	24.23562	24.23562	0		0	1
24hsalt_leaf_SS	ACT	23.65528	23.65528	22.57499	0.623702	23.29518	23.29518	0		0	1
24hsalt_stem_SS	ACT	Undetermined	22.46122	22.25774	0.143879	22.35948	22.35948	0		0	1
6hsalt_leaf_SS	ACT	24.12278	24.12278	28.8091	2.705649	25.68489	25.68489	0		0	1
6hsalt_stem_SS	ACT	25.40308	25.40308	24.47053	0.53841	25.09223	25.09223	0		0	1
1hsalt_leaf_SS	HSP100-07	32.27287	32.95839	32.19918	0.418686	32.47681	22.00433	10.47248	4.256441	6.216036	0.013452
1hsalt_stem_SS	HSP100-07	29.89261	28.98958	29.09169	0.49453	29.32463	24.23562	5.089009	7.113136	-2.02413	4.067457
24hsalt_leaf_SS	HSP100-07	29.22626	29.40761	29.60961	0.191768	29.41449	23.29518	6.11931	4.256441	1.862868	0.274929
24hsalt_stem_SS	HSP100-07	22.32156	21.7606	22.40939	0.351978	22.16385	22.35948	-0.19563	7.113136	-7.30876	158.5467
6hsalt_leaf_SS	HSP100-07	30.48824	30.48824	31.31486	0.477248	30.76378	25.68489	5.078894	4.256441	0.822452	0.56548
6hsalt_stem_SS	HSP100-07	27.27622	27.27622	28.52194	0.719213	27.69146	25.09223	2.599229	7.113136	-4.51391	22.8466
1hsalt_leaf_SS	HSP100-08	29.35499	29.35499	28.55631	0.461116	29.08876	22.00433	7.084427	2.530401	4.554026	0.04257
1hsalt_stem_SS	HSP100-08	29.14879	29.14879	28.81502	0.192699	29.03753	24.23562	4.801917	7.268533	-2.46662	5.52746
24hsalt_leaf_SS	HSP100-08	27.5022	27.5022	27.34631	0.090003	27.45024	23.29518	4.155054	2.530401	1.624654	0.324288
24hsalt_stem_SS	HSP100-08	25.32388	25.32388	25.29497	0.016689	25.31424	22.35948	2.954764	7.268533	-4.31377	19.88722
6hsalt_leaf_SS	HSP100-08	29.51787	29.51787	30.25261	0.4242	29.76279	25.68489	4.077901	2.530401	1.5475	0.342102
6hsalt_stem_SS	HSP100-08	25.55366	25.55366	25.44615	0.062069	25.51782	25.09223	0.42559	7.268533	-6.84294	114.7972
1hsalt_leaf_SS	HSP100-11	29.86441	29.86441	30.76761	0.521462	30.16547	22.00433	8.16114	8.080248	0.080892	0.945473
1hsalt_stem_SS	HSP100-11	33.31073	32.76572	34.33891	0.798864	33.47179	24.23562	9.23617	10.47297	-1.2368	2.356746
24hsalt_leaf_SS	HSP100-11	32.46313	33.36459	33.3636	0.520172	33.06377	23.29518	9.76859	8.080248	1.688342	0.310283
24hsalt_stem_SS	HSP100-11	25.78315	25.50623	22.00433	2.106318	24.43124	22.35948	2.071762	10.47297	-8.4012	338.076
6hsalt_leaf_SS	HSP100-11	27.16069	26.93033	24.23562	1.626376	26.10888	25.68489	0.423993	8.080248	-7.65626	201.7264
6hsalt_stem_SS	HSP100-11	32.86541	33.64767	33.29518	0.391765	33.26942	25.09223	8.177193	10.47297	-2.29577	4.91017
1hsalt_leaf_SS	HSP100-12	29.33995	29.56252	22.35948	4.095939	27.08732	22.00433	5.082981	11.20091	-6.11792	69.45102
1hsalt_stem_SS	HSP100-12	29.2584	28.65276	25.68489	1.912464	27.86535	24.23562	3.629734	12.39666	-8.76692	435.6187
24hsalt_leaf_SS	HSP100-12	25.62512	25.74655	25.09223	0.348054	25.48797	23.29518	2.192784	4.19763	-2.00485	4.013457
24hsalt_stem_SS	HSP100-12	25.63791	26.70231	22.00433	2.463291	24.78152	22.35948	2.42204	3.9666	-1.54456	2.917151
6hsalt_leaf_SS	HSP100-12	27.51397	27.25423	24.23562	1.822413	26.33461	25.68489	0.649721	2.0091	-1.35938	2.565746

6hsalt_stem_SS	HSP100-12	26.45982	26.6169	22.35948	2.413957	25.1454	25.09223	0.053168	12.39666	-12.3435	5197.087
1hsalt_leaf_SS	HSP100-16	25.55484	25.55484	25.40901	0.0842	25.50623	22.00433	3.501897	5.374351	-1.87245	3.661547
1hsalt_stem_SS	HSP100-16	26.70303	26.70303	27.38495	0.393707	26.93033	24.23562	2.694717	7.333867	-4.63915	24.91858
24hsalt_leaf_SS	HSP100-16	33.53222	33.53222	33.87857	0.199966	33.64767	23.29518	10.35249	5.374351	4.97814	0.031727
24hsalt_stem_SS	HSP100-16	29.41576	29.41576	29.85604	0.254193	29.56252	22.35948	7.20304	7.333867	-0.13083	1.094921
6hsalt_leaf_SS	HSP100-16	28.52419	28.52419	28.9099	0.222693	28.65276	25.68489	2.967874	5.374351	-2.40648	5.301781
6hsalt_stem_SS	HSP100-16	25.74335	25.74335	25.75294	0.005539	25.74655	25.09223	0.654317	7.333867	-6.67955	102.505
1hsalt_leaf_SS	HSP100-18	26.02708	26.22297	26.50936	0.242551	26.25313	22.00433	4.248799	5.008038	-0.75924	1.692598
1hsalt_stem_SS	HSP100-18	33.1767	33.80206	33.12407	0.377165	33.36761	24.23562	9.131993	7.268533	1.863459	0.274817
24hsalt_leaf_SS	HSP100-18	30.17745	30.55206	30.54793	0.215099	30.42582	23.29518	7.130633	5.008038	2.122595	0.229633
24hsalt_stem_SS	HSP100-18	23.73833	24.67634	24.31203	0.472885	24.24223	22.35948	1.882751	7.268533	-5.38578	41.81017
6hsalt_leaf_SS	HSP100-18	24.63038	25.27679	24.45398	0.433202	24.78705	25.68489	-0.89784	5.008038	-5.90587	59.95775
6hsalt_stem_SS	HSP100-18	21.3247	21.74624	21.43174	0.219112	21.50089	25.09223	-3.59134	2.68593	-6.27727	77.5614
1hsalt_leaf_SS	HSP100-20	24.48857	23.81845	24.29907	0.345436	24.20203	22.00433	2.197695	9.563736	-7.36604	164.9678
1hsalt_stem_SS	HSP100-20	34.27634	33.99267	32.41239	1.004328	33.56047	24.23562	9.32485	4.785678	4.539172	0.04301
24hsalt_leaf_SS	HSP100-20	26.54079	26.66036	27.2404	0.374211	26.81385	23.29518	3.518667	9.563736	-6.04507	66.03085
24hsalt_stem_SS	HSP100-20	25.18256	25.45622	25.64271	0.231448	25.42716	22.35948	3.067682	4.785678	-1.718	3.28979
6hsalt_leaf_SS	HSP100-20	22.06608	22.41697	22.47828	0.222406	22.32045	25.68489	-3.36444	1.1087	-4.47314	22.21004
6hsalt_stem_SS	HSP100-20	26.04772	26.81858	24.87534	0.97851	25.91388	25.09223	0.821647	4.785678	-3.96403	15.60602
1hsalt_leaf_SS	HSP60-02	29.67634	29.93033	29.40471	0.262857	29.67046	22.00433	7.666123	5.960718	1.705405	0.306635
1hsalt_stem_SS	HSP60-02	26.51499	26.83705	26.71482	0.162582	26.68895	24.23562	2.453338	10.27932	-7.82598	226.9113
24hsalt_leaf_SS	HSP60-02	27.50985	27.62151	27.58974	0.057531	27.5737	23.29518	4.278519	5.960718	-1.6822	3.209168
24hsalt_stem_SS	HSP60-02	26.92863	26.92576	26.80374	0.071293	26.88605	22.35948	4.526568	10.27932	-5.75275	53.92023
6hsalt_leaf_SS	HSP60-02	27.21439	27.29408	27.51397	0.155163	27.34081	25.68489	1.655928	5.960718	-4.30479	19.76383
6hsalt_stem_SS	HSP60-02	26.91216	26.84743	26.64076	0.141752	26.80012	25.09223	1.707884	10.27932	-8.57144	380.4171
1hsalt_leaf_SS	HSP60-03	34.30904	34.30904	34.45161	0.082313	34.35657	22.00433	12.35223	11.58692	0.76531	0.588327
1hsalt_stem_SS	HSP60-03	32.80377	32.80377	34.83309	1.171627	33.48021	24.23562	9.244594	9.741697	-0.4971	1.411377
24hsalt_leaf_SS	HSP60-03	33.56814	33.56814	33.97659	0.235815	33.70429	23.29518	10.40911	11.58692	-1.17782	2.262341
24hsalt_stem_SS	HSP60-03	34.04357	34.04357	32.84406	0.692539	33.64373	22.35948	11.28425	9.741697	1.542555	0.343277
6hsalt_leaf_SS	HSP60-03	34.7853	34.7853	34.53232	0.146058	34.70097	25.68489	9.016088	11.58692	-2.57084	5.941535
6hsalt_stem_SS	HSP60-03	35.01767	35.01767	35.02747	0.005656	35.02094	25.09223	9.928705	9.741697	0.187008	0.878426

1hsalt_leaf_SS	HSP60-05	25.62607	25.51329	25.56968	0.056392	25.56968	22.00433	3.565343	4.621545	-1.0562	2.079451
1hsalt_stem_SS	HSP60-05	33.70147	35.93682	34.81914	1.117676	34.81914	24.23562	10.58353	4.30362	6.279906	0.012869
24hsalt_leaf_SS	HSP60-05	Undetermined	36.04499	36.04499	0	36.04499	23.29518	12.7498	4.621545	8.12826	0.003574
24hsalt_stem_SS	HSP60-05	29.95771	28.93299	29.44535	0.51236	29.44535	22.35948	7.085872	4.30362	2.782252	0.145365
6hsalt_leaf_SS	HSP60-05	26.85995	27.63398	27.24696	0.387016	27.24696	25.68489	1.562077	4.621545	-3.05947	8.33665
6hsalt_stem_SS	HSP60-05	29.32695	29.90835	29.61765	0.290698	29.61765	25.09223	4.525417	4.30362	0.221798	0.857496
1hsalt_leaf_SS	HSP60-09	25.93134	25.91768	25.87404	0.02993	25.90768	22.00433	3.90335	2.370853	1.532497	0.345679
1hsalt_stem_SS	HSP60-09	25.59783	25.9514	25.69899	0.182098	25.74941	24.23562	1.513793	4.484283	-2.97049	7.838026
24hsalt_leaf_SS	HSP60-09	22.62301	22.88767	22.73094	0.13308	22.74721	23.29518	-0.54797	2.370853	-2.91883	7.562311
24hsalt_stem_SS	HSP60-09	27.27498	27.27498	26.59991	0.38975	27.04995	22.35948	4.690477	4.484283	0.206193	0.866821
6hsalt_leaf_SS	HSP60-09	27.58104	27.58104	27.67001	0.051365	27.6107	25.68489	1.925811	2.370853	-0.44504	1.361354
6hsalt_stem_SS	HSP60-09	25.76633	25.76633	25.46566	0.17359	25.6661	25.09223	0.573872	4.484283	-3.91041	15.03665
1hsalt_leaf_SS	HSP60-13	25.76866	25.76866	25.43453	0.192908	25.65728	22.00433	3.652951	1.320383	2.332567	0.198531
1hsalt_stem_SS	HSP60-13	27.92614	27.92614	28.25298	0.188699	28.03509	24.23562	3.79947	2.556249	1.243221	0.422429
24hsalt_leaf_SS	HSP60-13	26.34179	26.34179	26.47849	0.078922	26.38736	23.29518	3.092176	1.320383	1.771793	0.292845
24hsalt_stem_SS	HSP60-13	25.44403	25.44403	26.54164	0.633705	25.8099	22.35948	3.450422	2.556249	0.894173	0.538056
6hsalt_leaf_SS	HSP60-13	26.74545	26.51623	26.85495	0.172852	26.70554	25.68489	1.020658	1.320383	-0.29972	1.23091
6hsalt_stem_SS	HSP60-13	26.34935	26.00218	28.30709	1.242704	26.88621	25.09223	1.793977	2.556249	-0.76227	1.69616
1hsalt_leaf_SS	HSP60-14	24.98068	24.93107	24.96277	0.025124	24.95817	22.00433	2.953839	1.986789	0.96705	0.511551
1hsalt_stem_SS	HSP60-14	31.66244	31.23182	30.90209	0.381287	31.26545	24.23562	7.029832	8.863524	-1.83369	3.56448
24hsalt_leaf_SS	HSP60-14	27.83777	27.62417	27.63667	0.119876	27.69954	23.29518	4.404357	1.986789	2.417568	0.187171
24hsalt_stem_SS	HSP60-14	31.66244	31.23182	30.90209	0.381287	31.26545	22.35948	8.90597	8.863524	0.042446	0.971007
6hsalt_leaf_SS	HSP60-14	29.75375	29.42837	29.7268	0.180583	29.63631	25.68489	3.951423	1.986789	1.964633	0.256204
6hsalt_stem_SS	HSP60-14	26.83087	26.83087	27.30869	0.275872	26.99014	25.09223	1.89791	8.863524	-6.96561	124.9852
1hsalt_leaf_SS	HSP60-16	24.70204	24.70204	25.39839	0.402037	24.93416	22.00433	2.929822	3.760658	-0.83084	1.778716
1hsalt_stem_SS	HSP60-16	33.83374	33.74123	Undetermined	0.065412	33.78748	24.23562	9.551867	5.297616	4.254252	0.052401
24hsalt_leaf_SS	HSP60-16	32.48546	32.9318	32.46941	0.262448	32.62889	23.29518	9.333708	3.760658	5.57305	0.021006
24hsalt_stem_SS	HSP60-16	26.83395	27.43641	27.49717	0.366631	27.25585	22.35948	4.896369	5.297616	-0.40125	1.320649
6hsalt_leaf_SS	HSP60-16	27.76479	28.28676	26.72406	0.795569	27.59187	25.68489	1.906985	3.760658	-1.85367	3.614193
6hsalt_stem_SS	HSP60-16	Undetermined	34.12079	33.24667	0.61809	33.68373	25.09223	8.591499	5.297616	3.293883	0.101963
1hsalt_leaf_SS	HSP60-20	28.04167	28.79756	29.02602	0.515185	28.62175	22.00433	6.617413	6.426404	0.191009	0.875993

1hsalt_stem_SS	HSP60-20	27.57944	27.57154	28.06842	0.284625	27.7398	24.23562	3.504183	5.109433	-1.60525	3.042485
24hsalt_leaf_SS	HSP60-20	27.58889	26.49066	26.90824	0.554346	26.99593	23.29518	3.700747	6.426404	-2.72566	6.614617
24hsalt_stem_SS	HSP60-20	30.1839	28.17207	28.48935	1.081637	28.94844	22.35948	6.588962	5.109433	1.479528	0.358606
6hsalt_leaf_SS	HSP60-20	27.58889	27.14226	27.35905	0.22335	27.3634	25.68489	1.678514	6.426404	-4.74789	26.86936
6hsalt_stem_SS	HSP60-20	27.48028	27.50985	27.62151	0.074484	27.53722	25.09223	2.444985	5.109433	-2.66445	6.339847
1hsalt_leaf_SS	HSP70-01	25.33198	25.45327	25.95345	0.329425	25.57956	22.00433	3.57523	5.444796	-1.86957	3.654226
1hsalt_stem_SS	HSP70-01	27.48028	27.55498	28.3523	0.483341	27.79585	24.23562	3.560239	4.265441	-0.7052	1.630373
24hsalt_leaf_SS	HSP70-01	30.74076	31.28638	30.67258	0.336427	30.89991	23.29518	7.604724	5.444796	2.159928	0.223767
24hsalt_stem_SS	HSP70-01	33.31073	32.76572	34.33891	0.798864	33.47179	22.35948	11.11231	4.265441	6.846866	0.008687
6hsalt_leaf_SS	HSP70-01	32.46313	33.36459	33.3636	0.520172	33.06377	25.68489	7.378887	5.444796	1.934091	0.261686
6hsalt_stem_SS	HSP70-01	31.87148	31.70417	32.25341	0.28152	31.94302	25.09223	6.850788	4.265441	2.585347	0.166622
1hsalt_leaf_SS	HSP70-04	26.487	26.73391	26.67071	0.128264	26.63054	22.00433	4.626208	1.272139	3.354068	0.097797
1hsalt_stem_SS	HSP70-04	30.98024	31.92718	31.77396	0.508292	31.56046	24.23562	7.324844	7.406306	-0.08146	1.05809
24hsalt_leaf_SS	HSP70-04	25.45327	26.06545	25.57944	0.32324	25.69939	23.29518	2.404203	1.272139	1.132064	0.456263
24hsalt_stem_SS	HSP70-04	27.48028	27.83777	27.62417	0.179873	27.64741	22.35948	5.287932	7.406306	-2.11837	4.342043
6hsalt_leaf_SS	HSP70-04	27.48028	27.48628	27.58889	0.06105	27.51848	25.68489	1.833599	1.272139	0.56146	0.677616
6hsalt_stem_SS	HSP70-04	27.48028	26.31973	Undetermined	0.820636	26.90001	25.09223	1.807776	7.406306	-5.59853	48.45352
1hsalt_leaf_SS	HSP70-06	30.44643	30.1839	30.02603	0.212362	30.21879	22.00433	8.214454	9.314555	-1.1001	2.143697
1hsalt_stem_SS	HSP70-06	25.33198	25.93134	25.91768	0.342167	25.727	24.23562	1.491382	11.81958	-10.3282	1285.571
24hsalt_leaf_SS	HSP70-06	32.64065	32.64065	33.83352	0.688705	33.03827	23.29518	9.74309	9.314555	0.428535	0.743016
24hsalt_stem_SS	HSP70-06	33.07114	33.07114	35.19228	1.224643	33.77818	22.35948	11.41871	11.81958	-0.40087	1.320304
6hsalt_leaf_SS	HSP70-06	34.03621	34.03621	33.2144	0.474474	33.76228	25.68489	8.07739	9.314555	-1.23716	2.357348
6hsalt_stem_SS	HSP70-06	33.48124	33.48124	33.58433	0.059521	33.5156	25.09223	8.42337	11.81958	-3.39621	10.52834
1hsalt_leaf_SS	HSP70-15	30.94935	30.98024	30.80831	0.091655	30.91263	22.00433	8.9083	2.556249	6.35205	0.012242
1hsalt_stem_SS	HSP70-15	28.83676	29.08653	28.85088	0.140307	28.92472	24.23562	4.689107	2.968342	1.720764	0.303388
24hsalt_leaf_SS	HSP70-15	25.33198	25.45327	25.95345	0.329425	25.57956	23.29518	2.284382	2.556249	-0.27187	1.20737
24hsalt_stem_SS	HSP70-15	27.48028	27.55498	28.3523	0.483341	27.79585	22.35948	5.436377	2.968342	2.468034	0.180737
6hsalt_leaf_SS	HSP70-15	23.85489	24.65246	24.65093	0.460037	24.38609	25.68489	-1.2988	2.556249	-3.85505	14.47052
6hsalt_stem_SS	HSP70-15	30.70225	30.70225	30.44643	0.147695	30.61698	25.09223	5.524747	2.968342	2.556405	0.169999
1hsalt_leaf_SS	HSP70-16	30.28738	30.28738	31.36537	0.622379	30.64671	22.00433	8.642379	8.545858	0.096521	0.935286
1hsalt_stem_SS	HSP70-16	32.34478	32.34478	30.48597	1.073186	31.72518	24.23562	7.48956	11.52239	-4.03283	16.36829

24hsalt_leaf_SS	HSP70-16	31.89106	31.89106	32.78183	0.514286	32.18799	23.29518	8.892805	8.545858	0.346947	0.786246
24hsalt_stem_SS	HSP70-16	34.5104	34.5104	33.96862	0.312795	34.32981	22.35948	11.97033	11.52239	0.447938	0.73309
6hsalt_leaf_SS	HSP70-16	32.29041	32.29041	32.45801	0.096766	32.34627	25.68489	6.661387	8.545858	-1.88447	3.692173
6hsalt_stem_SS	HSP70-16	29.86441	29.86441	30.76761	0.521462	30.16547	25.09223	5.073244	11.52239	-6.44915	87.37498
1hsalt_leaf_SS	HSP70-19	32.28615	32.28615	31.25241	0.596831	31.94157	22.00433	9.937234	8.184944	1.75229	0.29683
1hsalt_stem_SS	HSP70-19	33.88503	33.88503	34.77167	0.511902	34.18058	24.23562	9.944961	11.34792	-1.40296	2.644427
24hsalt_leaf_SS	HSP70-19	32.49904	32.49904	33.41288	0.527604	32.80366	23.29518	9.508473	8.184944	1.323529	0.399556
24hsalt_stem_SS	HSP70-19	35.72421	35.72421	35.98723	0.151853	35.81189	22.35948	13.45241	11.34792	2.104491	0.232533
6hsalt_leaf_SS	HSP70-19	30.70225	30.70225	30.44643	0.147695	30.61698	25.68489	4.932093	8.184944	-3.25285	9.532476
6hsalt_stem_SS	HSP70-19	31.90872	31.90872	31.94539	0.02117	31.92094	25.09223	6.828714	11.34792	-4.5192	22.9306
1hsalt_leaf_SS	HSP70-21	Undetermined	27.21439	27.29408	0.056354	27.25423	22.00433	5.249901	4.361949	0.887952	0.540381
1hsalt_stem_SS	HSP70-21	30.21587	30.21587	30.60879	0.226852	30.34684	24.23562	6.111227	7.347949	-1.23672	2.356624
24hsalt_leaf_SS	HSP70-21	34.89123	34.89123	34.71727	0.100432	34.83324	23.29518	11.53806	4.361949	7.176112	0.006915
24hsalt_stem_SS	HSP70-21	35.39623	35.39623	33.01996	1.37194	34.60414	22.35948	12.24466	7.347949	4.896713	0.033569
6hsalt_leaf_SS	HSP70-21	32.01457	32.48546	32.9318	0.458668	32.47728	25.68489	6.79239	4.361949	2.430441	0.185509
6hsalt_stem_SS	HSP70-21	25.4871	25.09282	26.04938	0.480734	25.5431	25.09223	0.450868	3.47949	-3.02862	8.160297
1hsalt_leaf_SS	HSP70-24	Undetermined	25.82601	25.74098	0.060127	25.78349	22.00433	3.77916	4.534038	-0.75488	1.687489
1hsalt_stem_SS	HSP70-24	28.66223	28.02286	27.72693	0.478043	28.13734	24.23562	3.901725	7.550413	-3.64869	12.54193
24hsalt_leaf_SS	HSP70-24	29.2345	29.72495	28.61203	0.557762	29.19049	23.29518	5.895309	4.534038	1.361271	0.389239
24hsalt_stem_SS	HSP70-24	27.84632	27.76479	28.28676	0.2808	27.96596	22.35948	5.606477	7.550413	-1.94394	3.847539
6hsalt_leaf_SS	HSP70-24	25.98752	26.49066	26.90824	0.461021	26.46214	25.68489	0.777252	4.534038	-3.75679	13.51777
6hsalt_stem_SS	HSP70-24	25.01174	26.29036	24.9731	0.749616	25.42507	25.09223	0.332835	7.550413	-7.21758	148.8359
1hsalt_leaf_SS	HSP90-02	27.52328	27.53911	27.7411	0.121444	27.60116	22.00433	5.596829	8.498686	-2.90186	7.47388
1hsalt_stem_SS	HSP90-02	28.02286	28.51739	28.80398	0.395143	28.44808	24.23562	4.212461	3.73102	0.481441	0.716262
24hsalt_leaf_SS	HSP90-02	25.09282	25.99571	26.39964	0.669097	25.82939	23.29518	2.534206	8.498686	-5.96448	62.44355
24hsalt_stem_SS	HSP90-02	30.61218	29.72495	28.61203	1.002196	29.64972	22.35948	7.29024	5.10741	2.18283	0.220243
6hsalt_leaf_SS	HSP90-02	26.21585	25.19537	25.44823	0.531438	25.61982	25.68489	-0.06507	8.498686	-8.56376	378.3969
6hsalt_stem_SS	HSP90-02	25.9315	25.84046	26.03082	0.095207	25.93426	25.09223	0.84203	5.067315	-4.22529	18.70413
1hsalt_leaf_SS	HSP90-03	24.8873	25.93134	25.91768	0.598872	25.57877	22.00433	3.574439	5.039092	-1.46465	2.759972
1hsalt_stem_SS	HSP90-03	27.52328	27.52328	28.93527	0.815209	27.99394	24.23562	3.758327	8.863524	-5.1052	34.42052
24hsalt_leaf_SS	HSP90-03	30.61218	30.61218	30.76115	0.08601	30.66184	23.29518	7.366656	5.039092	2.327563	0.19922

24hsalt_stem_SS	HSP90-03	Undetermined	31.01076	30.9068	0.073511	30.95878	22.35948	8.599298	8.863524	-0.26423	1.200991
6hsalt_leaf_SS	HSP90-03	27.8252	27.8252	28.45764	0.365141	28.03602	25.68489	2.35113	5.039092	-2.68796	6.444026
6hsalt_stem_SS	HSP90-03	27.85415	27.85415	28.61135	0.437172	28.10655	25.09223	3.01432	8.863524	-5.8492	57.64821
1hsalt_leaf_SS	HSP90-04	27.6488	27.56945	28.29785	0.399609	27.8387	22.00433	5.834364	3.029978	2.804385	0.143151
1hsalt_stem_SS	HSP90-04	33.64226	35.37273	35.82899	1.153579	34.94799	24.23562	10.71238	7.234384	3.477991	0.089747
24hsalt_leaf_SS	HSP90-04	27.79851	28.95956	29.30918	0.790819	28.68908	23.29518	5.393901	3.029978	2.363923	0.194262
24hsalt_stem_SS	HSP90-04	26.21585	28.01458	27.90528	1.008427	27.37857	22.35948	5.019091	7.234384	-2.21529	4.64376
6hsalt_leaf_SS	HSP90-04	34.31919	34.64957	36.22718	1.01968	35.06531	25.68489	9.380427	3.029978	6.350449	0.012255
6hsalt_stem_SS	HSP90-04	33.12806	Undetermined	32.93009	0.139986	33.02908	25.09223	7.936844	7.234384	0.70246	0.614523
1hsalt_leaf_SS	HSP90-05	Undetermined	34.68974	36.94445	1.594316	35.8171	22.00433	13.81276	2.201825	11.61094	0.00032
1hsalt_stem_SS	HSP90-05	31.55195	30.9627	33.95286	1.58391	32.15584	24.23562	7.920222	6.704037	1.216185	0.430419
24hsalt_leaf_SS	HSP90-05	29.22626	29.40761	29.60961	0.191768	29.41449	23.29518	6.11931	2.201825	3.917485	0.066179
24hsalt_stem_SS	HSP90-05	22.32156	21.7606	22.40939	0.351978	22.16385	22.35948	-0.19563	6.704037	-6.89967	119.4005
6hsalt_leaf_SS	HSP90-05	25.25638	24.67578	25.10325	0.30091	25.0118	25.68489	-0.67308	2.201825	-2.87491	7.33557
6hsalt_stem_SS	HSP90-05	29.74529	29.95771	28.93299	0.540834	29.54533	25.09223	4.4531	6.704037	-2.25094	4.759918
1hsalt_leaf_SS	HSP90-09	34.74693	34.63768	35.01113	0.192008	34.79858	22.00433	12.79425	9.670187	3.124059	0.1147
1hsalt_stem_SS	HSP90-09	34.74134	34.5789	34.4329	0.154295	34.58438	24.23562	10.34877	11.38556	-1.03679	2.051661
24hsalt_leaf_SS	HSP90-09	36.14188	35.47031	34.95631	0.59453	35.52283	23.29518	12.22765	9.670187	2.557461	0.169874
24hsalt_stem_SS	HSP90-09	29.74529	29.95771	28.93299	0.540834	29.54533	22.35948	7.185853	11.38556	-4.19971	18.37543
6hsalt_leaf_SS	HSP90-09	35.92656	36.6802	35.33402	0.674698	35.98026	25.68489	10.29537	9.670187	0.625188	0.648335
6hsalt_stem_SS	HSP90-09	30.44081	31.51466	30.07712	0.747437	30.67753	25.09223	5.585298	11.38556	-5.80026	55.72533
1hsalt_leaf_SS	sHSP-02	27.8507	27.84496	27.68275	0.09535	27.7928	22.00433	5.788467	7.727236	-1.93877	3.833783
1hsalt_stem_SS	sHSP-02	27.91368	27.91806	27.80031	0.066753	27.87735	24.23562	3.64173	10.22697	-6.58524	96.0182
24hsalt_leaf_SS	sHSP-02	29.96903	29.59254	29.86808	0.194875	29.80988	23.29518	6.514699	7.727236	-1.21254	2.317448
24hsalt_stem_SS	sHSP-02	27.43804	27.67162	27.42441	0.138955	27.51136	22.35948	5.151879	10.22697	-5.07509	33.70959
6hsalt_leaf_SS	sHSP-02	26.06545	25.57944	25.59727	0.2756	25.74739	25.68489	0.0625	7.727236	-7.66474	202.9156
6hsalt_stem_SS	sHSP-02	29.72495	29.72495	28.61203	0.642543	29.35397	25.09223	4.261742	10.22697	-5.96522	62.47575
1hsalt_leaf_SS	sHSP-07	30.59342	30.59342	31.23585	0.370903	30.80756	22.00433	8.803231	10.98343	-2.1802	4.532171
1hsalt_stem_SS	sHSP-07	32.72771	32.72771	33.2585	0.306453	32.90464	24.23562	8.669025	14.19922	-5.53019	46.21186
24hsalt_leaf_SS	sHSP-07	34.96949	34.96949	34.99641	0.015542	34.97847	23.29518	11.68329	10.98343	0.699852	0.615635
24hsalt_stem_SS	sHSP-07	Undetermined	25.99571	26.39964	0.285617	26.19767	22.35948	3.838196	14.19922	-10.361	1315.159

6hsalt_leaf_SS	sHSP-07	36.57129	36.57129	35.98951	0.335889	36.37736	25.68489	10.69248	10.98343	-0.29095	1.223449
6hsalt_stem_SS	sHSP-07	31.40219	31.40219	31.34025	0.035758	31.38154	25.09223	6.289311	14.19922	-7.90991	240.502
1hsalt_leaf_SS	sHSP-08	25.18256	25.45622	25.64271	0.231448	25.42716	22.00433	3.422826	6.28698	-2.86415	7.281088
1hsalt_stem_SS	sHSP-08	22.06608	22.41697	22.47828	0.222406	22.32045	24.23562	-1.91517	5.813431	-7.7286	212.1002
24hsalt_leaf_SS	sHSP-08	26.04772	26.81858	24.87534	0.97851	25.91388	23.29518	2.618695	6.28698	-3.66829	12.71346
24hsalt_stem_SS	sHSP-08	29.67634	29.93033	29.40471	0.262857	29.67046	22.35948	7.310979	5.813431	1.497548	0.354155
6hsalt_leaf_SS	sHSP-08	28.3762	28.46162	28.93995	0.303837	28.59259	25.68489	2.907704	6.28698	-3.37928	10.40551
6hsalt_stem_SS	sHSP-08	29.95436	29.87677	30.04578	0.084595	29.95897	25.09223	4.86674	5.813431	-0.94669	1.927448
1hsalt_leaf_SS	sHSP-11	27.53911	27.7411	27.40966	0.167036	27.56329	22.00433	5.558955	4.291369	1.267586	0.415354
1hsalt_stem_SS	sHSP-11	32.48546	32.48546	32.9318	0.257693	32.63424	24.23562	8.398623	4.853923	3.544701	0.085692
24hsalt_leaf_SS	sHSP-11	26.83395	26.83395	27.43641	0.34783	27.03477	23.29518	3.739591	4.291369	-0.55178	1.46589
24hsalt_stem_SS	sHSP-11	27.76479	27.76479	28.28676	0.301362	27.93878	22.35948	5.579299	4.853923	0.725377	0.604839
6hsalt_leaf_SS	sHSP-11	Undetermined	35.2874	34.12079	0.824921	34.70409	25.68489	9.019207	4.291369	4.727839	0.037738
6hsalt_stem_SS	sHSP-11	28.79851	28.95956	29.30918	0.261069	29.02242	25.09223	3.930186	4.853923	-0.92374	1.897022
1hsalt_leaf_SS	sHSP-15	28.32365	28.96292	28.90736	0.354132	28.73131	22.00433	6.726976	3.973532	2.753445	0.148296
1hsalt_stem_SS	sHSP-15	29.7912	29.51664	29.22453	0.283383	29.51079	24.23562	5.275174	7.387156	-2.11198	4.32285
24hsalt_leaf_SS	sHSP-15	30.31169	29.18178	28.96123	0.724462	29.4849	23.29518	6.189717	3.973532	2.216185	0.21521
24hsalt_stem_SS	sHSP-15	35.92656	36.6802	35.33402	0.674698	35.98026	22.35948	13.62078	7.387156	6.233626	0.013289
6hsalt_leaf_SS	sHSP-15	23.85489	24.65246	24.65093	0.460037	24.38609	25.68489	-1.2988	3.973532	-5.27233	38.64816
6hsalt_stem_SS	sHSP-15	28.17207	28.48935	27.85811	0.315621	28.17318	25.09223	3.080946	7.387156	-4.30621	19.78328
1hsalt_leaf_SS	sHSP-25	28.76231	28.55668	28.30115	0.231033	28.54005	22.00433	6.535713	7.119162	-0.58345	1.498427
1hsalt_stem_SS	sHSP-25	22.46122	22.25774	22.48682	0.125521	22.40192	24.23562	-1.83369	5.10987	-6.94356	123.0893
24hsalt_leaf_SS	sHSP-25	26.74545	26.51623	26.85495	0.172852	26.70554	23.29518	3.410361	7.119162	-3.7088	13.07556
24hsalt_stem_SS	sHSP-25	26.34935	26.00218	28.30709	1.242704	26.88621	22.35948	4.526729	11.28181	-6.75508	108.0141
6hsalt_leaf_SS	sHSP-25	26.20616	26.34653	26.12397	0.112543	26.22555	25.68489	0.540668	-2.4587	2.999368	0.125055
6hsalt_stem_SS	sHSP-25	26.09673	26.03325	26.59786	0.309288	26.24261	25.09223	1.150382	2.00793	-0.85755	1.811957
1hsalt_leaf_SS	sHSP-26	26.89708	26.73473	26.9209	0.101309	26.8509	22.00433	4.846571	4.7897	0.056871	0.961347
1hsalt_stem_SS	sHSP-26	26.00511	26.47907	26.59614	0.31296	26.36011	24.23562	2.124494	7.8581	-5.73361	53.20928
24hsalt_leaf_SS	sHSP-26	26.99183	26.4292	26.71834	0.28135	26.71312	23.29518	3.417939	4.7897	-1.37176	2.587863
24hsalt_stem_SS	sHSP-26	26.55728	26.98822	27.18542	0.321237	26.91031	22.35948	4.55083	4.1	0.45083	0.731622
6hsalt_leaf_SS	sHSP-26	34.56396	34.55016	36.68255	1.22717	35.26555	25.68489	9.580668	4.7897	4.790968	0.036122

6hsalt_stem_SS	sHSP-26	30.46435	30.23656	Undetermined	0.161068	30.35046	25.09223	5.258225	10.522	-5.26378	38.41972
1hsalt_leaf_SS	sHSP-27	28.13186	28.39304	27.68072	0.360356	28.06854	22.00433	6.064204	5.15657	0.907634	0.533058
1hsalt_stem_SS	sHSP-27	23.68166	22.90297	23.30608	0.389426	23.2969	24.23562	-0.93871	7.780998	-8.71971	421.5937
24hsalt_leaf_SS	sHSP-27	26.26523	27.07989	27.48732	0.622251	26.94415	23.29518	3.648963	5.15657	-1.50761	2.84338
24hsalt_stem_SS	sHSP-27	24.34508	24.47895	24.85045	0.261834	24.55816	22.35948	2.19868	7.780998	-5.58232	47.91207
6hsalt_leaf_SS	sHSP-27	23.85489	24.65246	24.65093	0.460037	24.38609	25.68489	-1.2988	2.8156	-4.1144	17.32035
6hsalt_stem_SS	sHSP-27	24.70468	25.5364	26.07283	0.689367	25.43797	25.09223	0.345742	7.780998	-7.43526	173.0752
1hsalt_leaf_SS	sHSP-30	26.89708	26.73473	26.9209	0.101309	26.8509	22.00433	4.846571	4.7897	0.056871	0.961347
1hsalt_stem_SS	sHSP-30	28.51739	28.80398	28.8155	0.168884	28.71229	24.23562	4.476673	2.524207	1.952465	0.258374
24hsalt_leaf_SS	sHSP-30	26.50151	26.98842	27.14805	0.336792	26.87932	23.29518	3.58414	6.190665	-2.60653	6.090351
24hsalt_stem_SS	sHSP-30	26.71159	26.95373	27.05001	0.174368	26.90511	22.35948	4.545631	2.524207	2.021424	0.246315
6hsalt_leaf_SS	sHSP-30	30.94935	30.98024	30.80831	0.091655	30.91263	25.68489	5.227748	6.190665	-0.96292	1.949247
6hsalt_stem_SS	sHSP-30	28.83676	29.08653	28.85088	0.140307	28.92472	25.09223	3.832492	6.190665	-2.35817	5.127209
**Tolerant cultivar in response to cold stress**											
**SAMPLE**	**DETECTOR**	**Ct1**	**Ct2**	**Ct3**	**sd**	**Avg Ct**	**End CTl**	**dCt**	**CTL0h**	**ddCt**	**Log Ct**
1hcold_leaf_ST	ACT	23.68341	23.78959	23.68633	0.060478	23.71978	23.71978	0		0	1
1hcold_stem_ST	ACT	26.39295	25.11949	25.67454	0.638473	25.72899	25.72899	0		0	1
24hcold_leaf_ST	ACT	23.81052	23.9396	23.95865	0.080587	23.90293	23.90293	0		0	1
24hcold_stem_ST	ACT	25.79201	25.57194	26.02629	0.227214	25.79675	25.79675	0		0	1
6hcold_leaf_ST	ACT	22.32156	21.7606	22.40939	0.351978	22.16385	22.16385	0		0	1
6hcold_stem_ST	ACT	25.76866	25.43453	25.75672	0.189554	25.6533	25.6533	0		0	1
1hcold_leaf_ST	HSP100-07	25.93585	25.68763	26.07311	0.195385	25.89886	23.71978	2.179085	-3.76111	5.940197	0.016286
1hcold_stem_ST	HSP100-07	27.92614	28.25298	27.50412	0.375435	27.89441	25.72899	2.165421	-8.93484	11.10026	0.000455
24hcold_leaf_ST	HSP100-07	26.68342	26.59494	26.92233	0.169354	26.73356	23.90293	2.830638	-3.76111	6.591751	0.010368
24hcold_stem_ST	HSP100-07	27.34448	27.4033	27.28838	0.057464	27.34539	25.79675	1.548637	8.934842	-7.38621	167.2897
6hcold_leaf_ST	HSP100-07	27.24379	27.22682	26.99248	0.140454	27.15436	22.16385	4.990513	-3.76111	8.751625	0.00232
6hcold_stem_ST	HSP100-07	28.76809	28.7286	29.04026	0.169692	28.84565	25.6533	3.192344	8.934842	-5.7425	53.53827
1hcold_leaf_ST	HSP100-08	22.44834	22.76286	23.14733	0.350075	22.78618	23.71978	-0.9336	-6.41278	5.479177	0.022418
1hcold_stem_ST	HSP100-08	26.34179	26.47849	26.47047	0.076711	26.43025	25.72899	0.701259	5.156522	-4.45526	21.93652
24hcold_leaf_ST	HSP100-08	28.71092	28.50418	28.77507	0.141565	28.66339	23.90293	4.760464	-6.41278	11.17324	0.000433

24hcold_stem_ST	HSP100-08	29.96928	30.20781	30.58065	0.308133	30.25258	25.79675	4.45583	5.156522	-0.70069	1.625285
6hcold_leaf_ST	HSP100-08	31.59303	31.95246	31.79421	0.180142	31.7799	22.16385	9.616051	-6.41278	16.02883	1.5E-05
6hcold_stem_ST	HSP100-08	31.15208	31.42894	31.10223	0.176011	31.22775	25.6533	5.574449	5.156522	0.417927	0.748499
1hcold_leaf_ST	HSP100-11	29.38926	29.783	29.26519	0.270353	29.47915	23.71978	5.759372	0.939984	4.819388	0.035418
1hcold_stem_ST	HSP100-11	31.66207	31.37707	32.67173	0.680291	31.90362	25.72899	6.174632	12.08982	-5.91519	60.34619
24hcold_leaf_ST	HSP100-11	27.57463	27.39433	27.30765	0.136194	27.42554	23.90293	3.522611	0.939984	2.582628	0.166937
24hcold_stem_ST	HSP100-11	31.05093	31.31459	31.10168	0.139899	31.15573	25.79675	5.358984	12.08982	-6.73084	106.2147
6hcold_leaf_ST	HSP100-11	32.8975	32.26675	32.40186	0.332108	32.52204	22.16385	10.35819	0.939984	9.418201	0.001462
6hcold_stem_ST	HSP100-11	29.40965	29.93574	30.32276	0.458319	29.88938	25.6533	4.236078	12.08982	-7.85374	231.3197
1hcold_leaf_ST	HSP100-12	33.38369	33.07406	33.673	0.299525	33.37692	23.71978	9.657141	0.37819	9.278951	0.00161
1hcold_stem_ST	HSP100-12	35.89113	Undetermined	35.88337	0.005486	35.88725	25.72899	10.15826	11.52094	-1.36268	2.571621
24hcold_leaf_ST	HSP100-12	28.17207	28.48935	27.85811	0.315621	28.17318	23.90293	4.270252	0.37819	3.892062	0.067355
24hcold_stem_ST	HSP100-12	27.24379	27.22682	26.99248	0.140454	27.15436	25.79675	1.357614	5.209399	-3.85178	14.43785
6hcold_leaf_ST	HSP100-12	28.76809	28.7286	29.04026	0.169692	28.84565	22.16385	6.681797	0.37819	6.303607	0.01266
6hcold_stem_ST	HSP100-12	28.71092	28.50418	28.77507	0.141565	28.66339	25.6533	3.010086	11.52094	-8.51085	364.7725
1hcold_leaf_ST	HSP100-16	29.96928	30.20781	30.58065	0.308133	30.25258	23.71978	6.532802	-3.05802	9.590818	0.001297
1hcold_stem_ST	HSP100-16	31.62919	33.3103	32.18398	0.856596	32.37449	25.72899	6.645497	8.364046	-1.71855	3.291052
24hcold_leaf_ST	HSP100-16	30.6584	30.89862	31.59553	0.486758	31.05085	23.90293	7.147925	-3.05802	10.20594	0.000847
24hcold_stem_ST	HSP100-16	31.62919	33.3103	32.18398	0.856596	32.37449	25.79675	6.57774	8.364046	-1.78631	3.449306
6hcold_leaf_ST	HSP100-16	26.81703	25.78814	26.38122	0.516443	26.3288	22.16385	4.164947	-3.05802	7.222963	0.006694
6hcold_stem_ST	HSP100-16	26.37807	25.54799	25.6908	0.443804	25.87229	25.6533	0.218985	8.364046	-8.14506	283.0789
1hcold_leaf_ST	HSP100-18	24.02629	24.02266	22.68998	0.770474	23.57965	23.71978	-0.14013	-0.04711	-0.09302	1.066604
1hcold_stem_ST	HSP100-18	29.0272	28.24851	29.26001	0.529728	28.84524	25.72899	3.116247	8.121113	-5.00487	32.10811
24hcold_leaf_ST	HSP100-18	33.47397	33.15795	33.0517	0.219647	33.22787	23.90293	9.324948	-0.04711	9.372055	0.001509
24hcold_stem_ST	HSP100-18	37.0333	32.88817	32.36753	2.556774	34.09633	25.79675	8.29958	8.121113	0.178468	0.883641
6hcold_leaf_ST	HSP100-18	31.52907	32.25711	31.7521	0.37301	31.84609	22.16385	9.682243	-0.04711	9.729351	0.001178
6hcold_stem_ST	HSP100-18	31.39899	Undetermined	31.07623	0.228229	31.23761	25.6533	5.584304	8.121113	-2.53681	5.803038
1hcold_leaf_ST	HSP100-20	27.24379	27.22682	26.99248	0.140454	27.15436	23.71978	3.434587	-3.50825	6.942838	0.008128
1hcold_stem_ST	HSP100-20	28.76809	28.7286	29.04026	0.169692	28.84565	25.72899	3.116656	2.393056	0.7236	0.605584
24hcold_leaf_ST	HSP100-20	28.84734	28.75181	28.80392	0.047833	28.80102	23.90293	4.898098	-3.50825	8.406349	0.002947
24hcold_stem_ST	HSP100-20	29.66451	29.93796	30.52295	0.438537	30.04181	25.79675	4.245059	2.393056	1.852003	0.277007

6hcold_leaf_ST	HSP100-20	28.79851	28.95956	29.30918	0.261069	29.02242	22.16385	6.858567	-3.50825	10.36682	0.000757
6hcold_stem_ST	HSP100-20	28.32365	28.96292	28.90736	0.354132	28.73131	25.6533	3.078007	2.393056	0.684951	0.622027
1hcold_leaf_ST	HSP60-02	29.7912	29.51664	29.22453	0.283383	29.51079	23.71978	5.791013	-4.85859	10.64961	0.000623
1hcold_stem_ST	HSP60-02	30.31169	29.18178	28.96123	0.724462	29.4849	25.72899	3.755908	3.028418	0.72749	0.603954
24hcold_leaf_ST	HSP60-02	30.17745	30.55206	30.54793	0.215099	30.42582	23.90293	6.52289	-4.85859	11.38148	0.000375
24hcold_stem_ST	HSP60-02	33.93066	Undetermined	33.08021	0.601363	33.50544	25.79675	7.708687	3.028418	4.680269	0.039003
6hcold_leaf_ST	HSP60-02	34.80298	35.17245	35.83423	0.522484	35.26989	22.16385	13.10604	-4.85859	17.96463	3.91E-06
6hcold_stem_ST	HSP60-02	Undetermined	25.99571	26.39964	0.285617	26.19767	25.6533	0.54437	3.028418	-2.48405	5.594651
1hcold_leaf_ST	HSP60-03	36.57129	36.57129	35.98951	0.335889	36.37736	23.71978	12.65759	4.814743	7.842845	0.004356
1hcold_stem_ST	HSP60-03	31.40219	31.40219	31.34025	0.035758	31.38154	25.72899	5.652551	13.10722	-7.45467	175.4197
24hcold_leaf_ST	HSP60-03	Undetermined	25.93134	25.91768	0.009653	25.92451	23.90293	2.021584	4.814743	-2.79316	6.93146
24hcold_stem_ST	HSP60-03	32.64065	32.64065	33.83352	0.688705	33.03827	25.79675	7.241523	13.10722	-5.86569	58.31091
6hcold_leaf_ST	HSP60-03	33.07114	33.07114	35.19228	1.224643	33.77818	22.16385	11.61433	4.814743	6.799591	0.008977
6hcold_stem_ST	HSP60-03	26.38348	26.11682	27.77058	0.887885	26.75696	25.6533	1.103656	7.217496	-6.11384	69.25468
1hcold_leaf_ST	HSP60-05	28.50637	28.46013	28.55793	0.048924	28.50814	23.71978	4.788366	2.073274	2.715092	0.152292
1hcold_stem_ST	HSP60-05	25.44403	26.54164	26.11581	0.553378	26.03383	25.72899	0.304835	-11.46	11.7648	0.000287
24hcold_leaf_ST	HSP60-05	27.49907	27.9112	28.01439	0.272657	27.80822	23.90293	3.905293	2.073274	1.832019	0.280871
24hcold_stem_ST	HSP60-05	26.02278	25.63796	26.50707	0.4355	26.05594	25.79675	0.259189	7.521639	-7.26245	153.5378
6hcold_leaf_ST	HSP60-05	32.64065	33.83352	33.54919	0.623062	33.34112	22.16385	11.17727	2.073274	9.103997	0.001817
6hcold_stem_ST	HSP60-05	33.07114	35.19228	33.13796	1.205816	33.80046	25.6533	8.147155	11.45997	-3.31281	9.937007
1hcold_leaf_ST	HSP60-09	27.11608	27.42363	27.76134	0.322747	27.43368	23.71978	3.713908	-0.14832	3.862229	0.068763
1hcold_stem_ST	HSP60-09	25.66739	25.617	25.69202	0.038241	25.6588	25.72899	-0.07019	8.451698	-8.52189	367.5726
24hcold_leaf_ST	HSP60-09	27.87881	27.63464	27.93376	0.159224	27.81573	23.90293	3.912808	-0.14832	4.061129	0.059907
24hcold_stem_ST	HSP60-09	28.80105	25.64463	25.69985	1.806632	26.71518	25.79675	0.918426	8.451698	-7.53327	185.2425
6hcold_leaf_ST	HSP60-09	26.81319	24.61252	24.93532	1.188385	25.45368	22.16385	3.28983	-0.14832	3.438151	0.09226
6hcold_stem_ST	HSP60-09	30.70225	30.44643	31.16147	0.362309	30.77005	25.6533	5.116749	8.451698	-3.33495	10.09066
1hcold_leaf_ST	HSP60-13	31.90872	31.94539	34.23858	1.334682	32.69756	23.71978	8.977786	-1.11553	10.09332	0.000915
1hcold_stem_ST	HSP60-13	26.10387	25.97194	25.37901	0.386087	25.81827	25.72899	0.08928	6.638692	-6.54941	93.66332
24hcold_leaf_ST	HSP60-13	25.90794	25.89382	25.93467	0.020747	25.91214	23.90293	2.009215	-1.11553	3.12475	0.114645
24hcold_stem_ST	HSP60-13	24.59753	24.03336	24.76292	0.382512	24.4646	25.79675	-1.33215	6.638692	-7.97084	250.8773
6hcold_leaf_ST	HSP60-13	23.89199	24.13788	24.24441	0.180743	24.09143	22.16385	1.927575	-1.11553	3.043109	0.12132

6hcold_stem_ST	HSP60-13	34.04357	32.84406	33.85418	0.644859	33.5806	25.6533	7.927299	6.638692	1.288607	0.409346
1hcold_leaf_ST	HSP60-14	27.7644	27.91184	27.82387	0.074179	27.83337	23.71978	4.113592	1.098698	3.014894	0.123716
1hcold_stem_ST	HSP60-14	25.66633	26.03157	26.28833	0.312576	25.99541	25.72899	0.266417	12.2208	-11.9544	3968.52
24hcold_leaf_ST	HSP60-14	26.60755	26.34211	26.3114	0.162844	26.42036	23.90293	2.51743	1.098698	1.418732	0.374041
24hcold_stem_ST	HSP60-14	27.8507	27.84496	27.68275	0.09535	27.7928	25.79675	1.996053	6.220803	-4.22475	18.6972
6hcold_leaf_ST	HSP60-14	25.67533	25.76434	26.04864	0.194984	25.82944	22.16385	3.665586	1.098698	2.566888	0.168768
6hcold_stem_ST	HSP60-14	27.14226	27.35905	27.11025	0.135353	27.20385	25.6533	1.550548	1.220803	0.329745	0.795677
1hcold_leaf_ST	HSP60-16	27.50985	27.62151	27.58974	0.057531	27.5737	23.71978	3.853925	-2.73807	6.59199	0.010366
1hcold_stem_ST	HSP60-16	26.95381	27.4293	27.53964	0.311304	27.30758	25.72899	1.578592	6.27347	-4.69488	25.89997
24hcold_leaf_ST	HSP60-16	25.60762	26.00605	25.67696	0.212864	25.76354	23.90293	1.860616	-2.73807	4.598681	0.041272
24hcold_stem_ST	HSP60-16	26.83087	27.30869	26.42916	0.440314	26.85624	25.79675	1.05949	6.27347	-5.21398	37.11627
6hcold_leaf_ST	HSP60-16	27.91368	27.91806	27.80031	0.066753	27.87735	22.16385	5.713496	-2.73807	8.451561	0.002856
6hcold_stem_ST	HSP60-16	25.9289	26.65245	27.32181	0.696632	26.63439	25.6533	0.981084	6.27347	-5.29239	39.18926
1hcold_leaf_ST	HSP60-20	34.5104	34.5104	33.96862	0.312795	34.32981	23.71978	10.61003	-4.0606	14.67063	3.83E-05
1hcold_stem_ST	HSP60-20	32.29041	32.29041	32.45801	0.096766	32.34627	25.72899	6.617281	5.911655	0.705627	0.613176
24hcold_leaf_ST	HSP60-20	29.86441	29.86441	30.76761	0.521462	30.16547	23.90293	6.262549	-4.0606	10.32315	0.000781
24hcold_stem_ST	HSP60-20	34.30904	34.30904	34.45161	0.082313	34.35657	25.79675	8.559819	5.911655	2.648164	0.159523
6hcold_leaf_ST	HSP60-20	32.80377	32.80377	34.83309	1.171627	33.48021	22.16385	11.31636	-4.0606	15.37696	2.35E-05
6hcold_stem_ST	HSP60-20	33.56814	33.56814	33.97659	0.235815	33.70429	25.6533	8.050986	5.911655	2.139332	0.226985
1hcold_leaf_ST	HSP90-02	26.29036	24.9731	25.3395	0.679898	25.53432	23.71978	1.814542	-2.88662	4.701158	0.038442
1hcold_stem_ST	HSP90-02	31.90872	31.90872	31.94539	0.02117	31.92094	25.72899	6.191953	7.069286	-0.87733	1.836976
24hcold_leaf_ST	HSP90-02	Undetermined	27.21439	27.29408	0.056354	27.25423	23.90293	3.351309	-2.88662	6.237925	0.013249
24hcold_stem_ST	HSP90-02	30.21587	30.21587	30.60879	0.226852	30.34684	25.79675	4.550094	7.069286	-2.51919	5.732609
6hcold_leaf_ST	HSP90-02	34.89123	34.89123	34.71727	0.100432	34.83324	22.16385	12.66939	-2.88662	15.55601	2.08E-05
6hcold_stem_ST	HSP90-02	35.39623	35.39623	33.01996	1.37194	34.60414	25.6533	8.950837	7.069286	1.881551	0.271392
1hcold_leaf_ST	HSP90-03	28.89705	28.83	28.6982	0.101166	28.80842	23.71978	5.088642	-2.98657	8.075208	0.003708
1hcold_stem_ST	HSP90-03	26.3339	26.43114	26.65067	0.162271	26.47191	25.72899	0.742914	5.364267	-4.62135	24.61308
24hcold_leaf_ST	HSP90-03	24.70204	25.39839	24.84924	0.366999	24.98322	23.90293	1.080298	-2.98657	4.066864	0.059669
24hcold_stem_ST	HSP90-03	29.96903	29.59254	29.86808	0.194875	29.80988	25.79675	4.013133	5.364267	-1.35113	2.551127
6hcold_leaf_ST	HSP90-03	27.19694	28.01931	27.79539	0.42516	27.67054	22.16385	5.506694	-2.98657	8.49326	0.002775
6hcold_stem_ST	HSP90-03	27.76479	28.28676	26.72406	0.795569	27.59187	25.6533	1.938567	5.364267	-3.4257	10.7458

1hcold_leaf_ST	HSP90-04	28.02286	27.72693	27.89438	0.148394	27.88139	23.71978	4.161616	-4.40525	8.566865	0.002637
1hcold_stem_ST	HSP90-04	26.3399	25.99933	26.01366	0.192626	26.11763	25.72899	0.38864	7.291443	-6.9028	119.6605
24hcold_leaf_ST	HSP90-04	26.76016	26.55289	26.92813	0.187965	26.74706	23.90293	2.844134	-4.40525	7.249384	0.006572
24hcold_stem_ST	HSP90-04	27.43804	27.67162	27.42441	0.138955	27.51136	25.79675	1.714609	7.291443	-5.57683	47.73033
6hcold_leaf_ST	HSP90-04	25.76487	25.55446	25.82218	0.140965	25.71384	22.16385	3.549987	-4.40525	7.955237	0.004029
6hcold_stem_ST	HSP90-04	30.61218	30.76115	31.57297	0.5171	30.9821	25.6533	5.328798	7.291443	-1.96265	3.897759
1hcold_leaf_ST	HSP90-05	28.08497	28.29386	28.37523	0.149727	28.25135	23.71978	4.531577	-3.82942	8.360998	0.003042
1hcold_stem_ST	HSP90-05	26.01418	26.33528	26.78084	0.385012	26.37677	25.72899	0.647776	5.167391	-4.51962	22.93716
24hcold_leaf_ST	HSP90-05	26.79444	26.9893	26.68116	0.155858	26.82163	23.90293	2.918706	-3.82942	6.748128	0.009303
24hcold_stem_ST	HSP90-05	28.9126	28.76854	28.80368	0.075111	28.82827	25.79675	3.031524	5.167391	-2.13587	4.395011
6hcold_leaf_ST	HSP90-05	27.15392	27.28213	27.36461	0.106167	27.26689	22.16385	5.103038	-3.82942	8.932459	0.002047
6hcold_stem_ST	HSP90-05	25.84046	26.03082	25.97714	0.098147	25.94948	25.6533	0.296172	5.167391	-4.87122	29.26733
1hcold_leaf_ST	HSP90-09	30.1839	30.02603	30.83975	0.431507	30.34989	23.71978	6.630116	-1.78163	8.411745	0.002936
1hcold_stem_ST	HSP90-09	27.39258	28.66847	28.00777	0.63808	28.02294	25.72899	2.293951	7.564647	-5.2707	38.60447
24hcold_leaf_ST	HSP90-09	24.02482	24.74506	24.76732	0.422406	24.5124	23.90293	0.609475	-1.78163	2.391104	0.190636
24hcold_stem_ST	HSP90-09	31.92718	31.77396	31.8087	0.080333	31.83661	25.79675	6.039865	13.56465	-7.52478	184.1557
6hcold_leaf_ST	HSP90-09	29.0695	29.25564	28.87839	0.188632	29.06784	22.16385	6.903994	-1.78163	8.685623	0.002429
6hcold_stem_ST	HSP90-09	25.82601	25.74098	25.85561	0.059507	25.80753	25.6533	0.154229	1.646467	-1.49224	2.81325
1hcold_leaf_ST	sHSP-02	28.49542	29.215	29.1701	0.403117	28.96017	23.71978	5.240396	-4.92564	10.16604	0.00087
1hcold_stem_ST	sHSP-02	26.11759	26.72544	26.92969	0.422437	26.59091	25.72899	0.861916	8.619916	-7.758	216.4665
24hcold_leaf_ST	sHSP-02	25.7297	25.379	26.53911	0.594976	25.8826	23.90293	1.979677	-4.92564	6.905321	0.008342
24hcold_stem_ST	sHSP-02	30.83554	31.08101	30.92369	0.124346	30.94674	25.79675	5.149996	8.619916	-3.46992	11.08026
6hcold_leaf_ST	sHSP-02	28.58673	29.16892	29.71123	0.562368	29.15563	22.16385	6.991778	-4.92564	11.91742	0.000259
6hcold_stem_ST	sHSP-02	25.99571	26.39964	25.87569	0.274493	26.09035	25.6533	0.437042	8.619916	-8.18287	290.5965
1hcold_leaf_ST	sHSP-07	29.72495	28.61203	28.47634	0.685082	28.93777	23.71978	5.217993	3.379002	1.838992	0.279517
1hcold_stem_ST	sHSP-07	25.19537	25.44823	25.97802	0.39941	25.54054	25.72899	-0.18845	8.746584	-8.93504	489.4559
24hcold_leaf_ST	sHSP-07	25.84046	26.03082	25.97714	0.098147	25.94948	23.90293	2.04655	3.379002	-1.33245	2.518303
24hcold_stem_ST	sHSP-07	25.93134	25.91768	25.87404	0.02993	25.90768	25.79675	0.110936	8.746584	-8.63565	397.7307
6hcold_leaf_ST	sHSP-07	27.52328	28.93527	26.60216	1.175127	27.6869	22.16385	5.523052	3.379002	2.14405	0.226244
6hcold_stem_ST	sHSP-07	30.61218	30.76115	31.57297	0.5171	30.9821	25.6533	5.328798	8.746584	-3.41779	10.68701
1hcold_leaf_ST	sHSP-08	31.01076	30.9068	31.41459	0.268247	31.11071	23.71978	7.390937	0.777854	6.613083	0.010216

1hcold_stem_ST	sHSP-08	27.8252	28.45764	26.83211	0.819408	27.70499	25.72899	1.975994	2.017716	-0.04172	1.029342
24hcold_leaf_ST	sHSP-08	27.85415	28.61135	26.83446	0.891672	27.76665	23.90293	3.863729	0.777854	3.085875	0.117777
24hcold_stem_ST	sHSP-08	25.93134	25.91768	25.87404	0.02993	25.90768	25.79675	0.110936	2.017716	-1.90678	3.749712
6hcold_leaf_ST	sHSP-08	32.64065	33.83352	33.54919	0.623062	33.34112	22.16385	11.17727	0.777854	10.39942	0.00074
6hcold_stem_ST	sHSP-08	33.07114	35.19228	33.13796	1.205816	33.80046	25.6533	8.147155	2.017716	6.12944	0.014284
1hcold_leaf_ST	sHSP-11	28.05428	28.14797	28.44382	0.203327	28.21536	23.71978	4.495579	0.844496	3.651083	0.0796
1hcold_stem_ST	sHSP-11	26.31527	26.31389	26.39479	0.046316	26.34131	25.72899	0.612323	1.036997	-0.42467	1.342269
24hcold_leaf_ST	sHSP-11	24.64231	24.23411	24.53282	0.211282	24.46975	23.90293	0.566823	0.844496	-0.27767	1.212237
24hcold_stem_ST	sHSP-11	25.70923	25.72526	25.92078	0.117786	25.78509	25.79675	-0.01166	6.996667	-7.00833	128.7408
6hcold_leaf_ST	sHSP-11	23.62955	23.91438	23.85913	0.151047	23.80102	22.16385	1.637167	0.844496	0.792671	0.577274
6hcold_stem_ST	sHSP-11	28.85445	28.45657	28.58456	0.203112	28.63186	25.6533	2.978556	8.149632	-5.17108	36.02874
1hcold_leaf_ST	sHSP-15	28.3762	28.46162	28.93995	0.303837	28.59259	23.71978	4.872813	-4.23746	9.110269	0.001809
1hcold_stem_ST	sHSP-15	26.16178	26.39729	26.82831	0.338013	26.46246	25.72899	0.733469	4.366667	-3.6332	12.40799
24hcold_leaf_ST	sHSP-15	24.58182	24.92205	25.40308	0.412639	24.96898	23.90293	1.066059	-4.23746	5.303515	0.025321
24hcold_stem_ST	sHSP-15	29.54688	29.59245	29.52037	0.036457	29.55323	25.79675	3.756486	8.502637	-4.74615	26.83699
6hcold_leaf_ST	sHSP-15	27.67535	27.54604	27.33037	0.174278	27.51725	22.16385	5.353403	-4.23746	9.590859	0.001297
6hcold_stem_ST	sHSP-15	27.26491	28.91977	30.97824	1.860319	29.05431	25.6533	3.401002	11.85026	-8.44926	349.5274
1hcold_leaf_ST	sHSP-25	29.95436	29.87677	30.04578	0.084595	29.95897	23.71978	6.239194	-0.9588	7.197998	0.006811
1hcold_stem_ST	sHSP-25	27.27498	26.59991	26.88701	0.338787	26.92063	25.72899	1.191641	5.921077	-4.72944	26.52785
24hcold_leaf_ST	sHSP-25	23.57761	23.33451	23.82261	0.244053	23.57824	23.90293	-0.32468	-0.9588	0.634124	0.644332
24hcold_stem_ST	sHSP-25	31.37121	30.71772	30.70908	0.37981	30.93267	25.79675	5.135919	13.59211	-8.45619	351.2096
6hcold_leaf_ST	sHSP-25	28.38702	28.34654	28.40567	0.03023	28.37974	22.16385	6.215892	-0.9588	7.174697	0.006922
6hcold_stem_ST	sHSP-25	30.9068	31.55379	31.41587	0.340779	31.29215	25.6533	5.638849	13.59211	-7.95326	247.8389
1hcold_leaf_ST	sHSP-26	27.53911	27.7411	27.40966	0.167036	27.56329	23.71978	3.843512	-2.74485	6.588364	0.010392
1hcold_stem_ST	sHSP-26	27.58104	27.67001	27.95358	0.194557	27.73488	25.72899	2.005885	7.276778	-5.27089	38.60973
24hcold_leaf_ST	sHSP-26	26.61405	27.3144	26.64956	0.394495	26.85933	23.90293	2.956408	-2.74485	5.70126	0.01922
24hcold_stem_ST	sHSP-26	27.23138	27.6442	27.04202	0.307927	27.30587	25.79675	1.509117	7.276778	-5.76766	54.48022
6hcold_leaf_ST	sHSP-26	25.81419	25.66905	25.67532	0.082049	25.71952	22.16385	3.555671	-2.74485	6.300523	0.012687
6hcold_stem_ST	sHSP-26	33.19505	34.90294	33.55143	0.900972	33.88314	25.6533	8.229834	7.276778	0.953056	0.516537
1hcold_leaf_ST	sHSP-27	27.93754	28.39068	28.5812	0.330636	28.30314	23.71978	4.583364	2.00304	2.580324	0.167203
1hcold_stem_ST	sHSP-27	25.76633	25.46566	26.38719	0.469947	25.87306	25.72899	0.144067	7.863667	-7.7196	210.7808

24hcold_leaf_ST	sHSP-27	29.01789	29.27894	29.99897	0.508119	29.43194	23.90293	5.529012	2.00304	3.525972	0.086811
24hcold_stem_ST	sHSP-27	27.74905	28.16279	28.43245	0.344221	28.11476	25.79675	2.318012	8.6667	-6.34869	81.49775
6hcold_leaf_ST	sHSP-27	21.8062	21.73986	22.06723	0.173063	21.8711	22.16385	-0.29275	2.00304	-2.29579	4.910242
6hcold_stem_ST	sHSP-27	28.70565	28.34646	28.73429	0.216119	28.59547	22.16385	2.318012	1.6667	0.651312	0.636701
1hcold_leaf_ST	sHSP-30	32.62406	33.91491	31.38751	1.263797	32.64216	23.71978	8.922384	-0.52198	9.444368	0.001435
1hcold_stem_ST	sHSP-30	33.66085	33.94127	34.02463	0.190578	33.87558	25.72899	8.146589	3.203407	4.943182	0.032505
24hcold_leaf_ST	sHSP-30	33.39828	33.531	33.84618	0.230063	33.59182	23.90293	9.688897	-0.52198	10.21088	0.000844
24hcold_stem_ST	sHSP-30	32.63204	32.75892	32.79751	0.086572	32.72949	25.79675	6.932742	3.203407	3.729335	0.075398
6hcold_leaf_ST	sHSP-30	33.05307	34.4377	32.96672	0.825475	33.48583	22.16385	11.32198	-0.52198	11.84397	0.000272
6hcold_stem_ST	sHSP-30	32.40395	33.03713	32.08045	0.486622	32.50718	25.6533	6.853872	3.203407	3.650465	0.079634
Suceptible cultivar in response to cold stress											
**SAMPLE**	**DETECTOR**	**Ct1**	**Ct2**	**Ct3**	**sd**	**Avg Ct**	**End CTl**	**dCt**	**CTL0h**	**ddCt**	**Log Ct**
1hcold_leaf_SS	ACT	21.79257	21.84413	21.897	0.052215	21.84457	21.84457	0		0	1
1hcold_stem_SS	ACT	24.35502	24.33128	24.23913	0.061217	24.30848	24.30848	0		0	1
24hcold_stem_SS	ACT	22.64505	22.88931	23.18418	0.269959	22.90618	22.90618	0		0	1
6hcold_leaf_SS	ACT	23.68166	22.90297	23.30608	0.389426	23.2969	23.2969	0		0	1
6hcold_stem_SS	ACT	21.43578	21.50107	21.44154	0.036145	21.45946	21.45946	0		0	1
1hcold_leaf_SS	HSP100-07	26.83866	27.1913	26.69828	0.25401	26.90941	21.84457	5.064845	4.256441	0.808404	0.571013
1hcold_stem_SS	HSP100-07	23.85489	24.65246	24.65093	0.460037	24.38609	24.30848	0.077614	1.113445	-1.03583	2.050294
24hcold_leaf_SS	HSP100-07	24.70468	25.5364	26.07283	0.689367	25.43797	24.30848	1.129498	4.256441	-3.12694	8.735823
24hcold_stem_SS	HSP100-07	26.89708	26.73473	26.9209	0.101309	26.8509	22.90618	3.944725	7.113136	-3.16841	8.990564
6hcold_leaf_SS	HSP100-07	28.51739	28.80398	28.8155	0.168884	28.71229	23.2969	5.415385	4.256441	1.158943	0.44784
6hcold_stem_SS	HSP100-07	26.50151	26.98842	27.14805	0.336792	26.87932	21.45946	5.419861	7.113136	-1.69328	3.233901
1hcold_leaf_SS	HSP100-08	26.71159	26.95373	27.05001	0.174368	26.90511	21.84457	5.060542	2.530401	2.530141	0.173122
1hcold_stem_SS	HSP100-08	30.94935	30.98024	30.80831	0.091655	30.91263	24.30848	6.604158	7.268533	-0.66438	1.584882
24hcold_leaf_SS	HSP100-08	28.83676	29.08653	28.85088	0.140307	28.92472	24.30848	4.616247	2.530401	2.085846	0.235558
24hcold_stem_SS	HSP100-08	32.41091	33.14599	33.48542	0.549261	33.0141	22.90618	10.10792	7.268533	2.839391	0.13972
6hcold_leaf_SS	HSP100-08	28.49288	28.32594	28.47364	0.091336	28.43082	23.2969	5.133917	2.530401	2.603517	0.164537
6hcold_stem_SS	HSP100-08	28.76764	28.84623	28.95328	0.093183	28.85572	21.45946	7.396255	7.268533	0.127722	0.915276
1hcold_leaf_SS	HSP100-11	29.23002	28.61839	28.75071	0.321801	28.86637	21.84457	7.021807	8.080248	-1.05844	2.082681

1hcold_stem_SS	HSP100-11	29.73103	30.95524	31.33834	0.839535	30.67487	24.30848	6.366392	10.47297	-4.10657	17.22669
24hcold_leaf_SS	HSP100-11	26.73174	26.95258	26.74114	0.124873	26.80849	24.30848	2.500012	8.080248	-5.58024	47.843
24hcold_stem_SS	HSP100-11	31.77611	31.79769	31.62458	0.094338	31.73279	22.90618	8.826613	10.47297	-1.64635	3.130412
6hcold_leaf_SS	HSP100-11	28.03131	31.71158	29.5749	1.848083	29.77259	23.2969	6.475691	8.080248	-1.60456	3.041025
6hcold_stem_SS	HSP100-11	30.85674	30.61984	31.05035	0.21562	30.84231	21.45946	9.38285	10.47297	-1.09012	2.128911
1hcold_leaf_SS	HSP100-12	33.28541	32.81591	33.34388	0.289426	33.1484	21.84457	11.30383	11.20091	0.102928	0.931141
1hcold_stem_SS	HSP100-12	21.8062	21.73986	22.06723	0.173063	21.8711	24.30848	-2.43738	1.255558	-3.69294	12.93257
24hcold_leaf_SS	HSP100-12	28.70565	28.34646	28.73429	0.216119	28.59547	24.30848	4.286993	4.19763	0.089363	0.939938
24hcold_stem_SS	HSP100-12	32.62406	33.91491	31.38751	1.263797	32.64216	22.90618	9.73598	3.9666	5.76938	0.018333
6hcold_leaf_SS	HSP100-12	33.66085	33.94127	34.02463	0.190578	33.87558	23.2969	10.57868	2.0091	8.569576	0.002632
6hcold_stem_SS	HSP100-12	26.68342	26.59494	26.92233	0.169354	26.73356	21.45946	5.274102	12.39666	-7.12255	139.3485
1hcold_leaf_SS	HSP100-16	29.0484	28.81562	28.75901	0.153373	28.87434	21.84457	7.029775	5.374351	1.655424	0.317444
1hcold_stem_SS	HSP100-16	24.02629	24.02266	22.68998	0.770474	23.57965	24.30848	-0.72883	3.33867	-4.0675	16.76639
24hcold_leaf_SS	HSP100-16	29.0272	28.24851	29.26001	0.529728	28.84524	24.30848	4.536763	5.374351	-0.83759	1.787059
24hcold_stem_SS	HSP100-16	32.69386	35.34673	34.83886	1.40811	34.29315	22.90618	11.38697	7.333867	4.053104	0.060241
6hcold_leaf_SS	HSP100-16	36.22703	35.44515	34.5129	0.858164	35.39503	23.2969	12.09812	5.374351	6.723771	0.009461
6hcold_stem_SS	HSP100-16	32.47515	32.08386	32.57673	0.26024	32.37858	21.45946	10.91912	7.333867	3.585251	0.083317
1hcold_leaf_SS	HSP100-18	28.03961	27.79799	28.3514	0.277443	28.063	21.84457	6.218431	5.008038	1.210393	0.432151
1hcold_stem_SS	HSP100-18	25.93585	25.68763	26.07311	0.195385	25.89886	24.30848	1.590386	7.268533	-5.67815	51.20269
24hcold_leaf_SS	HSP100-18	27.92614	28.25298	27.50412	0.375435	27.89441	24.30848	3.585937	5.008038	-1.4221	2.679755
24hcold_stem_SS	HSP100-18	26.68342	26.59494	26.92233	0.169354	26.73356	22.90618	3.827384	7.268533	-3.44115	10.86149
6hcold_leaf_SS	HSP100-18	27.34448	27.4033	27.28838	0.057464	27.34539	23.2969	4.048482	5.008038	-0.95956	1.944711
6hcold_stem_SS	HSP100-18	27.24379	27.22682	26.99248	0.140454	27.15436	21.45946	5.694901	2.68593	3.008971	0.124225
1hcold_leaf_SS	HSP100-20	25.70171	25.96931	25.635	0.176929	25.76867	21.84457	3.924105	9.563736	-5.63963	49.85377
1hcold_stem_SS	HSP100-20	26.82335	27.33385	28.4141	0.812205	27.52377	24.30848	3.215291	4.785678	-1.57039	2.969843
24hcold_leaf_SS	HSP100-20	32.87455	33.51234	32.39898	0.558646	32.92862	24.30848	8.620149	9.563736	-0.94359	1.923303
24hcold_stem_SS	HSP100-20	30.92519	32.27888	31.34027	0.69351	31.51478	22.90618	8.6086	4.785678	3.822922	0.070662
6hcold_leaf_SS	HSP100-20	30.06373	30.92839	31.31714	0.641587	30.76975	23.2969	7.472847	1.1087	6.364147	0.012139
6hcold_stem_SS	HSP100-20	31.97957	32.11514	32.6424	0.350172	32.2457	21.45946	10.78624	4.785678	6.00056	0.015619
1hcold_leaf_SS	HSP60-02	26.03436	26.13662	26.13504	0.058593	26.10201	21.84457	4.257438	5.960718	-1.70328	3.256404
1hcold_stem_SS	HSP60-02	26.42873	26.77429	27.4858	0.53899	26.89627	24.30848	2.587798	2.793125	-0.20533	1.152948

24hcold_leaf_SS	HSP60-02	26.26523	27.07989	27.48732	0.622251	26.94415	24.30848	2.63567	5.960718	-3.32505	10.02165
24hcold_stem_SS	HSP60-02	25.14053	25.23509	25.57603	0.229071	25.31722	22.90618	2.411036	2.794613	-0.38358	1.304572
6hcold_leaf_SS	HSP60-02	32.87455	33.51234	32.39898	0.558646	32.92862	23.2969	9.631721	5.960718	3.671003	0.078509
6hcold_stem_SS	HSP60-02	30.92519	32.27888	31.34027	0.69351	31.51478	21.45946	10.05532	10.27932	-0.224	1.167971
1hcold_leaf_SS	HSP60-03	27.32192	27.29828	27.36662	0.034706	27.32894	21.84457	5.484373	11.58692	-6.10255	68.71488
1hcold_stem_SS	HSP60-03	34.73101	33.23652	35.09171	0.983643	34.35308	24.30848	10.04461	9.741697	0.30291	0.810616
24hcold_leaf_SS	HSP60-03	33.0382	33.11002	33.50274	0.250058	33.21699	24.30848	8.90851	11.58692	-2.67841	6.401518
24hcold_stem_SS	HSP60-03	26.02009	26.87153	27.11047	0.573147	26.66737	22.90618	3.761185	9.741697	-5.98051	63.14128
6hcold_leaf_SS	HSP60-03	24.34508	24.47895	24.85045	0.261834	24.55816	23.2969	1.261255	5.869412	-4.60816	24.38898
6hcold_stem_SS	HSP60-03	22.83818	22.56533	22.80611	0.149136	22.73654	21.45946	1.277077	9.741697	-8.46462	353.2681
1hcold_leaf_SS	HSP60-05	25.35835	25.31386	25.22349	0.06872	25.29857	21.84457	3.453997	4.621545	-1.16755	2.246295
1hcold_stem_SS	HSP60-05	23.66118	23.89551	24.39874	0.376862	23.98514	24.30848	-0.32333	4.30362	-4.62695	24.70879
24hcold_leaf_SS	HSP60-05	29.1979	29.20135	29.45807	0.149228	29.28577	24.30848	4.977296	4.621545	0.355751	0.781463
24hcold_stem_SS	HSP60-05	24.58121	24.49818	24.88746	0.205027	24.65562	22.90618	1.749436	4.30362	-2.55418	5.873349
6hcold_leaf_SS	HSP60-05	23.85489	24.65246	24.65093	0.460037	24.38609	23.2969	1.089186	4.621545	-3.53236	11.57034
6hcold_stem_SS	HSP60-05	22.75415	22.54519	22.65485	0.104522	22.6514	21.45946	1.191936	4.30362	-3.11168	8.643908
1hcold_leaf_SS	HSP60-09	31.132	32.18185	31.05303	0.630166	31.45563	21.84457	9.611059	2.370853	7.240206	0.006614
1hcold_stem_SS	HSP60-09	24.14689	23.99715	24.06835	0.074898	24.0708	24.30848	-0.23768	4.484283	-4.72196	26.39079
24hcold_leaf_SS	HSP60-09	26.02708	26.22297	26.50936	0.242551	26.25313	24.30848	1.944657	2.370853	-0.4262	1.343686
24hcold_stem_SS	HSP60-09	29.85395	29.64786	29.92288	0.143101	29.80823	22.90618	6.90205	4.484283	2.417767	0.187146
6hcold_leaf_SS	HSP60-09	24.70468	25.5364	26.07283	0.689367	25.43797	23.2969	2.141069	2.370853	-0.22978	1.172659
6hcold_stem_SS	HSP60-09	25.24359	25.1771	25.47672	0.157348	25.29914	21.45946	3.839674	4.484283	-0.64461	1.563316
1hcold_leaf_SS	HSP60-13	25.94737	26.24834	26.26122	0.177599	26.15231	21.84457	4.307741	1.320383	2.987358	0.1261
1hcold_stem_SS	HSP60-13	27.6803	27.96013	28.18848	0.254524	27.94297	24.30848	3.63449	2.556249	1.078241	0.473606
24hcold_leaf_SS	HSP60-13	26.01395	26.58429	27.59543	0.800913	26.73122	24.30848	2.422747	1.320383	1.102364	0.465753
24hcold_stem_SS	HSP60-13	24.86399	25.53272	24.82635	0.397407	25.07435	22.90618	2.168173	2.556249	-0.38808	1.308648
6hcold_leaf_SS	HSP60-13	31.53689	30.9642	31.65337	0.368894	31.38482	23.2969	8.087917	1.320383	6.767534	0.009178
6hcold_stem_SS	HSP60-13	29.76419	29.80706	30.9417	0.667802	30.17098	21.45946	8.711522	2.556249	6.155273	0.014031
1hcold_leaf_SS	HSP60-14	28.67154	30.20755	30.52405	0.990904	29.80105	21.84457	7.956481	1.986789	5.969691	0.015957
1hcold_stem_SS	HSP60-14	29.46028	29.38857	28.9029	0.30323	29.25058	24.30848	4.942107	8.863524	-3.92142	15.15179
24hcold_leaf_SS	HSP60-14	29.58999	29.88498	30.17426	0.292143	29.88307	24.30848	5.574598	1.986789	3.587809	0.083169

24hcold_stem_SS	HSP60-14	28.51739	28.80398	28.8155	0.168884	28.71229	22.90618	5.806108	8.863524	-3.05742	8.324798
6hcold_leaf_SS	HSP60-14	26.93605	26.98261	26.79533	0.097502	26.90466	23.2969	3.607759	1.986789	1.62097	0.325117
6hcold_stem_SS	HSP60-14	27.15883	27.28043	27.75397	0.314436	27.39774	21.45946	5.938281	8.863524	-2.92524	7.596013
1hcold_leaf_SS	HSP60-16	28.56406	29.20096	29.11688	0.346008	28.96063	21.84457	7.116066	3.760658	3.355408	0.097706
1hcold_stem_SS	HSP60-16	26.50151	26.98842	27.14805	0.336792	26.87932	24.30848	2.570847	5.297616	-2.72677	6.619713
24hcold_leaf_SS	HSP60-16	27.28875	27.27073	27.18104	0.057689	27.24684	24.30848	2.938365	3.760658	-0.82229	1.768214
24hcold_stem_SS	HSP60-16	27.55153	27.49416	28.03207	0.295396	27.69258	22.90618	4.786404	5.297616	-0.51121	1.425247
6hcold_leaf_SS	HSP60-16	29.2446	29.23025	29.41623	0.103483	29.29702	23.2969	6.000119	3.760658	2.239461	0.211765
6hcold_stem_SS	HSP60-16	26.71159	26.95373	27.05001	0.174368	26.90511	21.45946	5.445648	5.297616	0.148032	0.902481
1hcold_leaf_SS	HSP60-20	24.50615	24.51768	24.93823	0.246204	24.65402	21.84457	2.809452	6.426404	-3.61695	12.26906
1hcold_stem_SS	HSP60-20	30.76523	29.85787	30.01624	0.484661	30.21311	24.30848	5.904636	5.109433	0.795203	0.576262
24hcold_leaf_SS	HSP60-20	30.29842	30.97813	31.20655	0.472383	30.8277	24.30848	6.519221	6.426404	0.092817	0.93769
24hcold_stem_SS	HSP60-20	30.94935	30.98024	30.80831	0.091655	30.91263	22.90618	8.006454	5.109433	2.89702	0.134249
6hcold_leaf_SS	HSP60-20	Undetermined	34.85381	35.31656	0.327216	35.08519	23.2969	11.78828	6.426404	5.361879	0.024317
6hcold_stem_SS	HSP60-20	32.09697	32.4728	32.70997	0.309102	32.42658	21.45946	10.96712	5.109433	5.857686	0.017245
1hcold_leaf_SS	HSP70-01	27.97661	29.85476	29.55774	1.009592	29.1297	21.84457	7.285137	5.444796	1.840341	0.279256
1hcold_stem_SS	HSP70-01	28.83676	29.08653	28.85088	0.140307	28.92472	24.30848	4.616247	4.265441	0.350806	0.784146
24hcold_leaf_SS	HSP70-01	31.6061	32.46096	33.31683	0.855365	32.4613	24.30848	8.15282	5.444796	2.708024	0.153039
24hcold_stem_SS	HSP70-01	30.02076	29.90109	29.98824	0.061879	29.97003	22.90618	7.063849	4.265441	2.798407	0.143746
6hcold_leaf_SS	HSP70-01	26.98425	26.94198	27.01202	0.035274	26.97942	23.2969	3.682514	5.444796	-1.76228	3.392344
6hcold_stem_SS	HSP70-01	22.37347	23.39253	23.53017	0.631847	23.09872	21.45946	1.639261	4.265441	-2.62618	6.173891
1hcold_leaf_SS	HSP70-04	25.33198	25.45327	25.95345	0.329425	25.57956	21.84457	3.734996	1.272139	2.462857	0.181387
1hcold_stem_SS	HSP70-04	25.51826	25.91625	24.61211	0.668368	25.34887	24.30848	1.040399	7.406306	-6.36591	82.47626
24hcold_leaf_SS	HSP70-04	30.49541	30.30186	30.51453	0.117654	30.43727	24.30848	6.128791	1.272139	4.856652	0.034515
24hcold_stem_SS	HSP70-04	28.14745	28.83333	29.19519	0.532155	28.72532	22.90618	5.819142	7.406306	-1.58716	3.004582
6hcold_leaf_SS	HSP70-04	27.48028	27.55498	28.3523	0.483341	27.79585	23.2969	4.498951	1.272139	3.226812	0.106815
6hcold_stem_SS	HSP70-04	26.78351	26.80038	26.89022	0.057364	26.82471	21.45946	5.365245	7.406306	-2.04106	4.115482
1hcold_leaf_SS	HSP70-06	32.63295	32.37235	32.31373	0.169923	32.43968	21.84457	10.59511	9.314555	1.280555	0.411637
1hcold_stem_SS	HSP70-06	30.22207	29.96559	30.86525	0.463475	30.35097	24.30848	6.042497	11.81958	-5.77708	54.83706
24hcold_leaf_SS	HSP70-06	30.74076	31.28638	30.67258	0.336427	30.89991	24.30848	6.591431	9.314555	-2.72312	6.603008
24hcold_stem_SS	HSP70-06	31.58211	31.9467	33.0372	0.757124	32.18867	22.90618	9.282489	11.81958	-2.53709	5.80416

6hcold_leaf_SS	HSP70-06	32.27287	32.95839	32.19918	0.418686	32.47681	23.2969	9.179908	9.314555	-0.13465	1.097824
6hcold_stem_SS	HSP70-06	27.94586	28.89501	29.43262	0.752812	28.75783	21.45946	7.298366	11.81958	-4.52121	22.96253
1hcold_leaf_SS	HSP70-15	27.10615	26.799	27.4347	0.317909	27.11328	21.84457	5.268714	2.556249	2.712465	0.152569
1hcold_stem_SS	HSP70-15	28.68798	28.66827	27.36599	0.757624	28.24075	24.30848	3.932271	2.968342	0.963929	0.512659
24hcold_leaf_SS	HSP70-15	29.89261	28.98958	29.09169	0.49453	29.32463	24.30848	5.01615	2.556249	2.4599	0.181759
24hcold_stem_SS	HSP70-15	27.57154	28.06842	28.89115	0.666477	28.17704	22.90618	5.270856	2.968342	2.302513	0.20271
6hcold_leaf_SS	HSP70-15	27.77003	28.85272	28.71049	0.588347	28.44441	23.2969	5.147507	2.556249	2.591258	0.165941
6hcold_stem_SS	HSP70-15	27.61066	27.83291	28.45764	0.439139	27.96707	21.45946	6.507611	2.968342	3.539269	0.086015
1hcold_leaf_SS	HSP70-16	23.8838	23.95994	23.90005	0.040102	23.91459	21.84457	2.070026	8.545858	-6.47583	89.00604
1hcold_stem_SS	HSP70-16	28.23629	27.68901	28.18109	0.301304	28.03546	24.30848	3.726984	11.52239	-7.79541	222.1527
24hcold_leaf_SS	HSP70-16	27.6488	27.56945	28.29785	0.399609	27.8387	24.30848	3.530222	8.545858	-5.01564	32.34869
24hcold_stem_SS	HSP70-16	33.64226	35.37273	35.82899	1.153579	34.94799	22.90618	12.04181	11.52239	0.519419	0.697653
6hcold_leaf_SS	HSP70-16	27.79851	28.95956	29.30918	0.790819	28.68908	23.2969	5.39218	8.545858	-3.15368	8.899214
6hcold_stem_SS	HSP70-16	26.21585	28.01458	27.90528	1.008427	27.37857	21.45946	5.919107	11.52239	-5.60329	48.6135
1hcold_leaf_SS	HSP70-19	30.14827	29.82715	29.40978	0.370291	29.79507	21.84457	7.950497	8.184944	-0.23445	1.176455
1hcold_stem_SS	HSP70-19	Undetermined	34.08273	32.88809	0.844738	33.48541	24.30848	9.176935	11.34792	-2.17098	4.503296
24hcold_leaf_SS	HSP70-19	24.92578	25.73759	25.19826	0.413149	25.28721	24.30848	0.978735	8.184944	-7.20621	147.6675
24hcold_stem_SS	HSP70-19	24.46081	24.95078	24.83125	0.25547	24.74761	22.90618	1.841432	11.34792	-9.50648	727.3389
6hcold_leaf_SS	HSP70-19	28.51739	28.80398	28.8155	0.168884	28.71229	23.2969	5.415385	8.184944	-2.76956	6.818994
6hcold_stem_SS	HSP70-19	26.50151	26.98842	27.14805	0.336792	26.87932	21.45946	5.419861	11.34792	-5.92806	60.8867
1hcold_leaf_SS	HSP70-21	30.46435	30.23656	Undetermined	0.161068	30.35046	21.84457	8.505888	4.361949	4.143939	0.056565
1hcold_stem_SS	HSP70-21	28.13186	28.39304	27.68072	0.360356	28.06854	24.30848	3.760063	7.347949	-3.58789	12.02434
24hcold_leaf_SS	HSP70-21	23.68166	22.90297	23.30608	0.389426	23.2969	24.30848	-1.01157	4.361949	-5.37352	41.45633
24hcold_stem_SS	HSP70-21	26.26523	27.07989	27.48732	0.622251	26.94415	22.90618	4.037966	7.347949	-3.30998	9.917545
6hcold_leaf_SS	HSP70-21	24.34508	24.47895	24.85045	0.261834	24.55816	23.2969	1.261255	4.361949	-3.10069	8.578315
6hcold_stem_SS	HSP70-21	23.85489	24.65246	24.65093	0.460037	24.38609	21.45946	2.926628	3.47949	-0.55286	1.466993
1hcold_leaf_SS	HSP70-24	24.70468	25.5364	26.07283	0.689367	25.43797	21.84457	3.593405	4.534038	-0.94063	1.91937
1hcold_stem_SS	HSP70-24	26.89708	26.73473	26.9209	0.101309	26.8509	24.30848	2.542429	7.550413	-5.00798	32.17758
24hcold_leaf_SS	HSP70-24	28.51739	28.80398	28.8155	0.168884	28.71229	24.30848	4.403813	4.534038	-0.13022	1.094464
24hcold_stem_SS	HSP70-24	26.50151	26.98842	27.14805	0.336792	26.87932	22.90618	3.973142	7.550413	-3.57727	11.93619
6hcold_leaf_SS	HSP70-24	26.71159	26.95373	27.05001	0.174368	26.90511	23.2969	3.608206	4.534038	-0.92583	1.89978

6hcold_stem_SS	HSP70-24	30.94935	30.98024	30.80831	0.091655	30.91263	21.45946	9.453172	7.550413	1.902759	0.267432
1hcold_leaf_SS	HSP90-02	28.83676	29.08653	28.85088	0.140307	28.92472	21.84457	7.080155	8.498686	-1.41853	2.673133
1hcold_stem_SS	HSP90-02	34.44129	32.11224	32.2856	1.297531	32.94638	24.30848	8.637901	3.73102	4.906881	0.033334
24hcold_leaf_SS	HSP90-02	31.15671	30.7942	29.16653	1.059994	30.37248	24.30848	6.064007	8.498686	-2.43468	5.406442
24hcold_stem_SS	HSP90-02	32.69386	35.34673	34.83886	1.40811	34.29315	22.90618	11.38697	5.10741	6.279561	0.012873
6hcold_leaf_SS	HSP90-02	36.22703	35.44515	34.5129	0.858164	35.39503	23.2969	12.09812	8.498686	3.599435	0.082502
6hcold_stem_SS	HSP90-02	32.47515	32.08386	32.57673	0.26024	32.37858	21.45946	10.91912	5.067315	5.851803	0.017315
1hcold_leaf_SS	HSP90-03	28.03961	27.79799	28.3514	0.277443	28.063	21.84457	6.218431	5.039092	1.179339	0.441554
1hcold_stem_SS	HSP90-03	33.37866	33.66043	34.20563	0.420419	33.74824	24.30848	9.439764	8.863524	0.576241	0.670709
24hcold_leaf_SS	HSP90-03	33.28052	32.77514	32.56901	0.366092	32.87489	24.30848	8.566414	5.039092	3.527322	0.08673
24hcold_stem_SS	HSP90-03	23.66118	23.89551	24.39874	0.376862	23.98514	22.90618	1.078962	3.52418	-2.44522	5.446078
6hcold_leaf_SS	HSP90-03	29.1979	29.20135	29.45807	0.149228	29.28577	23.2969	5.988867	5.039092	0.949775	0.517713
6hcold_stem_SS	HSP90-03	28.11772	27.98615	27.95056	0.088049	28.01814	21.45946	6.558682	8.863524	-2.30484	4.941132
1hcold_leaf_SS	HSP90-04	27.74746	27.84983	27.34196	0.268587	27.64641	21.84457	5.801846	3.029978	2.771867	0.146415
1hcold_stem_SS	HSP90-04	25.10076	26.49256	25.77788	0.695985	25.7904	24.30848	1.481923	7.234384	-5.75246	53.90926
24hcold_leaf_SS	HSP90-04	28.39124	28.82539	28.62257	0.217233	28.61307	24.30848	4.304592	3.029978	1.274613	0.413336
24hcold_stem_SS	HSP90-04	20.60145	21.39758	21.07269	0.400302	21.0239	22.90618	-1.88228	3.84522	-5.7275	52.98442
6hcold_leaf_SS	HSP90-04	22.62301	22.88767	22.73094	0.13308	22.74721	23.2969	-0.5497	3.029978	-3.57967	11.95609
6hcold_stem_SS	HSP90-04	20.69603	20.57241	20.70178	0.073089	20.65674	21.45946	-0.80272	2.341	-3.14372	8.838015
1hcold_leaf_SS	HSP90-05	22.01063	22.35659	22.21095	0.1737	22.19273	21.84457	0.348158	2.201825	-1.85367	3.614177
1hcold_stem_SS	HSP90-05	29.242	29.29919	29.63108	0.210082	29.39075	24.30848	5.082278	6.704037	-1.62176	3.077501
24hcold_leaf_SS	HSP90-05	28.26917	28.19065	28.32106	0.065657	28.26029	24.30848	3.951816	2.201825	1.749991	0.297304
24hcold_stem_SS	HSP90-05	22.74581	22.75136	22.82894	0.046474	22.77537	22.90618	-0.13081	4.1	-4.23081	18.77592
6hcold_leaf_SS	HSP90-05	36.22703	35.44515	34.5129	0.858164	35.39503	23.2969	12.09812	2.201825	9.896297	0.001049
6hcold_stem_SS	HSP90-05	32.47515	32.08386	32.57673	0.26024	32.37858	21.45946	10.91912	6.704037	4.215081	0.053844
1hcold_leaf_SS	HSP90-09	28.03961	27.79799	28.3514	0.277443	28.063	21.84457	6.218431	9.670187	-3.45176	10.94163
1hcold_stem_SS	HSP90-09	33.37866	33.66043	34.20563	0.420419	33.74824	24.30848	9.439764	11.38556	-1.94579	3.852499
24hcold_leaf_SS	HSP90-09	33.28052	32.77514	32.56901	0.366092	32.87489	24.30848	8.566414	9.670187	-1.10377	2.14916
24hcold_stem_SS	HSP90-09	34.27634	33.99267	32.41239	1.004328	33.56047	22.90618	10.65429	11.38556	-0.73127	1.660104
6hcold_leaf_SS	HSP90-09	26.54079	26.66036	27.2404	0.374211	26.81385	23.2969	3.516946	9.670187	-6.15324	71.17215
6hcold_stem_SS	HSP90-09	29.29169	28.94754	28.74875	0.274693	28.99599	21.45946	7.536531	11.38556	-3.84903	14.4103

1hcold_leaf_SS	sHSP-02	32.12342	32.54173	32.93776	0.407219	32.5343	21.84457	10.68973	7.727236	2.962497	0.128292
1hcold_stem_SS	sHSP-02	34.23447	34.05072	33.9184	0.158731	34.06787	24.30848	9.759392	10.22697	-0.46757	1.382783
24hcold_leaf_SS	sHSP-02	34.06779	33.98118	33.58911	0.255068	33.87936	24.30848	9.570884	7.727236	1.843648	0.278616
24hcold_stem_SS	sHSP-02	20.60145	21.39758	21.07269	0.400302	21.0239	22.90618	-1.88228	4.18932	-6.0716	67.25624
6hcold_leaf_SS	sHSP-02	22.62301	22.88767	22.73094	0.13308	22.74721	23.2969	-0.5497	1.087963	-1.63766	3.111603
6hcold_stem_SS	sHSP-02	20.69603	20.57241	20.70178	0.073089	20.65674	21.45946	-0.80272	2.20101	-3.00373	8.020723
1hcold_leaf_SS	sHSP-07	22.01063	22.35659	22.21095	0.1737	22.19273	21.84457	0.348158	10.98343	-10.6353	1590.512
1hcold_stem_SS	sHSP-07	29.242	29.29919	29.63108	0.210082	29.39075	24.30848	5.082278	14.19922	-9.11694	555.229
24hcold_leaf_SS	sHSP-07	28.26917	28.19065	28.32106	0.065657	28.26029	24.30848	3.951816	10.98343	-7.03162	130.8361
24hcold_stem_SS	sHSP-07	22.74581	22.75136	22.82894	0.046474	22.77537	22.90618	-0.13081	7.108542	-7.23935	151.0993
6hcold_leaf_SS	sHSP-07	31.50501	31.05158	31.0986	0.249324	31.21839	23.2969	7.921491	10.98343	-3.06194	8.35096
6hcold_stem_SS	sHSP-07	25.70171	25.96931	25.635	0.176929	25.76867	21.45946	4.309211	1.998546	2.310665	0.201568
1hcold_leaf_SS	sHSP-08	23.66118	23.89551	24.39874	0.376862	23.98514	21.84457	2.140575	6.28698	-4.14641	17.70894
1hcold_stem_SS	sHSP-08	29.1979	29.20135	29.45807	0.149228	29.28577	24.30848	4.977296	5.813431	-0.83614	1.785262
24hcold_leaf_SS	sHSP-08	27.8929	28.15237	28.13705	0.145585	28.06077	24.30848	3.752296	6.28698	-2.53468	5.794501
24hcold_stem_SS	sHSP-08	27.66347	27.67238	27.90043	0.134309	27.74543	22.90618	4.839246	5.813431	-0.97419	1.964532
6hcold_leaf_SS	sHSP-08	31.97957	32.11514	32.6424	0.350172	32.2457	23.2969	8.948795	6.28698	2.661815	0.158021
6hcold_stem_SS	sHSP-08	34.18128	34.34284	34.57661	0.198761	34.36691	21.45946	12.90745	5.813431	7.094017	0.00732
1hcold_leaf_SS	sHSP-11	31.63097	32.11416	31.79771	0.24543	31.84761	21.84457	10.00305	4.291369	5.711677	0.019082
1hcold_stem_SS	sHSP-11	32.67087	33.16291	32.64088	0.293122	32.82489	24.30848	8.51641	4.853923	3.662487	0.078974
24hcold_leaf_SS	sHSP-11	27.74905	28.16279	28.43245	0.344221	28.11476	24.30848	3.806285	4.291369	-0.48508	1.399667
24hcold_stem_SS	sHSP-11	21.8062	21.73986	22.06723	0.173063	21.8711	22.90618	-1.03508	4.853923	-5.88901	59.26082
6hcold_leaf_SS	sHSP-11	28.70565	28.34646	28.73429	0.216119	28.59547	23.2969	5.298565	4.291369	1.007196	0.497512
6hcold_stem_SS	sHSP-11	32.62406	33.91491	31.38751	1.263797	32.64216	21.45946	11.1827	4.853923	6.328776	0.012441
1hcold_leaf_SS	sHSP-15	33.66085	33.94127	34.02463	0.190578	33.87558	21.84457	12.03101	3.973532	8.057481	0.003754
1hcold_stem_SS	sHSP-15	34.52968	31.08104	32.40417	1.739808	32.67163	24.30848	8.363156	7.387156	0.975999	0.508388
24hcold_leaf_SS	sHSP-15	33.17757	Undetermined	Undetermined	#DIV/0!	33.17757	24.30848	8.869092	3.973532	4.89556	0.033596
24hcold_stem_SS	sHSP-15	25.87383	25.72547	26.19626	0.2407	25.93186	22.90618	3.025675	7.387156	-4.36148	20.55591
6hcold_leaf_SS	sHSP-15	34.95469	34.9197	35.90403	0.558476	35.25947	23.2969	11.96257	3.973532	7.989035	0.003936
6hcold_stem_SS	sHSP-15	27.7945	27.72374	28.42882	0.388266	27.98235	21.45946	6.52289	7.387156	-0.86427	1.820413
1hcold_leaf_SS	sHSP-25	32.43135	32.31139	33.42099	0.608958	32.72124	21.84457	10.87668	7.119162	3.757515	0.073939

1hcold_stem_SS	sHSP-25	31.6191	32.21506	33.17901	0.787158	32.33772	24.30848	8.029245	5.10987	2.919375	0.132184
24hcold_leaf_SS	sHSP-25	29.35702	30.4772	30.10041	0.569999	29.97821	24.30848	5.669732	7.119162	-1.44943	2.731002
24hcold_stem_SS	sHSP-25	29.25862	28.50294	28.43503	0.457158	28.7322	22.90618	5.826017	11.28181	-5.45579	43.88903
6hcold_leaf_SS	sHSP-25	35.27097	34.9223	32.72495	1.380346	34.30608	23.2969	11.00917	-2.4587	13.46787	8.83E-05
6hcold_stem_SS	sHSP-25	34.73101	33.23652	35.09171	0.983643	34.35308	21.45946	12.89362	2.00793	10.88569	0.000529
1hcold_leaf_SS	sHSP-26	33.0382	33.11002	33.50274	0.250058	33.21699	21.84457	11.37242	4.7897	6.582717	0.010433
1hcold_stem_SS	sHSP-26	35.60633	33.48606	32.51612	1.580386	33.8695	24.30848	9.561025	7.8581	1.702925	0.307163
24hcold_leaf_SS	sHSP-26	29.70767	27.82071	27.70324	1.124885	28.41054	24.30848	4.102062	4.7897	-0.68764	1.610645
24hcold_stem_SS	sHSP-26	33.52704	33.03802	32.9831	0.299454	33.18272	22.90618	10.27654	4.1	6.17654	0.013825
6hcold_leaf_SS	sHSP-26	29.5592	29.82302	29.5971	0.142636	29.65977	23.2969	6.362869	4.7897	1.573169	0.336069
6hcold_stem_SS	sHSP-26	32.15236	32.54024	31.91099	0.317454	32.2012	21.45946	10.74173	10.522	0.219733	0.858724
1hcold_leaf_SS	sHSP-27	31.02851	30.57032	26.66107	2.400231	29.41997	21.84457	7.575399	5.15657	2.418829	0.187008
1hcold_stem_SS	sHSP-27	30.92117	30.35887	32.60022	1.166123	31.29342	24.30848	6.984943	7.780998	-0.79605	1.736346
24hcold_leaf_SS	sHSP-27	25.86894	26.56777	26.7788	0.476224	26.40517	24.30848	2.096696	5.15657	-3.05987	8.339
24hcold_stem_SS	sHSP-27	30.23967	31.14198	30.95139	0.475574	30.77768	22.90618	7.871497	7.780998	0.0905	0.939197
6hcold_leaf_SS	sHSP-27	31.50501	31.05158	31.0986	0.249324	31.21839	23.2969	7.921491	2.8156	5.105891	0.029038
6hcold_stem_SS	sHSP-27	25.70171	25.96931	25.635	0.176929	25.76867	21.45946	4.309211	7.780998	-3.47179	11.09461
1hcold_leaf_SS	sHSP-30	26.82335	27.33385	28.4141	0.812205	27.52377	21.84457	5.679199	4.7897	0.889499	0.539802
1hcold_stem_SS	sHSP-30	32.87455	33.51234	32.39898	0.558646	32.92862	24.30848	8.620149	2.524207	6.095942	0.01462
24hcold_leaf_SS	sHSP-30	30.92519	32.27888	31.34027	0.69351	31.51478	24.30848	7.206304	6.190665	1.015639	0.494609
24hcold_stem_SS	sHSP-30	30.06373	30.92839	31.31714	0.641587	30.76975	22.90618	7.863571	2.524207	5.339364	0.0247
6hcold_leaf_SS	sHSP-30	27.8929	28.15237	28.13705	0.145585	28.06077	23.2969	4.763868	6.190665	-1.4268	2.688493
6hcold_stem_SS	sHSP-30	27.66347	27.67238	27.90043	0.134309	27.74543	21.45946	6.285964	6.190665	0.095299	0.936078
**Tolerant cultivar in response to dehydration stress**											
**SAMPLE**	**DETECTOR**	**Ct1**	**Ct2**	**Ct3**	**sd**	**Avg Ct**	**End CTl**	**dCt**	**CTL0h**	**ddCt**	**Log Ct**
1hdehyd_leaf_ST	ACT	25.18256	25.45622	25.64271	0.231448	25.42716	25.42716	0		0	1
1hdehyd_stem_ST	ACT	22.06608	22.41697	22.47828	0.222406	22.32045	22.32045	0		0	1
24hdehyd_stem_ST	ACT	22.12395	22.98197	22.26468	0.460164	22.45687	22.45687	0		0	1
6hdehyd_leaf_ST	ACT	22.41859	21.97412	22.85413	0.440013	22.41561	22.41561	0		0	1
6hdehyd_stem_ST	ACT	25.09282	26.04938	25.03711	0.569036	25.3931	25.3931	0		0	1

6hheat_leaf_ST	ACT	25.82601	25.74098	25.85561	0.059507	25.80753	25.3931	0.414431		0.414431	0.750316
1hdehyd_leaf_ST	HSP100-07	27.48628	27.58889	27.98004	0.260555	27.68507	25.42716	2.25791	-3.76111	6.019022	0.01542
1hdehyd_stem_ST	HSP100-07	25.63411	25.94226	26.29443	0.330403	25.95693	22.32045	3.636485	-8.93484	12.57133	0.000164
24hdehyd_leaf_ST	HSP100-07	26.94784	26.00558	27.67332	0.836216	26.87558	22.32045	4.555132	-3.76111	8.316245	0.003137
24hdehyd_stem_ST	HSP100-07	27.14226	27.35905	27.11025	0.135353	27.20385	22.45687	4.746986	8.934842	-4.18786	18.22512
6hdehyd_leaf_ST	HSP100-07	26.67082	26.71103	26.91674	0.131917	26.7662	22.41561	4.350582	-3.76111	8.111694	0.003615
6hdehyd_stem_ST	HSP100-07	27.65285	27.64542	Undetermined	0.005251	27.64914	25.3931	2.256036	8.934842	-6.67881	102.4522
1hdehyd_leaf_ST	HSP100-08	26.29036	24.9731	25.3395	0.679898	25.53432	25.42716	0.107158	-6.41278	6.519936	0.010897
1hdehyd_stem_ST	HSP100-08	26.36649	26.2802	26.27881	0.050227	26.3085	22.32045	3.988052	5.156522	-1.16847	2.247733
24hdehyd_leaf_ST	HSP100-08	26.30015	26.07559	25.90697	0.197251	26.09424	22.32045	3.773793	-6.41278	10.18657	0.000858
24hdehyd_stem_ST	HSP100-08	26.18481	26.08663	25.96276	0.111274	26.07807	22.45687	3.621203	5.156522	-1.53532	2.898525
6hdehyd_leaf_ST	HSP100-08	25.74733	25.97733	25.93443	0.1223	25.88636	22.41561	3.470751	-6.41278	9.883529	0.001059
6hdehyd_stem_ST	HSP100-08	26.31973	Undetermined	26.25373	0.04667	26.28673	25.3931	0.893628	5.156522	-4.26289	19.19813
1hdehyd_leaf_ST	HSP100-11	26.49066	26.90824	27.31562	0.41249	26.90484	25.42716	1.477676	0.939984	0.537692	0.688872
1hdehyd_stem_ST	HSP100-11	26.71658	27.0231	26.92653	0.156715	26.88874	22.32045	4.568293	12.08982	-7.52153	183.741
24hdehyd_leaf_ST	HSP100-11	26.20616	26.34653	26.12397	0.112543	26.22555	22.32045	3.905109	0.939984	2.965125	0.128059
24hdehyd_stem_ST	HSP100-11	26.95246	26.28134	26.45982	0.347599	26.56454	22.45687	4.107675	12.08982	-7.98215	252.8517
6hdehyd_leaf_ST	HSP100-11	26.09673	26.03325	26.59786	0.309288	26.24261	22.41561	3.826999	0.939984	2.887015	0.135183
6hdehyd_stem_ST	HSP100-11	26.51499	26.83705	26.71482	0.162582	26.68895	25.3931	1.295853	12.08982	-10.794	1775.451
1hdehyd_leaf_ST	HSP100-12	26.89708	26.73473	26.9209	0.101309	26.8509	25.42716	1.423744	0.37819	1.045554	0.484459
1hdehyd_stem_ST	HSP100-12	27.50985	27.62151	27.58974	0.057531	27.5737	22.32045	5.253256	11.52094	-6.26768	77.04786
24hdehyd_leaf_ST	HSP100-12	26.00511	26.47907	26.59614	0.31296	26.36011	22.32045	4.039665	0.37819	3.661475	0.079029
24hdehyd_stem_ST	HSP100-12	26.92863	26.92576	26.80374	0.071293	26.88605	22.45687	4.429181	5.209399	-0.78022	1.71739
6hdehyd_leaf_ST	HSP100-12	26.86088	26.89069	26.0047	0.503144	26.58543	22.41561	4.169812	0.37819	3.791622	0.072212
6hdehyd_stem_ST	HSP100-12	27.21439	27.29408	27.51397	0.155163	27.34081	25.3931	1.947712	11.52094	-9.57323	761.7783
1hdehyd_leaf_ST	HSP100-16	26.06545	25.57944	25.59727	0.2756	25.74739	25.42716	0.320225	-3.05802	3.378241	0.096172
1hdehyd_stem_ST	HSP100-16	26.91216	26.84743	26.64076	0.141752	26.80012	22.32045	4.47967	8.364046	-3.88438	14.76772
24hdehyd_leaf_ST	HSP100-16	25.99571	26.39964	25.87569	0.274493	26.09035	22.32045	3.7699	-3.05802	6.827916	0.008802
24hdehyd_stem_ST	HSP100-16	26.97777	27.02291	26.87775	0.074287	26.95948	22.45687	4.502613	8.364046	-3.86143	14.53473
6hdehyd_leaf_ST	HSP100-16	25.19537	25.44823	25.97802	0.39941	25.54054	22.41561	3.124927	-3.05802	6.182943	0.013764
6hdehyd_stem_ST	HSP100-16	25.96137	27.51397	27.48997	0.889549	26.98844	25.3931	1.595334	8.364046	-6.76871	109.0398

1hdehyd_leaf_ST	HSP100-18	25.84046	26.03082	25.97714	0.098147	25.94948	25.42716	0.522315	-0.04711	0.569422	0.673887
1hdehyd_stem_ST	HSP100-18	27.1786	26.86223	26.08436	0.563107	26.7084	22.32045	4.387951	8.121113	-3.73316	13.29822
24hdehyd_leaf_ST	HSP100-18	26.02379	26.00824	26.12372	0.062667	26.05192	22.32045	3.731472	-0.04711	3.778579	0.072868
24hdehyd_stem_ST	HSP100-18	26.73391	26.67071	25.63791	0.615347	26.34751	22.45687	3.890645	8.121113	-4.23047	18.77144
6hdehyd_leaf_ST	HSP100-18	22.46122	22.25774	22.48682	0.125521	22.40192	22.41561	-0.01369	-0.04711	0.033418	0.977103
6hdehyd_stem_ST	HSP100-18	26.74545	26.51623	26.85495	0.172852	26.70554	25.3931	1.312442	8.121113	-6.80867	112.1022
1hdehyd_leaf_ST	HSP100-20	26.34935	26.00218	28.30709	1.242704	26.88621	25.42716	1.459047	-3.50825	4.967298	0.031966
1hdehyd_stem_ST	HSP100-20	26.20616	26.34653	26.12397	0.112543	26.22555	22.32045	3.905109	2.393056	1.512053	0.350612
24hdehyd_leaf_ST	HSP100-20	26.09673	26.03325	26.59786	0.309288	26.24261	22.32045	3.922167	-3.50825	7.430418	0.005797
24hdehyd_stem_ST	HSP100-20	26.89708	26.73473	26.9209	0.101309	26.8509	22.45687	4.394039	2.393056	2.000984	0.24983
6hdehyd_leaf_ST	HSP100-20	26.00511	26.47907	26.59614	0.31296	26.36011	22.41561	3.944496	-3.50825	7.452747	0.005708
6hdehyd_stem_ST	HSP100-20	26.99183	26.4292	26.71834	0.28135	26.71312	25.3931	1.32002	2.393056	-1.07304	2.103856
1hdehyd_leaf_ST	HSP60-02	26.55728	26.98822	27.18542	0.321237	26.91031	25.42716	1.483147	-4.85859	6.341742	0.01233
1hdehyd_stem_ST	HSP60-02	34.56396	34.55016	36.68255	1.22717	35.26555	22.32045	12.94511	3.028418	9.91669	0.001035
24hdehyd_leaf_ST	HSP60-02	30.46435	30.23656	Undetermined	0.161068	30.35046	22.32045	8.030011	-4.85859	12.8886	0.000132
24hdehyd_stem_ST	HSP60-02	29.66451	29.93796	30.52295	0.438537	30.04181	22.45687	7.584942	3.028418	4.556524	0.042496
6hdehyd_leaf_ST	HSP60-02	28.79851	28.95956	29.30918	0.261069	29.02242	22.41561	6.606803	-4.85859	11.4654	0.000354
6hdehyd_stem_ST	HSP60-02	28.32365	28.96292	28.90736	0.354132	28.73131	25.3931	3.338209	3.028418	0.309791	0.806759
1hdehyd_leaf_ST	HSP60-03	29.7912	29.51664	29.22453	0.283383	29.51079	25.42716	4.083629	4.814743	-0.73111	1.65992
1hdehyd_stem_ST	HSP60-03	30.31169	29.18178	28.96123	0.724462	29.4849	22.32045	7.164454	13.10722	-5.94276	61.51059
24hdehyd_leaf_ST	HSP60-03	30.17745	30.55206	30.54793	0.215099	30.42582	22.32045	8.105371	4.814743	3.290628	0.102193
24hdehyd_stem_ST	HSP60-03	33.93066	Undetermined	33.08021	0.601363	33.50544	22.45687	11.04857	13.10722	-2.05865	4.165953
6hdehyd_leaf_ST	HSP60-03	34.80298	35.17245	35.83423	0.522484	35.26989	22.41561	12.85427	4.814743	8.039529	0.003801
6hdehyd_stem_ST	HSP60-03	Undetermined	25.99571	26.39964	0.285617	26.19767	25.3931	0.804572	7.217496	-6.41292	85.2084
1hdehyd_leaf_ST	HSP60-05	25.86894	26.56777	26.7788	0.476224	26.40517	25.42716	0.978011	2.073274	-1.09526	2.13652
1hdehyd_stem_ST	HSP60-05	30.23967	31.14198	30.95139	0.475574	30.77768	22.32045	8.457232	-11.46	19.9172	1.01E-06
24hdehyd_leaf_ST	HSP60-05	31.50501	31.05158	31.0986	0.249324	31.21839	22.32045	8.89795	2.073274	6.824676	0.008822
24hdehyd_stem_ST	HSP60-05	25.70171	25.96931	25.635	0.176929	25.76867	22.45687	3.311807	7.521639	-4.20983	18.50485
6hdehyd_leaf_ST	HSP60-05	26.82335	27.33385	28.4141	0.812205	27.52377	22.41561	5.108153	2.073274	3.034879	0.122014
6hdehyd_stem_ST	HSP60-05	32.87455	33.51234	32.39898	0.558646	32.92862	25.3931	7.535523	11.45997	-3.92444	15.18361
1hdehyd_leaf_ST	HSP60-09	30.92519	32.27888	31.34027	0.69351	31.51478	25.42716	6.087619	-0.14832	6.23594	0.013268

1hdehyd_stem_ST	HSP60-09	30.06373	30.92839	31.31714	0.641587	30.76975	22.32045	8.449306	8.451698	-0.00239	1.001659
24hdehyd_leaf_ST	HSP60-09	27.8929	28.15237	28.13705	0.145585	28.06077	22.32045	5.740326	-0.14832	5.888647	0.016879
24hdehyd_stem_ST	HSP60-09	27.66347	27.67238	27.90043	0.134309	27.74543	22.45687	5.288561	8.451698	-3.16314	8.957756
6hdehyd_leaf_ST	HSP60-09	28.95706	30.60958	29.68297	0.828287	29.74987	22.41561	7.334259	-0.14832	7.48258	0.005591
6hdehyd_stem_ST	HSP60-09	26.5793	26.78285	26.92069	0.171749	26.76095	25.3931	1.367847	8.451698	-7.08385	135.66
1hdehyd_leaf_ST	HSP60-13	29.25862	28.50294	28.43503	0.457158	28.7322	25.42716	3.305037	-1.11553	4.420571	0.046696
1hdehyd_stem_ST	HSP60-13	30.5948	30.09433	31.12007	0.512923	30.60307	22.32045	8.28262	6.638692	1.643928	0.319984
24hdehyd_leaf_ST	HSP60-13	30.46845	30.55067	30.77137	0.156645	30.59683	22.32045	8.276383	-1.11553	9.391918	0.001489
24hdehyd_stem_ST	HSP60-13	31.97957	32.11514	32.6424	0.350172	32.2457	22.45687	9.788834	6.638692	3.150142	0.112645
6hdehyd_leaf_ST	HSP60-13	34.18128	34.34284	34.57661	0.198761	34.36691	22.41561	11.9513	-1.11553	13.06683	0.000117
6hdehyd_stem_ST	HSP60-13	31.63097	32.11416	31.79771	0.24543	31.84761	25.3931	6.454512	6.638692	-0.18418	1.136171
1hdehyd_leaf_ST	HSP60-14	32.67087	33.16291	32.64088	0.293122	32.82489	25.42716	7.397725	1.098698	6.299027	0.0127
1hdehyd_stem_ST	HSP60-14	32.64065	33.83352	33.54919	0.623062	33.34112	22.32045	11.02068	12.2208	-1.20013	2.297599
24hdehyd_leaf_ST	HSP60-14	33.07114	35.19228	33.13796	1.205816	33.80046	22.32045	11.48001	1.098698	10.38132	0.00075
24hdehyd_stem_ST	HSP60-14	27.11608	27.42363	27.76134	0.322747	27.43368	22.45687	4.976819	6.220803	-1.24398	2.368517
6hdehyd_leaf_ST	HSP60-14	25.66739	25.617	25.69202	0.038241	25.6588	22.41561	3.243191	1.098698	2.144493	0.226174
6hdehyd_stem_ST	HSP60-14	27.87881	27.63464	27.93376	0.159224	27.81573	25.3931	2.422632	1.220803	1.20183	0.434724
1hdehyd_leaf_ST	HSP60-16	28.80105	25.64463	25.69985	1.806632	26.71518	25.42716	1.288015	-2.73807	4.02608	0.06138
1hdehyd_stem_ST	HSP60-16	26.81319	24.61252	24.93532	1.188385	25.45368	22.32045	3.133235	6.27347	-3.14024	8.816677
24hdehyd_leaf_ST	HSP60-16	30.70225	30.44643	31.16147	0.362309	30.77005	22.32045	8.449607	-2.73807	11.18767	0.000429
24hdehyd_stem_ST	HSP60-16	31.90872	31.94539	34.23858	1.334682	32.69756	22.45687	10.2407	6.27347	3.967227	0.063936
6hdehyd_leaf_ST	HSP60-16	26.10387	25.97194	25.37901	0.386087	25.81827	22.41561	3.402658	-2.73807	6.140723	0.014173
6hdehyd_stem_ST	HSP60-16	25.90794	25.89382	25.93467	0.020747	25.91214	25.3931	0.519039	6.27347	-5.75443	53.9829
1hdehyd_leaf_ST	HSP60-20	24.59753	24.03336	24.76292	0.382512	24.4646	25.42716	-0.96256	-4.0606	3.098041	0.116788
1hdehyd_stem_ST	HSP60-20	23.89199	24.13788	24.24441	0.180743	24.09143	22.32045	1.77098	5.911655	-4.14067	17.63873
24hdehyd_leaf_ST	HSP60-20	34.04357	32.84406	33.85418	0.644859	33.5806	22.32045	11.26016	-4.0606	15.32076	2.44E-05
24hdehyd_stem_ST	HSP60-20	27.7644	27.91184	27.82387	0.074179	27.83337	22.45687	5.376503	5.911655	-0.53515	1.449094
6hdehyd_leaf_ST	HSP60-20	25.66633	26.03157	26.28833	0.312576	25.99541	22.41561	3.579795	-4.0606	7.640394	0.005012
6hdehyd_stem_ST	HSP60-20	26.60755	26.34211	26.3114	0.162844	26.42036	25.3931	1.027254	5.911655	-4.8844	29.53597
1hdehyd_leaf_ST	HSP70-01	27.02491	26.50502	26.60494	0.275874	26.71162	25.42716	1.284463	-2.88662	4.171079	0.055511
1hdehyd_stem_ST	HSP70-01	31.78365	30.67354	29.88179	0.955364	30.77966	22.32045	8.459214	7.069286	1.389928	0.381584

24hdehyd_leaf_ST	HSP70-01	29.77603	29.42248	29.96909	0.277204	29.72253	22.32045	7.402087	-2.88662	10.2887	0.000799
24hdehyd_stem_ST	HSP70-01	30.6542	Undetermined	30.41925	0.166133	30.53673	22.45687	8.079861	7.069286	1.010575	0.496348
6hdehyd_leaf_ST	HSP70-01	26.79255	25.9038	26.08782	0.469112	26.26139	22.41561	3.845775	-2.88662	6.732391	0.009405
6hdehyd_stem_ST	HSP70-01	25.45696	24.80629	24.16276	0.647102	24.80867	25.3931	-0.58443	7.069286	-7.65372	201.3719
1hdehyd_leaf_ST	HSP70-04	24.69885	24.69057	25.34372	0.374731	24.91105	25.42716	-0.51612	-2.98657	2.47045	0.180435
1hdehyd_stem_ST	HSP70-04	25.55366	25.44615	25.33229	0.110699	25.44403	22.32045	3.123587	5.364267	-2.24068	4.7262
24hdehyd_leaf_ST	HSP70-04	36.14188	35.47031	34.95631	0.59453	35.52283	22.32045	13.20239	-2.98657	16.18895	1.34E-05
24hdehyd_stem_ST	HSP70-04	22.62301	22.88767	22.73094	0.13308	22.74721	22.45687	0.290343	5.364267	-5.07392	33.68243
6hdehyd_leaf_ST	HSP70-04	20.69603	20.57241	20.70178	0.073089	20.65674	22.41561	-1.75887	-2.98657	1.227691	0.427
6hdehyd_stem_ST	HSP70-04	22.01063	22.35659	22.21095	0.1737	22.19273	25.3931	-3.20038	5.364267	-8.56464	378.6296
1hdehyd_leaf_ST	HSP70-06	29.242	29.29919	29.63108	0.210082	29.39075	25.42716	3.963593	-4.40525	8.368842	0.003025
1hdehyd_stem_ST	HSP70-06	28.26917	28.19065	28.32106	0.065657	28.26029	22.32045	5.939846	7.291443	-1.3516	2.551944
24hdehyd_leaf_ST	HSP70-06	22.74581	22.75136	22.82894	0.046474	22.77537	22.32045	0.454924	-4.40525	4.860173	0.03443
24hdehyd_stem_ST	HSP70-06	24.63038	25.27679	24.45398	0.433202	24.78705	22.45687	2.330184	7.291443	-4.96126	31.15214
6hdehyd_leaf_ST	HSP70-06	29.10174	27.67875	27.3989	0.913137	28.0598	22.41561	5.644185	-4.40525	10.04943	0.000944
6hdehyd_stem_ST	HSP70-06	23.99408	25.11066	25.48217	0.774508	24.8623	25.3931	-0.5308	7.291443	-7.82224	226.323
1hdehyd_leaf_ST	HSP70-15	23.01842	23.51464	24.01229	0.496934	23.51511	25.42716	-1.91205	-3.82942	1.917374	0.264736
1hdehyd_stem_ST	HSP70-15	30.74289	31.1356	30.6959	0.241444	30.85813	22.32045	8.537685	5.167391	3.370294	0.096703
24hdehyd_leaf_ST	HSP70-15	35.93066	Undetermined	33.08021	2.015577	34.50544	22.32045	12.18499	-3.82942	16.01441	1.51E-05
24hdehyd_stem_ST	HSP70-15	30.42485	32.88528	30.81874	1.321578	31.37629	22.45687	8.919424	5.167391	3.752033	0.074221
6hdehyd_leaf_ST	HSP70-15	32.82025	33.36525	33.35513	0.311775	33.18021	22.41561	10.7646	-3.82942	14.59402	4.04E-05
6hdehyd_stem_ST	HSP70-15	36.75892	36.82159	Undetermined	0.044314	36.79026	25.3931	11.39715	5.167391	6.229763	0.013325
1hdehyd_leaf_ST	HSP70-16	36.60319	34.74488	34.25642	1.238225	35.2015	25.42716	9.774336	-1.78163	11.55597	0.000332
1hdehyd_stem_ST	HSP70-16	36.5269	Undetermined	36.87495	0.246109	36.70093	22.32045	14.38048	7.564647	6.815833	0.008876
24hdehyd_leaf_ST	HSP70-16	36.47678	34.95816	36.45321	0.87005	35.96272	22.32045	13.64227	-1.78163	15.4239	2.27E-05
24hdehyd_stem_ST	HSP70-16	34.80298	30.17245	36.83423	3.414362	33.93655	22.45687	11.47969	13.56465	-2.08496	4.242632
6hdehyd_leaf_ST	HSP70-16	29.66788	29.91172	29.78764	0.121929	29.78908	22.41561	7.373467	-1.78163	9.155097	0.001754
6hdehyd_stem_ST	HSP70-16	25.93585	25.68763	26.07311	0.195385	25.89886	25.3931	0.50576	1.646467	-1.14071	2.20489
1hdehyd_leaf_ST	HSP70-19	27.92614	28.25298	27.50412	0.375435	27.89441	25.42716	2.467252	-4.92564	7.392896	0.00595
1hdehyd_stem_ST	HSP70-19	26.68342	26.59494	26.92233	0.169354	26.73356	22.32045	4.413119	8.619916	-4.2068	18.46597
24hdehyd_leaf_ST	HSP70-19	27.34448	27.4033	27.28838	0.057464	27.34539	22.32045	5.024941	-4.92564	9.950584	0.001011

24hdehyd_stem_ST	HSP70-19	27.24379	27.22682	26.99248	0.140454	27.15436	22.45687	4.697498	8.619916	-3.92242	15.16231
6hdehyd_leaf_ST	HSP70-19	28.9174	30.99837	30.52771	1.091256	30.14783	22.41561	7.732216	-4.92564	12.65786	0.000155
6hdehyd_stem_ST	HSP70-19	34.80298	30.17245	36.83423	3.414362	33.93655	25.3931	8.543451	8.619916	-0.07646	1.054431
1hdehyd_leaf_ST	HSP70-21	21.35093	21.44282	21.58208	0.116382	21.45861	25.42716	-3.96855	3.379002	-7.34755	162.8674
1hdehyd_stem_ST	HSP70-21	23.66929	23.58276	23.62448	0.043275	23.62551	22.32045	1.305065	8.746584	-7.44152	173.8282
24hdehyd_leaf_ST	HSP70-21	30.21972	30.23488	30.49759	0.156235	30.3174	22.32045	7.996953	3.379002	4.617952	0.040725
24hdehyd_stem_ST	HSP70-21	31.53533	31.32369	31.26651	0.141609	31.37518	22.45687	8.918312	8.746584	0.171728	0.887779
6hdehyd_leaf_ST	HSP70-21	26.43199	26.50776	26.68833	0.131689	26.54269	22.41561	4.12708	3.379002	0.748078	0.595396
6hdehyd_stem_ST	HSP70-21	34.74134	34.5789	34.4329	0.154295	34.58438	25.3931	9.191281	8.746584	0.444697	0.734739
1hdehyd_leaf_ST	HSP70-24	28.13186	28.39304	27.68072	0.360356	28.06854	25.42716	2.641378	0.777854	1.863524	0.274804
1hdehyd_stem_ST	HSP70-24	23.68166	22.90297	23.30608	0.389426	23.2969	22.32045	0.976459	2.017716	-1.04126	2.05802
24hdehyd_leaf_ST	HSP70-24	26.26523	27.07989	27.48732	0.622251	26.94415	22.32045	4.623701	0.777854	3.845847	0.069548
24hdehyd_stem_ST	HSP70-24	24.34508	24.47895	24.85045	0.261834	24.55816	22.45687	2.101293	2.017716	0.083577	0.943715
6hdehyd_leaf_ST	HSP70-24	23.85489	24.65246	24.65093	0.460037	24.38609	22.41561	1.970476	0.777854	1.192622	0.437507
6hdehyd_stem_ST	HSP70-24	24.70468	25.5364	26.07283	0.689367	25.43797	25.3931	0.044872	2.017716	-1.97284	3.925412
1hdehyd_leaf_ST	HSP90-02	23.85489	24.65246	24.65093	0.460037	24.38609	25.42716	-1.04107	0.844496	-1.88557	3.69498
1hdehyd_stem_ST	HSP90-02	24.70468	25.5364	26.07283	0.689367	25.43797	22.32045	3.117528	1.036997	2.080531	0.236427
24hdehyd_leaf_ST	HSP90-02	26.89708	26.73473	26.9209	0.101309	26.8509	22.32045	4.53046	0.844496	3.685964	0.077699
24hdehyd_stem_ST	HSP90-02	28.51739	28.80398	28.8155	0.168884	28.71229	22.45687	6.255423	6.996667	-0.74124	1.671616
6hdehyd_leaf_ST	HSP90-02	26.50151	26.98842	27.14805	0.336792	26.87932	22.41561	4.463709	0.844496	3.619213	0.081378
6hdehyd_stem_ST	HSP90-02	26.71159	26.95373	27.05001	0.174368	26.90511	25.3931	1.512008	8.149632	-6.63762	99.56895
1hdehyd_leaf_ST	HSP90-03	30.94935	30.98024	30.80831	0.091655	30.91263	25.42716	5.485473	-4.23746	9.722929	0.001183
1hdehyd_stem_ST	HSP90-03	28.83676	29.08653	28.85088	0.140307	28.92472	22.32045	6.604277	4.366667	2.237611	0.212037
24hdehyd_leaf_ST	HSP90-03	27.5022	27.34631	27.8679	0.267734	27.57214	22.32045	5.251691	-4.23746	9.489147	0.001391
24hdehyd_stem_ST	HSP90-03	30.42485	30.88528	30.81874	0.248853	30.70962	22.45687	8.252758	8.502637	-0.24988	1.189107
6hdehyd_leaf_ST	HSP90-03	34.52968	31.08104	32.40417	1.739808	32.67163	22.41561	10.25602	-4.23746	14.49347	4.34E-05
6hdehyd_stem_ST	HSP90-03	33.17757	Undetermined	Undetermined	#DIV/0!	33.17757	25.3931	7.784466	11.85026	-4.0658	16.74662
1hdehyd_leaf_ST	HSP90-04	25.87383	25.72547	26.19626	0.2407	25.93186	25.42716	0.504695	-0.9588	1.463499	0.362613
1hdehyd_stem_ST	HSP90-04	34.95469	34.9197	35.90403	0.558476	35.25947	22.32045	12.93903	5.921077	7.017949	0.007716
24hdehyd_leaf_ST	HSP90-04	27.7945	27.72374	28.42882	0.388266	27.98235	22.32045	5.661907	-0.9588	6.620711	0.010162
24hdehyd_stem_ST	HSP90-04	32.43135	32.31139	33.42099	0.608958	32.72124	22.45687	10.26438	13.59211	-3.32773	10.04029

6hdehyd_leaf_ST	HSP90-04	31.6191	32.21506	33.17901	0.787158	32.33772	22.41561	9.922107	-0.9588	10.88091	0.00053
6hdehyd_stem_ST	HSP90-04	29.35702	30.4772	30.10041	0.569999	29.97821	25.3931	4.585106	13.59211	-9.007	514.491
1hdehyd_leaf_ST	HSP90-05	30.46435	30.23656	Undetermined	0.161068	30.35046	25.42716	4.923295	-2.74485	7.668147	0.004917
1hdehyd_stem_ST	HSP90-05	28.13186	28.39304	27.68072	0.360356	28.06854	22.32045	5.748093	7.276778	-1.52868	2.885226
24hdehyd_leaf_ST	HSP90-05	23.68166	22.90297	23.30608	0.389426	23.2969	22.32045	0.976459	-2.74485	3.721311	0.075818
24hdehyd_stem_ST	HSP90-05	26.26523	27.07989	27.48732	0.622251	26.94415	22.45687	4.48728	7.276778	-2.7895	6.913888
6hdehyd_leaf_ST	HSP90-05	24.34508	24.47895	24.85045	0.261834	24.55816	22.41561	2.142545	-2.74485	4.887397	0.033787
6hdehyd_stem_ST	HSP90-05	23.85489	24.65246	24.65093	0.460037	24.38609	25.3931	-1.00701	7.276778	-8.28379	311.6515
1hdehyd_leaf_ST	HSP90-09	24.70468	25.5364	26.07283	0.689367	25.43797	25.42716	0.010813	2.00304	-1.99223	3.978508
1hdehyd_stem_ST	HSP90-09	26.89708	26.73473	26.9209	0.101309	26.8509	22.32045	4.53046	7.863667	-3.33321	10.07849
24hdehyd_leaf_ST	HSP90-09	28.51739	28.80398	28.8155	0.168884	28.71229	22.32045	6.391843	2.00304	4.388803	0.047735
24hdehyd_stem_ST	HSP90-09	26.50151	26.98842	27.14805	0.336792	26.87932	22.45687	4.422457	8.6667	-4.24424	18.95154
6hdehyd_leaf_ST	HSP90-09	26.71159	26.95373	27.05001	0.174368	26.90511	22.41561	4.489496	2.00304	2.486456	0.178444
6hdehyd_stem_ST	HSP90-09	30.94935	30.98024	30.80831	0.091655	30.91263	25.3931	5.519532	1.6667	3.852832	0.069212
1hdehyd_leaf_ST	sHSP-02	28.83676	29.08653	28.85088	0.140307	28.92472	25.42716	3.497562	-0.52198	4.019546	0.061659
1hdehyd_stem_ST	sHSP-02	29.67634	29.93033	29.40471	0.262857	29.67046	22.32045	7.350012	3.203407	4.146605	0.056461
24hdehyd_leaf_ST	sHSP-02	28.05428	28.14797	28.44382	0.203327	28.21536	22.32045	5.89491	-0.52198	6.416895	0.011704
24hdehyd_stem_ST	sHSP-02	28.17445	27.52642	27.32367	0.444385	27.67485	22.45687	5.21798	3.203407	2.014573	0.247487
6hdehyd_leaf_ST	sHSP-02	26.83395	27.43641	27.49717	0.366631	27.25585	22.41561	4.840233	-0.52198	5.362218	0.024311
6hdehyd_stem_ST	sHSP-02	27.83793	27.71544	26.51766	0.729471	27.35701	25.3931	1.963907	3.203407	-1.2395	2.361166
1hdehyd_leaf_ST	sHSP-07	34.44129	32.11224	32.2856	1.297531	32.94638	25.42716	7.519216	2.00304	5.516176	0.021851
1hdehyd_stem_ST	sHSP-07	31.15671	30.7942	29.16653	1.059994	30.37248	22.32045	8.052037	7.863667	0.188371	0.877596
24hdehyd_leaf_ST	sHSP-07	32.69386	35.34673	34.83886	1.40811	34.29315	22.32045	11.97271	2.00304	9.969666	0.000997
24hdehyd_stem_ST	sHSP-07	36.22703	35.44515	34.5129	0.858164	35.39503	22.45687	12.93816	8.6667	4.27146	0.05178
6hdehyd_leaf_ST	sHSP-07	32.47515	32.08386	32.57673	0.26024	32.37858	22.41561	9.962967	2.00304	7.959927	0.004016
6hdehyd_stem_ST	sHSP-07	28.03961	27.79799	28.3514	0.277443	28.063	25.3931	2.669898	1.6667	1.003198	0.498893
1hdehyd_leaf_ST	sHSP-08	33.37866	33.66043	34.20563	0.420419	33.74824	25.42716	8.321079	-0.52198	8.843064	0.002178
1hdehyd_stem_ST	sHSP-08	33.28052	32.77514	32.56901	0.366092	32.87489	22.32045	10.55444	3.203407	7.351038	0.006125
24hdehyd_leaf_ST	sHSP-08	25.45696	24.80629	24.16276	0.647102	24.80867	22.32045	2.488224	-0.52198	3.010208	0.124119
24hdehyd_stem_ST	sHSP-08	24.69885	24.69057	25.34372	0.374731	24.91105	22.45687	2.45418	3.203407	-0.74923	1.680892
6hdehyd_leaf_ST	sHSP-08	25.55366	25.44615	25.33229	0.110699	25.44403	22.41561	3.028418	-0.52198	3.550403	0.085354

6hdehyd_stem_ST	sHSP-08	34.74134	34.5789	34.4329	0.154295	34.58438	25.3931	9.191281	2.00304	7.188241	0.006857
1hdehyd_leaf_ST	sHSP-11	26.74545	26.51623	26.85495	0.172852	26.70554	25.42716	1.278383	7.863667	-6.58528	96.02135
1hdehyd_stem_ST	sHSP-11	26.34935	26.00218	28.30709	1.242704	26.88621	22.32045	4.565762	2.00304	2.562722	0.169256
24hdehyd_leaf_ST	sHSP-11	26.20616	26.34653	26.12397	0.112543	26.22555	22.32045	3.905109	8.6667	-4.76159	27.12575
24hdehyd_stem_ST	sHSP-11	26.09673	26.03325	26.59786	0.309288	26.24261	22.45687	3.785747	2.00304	1.782707	0.290638
6hdehyd_leaf_ST	sHSP-11	32.15436	34.38535	32.81613	1.145843	33.11861	22.41561	10.703	1.6667	9.0363	0.001905
6hdehyd_stem_ST	sHSP-11	28.38702	28.34654	28.40567	0.03023	28.37974	25.3931	2.986641	-0.52198	3.508625	0.087861
1hdehyd_leaf_ST	sHSP-15	30.9068	31.55379	31.41587	0.340779	31.29215	25.42716	5.864992	3.203407	2.661585	0.158046
1hdehyd_stem_ST	sHSP-15	27.53911	27.7411	27.40966	0.167036	27.56329	22.32045	5.242844	-0.52198	5.764828	0.018391
24hdehyd_leaf_ST	sHSP-15	27.58104	27.67001	27.95358	0.194557	27.73488	22.32045	5.414431	3.203407	2.211024	0.215981
24hdehyd_stem_ST	sHSP-15	26.61405	27.3144	26.64956	0.394495	26.85933	22.45687	4.402468	-0.52198	4.924453	0.03293
6hdehyd_leaf_ST	sHSP-15	27.23138	27.6442	27.04202	0.307927	27.30587	22.41561	4.890252	2.00304	2.887212	0.135164
6hdehyd_stem_ST	sHSP-15	25.81419	25.66905	25.67532	0.082049	25.71952	25.3931	0.326419	7.863667	-7.53725	185.7537
1hdehyd_leaf_ST	sHSP-25	33.19505	34.90294	33.55143	0.900972	33.88314	25.42716	8.455977	2.00304	6.452937	0.011415
1hdehyd_stem_ST	sHSP-25	27.74905	28.16279	28.43245	0.344221	28.11476	22.32045	5.794315	8.6667	-2.87238	7.322746
24hdehyd_leaf_ST	sHSP-25	21.8062	21.73986	22.06723	0.173063	21.8711	22.32045	-0.44935	2.00304	-2.45239	5.473217
24hdehyd_stem_ST	sHSP-25	28.70565	28.34646	28.73429	0.216119	28.59547	22.45687	6.138603	1.6667	4.471903	0.045063
6hdehyd_leaf_ST	sHSP-25	32.62406	33.91491	31.38751	1.263797	32.64216	22.41561	10.22655	-0.52198	10.74853	0.000581
6hdehyd_stem_ST	sHSP-25	33.66085	33.94127	34.02463	0.190578	33.87558	25.3931	8.482479	3.203407	5.279072	0.025754
1hdehyd_leaf_ST	sHSP-26	26.74545	26.51623	26.85495	0.172852	26.70554	25.42716	1.278383	-0.52198	1.800368	0.287101
1hdehyd_stem_ST	sHSP-26	26.15495	25.76323	25.34481	0.405143	25.75433	22.32045	3.433885	3.203407	0.230478	0.852352
24hdehyd_leaf_ST	sHSP-26	26.34935	26.00218	28.30709	1.242704	26.88621	22.32045	4.565762	-0.52198	5.087747	0.029406
24hdehyd_stem_ST	sHSP-26	24.64348	24.7473	24.7208	0.053942	24.70386	22.45687	2.246995	2.00304	0.243955	0.844427
6hdehyd_leaf_ST	sHSP-26	24.98068	24.93107	24.96277	0.025124	24.95817	22.41561	2.54256	7.863667	-5.32111	39.97723
6hdehyd_stem_ST	sHSP-26	24.93211	25.80119	25.3775	0.434588	25.37027	25.3931	-0.02284	2.00304	-2.02588	4.072388
1hdehyd_leaf_ST	sHSP-27	31.66244	31.23182	30.90209	0.381287	31.26545	25.42716	5.838287	8.6667	-2.82841	7.102922
1hdehyd_stem_ST	sHSP-27	28.9174	30.99837	30.52771	1.091256	30.14783	22.32045	7.827384	2.00304	5.824344	0.017648
24hdehyd_leaf_ST	sHSP-27	30.92117	30.35887	32.60022	1.166123	31.29342	22.32045	8.972973	1.6667	7.306273	0.006318
24hdehyd_stem_ST	sHSP-27	35.27097	34.9223	32.72495	1.380346	34.30608	22.45687	11.84921	-0.52198	12.37119	0.000189
6hdehyd_leaf_ST	sHSP-27	34.73101	33.23652	35.09171	0.983643	34.35308	22.41561	11.93747	3.203407	8.734062	0.002348
6hdehyd_stem_ST	sHSP-27	33.0382	33.11002	33.50274	0.250058	33.21699	25.3931	7.823884	-0.52198	8.345868	0.003074

1hdehyd_leaf_ST	sHSP-30	35.60633	33.48606	32.51612	1.580386	33.8695	25.42716	8.44234	3.203407	5.238933	0.02648
1hdehyd_stem_ST	sHSP-30	29.70767	27.82071	27.70324	1.124885	28.41054	22.32045	6.090092	-0.52198	6.612076	0.010223
24hdehyd_leaf_ST	sHSP-30	33.52704	33.03802	32.9831	0.299454	33.18272	22.32045	10.86227	2.00304	8.859235	0.002153
24hdehyd_stem_ST	sHSP-30	29.5592	29.82302	29.5971	0.142636	29.65977	22.45687	7.202907	1.6667	5.536207	0.021549
6hdehyd_leaf_ST	sHSP-30	32.15236	32.54024	31.91099	0.317454	32.2012	22.41561	9.785582	-0.52198	10.30757	0.000789
6hdehyd_stem_ST	sHSP-30	31.02851	30.57032	26.66107	2.400231	29.41997	25.3931	4.026865	3.203407	0.823458	0.565086
**Susceptible cultivar in response to dehydration stress**											
**SAMPLE**	**DETECTOR**	**Ct1**	**Ct2**	**Ct3**	**sd**	**Avg Ct**	**End CTl**	**dCt**	**CTL0h**	**ddCt**	**Log Ct**
1hdehyd_leaf_SS	ACT	24.59753	24.03336	24.76292	0.382512	24.4646	24.4646	0		0	1
1hdehyd_stem_SS	ACT	21.3247	21.74624	21.43174	0.219112	21.50089	21.50089	0		0	1
24hdehyd_stem_SS	ACT	24.9295	24.73612	24.53213	0.198706	24.73258	24.73258	0		0	1
6hdehyd_leaf_SS	ACT	23.90869	24.35143	24.05428	0.22565	24.1048	24.73258	0		0	1
6hdehyd_stem_SS	ACT	25.03057	24.62624	24.87519	0.203962	24.844	24.844	0		0	1
1hdehyd_leaf_SS	HSP100-07	33.05629	33.68501	34.04718	0.501389	33.59616	24.4646	9.131557	4.256441	4.875116	0.034076
1hdehyd_stem_SS	HSP100-07	33.35494	33.90682	35.30143	1.003192	34.18773	21.50089	12.68684	7.113136	5.5737	0.020997
24hdehyd_leaf_SS	HSP100-07	28.36953	29.3157	29.80355	0.729117	29.16293	21.50089	7.662032	4.256441	3.405591	0.094366
24hdehyd_stem_SS	HSP100-07	27.70894	27.78297	28.38883	0.373005	27.96025	24.73258	3.227667	7.113136	-3.88547	14.77893
6hdehyd_leaf_SS	HSP100-07	33.93436	33.28621	33.42141	0.341932	33.54733	24.73258	8.814745	4.256441	4.558303	0.042444
6hdehyd_stem_SS	HSP100-07	30.5948	30.09433	31.12007	0.512923	30.60307	24.844	5.759068	7.113136	-1.35407	2.55632
1hdehyd_leaf_SS	HSP100-08	30.46845	30.55067	30.77137	0.156645	30.59683	24.4646	6.132226	2.530401	3.601825	0.082365
1hdehyd_stem_SS	HSP100-08	25.85951	26.10044	26.21724	0.18242	26.05906	21.50089	4.558169	7.268533	-2.71036	6.544868
24hdehyd_leaf_SS	HSP100-08	30.50165	30.31783	29.93725	0.287862	30.25224	21.50089	8.75135	2.530401	6.22095	0.013406
24hdehyd_stem_SS	HSP100-08	30.2281	29.97965	30.34102	0.184876	30.18292	24.73258	5.450341	7.268533	-1.81819	3.52639
6hdehyd_leaf_SS	HSP100-08	31.62919	33.3103	32.18398	0.856596	32.37449	24.73258	7.641908	2.530401	5.111507	0.028926
6hdehyd_stem_SS	HSP100-08	26.81703	25.78814	26.38122	0.516443	26.3288	24.844	1.4848	7.268533	-5.78373	55.09058
1hdehyd_leaf_SS	HSP100-11	26.37807	25.54799	25.6908	0.443804	25.87229	24.4646	1.407686	8.080248	-6.67256	102.0097
1hdehyd_stem_SS	HSP100-11	24.02629	24.02266	22.68998	0.770474	23.57965	21.50089	2.078751	10.47297	-8.39421	336.4421
24hdehyd_leaf_SS	HSP100-11	29.0272	28.24851	29.26001	0.529728	28.84524	21.50089	7.344345	8.080248	-0.7359	1.66544
24hdehyd_stem_SS	HSP100-11	22.62301	22.88767	22.73094	0.13308	22.74721	24.73258	-1.98537	1.5203	-3.50567	11.35828
6hdehyd_leaf_SS	HSP100-11	20.69603	20.57241	20.70178	0.073089	20.65674	24.73258	-4.07584	0.8	-4.87584	29.36124

6hdehyd_stem_SS	HSP100-11	22.01063	22.35659	22.21095	0.1737	22.19273	24.844	-2.65127	4.7297	-7.38097	166.684
1hdehyd_leaf_SS	HSP100-12	29.242	29.29919	29.63108	0.210082	29.39075	24.4646	4.92615	11.20091	-6.27476	77.42647
1hdehyd_stem_SS	HSP100-12	28.26917	28.19065	28.32106	0.065657	28.26029	21.50089	6.759398	12.39666	-5.63726	49.77186
24hdehyd_leaf_SS	HSP100-12	22.74581	22.75136	22.82894	0.046474	22.77537	21.50089	1.274475	4.19763	-2.92316	7.585031
24hdehyd_stem_SS	HSP100-12	26.5793	26.78285	26.92069	0.171749	26.76095	24.73258	2.028368	3.9666	-1.93823	3.832358
6hdehyd_leaf_SS	HSP100-12	29.25862	28.50294	28.43503	0.457158	28.7322	24.73258	3.999617	2.0091	1.990517	0.251649
6hdehyd_stem_SS	HSP100-12	33.90617	34.85355	34.68067	0.504526	34.48013	24.844	9.636131	12.39666	-2.76052	6.776427
1hdehyd_leaf_SS	HSP100-16	33.05629	33.68501	34.04718	0.501389	33.59616	24.4646	9.131557	5.374351	3.757207	0.073955
1hdehyd_stem_SS	HSP100-16	33.35494	33.90682	35.30143	1.003192	34.18773	21.50089	12.68684	7.333867	5.352969	0.024468
24hdehyd_leaf_SS	HSP100-16	28.36953	29.3157	29.80355	0.729117	29.16293	21.50089	7.662032	5.374351	2.287682	0.204804
24hdehyd_stem_SS	HSP100-16	27.70894	27.78297	28.38883	0.373005	27.96025	24.73258	3.227667	7.333867	-4.1062	17.22223
6hdehyd_leaf_SS	HSP100-16	33.93436	33.28621	33.42141	0.341932	33.54733	24.73258	8.814745	5.374351	3.440394	0.092117
6hdehyd_stem_SS	HSP100-16	30.5948	30.09433	31.12007	0.512923	30.60307	24.844	5.759068	7.333867	-1.5748	2.97894
1hdehyd_leaf_SS	HSP100-18	30.46845	30.55067	30.77137	0.156645	30.59683	24.4646	6.132226	5.008038	1.124188	0.45876
1hdehyd_stem_SS	HSP100-18	25.85951	26.10044	26.21724	0.18242	26.05906	21.50089	4.558169	7.268533	-2.71036	6.544868
24hdehyd_leaf_SS	HSP100-18	30.50165	30.31783	29.93725	0.287862	30.25224	21.50089	8.75135	5.008038	3.743312	0.074671
24hdehyd_stem_SS	HSP100-18	30.2281	29.97965	30.34102	0.184876	30.18292	24.73258	5.450341	7.268533	-1.81819	3.52639
6hdehyd_leaf_SS	HSP100-18	27.59856	27.96668	27.98864	0.219148	27.85129	24.73258	3.118712	5.008038	-1.88933	3.70462
6hdehyd_stem_SS	HSP100-18	29.96286	30.51229	30.21961	0.274907	30.23159	24.844	5.387589	2.68593	2.701659	0.153716
1hdehyd_leaf_SS	HSP100-20	26.93098	26.15485	26.97079	0.46002	26.68554	24.4646	2.220939	9.563736	-7.3428	162.3312
1hdehyd_stem_SS	HSP100-20	29.98253	29.19361	28.50124	0.74117	29.2258	21.50089	7.724901	4.785678	2.939224	0.130378
24hdehyd_leaf_SS	HSP100-20	26.78601	25.70643	25.76537	0.606999	26.08593	21.50089	4.585041	9.563736	-4.9787	31.53091
24hdehyd_stem_SS	HSP100-20	29.91775	29.85362	29.98916	0.067801	29.92018	24.73258	5.187596	4.785678	0.401919	0.756851
6hdehyd_leaf_SS	HSP100-20	26.8879	26.8054	26.85665	0.041655	26.84998	24.73258	2.117402	1.1087	1.008702	0.496993
6hdehyd_stem_SS	HSP100-20	28.29796	28.42448	28.40741	0.068653	28.37662	24.844	3.532621	4.785678	-1.25306	2.383458
1hdehyd_leaf_SS	HSP60-02	28.08393	28.58584	28.67753	0.31955	28.4491	24.4646	3.984498	5.960718	-1.97622	3.934609
1hdehyd_stem_SS	HSP60-02	31.73524	31.95582	31.37143	0.295108	31.6875	21.50089	10.1866	10.27932	-0.09272	1.06638
24hdehyd_leaf_SS	HSP60-02	23.21539	23.84487	23.40509	0.322916	23.48845	21.50089	1.987555	5.960718	-3.97316	15.70512
24hdehyd_stem_SS	HSP60-02	33.57513	33.67522	33.69787	0.065315	33.6494	24.73258	8.916824	10.27932	-1.3625	2.571302
6hdehyd_leaf_SS	HSP60-02	24.20753	24.29103	24.44807	0.122129	24.31554	24.73258	-0.41704	5.960718	-6.37776	83.15643
6hdehyd_stem_SS	HSP60-02	28.17844	28.31796	27.93915	0.191582	28.14518	24.844	3.301187	10.27932	-6.97814	126.0748

1hdehyd_leaf_SS	HSP60-03	30.68306	30.26536	30.66324	0.235643	30.53722	24.4646	6.072616	11.58692	-5.51431	45.70586
1hdehyd_stem_SS	HSP60-03	33.31495	Undetermined	33.46542	0.106401	33.39019	21.50089	11.88929	9.741697	2.147596	0.225688
24hdehyd_leaf_SS	HSP60-03	25.34345	25.59441	24.9851	0.306231	25.30765	21.50089	3.806759	11.58692	-7.78017	219.8179
24hdehyd_stem_SS	HSP60-03	34.47568	34.07215	34.91973	0.423951	34.48918	24.73258	9.756603	9.741697	0.014906	0.989721
6hdehyd_leaf_SS	HSP60-03	26.07913	26.23302	26.00517	0.116239	26.10577	24.73258	1.373191	5.896233	-4.52304	22.99171
6hdehyd_stem_SS	HSP60-03	Undetermined	27.59643	27.00661	0.417066	27.30152	24.844	2.457523	9.741697	-7.28417	155.8673
1hdehyd_leaf_SS	HSP60-05	28.68059	29.13791	29.66096	0.490553	29.15982	24.4646	4.695216	4.621545	0.073671	0.950217
1hdehyd_stem_SS	HSP60-05	29.91423	30.92076	30.13197	0.529578	30.32232	21.50089	8.821425	4.30362	4.517805	0.043652
24hdehyd_leaf_SS	HSP60-05	25.78682	25.75318	25.89259	0.072747	25.81086	21.50089	4.309969	4.621545	-0.31158	1.241062
24hdehyd_stem_SS	HSP60-05	31.51789	32.80726	31.45136	0.764344	31.9255	24.73258	7.192922	4.30362	2.889302	0.134969
6hdehyd_leaf_SS	HSP60-05	26.45857	26.42566	26.59039	0.087171	26.49154	24.73258	1.758958	4.621545	-2.86259	7.273182
6hdehyd_stem_SS	HSP60-05	29.77603	29.42248	29.96909	0.277204	29.72253	24.844	4.878535	4.30362	0.574915	0.671326
1hdehyd_leaf_SS	HSP60-09	Undetermined	Undetermined	34.9223	#DIV/0!	34.9223	24.4646	10.4577	2.370853	8.086844	0.003678
1hdehyd_stem_SS	HSP60-09	34.12444	Undetermined	34.20323	0.055716	34.16383	21.50089	12.66294	4.484283	8.178655	0.003451
24hdehyd_leaf_SS	HSP60-09	31.45136	35.27097	Undetermined	2.700872	33.36117	21.50089	11.86027	2.370853	9.489421	0.001391
24hdehyd_stem_SS	HSP60-09	35.09171	31.51789	32.80726	1.809852	33.13895	24.73258	8.406371	4.484283	3.922088	0.065968
6hdehyd_leaf_SS	HSP60-09	35.27097	34.9223	32.72495	1.380346	34.30608	24.73258	9.573495	2.370853	7.202642	0.006789
6hdehyd_stem_SS	HSP60-09	34.73101	33.23652	35.09171	0.983643	34.35308	24.844	9.509085	4.484283	5.024802	0.030717
1hdehyd_leaf_SS	HSP60-13	33.0382	33.11002	33.50274	0.250058	33.21699	24.4646	8.752382	1.320383	7.431999	0.005791
1hdehyd_stem_SS	HSP60-13	35.60633	33.48606	32.51612	1.580386	33.8695	21.50089	12.36861	2.556249	9.812357	0.001112
24hdehyd_leaf_SS	HSP60-13	29.70767	27.82071	27.70324	1.124885	28.41054	21.50089	6.909643	1.320383	5.58926	0.020771
24hdehyd_stem_SS	HSP60-13	33.52704	33.03802	32.9831	0.299454	33.18272	24.73258	8.450139	2.556249	5.89389	0.016818
6hdehyd_leaf_SS	HSP60-13	29.5592	29.82302	29.5971	0.142636	29.65977	24.73258	4.927192	1.320383	3.606809	0.082081
6hdehyd_stem_SS	HSP60-13	32.15236	32.54024	31.91099	0.317454	32.2012	24.844	7.357198	2.556249	4.800948	0.035873
1hdehyd_leaf_SS	HSP60-14	31.02851	30.57032	26.66107	2.400231	29.41997	24.4646	4.955364	1.986789	2.968574	0.127753
1hdehyd_stem_SS	HSP60-14	30.92117	30.35887	32.60022	1.166123	31.29342	21.50089	9.792525	8.863524	0.929001	0.525222
24hdehyd_leaf_SS	HSP60-14	26.563	26.7531	26.6668	0.095183	26.66097	21.50089	5.160072	1.986789	3.173283	0.110853
24hdehyd_stem_SS	HSP60-14	32.83416	31.90778	32.17202	0.47722	32.30465	24.73258	7.572072	8.863524	-1.29145	2.447743
6hdehyd_leaf_SS	HSP60-14	28.0561	28.37367	29.25517	0.621244	28.56164	24.73258	3.829064	1.986789	1.842275	0.278882
6hdehyd_stem_SS	HSP60-14	30.69705	31.02624	30.853	0.164673	30.85876	24.844	6.014767	8.863524	-2.84876	7.203792
1hdehyd_leaf_SS	HSP60-16	30.72092	31.02899	31.34821	0.313664	31.03271	24.4646	6.568103	3.760658	2.807444	0.142848

1hdehyd_stem_SS	HSP60-16	29.73103	30.95524	31.33834	0.839535	30.67487	21.50089	9.173974	5.297616	3.876358	0.068093
24hdehyd_leaf_SS	HSP60-16	26.73174	26.95258	26.74114	0.124873	26.80849	21.50089	5.307594	3.760658	1.546936	0.342236
24hdehyd_stem_SS	HSP60-16	31.77611	31.79769	31.62458	0.094338	31.73279	24.73258	7.000213	5.297616	1.702597	0.307233
6hdehyd_leaf_SS	HSP60-16	28.03131	31.71158	29.5749	1.848083	29.77259	24.73258	5.040014	3.760658	1.279356	0.411979
6hdehyd_stem_SS	HSP60-16	30.85674	30.61984	31.05035	0.21562	30.84231	24.844	5.998315	5.297616	0.700699	0.615274
1hdehyd_leaf_SS	HSP60-20	31.59664	32.31396	32.067	0.364412	31.99253	24.4646	7.527931	6.426404	1.101526	0.466023
1hdehyd_stem_SS	HSP60-20	Undetermined	35.01448	Undetermined	#DIV/0!	35.01448	21.50089	13.51359	5.109433	8.404157	0.002952
24hdehyd_leaf_SS	HSP60-20	27.16178	32.0122	25.6173	3.336833	28.26376	21.50089	6.762867	6.426404	0.336463	0.791981
24hdehyd_stem_SS	HSP60-20	35.70366	Undetermined	Undetermined	#DIV/0!	35.70366	24.73258	10.97107	5.109433	5.861641	0.017198
6hdehyd_leaf_SS	HSP60-20	Undetermined	33.73105	35.99252	1.599099	34.86179	24.73258	10.12921	6.426404	3.702801	0.076797
6hdehyd_stem_SS	HSP60-20	32.98121	33.89576	34.38222	0.711319	33.75306	24.844	8.909065	5.109433	3.799631	0.071812
1hdehyd_leaf_SS	HSP70-01	29.65759	29.92016	29.67707	0.146295	29.75161	24.4646	5.287004	5.444796	-0.15779	1.115579
1hdehyd_stem_SS	HSP70-01	29.01649	28.17854	28.60578	0.419002	28.60027	21.50089	7.099373	4.265441	2.833932	0.14025
24hdehyd_leaf_SS	HSP70-01	25.67391	25.98591	25.34668	0.319643	25.66883	21.50089	4.167939	5.444796	-1.27686	2.423106
24hdehyd_stem_SS	HSP70-01	30.87603	30.88447	30.53872	0.197228	30.7664	24.73258	6.033824	4.265441	1.768383	0.293538
6hdehyd_leaf_SS	HSP70-01	26.9416	26.69354	26.95652	0.147714	26.86388	24.73258	2.131304	5.444796	-3.31349	9.941694
6hdehyd_stem_SS	HSP70-01	28.73019	28.65694	29.00513	0.183569	28.79742	24.844	3.953422	4.265441	-0.31202	1.241444
1hdehyd_leaf_SS	HSP70-04	28.41702	28.94089	28.73528	0.263945	28.69773	24.4646	4.233126	1.272139	2.960987	0.128426
1hdehyd_stem_SS	HSP70-04	30.76478	30.5011	29.55434	0.636534	30.2734	21.50089	8.772509	7.406306	1.366203	0.387911
24hdehyd_leaf_SS	HSP70-04	23.73397	23.96059	23.97176	0.13418	23.88877	21.50089	2.38788	1.272139	1.11574	0.461454
24hdehyd_stem_SS	HSP70-04	29.99067	30.30667	30.94248	0.484776	30.41327	24.73258	5.680694	7.406306	-1.72561	3.307205
6hdehyd_leaf_SS	HSP70-04	24.86796	24.99712	25.17829	0.155888	25.01446	24.73258	0.281879	1.272139	-0.99026	1.986543
6hdehyd_stem_SS	HSP70-04	29.66367	29.09223	30.49269	0.704169	29.74953	24.844	4.905533	7.406306	-2.50077	5.659885
1hdehyd_leaf_SS	HSP70-06	21.35093	21.44282	21.58208	0.116382	21.45861	24.4646	-3.00599	9.314555	-12.3205	5115.108
1hdehyd_stem_SS	HSP70-06	23.66929	23.58276	23.62448	0.043275	23.62551	21.50089	2.124617	11.81958	-9.69496	828.8455
24hdehyd_leaf_SS	HSP70-06	30.21972	30.23488	30.49759	0.156235	30.3174	21.50089	8.816505	9.314555	-0.49805	1.412303
24hdehyd_stem_SS	HSP70-06	31.53533	31.32369	31.26651	0.141609	31.37518	24.73258	6.642597	11.81958	-5.17698	36.17645
6hdehyd_leaf_SS	HSP70-06	26.43199	26.50776	26.68833	0.131689	26.54269	24.73258	1.810113	9.314555	-7.50444	181.5775
6hdehyd_stem_SS	HSP70-06	28.13186	28.39304	27.68072	0.360356	28.06854	24.844	3.224541	11.81958	-8.59504	386.6904
1hdehyd_leaf_SS	HSP70-15	23.68166	22.90297	23.30608	0.389426	23.2969	24.4646	-1.1677	2.556249	-3.72395	13.21357
1hdehyd_stem_SS	HSP70-15	26.26523	27.07989	27.48732	0.622251	26.94415	21.50089	5.443252	2.968342	2.47491	0.179878

24hdehyd_leaf_SS	HSP70-15	24.34508	24.47895	24.85045	0.261834	24.55816	21.50089	3.057265	2.556249	0.501015	0.706609
24hdehyd_stem_SS	HSP70-15	23.85489	24.65246	24.65093	0.460037	24.38609	24.73258	-0.34649	2.968342	-3.31483	9.950944
6hdehyd_leaf_SS	HSP70-15	24.70468	25.5364	26.07283	0.689367	25.43797	24.73258	0.705393	2.556249	-1.85086	3.607143
6hdehyd_stem_SS	HSP70-15	25.86894	26.56777	26.7788	0.476224	26.40517	24.844	1.561174	2.968342	-1.40717	2.652162
1hdehyd_leaf_SS	HSP70-16	30.23967	31.14198	30.95139	0.475574	30.77768	24.4646	6.313075	8.545858	-2.23278	4.700398
1hdehyd_stem_SS	HSP70-16	31.50501	31.05158	31.0986	0.249324	31.21839	21.50089	9.717501	11.52239	-1.80489	3.494028
24hdehyd_leaf_SS	HSP70-16	30.15948	30.83181	30.99775	0.443893	30.66301	21.50089	9.16212	8.545858	0.616262	0.652359
24hdehyd_stem_SS	HSP70-16	27.20658	27.21153	26.93239	0.159751	27.11683	24.73258	2.38425	11.52239	-9.13814	563.4492
6hdehyd_leaf_SS	HSP70-16	26.74441	26.44323	26.17784	0.283472	26.45516	24.73258	1.722581	8.545858	-6.82328	113.2429
6hdehyd_stem_SS	HSP70-16	31.16855	32.03036	32.40502	0.634028	31.86798	24.844	7.023978	11.52239	-4.49841	22.60256
1hdehyd_leaf_SS	HSP70-19	30.47015	30.92189	30.24818	0.343323	30.54674	24.4646	6.082134	8.184944	-2.10281	4.295452
1hdehyd_stem_SS	HSP70-19	28.78678	28.71104	28.52616	0.134064	28.67466	21.50089	7.173767	11.34792	-4.17415	18.05278
24hdehyd_leaf_SS	HSP70-19	20.60145	21.39758	21.07269	0.400302	21.0239	21.50089	-0.47699	2.1396	-2.61659	6.132987
24hdehyd_stem_SS	HSP70-19	22.62301	22.88767	22.73094	0.13308	22.74721	24.73258	-1.98537	2.845	-4.83037	28.4503
6hdehyd_leaf_SS	HSP70-19	20.69603	20.57241	20.70178	0.073089	20.65674	24.73258	-4.07584	1.0289	-5.10474	34.40964
6hdehyd_stem_SS	HSP70-19	22.01063	22.35659	22.21095	0.1737	22.19273	24.844	-2.65127	8.100045	-10.7513	1723.729
1hdehyd_leaf_SS	HSP70-21	29.242	29.29919	29.63108	0.210082	29.39075	24.4646	4.92615	4.361949	0.564201	0.67633
1hdehyd_stem_SS	HSP70-21	28.26917	28.19065	28.32106	0.065657	28.26029	21.50089	6.759398	7.347949	-0.58855	1.503736
24hdehyd_leaf_SS	HSP70-21	22.74581	22.75136	22.82894	0.046474	22.77537	21.50089	1.274475	4.361949	-3.08747	8.500066
24hdehyd_stem_SS	HSP70-21	34.56589	33.09659	32.19814	1.195287	33.28688	24.73258	8.554295	7.347949	1.206346	0.433365
6hdehyd_leaf_SS	HSP70-21	28.23629	27.68901	28.18109	0.301304	28.03546	24.73258	3.302879	4.361949	-1.05907	2.083587
6hdehyd_stem_SS	HSP70-21	27.6488	27.56945	28.29785	0.399609	27.8387	24.844	2.9947	3.47949	-0.48479	1.399382
1hdehyd_leaf_SS	HSP70-24	33.64226	35.37273	35.82899	1.153579	34.94799	24.4646	10.48339	4.534038	5.94935	0.016183
1hdehyd_stem_SS	HSP70-24	27.79851	28.95956	29.30918	0.790819	28.68908	21.50089	7.18819	7.550413	-0.36222	1.285406
24hdehyd_leaf_SS	HSP70-24	26.21585	28.01458	27.90528	1.008427	27.37857	21.50089	5.877675	4.534038	1.343637	0.394026
24hdehyd_stem_SS	HSP70-24	30.14827	29.82715	29.40978	0.370291	29.79507	24.73258	5.062485	7.550413	-2.48793	5.60972
6hdehyd_leaf_SS	HSP70-24	Undetermined	34.08273	32.88809	0.844738	33.48541	24.73258	8.75283	4.534038	4.218792	0.053705
6hdehyd_stem_SS	HSP70-24	24.92578	25.73759	25.19826	0.413149	25.28721	24.844	0.443213	7.550413	-7.1072	137.8734
1hdehyd_leaf_SS	HSP90-02	24.46081	24.95078	24.83125	0.25547	24.74761	24.4646	0.283009	8.498686	-8.21568	297.2797
1hdehyd_stem_SS	HSP90-02	32.90216	33.093	32.73766	0.177832	32.91094	21.50089	11.41004	3.73102	7.679025	0.00488
24hdehyd_leaf_SS	HSP90-02	30.32365	28.96292	28.90736	0.802141	29.39798	21.50089	7.897083	8.498686	-0.6016	1.517402

24hdehyd_stem_SS	HSP90-02	26.08532	26.45027	26.5168	0.232303	26.3508	24.73258	1.618216	5.10741	-3.48919	11.22928
6hdehyd_leaf_SS	HSP90-02	25.89848	26.21016	25.62507	0.292756	25.91124	24.73258	1.178656	8.498686	-7.32003	159.7897
6hdehyd_stem_SS	HSP90-02	32.41091	33.14599	33.48542	0.549261	33.0141	24.844	8.170107	5.067315	3.102792	0.116404
1hdehyd_leaf_SS	HSP90-03	28.49288	28.32594	28.47364	0.091336	28.43082	24.4646	3.966218	5.039092	-1.07287	2.10362
1hdehyd_stem_SS	HSP90-03	28.76764	28.84623	28.95328	0.093183	28.85572	21.50089	7.354823	8.863524	-1.5087	2.845536
24hdehyd_leaf_SS	HSP90-03	25.01277	25.13462	25.14888	0.074811	25.09876	21.50089	3.597864	5.039092	-1.44123	2.71552
24hdehyd_stem_SS	HSP90-03	21.82279	21.82279	22.36743	0.31445	22.00433	24.73258	-2.72825	8.863524	-11.5918	3086.53
6hdehyd_leaf_SS	HSP90-03	24.90869	24.90869	22.88948	1.165792	24.23562	24.73258	-0.49696	5.039092	-5.53606	46.40013
6hdehyd_stem_SS	HSP90-03	23.65528	23.65528	22.57499	0.623702	23.29518	24.844	-1.54882	8.863524	-10.4123	1362.782
1hdehyd_leaf_SS	HSP90-04	Undetermined	22.46122	22.25774	0.143879	22.35948	24.4646	-2.10512	3.029978	-5.1351	35.14148
1hdehyd_stem_SS	HSP90-04	24.12278	24.12278	28.8091	2.705649	25.68489	21.50089	4.183992	7.234384	-3.05039	8.284372
24hdehyd_leaf_SS	HSP90-04	25.40308	25.40308	24.47053	0.53841	25.09223	21.50089	3.591337	3.029978	0.561359	0.677664
24hdehyd_stem_SS	HSP90-04	24.63038	25.27679	24.45398	0.433202	24.78705	24.73258	0.054469	7.234384	-7.17992	145.0006
6hdehyd_leaf_SS	HSP90-04	29.10174	27.67875	27.3989	0.913137	28.0598	24.73258	3.327218	3.029978	0.297239	0.813808
6hdehyd_stem_SS	HSP90-04	26.99183	26.4292	26.71834	0.28135	26.71312	24.844	1.869124	7.234384	-5.36526	41.21964
1hdehyd_leaf_SS	HSP90-05	28.38702	28.34654	28.40567	0.03023	28.37974	24.4646	3.91514	2.201825	1.713315	0.304959
1hdehyd_stem_SS	HSP90-05	30.9068	31.55379	31.41587	0.340779	31.29215	21.50089	9.791259	6.704037	3.087222	0.117667
24hdehyd_leaf_SS	HSP90-05	27.53911	27.7411	27.40966	0.167036	27.56329	21.50089	6.062395	2.201825	3.86057	0.068842
24hdehyd_stem_SS	HSP90-05	27.58104	27.67001	27.95358	0.194557	27.73488	24.73258	3.002296	6.704037	-3.70174	13.01173
6hdehyd_leaf_SS	HSP90-05	26.61405	27.3144	26.64956	0.394495	26.85933	24.73258	2.126753	2.201825	-0.07507	1.053413
6hdehyd_stem_SS	HSP90-05	27.23138	27.6442	27.04202	0.307927	27.30587	24.844	2.461868	6.704037	-4.24217	18.92431
1hdehyd_leaf_SS	HSP90-09	25.81419	25.66905	25.67532	0.082049	25.71952	24.4646	1.254918	9.670187	-8.41527	341.3881
1hdehyd_stem_SS	HSP90-09	33.19505	34.90294	33.55143	0.900972	33.88314	21.50089	12.38224	11.38556	0.996684	0.50115
24hdehyd_leaf_SS	HSP90-09	28.95706	30.60958	29.68297	0.828287	29.74987	21.50089	8.248979	9.670187	-1.42121	2.678097
24hdehyd_stem_SS	HSP90-09	26.5793	26.78285	26.92069	0.171749	26.76095	24.73258	2.028368	11.38556	-9.35719	655.836
6hdehyd_leaf_SS	HSP90-09	26.99183	26.4292	26.71834	0.28135	26.71312	24.73258	1.980541	9.670187	-7.68965	206.4496
6hdehyd_stem_SS	HSP90-09	23.99408	25.11066	25.48217	0.774508	24.8623	24.844	0.018307	11.38556	-11.3673	2641.702
1hdehyd_leaf_SS	sHSP-02	34.52968	31.08104	32.40417	1.739808	32.67163	24.4646	8.207028	7.727236	0.479792	0.717081
1hdehyd_stem_SS	sHSP-02	33.17757	Undetermined	Undetermined	#DIV/0!	33.17757	21.50089	11.67667	10.22697	1.449707	0.366096
24hdehyd_leaf_SS	sHSP-02	25.87383	25.72547	26.19626	0.2407	25.93186	21.50089	4.430961	7.727236	-3.29627	9.823756
24hdehyd_stem_SS	sHSP-02	34.95469	34.9197	35.90403	0.558476	35.25947	24.73258	10.52689	10.22697	0.299924	0.812295

6hdehyd_leaf_SS	sHSP-02	27.7945	27.72374	28.42882	0.388266	27.98235	24.73258	3.249772	7.727236	-4.47746	22.27671
6hdehyd_stem_SS	sHSP-02	32.43135	32.31139	33.42099	0.608958	32.72124	24.844	7.877247	10.22697	-2.34972	5.09725
1hdehyd_leaf_SS	sHSP-07	31.6191	32.21506	33.17901	0.787158	32.33772	24.4646	7.873118	10.98343	-3.11032	8.635711
1hdehyd_stem_SS	sHSP-07	29.35702	30.4772	30.10041	0.569999	29.97821	21.50089	8.477313	14.19922	-5.7219	52.77941
24hdehyd_leaf_SS	sHSP-07	34.18128	34.34284	34.57661	0.198761	34.36691	21.50089	12.86602	10.98343	1.882583	0.271198
24hdehyd_stem_SS	sHSP-07	31.63097	32.11416	31.79771	0.24543	31.84761	24.73258	7.115033	14.19922	-7.08418	135.6912
6hdehyd_leaf_SS	sHSP-07	32.67087	33.16291	32.64088	0.293122	32.82489	24.73258	8.092305	10.98343	-2.89113	7.418504
6hdehyd_stem_SS	sHSP-07	23.85489	24.65246	24.65093	0.460037	24.38609	24.844	-0.45791	14.19922	-14.6571	25836.53
1hdehyd_leaf_SS	sHSP-08	24.70468	25.5364	26.07283	0.689367	25.43797	24.4646	0.97337	6.28698	-5.31361	39.77004
1hdehyd_stem_SS	sHSP-08	26.89708	26.73473	26.9209	0.101309	26.8509	21.50089	5.350011	5.813431	-0.46342	1.378807
24hdehyd_leaf_SS	sHSP-08	28.51739	28.80398	28.8155	0.168884	28.71229	21.50089	7.211395	6.28698	0.924414	0.526894
24hdehyd_stem_SS	sHSP-08	26.50151	26.98842	27.14805	0.336792	26.87932	24.73258	2.146742	5.813431	-3.66669	12.69941
6hdehyd_leaf_SS	sHSP-08	26.71159	26.95373	27.05001	0.174368	26.90511	24.73258	2.172529	6.28698	-4.11445	17.32101
6hdehyd_stem_SS	sHSP-08	30.94935	30.98024	30.80831	0.091655	30.91263	24.844	6.068636	5.813431	0.255205	0.837868
1hdehyd_leaf_SS	sHSP-11	28.83676	29.08653	28.85088	0.140307	28.92472	24.4646	4.46012	4.291369	0.168751	0.889613
1hdehyd_stem_SS	sHSP-11	23.90869	24.35143	24.05428	0.22565	24.1048	21.50089	2.603904	4.853923	-2.25002	4.75689
24hdehyd_leaf_SS	sHSP-11	25.03057	24.62624	24.87519	0.203962	24.844	21.50089	3.343104	4.291369	-0.94826	1.929551
24hdehyd_stem_SS	sHSP-11	30.81909	30.88235	30.41424	0.253982	30.70523	24.73258	5.972645	4.853923	1.118722	0.460502
6hdehyd_leaf_SS	sHSP-11	33.52779	35.37207	33.76587	1.003159	34.22191	24.73258	9.489331	4.291369	5.197962	0.027243
6hdehyd_stem_SS	sHSP-11	31.97957	32.11514	32.6424	0.350172	32.2457	24.844	7.401702	4.853923	2.547779	0.171018
1hdehyd_leaf_SS	sHSP-15	34.18128	34.34284	34.57661	0.198761	34.36691	24.4646	9.902307	3.973532	5.928776	0.016416
1hdehyd_stem_SS	sHSP-15	31.63097	32.11416	31.79771	0.24543	31.84761	21.50089	10.34672	7.387156	2.959563	0.128553
24hdehyd_leaf_SS	sHSP-15	32.67087	33.16291	32.64088	0.293122	32.82489	21.50089	11.32399	3.973532	7.35046	0.006128
24hdehyd_stem_SS	sHSP-15	30.17745	30.55206	30.54793	0.215099	30.42582	24.73258	5.693235	7.387156	-1.69392	3.235348
6hdehyd_leaf_SS	sHSP-15	23.73833	24.67634	24.31203	0.472885	24.24223	24.73258	-0.49035	3.973532	-4.46388	22.06798
6hdehyd_stem_SS	sHSP-15	24.63038	25.27679	24.45398	0.433202	24.78705	24.844	-0.05695	7.387156	-7.4441	174.1401
1hdehyd_leaf_SS	sHSP-25	21.3247	21.74624	21.43174	0.219112	21.50089	24.4646	-2.96371	7.119162	-10.0829	1084.543
1hdehyd_stem_SS	sHSP-25	24.48857	23.81845	24.29907	0.345436	24.20203	21.50089	2.701136	5.10987	-2.40873	5.310083
24hdehyd_leaf_SS	sHSP-25	34.27634	33.99267	32.41239	1.004328	33.56047	21.50089	12.05957	7.119162	4.94041	0.032568
24hdehyd_stem_SS	sHSP-25	26.54079	26.66036	27.2404	0.374211	26.81385	24.73258	2.081269	11.28181	-9.20054	588.3522
6hdehyd_leaf_SS	sHSP-25	34.30904	34.30904	34.45161	0.082313	34.35657	24.73258	9.623987	-2.4587	12.08269	0.000231

6hdehyd_stem_SS	sHSP-25	32.80377	32.80377	34.83309	1.171627	33.48021	24.844	8.636212	2.00793	6.628282	0.010109
1hdehyd_leaf_SS	sHSP-26	35.27097	34.9223	32.72495	1.380346	34.30608	24.4646	9.841473	4.7897	5.051773	0.030148
1hdehyd_stem_SS	sHSP-26	34.73101	33.23652	35.09171	0.983643	34.35308	21.50089	12.85219	7.8581	4.994089	0.031378
24hdehyd_leaf_SS	sHSP-26	33.0382	33.11002	33.50274	0.250058	33.21699	21.50089	11.71609	4.7897	6.926391	0.008221
24hdehyd_stem_SS	sHSP-26	35.60633	33.48606	32.51612	1.580386	33.8695	24.73258	9.13692	4.1	5.03692	0.03046
6hdehyd_leaf_SS	sHSP-26	29.70767	27.82071	27.70324	1.124885	28.41054	24.73258	3.677957	4.7897	-1.11174	2.161066
6hdehyd_stem_SS	sHSP-26	33.52704	33.03802	32.9831	0.299454	33.18272	24.844	8.338722	10.522	-2.18328	4.541843
1hdehyd_leaf_SS	sHSP-27	29.5592	29.82302	29.5971	0.142636	29.65977	24.4646	5.19517	5.15657	0.0386	0.973599
1hdehyd_stem_SS	sHSP-27	32.15236	32.54024	31.91099	0.317454	32.2012	21.50089	10.7003	7.780998	2.919304	0.132191
24hdehyd_leaf_SS	sHSP-27	31.02851	30.57032	26.66107	2.400231	29.41997	21.50089	7.919073	5.15657	2.762503	0.147368
24hdehyd_stem_SS	sHSP-27	30.92117	30.35887	32.60022	1.166123	31.29342	24.73258	6.560838	7.780998	-1.22016	2.329725
6hdehyd_leaf_SS	sHSP-27	30.69705	31.02624	30.853	0.164673	30.85876	24.73258	6.126184	2.8156	3.310584	0.100789
6hdehyd_stem_SS	sHSP-27	30.72092	31.02899	31.34821	0.313664	31.03271	24.844	6.188708	7.780998	-1.59229	3.015275
1hdehyd_leaf_SS	sHSP-30	27.74905	28.16279	28.43245	0.344221	28.11476	24.4646	3.650158	4.7897	-1.13954	2.203111
1hdehyd_stem_SS	sHSP-30	21.8062	21.73986	22.06723	0.173063	21.8711	21.50089	0.370202	2.524207	-2.15401	4.450616
24hdehyd_leaf_SS	sHSP-30	28.70565	28.34646	28.73429	0.216119	28.59547	21.50089	7.094575	6.190665	0.903909	0.534437
24hdehyd_stem_SS	sHSP-30	32.62406	33.91491	31.38751	1.263797	32.64216	24.73258	7.90958	2.524207	5.385372	0.023924
6hdehyd_leaf_SS	sHSP-30	33.66085	33.94127	34.02463	0.190578	33.87558	24.73258	9.143	6.190665	2.952334	0.129199
6hdehyd_stem_SS	sHSP-30	27.66347	27.67238	27.90043	0.134309	27.74543	24.844	2.901429	6.190665	-3.28924	9.775949
**Tolerant cultivar in response to heat stress**											
**SAMPLE**	**DETECTOR**	**Ct1**	**Ct2**	**Ct3**	**sd**	**Avg Ct**	**End CTl**	**dCt**	**CTL0h**	**ddCt**	**Log Ct**
1hheat_leaf_ST	HSP100-07	26.55728	26.98822	27.18542	0.321237	26.91031	24.80167	2.108641	-1.7011	3.809742	0.071311
1hheat_stem_ST	HSP100-07	30.81909	30.88235	30.41424	0.253982	30.70523	25.76063	4.944596	1.011604	3.932992	0.065471
24hheat_leaf_ST	HSP100-07	33.52779	35.37207	33.76587	1.003159	34.22191	24.95838	9.263531	-1.7011	10.96463	0.0005
24hheat_stem_ST	HSP100-07	34.56396	34.55016	36.68255	1.22717	35.26555	25.67234	9.593215	1.011604	8.581611	0.00261
6hheat_leaf_ST	HSP100-07	26.99183	26.4292	26.71834	0.28135	26.71312	25.70032	1.012802	-1.7011	2.713903	0.152417
6hheat_stem_ST	HSP100-07	28.51739	28.80398	28.8155	0.168884	28.71229	23.2969	5.415385	4.256441	1.158943	0.44784
1hheat_leaf_ST	HSP100-08	26.02708	26.22297	26.50936	0.242551	26.25313	24.80167	1.451466	-5.52741	6.978876	0.007928
1hheat_stem_ST	HSP100-08	29.87155	29.86761	30.01728	0.085298	29.91881	25.76063	4.158182	0.086964	4.071218	0.05949
24hheat_leaf_ST	HSP100-08	33.85988	35.28003	33.37694	0.989261	34.17228	24.95838	9.213901	-5.52741	14.74131	3.65E-05

24hheat_stem_ST	HSP100-08	33.1767	33.80206	33.12407	0.377165	33.36761	25.67234	7.695269	0.086964	7.608305	0.005125
6hheat_leaf_ST	HSP100-08	24.14689	23.99715	24.06835	0.074898	24.0708	25.70032	-1.62952	-5.52741	3.897887	0.067084
6hheat_leaf_ST	HSP100-08	23.73833	24.67634	24.31203	0.472885	24.24223	29.76964	-5.52741	-5.52741	2.411	0.188025
6hheat_stem_ST	HSP100-08	28.51739	28.80398	28.8155	0.168884	28.71229	23.2969	5.415385	4.256441	1.158943	0.44784
1hheat_leaf_ST	HSP100-11	23.01842	23.51464	24.01229	0.496934	23.51511	24.80167	-1.28655	1.60665	-2.8932	7.429187
1hheat_stem_ST	HSP100-11	24.63038	25.27679	24.45398	0.433202	24.78705	25.76063	-0.97358	4.166584	-5.14016	35.26498
24hheat_leaf_ST	HSP100-11	29.10174	27.67875	27.3989	0.913137	28.0598	24.95838	3.101418	1.60665	1.494768	0.354838
24hheat_stem_ST	HSP100-11	30.74289	31.1356	30.6959	0.241444	30.85813	25.67234	5.185791	4.166584	1.019207	0.493387
6hheat_leaf_ST	HSP100-11	23.99408	25.11066	25.48217	0.774508	24.8623	25.70032	-0.83801	1.60665	-2.44467	5.443992
6hheat_leaf_ST	HSP100-11	30.42485	32.88528	30.81874	1.321578	31.37629	29.76964	1.60665	1.60665	2.411	0.188025
6hheat_stem_ST		26.36649	26.2802	26.27881	0.050227	26.3085	22.32045	3.988052	5.156522	-1.16847	2.247733
1hheat_leaf_ST	HSP100-12	32.82025	33.36525	33.35513	0.311775	33.18021	28.47389	4.706319	0.37819	4.328129	0.049786
1hheat_stem_ST	HSP100-12	36.75892	36.82159	Undetermined	0.044314	36.79026	29.7048	7.085453	3.597701	3.487752	0.089142
24hheat_leaf_ST	HSP100-12	36.60319	34.74488	34.25642	1.238225	35.2015	29.53552	5.665978	0.37819	5.287788	0.025599
24hheat_stem_ST	HSP100-12	36.5269	Undetermined	36.87495	0.246109	36.70093	28.69007	8.010854	3.597701	4.413153	0.046936
6hheat_leaf_ST	HSP100-12	36.47678	34.95816	36.45321	0.87005	35.96272	28.91894	7.043779	0.37819	6.665589	0.00985
6hheat_stem_ST	HSP100-12	28.9174	30.99837	30.52771	1.091256	30.14783	29.76964	0.37819	0.37819	2.662427	0.157954
1hheat_leaf_ST	HSP100-16	29.29169	28.94754	28.74875	0.274693	28.99599	28.47389	0.522102	-3.05802	3.580118	0.083614
1hheat_stem_ST	HSP100-16	32.12342	32.54173	32.93776	0.407219	32.5343	29.7048	2.829498	0.440807	2.388691	0.190956
24hheat_leaf_ST	HSP100-16	34.23447	34.05072	33.9184	0.158731	34.06787	29.53552	4.532349	-3.05802	7.590365	0.005189
24hheat_stem_ST	HSP100-16	34.06779	33.98118	33.58911	0.255068	33.87936	28.69007	5.189288	0.440807	4.748481	0.037202
6hheat_leaf_ST	HSP100-16	28.49868	28.67298	28.39839	0.138945	28.52335	28.91894	-0.39559	-3.05802	2.662427	0.157954
6hheat_stem_ST	HSP100-16	27.02491	26.50502	26.60494	0.275874	26.71162	29.76964	-3.05802	-3.05802	0	1
1hheat_leaf_ST	HSP100-18	26.20616	26.34653	26.12397	0.112543	26.22555	22.32045	3.905109	0.939984	2.965125	0.128059
1hheat_stem_ST	HSP100-18	26.89708	26.73473	26.9209	0.101309	26.8509	22.90618	3.944725	7.113136	-3.16841	8.990564
24hheat_leaf_ST	HSP100-18	28.51739	28.80398	28.8155	0.168884	28.71229	23.2969	5.415385	4.256441	1.158943	0.44784
24hheat_stem_ST	HSP100-18	26.50151	26.98842	27.14805	0.336792	26.87932	21.45946	5.419861	7.113136	-1.69328	3.233901
6hheat_leaf_ST	HSP100-18	26.71159	26.95373	27.05001	0.174368	26.90511	21.84457	5.060542	2.530401	2.530141	0.173122
6hheat_stem_ST	HSP100-18	30.94935	30.98024	30.80831	0.091655	30.91263	24.30848	6.604158	7.268533	-0.66438	1.584882
1hheat_leaf_ST	HSP100-20	28.83676	29.08653	28.85088	0.140307	28.92472	24.30848	4.616247	2.530401	2.085846	0.235558
1hheat_stem_ST	HSP100-20	26.50151	26.98842	27.14805	0.336792	26.87932	23.29518	3.58414	6.190665	-2.60653	6.090351

24hheat_leaf_ST	HSP100-20	26.71159	26.95373	27.05001	0.174368	26.90511	22.35948	4.545631	2.524207	2.021424	0.246315
24hheat_stem_ST	HSP100-20	30.94935	30.98024	30.80831	0.091655	30.91263	25.68489	5.227748	6.190665	-0.96292	1.949247
6hheat_leaf_ST	HSP100-20	28.83676	29.08653	28.85088	0.140307	28.92472	25.09223	3.832492	6.190665	-2.35817	5.127209
6hheat_stem_ST	HSP100-20	28.76764	28.84623	28.95328	0.093183	28.85572	21.45946	7.396255	7.268533	0.127722	0.915276
1hheat_leaf_ST	HSP60-02	29.23002	28.61839	28.75071	0.321801	28.86637	21.84457	7.021807	8.080248	-1.05844	2.082681
1hheat_stem_ST	HSP60-02	29.73103	30.95524	31.33834	0.839535	30.67487	24.30848	6.366392	10.47297	-4.10657	17.22669
24hheat_leaf_ST	HSP60-02	26.73174	26.95258	26.74114	0.124873	26.80849	24.30848	2.500012	8.080248	-5.58024	47.843
24hheat_stem_ST	HSP60-02	31.77611	31.79769	31.62458	0.094338	31.73279	22.90618	8.826613	10.47297	-1.64635	3.130412
6hheat_leaf_ST	HSP60-02	28.03131	31.71158	29.5749	1.848083	29.77259	23.2969	6.475691	8.080248	-1.60456	3.041025
6hheat_stem_ST	HSP60-02	26.99183	26.4292	26.71834	0.28135	26.71312	23.29518	3.417939	4.7897	-1.37176	2.587863
1hheat_leaf_ST	HSP60-03	26.55728	26.98822	27.18542	0.321237	26.91031	22.35948	4.55083	4.1	0.45083	0.731622
1hheat_stem_ST	HSP60-03	34.56396	34.55016	36.68255	1.22717	35.26555	25.68489	9.580668	4.7897	4.790968	0.036122
24hheat_leaf_ST	HSP60-03	34.18128	34.34284	34.57661	0.177777	34.36691	30.98837	3.37854	0.788017	2.590523	0.166026
24hheat_stem_ST	HSP60-03	31.63097	32.11416	31.79771	0.219519	31.84761	32.58468	-0.73707	-0.66351	-0.07356	1.052307
6hheat_leaf_ST	HSP60-03	32.67087	33.16291	32.64088	0.262176	32.82489	28.67466	4.150224	2.31406	1.836165	0.280065
6hheat_stem_ST	HSP60-03	25.74733	25.97733	25.93443	0.1223	25.88636	22.41561	3.470751	-6.41278	9.883529	0.001059
1hheat_leaf_ST	HSP60-05	28.22206	28.09266	28.31386	0.111133	28.20952	28.47389	-0.26437	2.219692	-2.48406	5.594687
1hheat_stem_ST	HSP60-05	28.33728	28.52878	29.98344	0.900236	28.94983	29.7048	-0.75497	1.382468	-2.13744	4.399795
24hheat_leaf_ST	HSP60-05	34.97513	33.28295	33.18544	1.006311	33.81451	29.53552	4.27899	2.219692	2.059299	0.239933
24hheat_stem_ST	HSP60-05	33.07391	32.38612	32.408	0.390932	32.62268	28.69007	3.932606	1.382468	2.550138	0.170739
6hheat_leaf_ST	HSP60-05	27.95778	27.99689	28.10234	0.074771	28.019	28.91894	-0.89994	2.219692	-3.11963	8.691645
6hheat_stem_ST	HSP60-05	30.94935	30.98024	30.80831	0.091655	30.91263	24.30848	6.604158	7.268533	-0.66438	1.584882
1hheat_leaf_ST	HSP60-09	30.50515	29.74861	29.4805	0.531376	29.91142	28.47389	1.43753	0.491405	0.946126	0.519024
1hheat_stem_ST	HSP60-09	31.80195	32.51987	32.51049	0.41181	32.27744	29.7048	2.572633	-4.49025	7.062883	0.007479
24hheat_leaf_ST	HSP60-09	35.52724	35.04794	34.59124	0.468044	35.05547	29.53552	5.519952	0.491405	5.028548	0.030638
24hheat_stem_ST	HSP60-09	34.63022	34.64909	35.27303	0.3658	34.85078	28.69007	6.16071	-4.49025	10.65096	0.000622
6hheat_leaf_ST	HSP60-09	28.46089	28.69093	28.8643	0.20237	28.67204	28.91894	-0.2469	0.491405	-0.73831	1.668215
6hheat_stem_ST	HSP60-09	29.23002	28.61839	28.75071	0.321801	28.86637	21.84457	7.021807	8.080248	-1.05844	2.082681
1hheat_leaf_ST	HSP60-13	28.39124	28.82539	28.62257	0.217233	28.61307	28.47389	0.139177	-0.81332	0.952499	0.516737
1hheat_stem_ST	HSP60-13	30.73536	30.45553	30.6658	0.145694	30.6189	29.7048	0.914093	-1.66392	2.578012	0.167472
24hheat_leaf_ST	HSP60-13	34.24104	33.72179	33.44857	0.40255	33.8038	29.53552	4.268282	-0.81332	5.081604	0.029531

24hheat_stem_ST	HSP60-13	33.17775	34.00139	33.80778	0.430661	33.6623	28.69007	4.972233	-1.66392	6.636152	0.010054
6hheat_leaf_ST	HSP60-13	28.20884	27.85894	27.97279	0.178468	28.01352	28.91894	-0.90542	-0.81332	-0.09209	1.065915
6hheat_stem_ST	HSP60-13	26.71159	26.95373	27.05001	0.174368	26.90511	21.84457	5.060542	2.530401	2.530141	0.173122
1hheat_leaf_ST	HSP60-14	31.88747	30.85745	32.27595	0.733029	31.67362	28.47389	3.199732	1.098698	2.101034	0.233091
1hheat_stem_ST	HSP60-14	36.89632	35.19435	Undetermined	1.203477	36.04534	29.7048	6.340535	1.994256	4.346279	0.049163
24hheat_leaf_ST	HSP60-14	35.38864	33.39282	35.19705	1.101154	34.6595	29.53552	5.123986	1.098698	4.025288	0.061414
24hheat_stem_ST	HSP60-14	Undetermined	34.87662	34.17323	0.497375	34.52492	28.69007	5.834852	1.994256	3.840596	0.069802
6hheat_leaf_ST	HSP60-14	28.89203	28.58772	28.48211	0.212837	28.65395	28.91894	-0.26499	1.098698	-1.36369	2.57342
6hheat_stem_ST	HSP60-14	30.50165	30.31783	29.93725	0.287862	30.25224	21.50089	8.75135	5.008038	3.743312	0.074671
1hheat_leaf_ST	HSP60-16	27.6488	27.56945	28.29785	0.399609	27.8387	24.80167	3.037031	-2.39107	5.428101	0.023226
1hheat_stem_ST	HSP60-16	30.40351	30.50704	30.46952	0.052416	30.46002	25.76063	4.699393	-1.64977	6.349162	0.012266
24hheat_leaf_ST	HSP60-16	34.56589	33.09659	32.19814	1.195287	33.28688	24.95838	8.328495	-2.39107	10.71957	0.000593
24hheat_stem_ST	HSP60-16	33.64226	35.37273	35.82899	1.153579	34.94799	25.67234	9.275652	-1.64977	10.92542	0.000514
6hheat_leaf_ST	HSP60-16	28.23629	27.68901	28.18109	0.301304	28.03546	25.70032	2.33514	-2.39107	4.726211	0.037781
6hheat_stem_ST	HSP60-16	32.63204	32.75892	32.79751	0.086572	32.72949	25.79675	6.932742	3.203407	3.729335	0.075398
1hheat_leaf_ST	HSP60-20	29.23002	28.61839	28.75071	0.321801	28.86637	21.84457	7.021807	8.080248	-1.05844	2.082681
1hheat_stem_ST	HSP60-20	29.73103	30.95524	31.33834	0.839535	30.67487	24.30848	6.366392	10.47297	-4.10657	17.22669
24hheat_leaf_ST	HSP60-20	26.73174	26.95258	26.74114	0.124873	26.80849	24.30848	2.500012	8.080248	-5.58024	47.843
24hheat_stem_ST	HSP60-20	31.77611	31.79769	31.62458	0.094338	31.73279	22.90618	8.826613	10.47297	-1.64635	3.130412
6hheat_leaf_ST	HSP60-20	28.03131	31.71158	29.5749	1.848083	29.77259	23.2969	6.475691	8.080248	-1.60456	3.041025
6hheat_stem_ST	HSP60-20	30.85674	30.61984	31.05035	0.21562	30.84231	21.45946	9.38285	10.47297	-1.09012	2.128911
1hheat_leaf_ST	HSP90-02	33.28541	32.81591	33.34388	0.289426	33.1484	21.84457	11.30383	11.20091	0.102928	0.931141
1hheat_stem_ST	HSP90-02	21.8062	21.73986	22.06723	0.173063	21.8711	24.30848	-2.43738	1.255558	-3.69294	12.93257
24hheat_leaf_ST	HSP90-02	28.70565	28.34646	28.73429	0.216119	28.59547	24.30848	4.286993	4.19763	0.089363	0.939938
24hheat_stem_ST	HSP90-02	32.62406	33.91491	31.38751	1.263797	32.64216	22.90618	9.73598	3.9666	5.76938	0.018333
6hheat_leaf_ST	HSP90-02	33.66085	33.94127	34.02463	0.190578	33.87558	23.2969	10.57868	2.0091	8.569576	0.002632
6hheat_stem_ST	HSP90-02	26.71159	26.95373	27.05001	0.174368	26.90511	21.84457	5.060542	2.530401	2.530141	0.173122
1hheat_leaf_ST	HSP90-03	24.46081	24.95078	24.83125	0.25547	24.74761	24.80167	-0.05405	-1.37812	1.324069	0.399407
1hheat_stem_ST	HSP90-03	30.14827	29.82715	29.40978	0.370291	29.79507	25.76063	4.034436	-0.94087	4.975311	0.031789
24hheat_leaf_ST	HSP90-03	Undetermined	34.08273	32.88809	0.844738	33.48541	24.95838	8.52703	-1.37812	9.905154	0.001043
24hheat_stem_ST	HSP90-03	32.90216	33.093	32.73766	0.177832	32.91094	25.67234	7.238599	-0.94087	8.179474	0.003449

6hheat_leaf_ST	HSP90-03	24.92578	25.73759	25.19826	0.413149	25.28721	25.70032	-0.41311	-1.37812	0.965015	0.512273
6hheat_stem_ST	HSP90-03	28.36801	28.31447	28.49207	0.091106	28.39152	29.76964	-1.37812	-1.37812	10.18657	0.000858
1hheat_leaf_ST	HSP90-04	24.9295	24.73612	24.53213	0.198706	24.73258	24.80167	-0.06909	-4.34938	4.280298	0.051464
1hheat_stem_ST	HSP90-04	26.97546	26.96951	27.61783	0.372602	27.1876	25.76063	1.426973	-0.82806	2.255035	0.209492
24hheat_leaf_ST	HSP90-04	32.82974	33.9918	32.0201	0.991084	32.94721	24.95838	7.988833	-4.34938	12.33822	0.000193
24hheat_stem_ST	HSP90-04	33.60768	31.46277	32.78318	1.081965	32.61788	25.67234	6.945539	-0.82806	7.773601	0.00457
6hheat_leaf_ST	HSP90-04	26.14578	25.4596	25.53615	0.376018	25.71385	25.70032	0.013526	-4.34938	4.362911	0.0486
6hheat_stem_ST	HSP90-04	32.63204	32.75892	32.79751	0.086572	32.72949	25.79675	6.932742	3.203407	3.729335	0.075398
1hheat_leaf_ST	HSP90-05	27.14665	27.70773	27.30558	0.289194	27.38665	24.80167	2.584985	-2.88662	5.471601	0.022536
1hheat_stem_ST	HSP90-05	31.65812	30.72762	31.5654	0.51256	31.31705	25.76063	5.556418	-0.85395	6.41037	0.011757
24hheat_leaf_ST	HSP90-05	33.07193	32.61632	33.29932	0.347797	32.99585	24.95838	8.037475	-2.88662	10.92409	0.000515
24hheat_stem_ST	HSP90-05	32.87948	33.20145	34.05013	0.604748	33.37702	25.67234	7.704679	-0.85395	8.558632	0.002652
6hheat_leaf_ST	HSP90-05	25.53903	26.01116	25.61843	0.2528	25.72287	25.70032	0.022553	-2.88662	2.909169	0.133123
6hheat_stem_ST	HSP90-05	27.04497	26.71314	26.89096	0.166057	26.88302	29.76964	-2.88662	-2.88662	0.569422	0.673887
1hheat_leaf_ST	HSP90-09	25.25638	24.67578	25.10325	0.30091	25.0118	24.80167	0.210134	-0.42544	0.635578	0.643683
1hheat_stem_ST	HSP90-09	24.91166	24.49517	24.61474	0.214446	24.67385	25.76063	-1.08678	4.974742	-6.06152	66.78803
24hheat_leaf_ST	HSP90-09	29.22626	29.40761	29.60961	0.191768	29.41449	24.95838	4.456112	-0.42544	4.881556	0.033924
24hheat_stem_ST	HSP90-09	29.74529	29.95771	28.93299	0.540834	29.54533	25.67234	3.872992	4.974742	-1.10175	2.146148
6hheat_leaf_ST	HSP90-09	22.32156	21.7606	22.40939	0.351978	22.16385	25.70032	-3.53647	-0.42544	-3.11103	8.639962
6hheat_stem_ST	HSP90-09	30.44081	31.51466	26.07712	2.879867	29.3442	29.76964	-0.42544	-0.42544	-1.60456	3.041025
1hheat_leaf_ST	sHSP-02	32.34584	33.07082	32.62709	0.365511	32.68125	28.47389	4.207356	1.994441	2.212915	0.215698
1hheat_stem_ST	sHSP-02	35.59685	34.98147	34.33288	0.632058	34.9704	29.7048	5.265598	2.032746	3.232853	0.106369
24hheat_leaf_ST	sHSP-02	32.39346	32.01263	32.02478	0.216452	32.14362	29.53552	2.608103	1.994441	0.613661	0.653536
24hheat_stem_ST	sHSP-02	32.41048	32.29317	32.37098	0.05969	32.35821	28.69007	3.668139	2.032746	1.635393	0.321883
6hheat_leaf_ST	sHSP-02	34.01301	32.81509	33.50214	0.601114	33.44342	28.91894	4.524477	1.994441	2.530036	0.173134
6hheat_stem_ST	sHSP-02	26.30015	26.07559	25.90697	0.197251	26.09424	22.32045	3.773793	-6.41278	10.18657	0.000858
1hheat_leaf_ST	sHSP-07	26.18481	26.08663	25.96276	0.111274	26.07807	22.45687	3.621203	5.156522	-1.53532	2.898525
1hheat_stem_ST	sHSP-07	25.74733	25.97733	25.93443	0.1223	25.88636	22.41561	3.470751	-6.41278	9.883529	0.001059
24hheat_leaf_ST	sHSP-07	26.31973	Undetermined	26.25373	0.04667	26.28673	25.3931	0.893628	5.156522	-4.26289	19.19813
24hheat_stem_ST	sHSP-07	26.49066	26.90824	27.31562	0.41249	26.90484	25.42716	1.477676	0.939984	0.537692	0.688872
6hheat_leaf_ST	sHSP-07	33.05307	34.4377	32.96672	0.738327	33.48583	30.38865	3.097181	3.274928	-0.17775	1.131116

6hheat_stem_ST	sHSP-07	34.52968	31.08104	32.40417	1.556132	32.67163	30.98837	1.683261	2.00304	-0.31978	1.24814
1hheat_leaf_ST	sHSP-08	33.17757	Undetermined	Undetermined	0	33.17757	32.58468	0.592887	2.979305	-2.38642	5.228574
1hheat_stem_ST	sHSP-08	25.87383	25.72547	26.19626	0.215289	25.93186	27.11683	-1.18498	2.00304	-3.18802	9.113566
24hheat_leaf_ST	sHSP-08	34.95469	34.9197	35.90403	0.499516	35.25947	31.86798	3.391495	2.979305	0.41219	0.751482
24hheat_stem_ST	sHSP-08	27.7945	27.72374	28.42882	0.347276	27.98235	28.67466	-0.69231	2.00304	-2.69535	6.477103
6hheat_leaf_ST	sHSP-08	32.43135	32.31139	33.42099	0.544669	32.72124	30.38865	2.332595	2.979305	-0.64671	1.565594
6hheat_stem_ST	sHSP-08	32.63204	32.75892	32.79751	0.086572	32.72949	25.79675	6.932742	3.203407	3.729335	0.075398
1hheat_leaf_ST	sHSP-11	30.87317	30.86753	30.69231	0.102831	30.81101	28.47389	2.337116	0.844496	1.49262	0.355367
1hheat_stem_ST	sHSP-11	31.54091	31.05649	31.08327	0.272277	31.22689	29.7048	1.522085	2.81045	-1.28837	2.442511
24hheat_leaf_ST	sHSP-11	31.59137	31.34055	32.93908	0.8597	31.957	29.53552	2.42148	0.844496	1.576985	0.335182
24hheat_stem_ST	sHSP-11	32.94942	31.74637	32.02668	0.629464	32.24082	28.69007	3.55075	2.81045	0.7403	0.598615
6hheat_leaf_ST	sHSP-11	31.45853	31.20621	31.34532	0.126384	31.33668	28.91894	2.417745	0.844496	1.573249	0.336051
6hheat_stem_ST	sHSP-11	26.71159	26.95373	27.05001	0.174368	26.90511	21.84457	5.060542	2.530401	2.530141	0.173122
1hheat_leaf_ST	sHSP-15	21.35093	21.44282	21.58208	0.116382	21.45861	28.47389	-7.01528	0.777854	-7.79314	221.8031
1hheat_stem_ST	sHSP-15	23.66929	23.58276	23.62448	0.043275	23.62551	29.7048	-6.07929	1.623717	-7.70301	208.3708
24hheat_leaf_ST	sHSP-15	30.21972	30.23488	30.49759	0.156235	30.3174	29.53552	0.78188	0.777854	0.004026	0.997213
24hheat_stem_ST	sHSP-15	31.53533	31.32369	31.26651	0.141609	31.37518	28.69007	2.685106	1.623717	1.061389	0.47917
6hheat_leaf_ST	sHSP-15	26.43199	26.50776	26.68833	0.131689	26.54269	28.91894	-2.37625	0.777854	-3.1541	8.901822
6hheat_stem_ST	sHSP-15	32.63204	32.75892	32.79751	0.086572	32.72949	25.79675	6.932742	3.203407	3.729335	0.075398
1hheat_leaf_ST	sHSP-25	23.16308	23.02493	22.91291	0.125312	23.03364	28.47389	-5.44025	0.658062	-6.09831	68.5132
1hheat_stem_ST	sHSP-25	26.29036	24.9731	25.3395	0.679898	25.53432	25.42716	0.107158	-6.41278	6.519936	0.010897
24hheat_leaf_ST	sHSP-25	33.3182	33.36997	32.10536	0.715648	32.93118	29.53552	3.39566	0.658062	2.737598	0.149934
24hheat_stem_ST	sHSP-25	30.2763	30.02935	30.98555	0.496377	30.4304	28.69007	1.740328	3.365561	-1.62523	3.08492
6hheat_leaf_ST	sHSP-25	24.70487	24.71384	24.78697	0.045038	24.73523	28.91894	-4.18371	0.658062	-4.84177	28.67605
6hheat_stem_ST	sHSP-25	26.29036	24.9731	25.3395	0.679898	25.53432	25.42716	0.107158	-6.41278	6.519936	0.010897
1hheat_leaf_ST	sHSP-26	24.80072	25.01397	24.84492	0.112552	24.88654	28.47389	-3.58736	0.788017	-4.37537	20.75479
1hheat_stem_ST	sHSP-26	25.59783	25.9514	25.69899	0.182098	24.87519	0.823801	3.274928	-2.45113	5.46843	0.022586
24hheat_leaf_ST	sHSP-26	32.84811	32.51626	33.00184	0.248174	32.78874	29.53552	3.25322	0.788017	2.465203	0.181092
24hheat_stem_ST	sHSP-26	31.91303	32.62022	31.58115	0.530711	32.03813	28.69007	3.348063	3.274928	0.073135	0.95057
6hheat_leaf_ST	sHSP-26	26.91409	27.00909	26.99966	0.052339	26.97428	28.91894	-1.94466	0.788017	-2.73268	6.646876
6hheat_stem_ST	sHSP-26	26.29036	24.9731	25.3395	0.679898	25.53432	25.42716	0.107158	-6.41278	6.519936	0.010897

1hheat_leaf_ST	sHSP-27	21.21903	20.60145	21.39758	0.417756	21.07269	24.05428	-2.98159	2.00304	-4.98463	31.66099
1hheat_stem_ST	sHSP-27	22.68214	22.62301	22.88767	0.138917	22.73094	24.87519	-2.14425	2.979305	-5.12355	34.86124
24hheat_leaf_ST	sHSP-27	29.242	29.29919	29.63108	0.210082	29.39075	29.53552	-0.14477	2.00304	-2.14781	4.431531
24hheat_stem_ST	sHSP-27	28.26917	28.19065	28.32106	0.065657	28.26029	28.69007	-0.42978	2.979305	-3.40908	10.62274
6hheat_leaf_ST	sHSP-27	23.8564	23.8838	23.95994	0.053649	23.90005	23.71388	0.186164	2.00304	-1.81688	3.523175
6hheat_stem_ST	sHSP-27	26.71159	26.95373	27.05001	0.174368	26.90511	21.84457	5.060542	2.530401	2.530141	0.173122
1hheat_leaf_ST	sHSP-30	25.81419	25.66905	25.67532	0.082049	25.71952	22.16385	3.555671	-2.74485	6.300523	0.012687
1hheat_stem_ST	sHSP-30	33.19505	34.90294	33.55143	0.900972	33.88314	25.6533	8.229834	7.276778	0.953056	0.516537
24hheat_leaf_ST	sHSP-30	24.34508	24.47895	24.85045	0.261834	24.55816	22.35948	2.19868	7.780998	-5.58232	47.91207
24hheat_stem_ST	sHSP-30	23.85489	24.65246	24.65093	0.460037	24.38609	25.68489	-1.2988	2.8156	-4.1144	17.32035
6hheat_leaf_ST	sHSP-30	35.12326	Undetermined	35.22429	0.058327	35.17377	32.58468	2.589093	3.365561	-0.77647	1.712932
6hheat_stem_ST	sHSP-30	26.44326	26.51584	26.40086	0.052008	26.45332	27.11683	-0.66351	0.658062	-1.32157	2.499384
**Susceptible cultivar in response to heat stress**											
**SAMPLE**	**DETECTOR**	**Ct1**	**Ct2**	**Ct3**	**sd**	**Avg Ct**	**End CTl**	**dCt**	**CTL0h**	**ddCt**	**Log Ct**
1hheat_leaf_SS	HSP100-07	25.96697	26.98838	25.75225	0.660478	26.23587	24.12828	2.10759	-2.00306	4.110645	0.057886
1hheat_stem_SS	HSP100-07	25.92741	26.9796	27.07663	0.637344	26.66121	25.93535	0.725858	0.585873	0.139984	0.907529
24hheat_leaf_SS	HSP100-07	35.45438	35.13613	34.40099	0.540269	34.99716	25.10597	9.891198	-2.00306	11.89425	0.000263
24hheat_stem_SS	HSP100-07	28.88817	29.56423	29.74039	0.449885	29.3976	29.9759	-0.5783	0.585873	-1.16418	2.241052
6hheat_leaf_SS	HSP100-07	27.22759	27.88017	28.3465	0.562033	27.81809	24.11908	3.699004	-2.00306	5.702059	0.019209
6hheat_stem_SS	HSP100-07	25.65985	24.96659	24.71395	0.489756	25.11346	24.97927	0.134197	0.585873	-0.45168	1.367629
1hheat_leaf_SS	HSP100-08	21.3247	21.74624	21.43174	0.219112	21.50089	24.12828	-2.62739	-3.7291	1.101711	0.465964
1hheat_stem_SS	HSP100-08	24.48857	23.81845	24.29907	0.345436	24.20203	25.93535	-1.73333	0.74127	-2.4746	5.558114
24hheat_leaf_SS	HSP100-08	34.27634	33.99267	32.41239	1.004328	33.56047	25.10597	8.4545	-3.7291	12.1836	0.000215
24hheat_stem_SS	HSP100-08	26.54079	26.66036	27.2404	0.374211	26.81385	29.9759	-3.16205	0.74127	-3.90332	14.9629
6hheat_leaf_SS	HSP100-08	25.18256	25.45622	25.64271	0.231448	25.42716	24.11908	1.308078	-3.7291	5.037174	0.030455
6hheat_stem_SS	HSP100-08	22.06608	22.41697	22.47828	0.222406	22.32045	24.97927	-2.65882	0.74127	-3.40009	10.55673
1hheat_leaf_SS	HSP100-11	31.9977	30.92293	30.23383	0.888934	31.05148	24.12828	6.923206	1.820752	5.102454	0.029108
1hheat_stem_SS	HSP100-11	32.15781	32.09598	31.93593	0.114508	32.06324	25.93535	6.127883	3.945703	2.182181	0.220342
24hheat_leaf_SS	HSP100-11	33.41622	31.85324	33.19852	0.846571	32.82266	25.10597	7.716694	1.820752	5.895943	0.016794
24hheat_stem_SS	HSP100-11	33.31073	32.76572	34.33891	0.798864	33.47179	29.9759	3.495888	3.945703	-0.44982	1.365865

6hheat_leaf_SS	HSP100-11	32.46313	33.36459	33.3636	0.520172	33.06377	24.11908	8.94469	1.820752	7.123938	0.007169
6hheat_stem_SS	HSP100-11	31.87148	31.70417	32.25341	0.28152	31.94302	24.97927	6.963753	3.945703	3.01805	0.123446
1hheat_leaf_SS	HSP100-12	34.44129	32.11224	32.2856	1.297531	32.94638	24.12828	8.818097	4.105266	4.712831	0.038133
1hheat_stem_SS	HSP100-12	31.15671	30.7942	29.16653	1.059994	30.37248	25.93535	4.437128	3.945703	0.491425	0.711322
24hheat_leaf_SS	HSP100-12	32.69386	35.34673	34.83886	1.40811	34.29315	25.10597	9.187186	4.105266	5.08192	0.029525
24hheat_stem_SS	HSP100-12	36.22703	35.44515	34.5129	0.858164	35.39503	29.9759	5.419127	3.945703	1.473425	0.360126
6hheat_leaf_SS	HSP100-12	32.47515	32.08386	32.57673	0.26024	32.37858	24.11908	8.259497	4.105266	4.154231	0.056163
6hheat_stem_SS	HSP100-12	28.03961	27.79799	28.3514	0.277443	28.063	24.97927	3.083733	3.945703	-0.86197	1.817518
1hheat_leaf_SS	HSP60-02	24.70468	25.5364	26.07283	0.689367	25.43797	25.09223	0.345742	7.780998	-7.43526	1.075215
1hheat_stem_SS	HSP60-02	26.89708	26.73473	26.9209	0.101309	26.8509	22.00433	4.846571	4.7897	0.056871	0.961347
24hheat_leaf_SS	HSP60-02	28.51739	28.80398	28.8155	0.168884	28.71229	24.23562	4.476673	2.524207	1.952465	0.258374
24hheat_stem_SS	HSP60-02	26.50151	26.98842	27.14805	0.336792	26.87932	23.29518	3.58414	6.190665	-2.60653	6.090351
6hheat_leaf_SS	HSP60-02	26.71159	26.95373	27.05001	0.174368	26.90511	22.35948	4.545631	2.524207	2.021424	0.246315
6hheat_stem_SS	HSP60-02	30.94935	30.98024	30.80831	0.091655	30.91263	25.68489	5.227748	6.190665	-0.96292	1.949247
1hheat_leaf_SS	HSP60-03	28.83676	29.08653	28.85088	0.140307	28.92472	25.09223	3.832492	6.190665	-2.35817	5.127209
1hheat_stem_SS	HSP60-03	26.00511	26.47907	26.59614	0.31296	26.36011	22.32045	4.039665	0.37819	3.661475	0.079029
24hheat_leaf_SS	HSP60-03	26.92863	26.92576	26.80374	0.071293	26.88605	22.45687	4.429181	5.209399	-0.78022	1.71739
24hheat_stem_SS	HSP60-03	26.86088	26.89069	26.0047	0.503144	26.58543	22.41561	4.169812	0.37819	3.791622	0.072212
6hheat_leaf_SS	HSP60-03	26.34935	26.00218	28.30709	1.242704	26.88621	25.42716	1.459047	-3.50825	4.967298	0.031966
6hheat_stem_SS	HSP60-03	26.20616	26.34653	26.12397	0.112543	26.22555	22.32045	3.905109	2.393056	1.512053	0.350612
1hheat_leaf_SS	HSP60-05	25.97441	26.43982	25.90976	0.289179	26.10799	24.12828	1.979715	-0.58811	2.567821	0.168659
1hheat_stem_SS	HSP60-05	25.16883	24.8282	24.36292	0.404557	24.78665	25.93535	-1.14871	-1.38429	0.235588	0.849339
24hheat_leaf_SS	HSP60-05	33.61033	33.39347	32.41621	0.63613	33.14	25.10597	8.034039	-0.58811	8.622145	0.002538
24hheat_stem_SS	HSP60-05	28.53537	28.48901	28.82282	0.180836	28.61574	29.9759	-1.36016	-1.38429	0.024132	0.983412
6hheat_leaf_SS	HSP60-05	28.72062	28.52296	29.28976	0.398114	28.84445	24.11908	4.725366	-0.58811	5.313472	0.025147
6hheat_stem_SS	HSP60-05	24.03825	23.7519	23.72317	0.174209	23.83777	24.97927	-1.14149	-1.38429	0.2428	0.845104
1hheat_leaf_SS	HSP60-09	26.08532	26.45027	26.5168	0.232303	26.3508	24.12828	2.222517	-0.96885	3.191365	0.109472
1hheat_stem_SS	HSP60-09	25.89848	26.21016	25.62507	0.292756	25.91124	25.93535	-0.02412	0.200704	-0.22482	1.168633
24hheat_leaf_SS	HSP60-09	32.41091	33.14599	33.48542	0.549261	33.0141	25.10597	7.908139	-0.96885	8.876987	0.002127
24hheat_stem_SS	HSP60-09	28.49288	28.32594	28.47364	0.091336	28.43082	29.9759	-1.54508	0.200704	-1.74578	3.353762
6hheat_leaf_SS	HSP60-09	28.76764	28.84623	28.95328	0.093183	28.85572	24.11908	4.736634	-0.96885	5.705482	0.019164

6hheat_stem_SS	HSP60-09	25.01277	25.13462	25.14888	0.074811	25.09876	24.97927	0.119491	0.200704	-0.08121	1.057907
1hheat_leaf_SS	HSP60-13	25.15061	24.85999	24.77397	0.197364	24.92819	24.12828	0.799912	-1.88448	2.684395	0.155567
1hheat_stem_SS	HSP60-13	24.82015	24.14651	24.33324	0.347793	24.4333	25.93535	-1.50206	-2.08829	0.586236	0.666078
24hheat_leaf_SS	HSP60-13	32.31046	32.21015	33.14884	0.515442	32.55648	25.10597	7.450518	-1.88448	9.335001	0.001548
24hheat_stem_SS	HSP60-13	28.05185	28.15435	28.42309	0.191722	28.20976	29.9759	-1.76613	-2.08829	0.322158	0.799873
6hheat_leaf_SS	HSP60-13	27.41808	27.72603	28.13516	0.359732	27.75976	24.11908	3.640674	-1.88448	5.525157	0.021715
6hheat_stem_SS	HSP60-13	22.78882	23.12671	23.61628	0.416038	23.17727	24.97927	-1.802	-2.08829	0.286296	0.820005
1hheat_leaf_SS	HSP60-14	25.07403	26.18554	26.01084	0.597718	25.7568	24.12828	1.628525	-1.15998	2.788503	0.144736
1hheat_stem_SS	HSP60-14	31.11654	29.27041	29.77112	0.954731	30.05269	25.93535	4.117339	5.637083	-1.51974	2.867402
24hheat_leaf_SS	HSP60-14	32.40037	32.35703	32.52288	0.086016	32.42676	25.10597	7.320796	-1.15998	8.480774	0.002799
24hheat_stem_SS	HSP60-14	31.78289	33.19474	32.834	0.733517	32.60388	29.9759	2.627982	5.637083	-3.0091	8.050628
6hheat_leaf_SS	HSP60-14	29.86219	31.13912	31.23752	0.767221	30.74627	24.11908	6.62719	-1.15998	7.787168	0.004527
6hheat_stem_SS	HSP60-14	28.4933	28.28228	29.04962	0.396406	28.6084	24.97927	3.629131	5.637083	-2.00795	4.022108
1hheat_leaf_SS	HSP60-16	25.84692	26.04989	25.27851	0.399854	25.72511	24.12828	1.596828	-0.3572	1.95403	0.258094
1hheat_stem_SS	HSP60-16	25.28692	25.6376	24.38043	0.648736	25.10165	25.93535	-0.8337	-0.91104	0.077339	0.947804
24hheat_leaf_SS	HSP60-16	33.59608	33.45806	34.30518	0.454515	33.78644	25.10597	8.680474	-0.3572	9.037676	0.001903
24hheat_stem_SS	HSP60-16	28.12162	28.40031	28.64201	0.260414	28.38798	29.9759	-1.58792	-0.91104	-0.67688	1.598676
6hheat_leaf_SS	HSP60-16	27.48306	28.21205	28.87344	0.695466	28.18952	24.11908	4.070434	-0.3572	4.427637	0.046467
6hheat_stem_SS	HSP60-16	23.66118	23.89551	24.39874	0.376862	23.98514	24.97927	-0.99412	-0.91104	-0.08308	1.059278
1hheat_leaf_SS	HSP90-02	28.70565	28.34646	28.73429	0.216119	28.59547	22.16385	2.318012	1.6667	0.651312	0.636701
1hheat_stem_SS	HSP90-02	32.62406	33.91491	31.38751	1.263797	32.64216	23.71978	8.922384	-0.52198	9.444368	0.001435
24hheat_leaf_SS	HSP90-02	33.66085	33.94127	34.02463	0.190578	33.87558	25.72899	8.146589	3.203407	4.943182	0.032505
24hheat_stem_SS	HSP90-02	33.39828	33.531	33.84618	0.230063	33.59182	23.90293	9.688897	-0.52198	10.21088	0.000844
6hheat_leaf_SS	HSP90-02	25.66739	25.617	25.69202	0.038241	25.6588	22.41561	3.243191	1.098698	2.144493	0.226174
6hheat_stem_SS	HSP90-02	27.87881	27.63464	27.93376	0.159224	27.81573	25.3931	2.422632	1.220803	1.20183	0.434724
1hheat_leaf_SS	HSP90-03	27.74746	27.84983	27.34196	0.268587	27.64641	24.12828	3.518134	1.134704	2.383431	0.191653
1hheat_stem_SS	HSP90-03	25.10076	26.49256	25.77788	0.695985	25.7904	25.93535	-0.14496	2.289208	-2.43416	5.404511
24hheat_leaf_SS	HSP90-03	32.83027	33.01808	33.92234	0.583889	33.2569	25.10597	8.150931	1.134704	7.016227	0.007725
24hheat_stem_SS	HSP90-03	28.91739	29.00308	29.65062	0.400892	29.19036	29.9759	-0.78553	2.289208	-3.07474	8.425379
6hheat_leaf_SS	HSP90-03	30.67094	30.77326	31.22075	0.292407	30.88832	24.11908	6.769235	1.134704	5.634531	0.02013
6hheat_stem_SS	HSP90-03	25.86894	26.56777	26.7788	0.476224	26.40517	24.97927	1.425905	2.289208	-0.8633	1.819199

1hheat_leaf_SS	HSP90-04	25.70171	25.96931	25.635	0.176929	25.76867	24.12828	1.640394	-1.89755	3.537941	0.086094
1hheat_stem_SS	HSP90-04	26.82335	27.33385	28.4141	0.812205	27.52377	25.93535	1.588412	0.820686	0.767726	0.587342
24hheat_leaf_SS	HSP90-04	32.87455	33.51234	32.39898	0.558646	32.92862	25.10597	7.822659	-1.89755	9.720207	0.001186
24hheat_stem_SS	HSP90-04	30.92519	32.27888	31.34027	0.69351	31.51478	29.9759	1.538882	0.820686	0.718196	0.607857
6hheat_leaf_SS	HSP90-04	30.06373	30.92839	31.31714	0.641587	30.76975	24.11908	6.650668	-1.89755	8.548216	0.002671
6hheat_stem_SS	HSP90-04	27.8929	28.15237	28.13705	0.145585	28.06077	24.97927	3.081505	0.820686	2.260819	0.208653
1hheat_leaf_SS	HSP90-05	26.5793	26.78285	26.92069	0.171749	26.76095	24.12828	2.632669	-1.72546	4.358128	0.048761
1hheat_stem_SS	HSP90-05	29.25862	28.50294	28.43503	0.457158	28.7322	25.93535	2.796843	1.02315	1.773692	0.292459
24hheat_leaf_SS	HSP90-05	33.90617	34.85355	34.68067	0.504526	34.48013	25.10597	9.374163	-1.72546	11.09962	0.000456
24hheat_stem_SS	HSP90-05	30.85424	30.88251	30.93016	0.038371	30.88897	29.9759	0.913075	1.02315	-0.11008	1.079285
6hheat_leaf_SS	HSP90-05	29.20722	30.97675	31.20275	1.092734	30.46224	24.11908	6.343158	-1.72546	8.068616	0.003725
6hheat_stem_SS	HSP90-05	27.37338	27.57637	27.68936	0.160113	27.54637	24.97927	2.567104	1.02315	1.543954	0.342944
1hheat_leaf_SS	HSP90-09	27.30615	26.45748	27.55037	0.57363	27.10467	24.12828	2.976388	2.23919	0.737198	0.599903
1hheat_stem_SS	HSP90-09	31.44063	30.42307	Undetermined	0.71952	30.93185	25.93535	4.996496	6.543193	-1.5467	2.921475
24hheat_leaf_SS	HSP90-09	30.96681	30.24748	29.73193	0.62024	30.31541	25.10597	5.20944	2.23919	2.97025	0.127604
24hheat_stem_SS	HSP90-09	Undetermined	37.14817	32.58602	3.225926	34.86709	29.9759	4.891195	6.543193	-1.652	3.142686
6hheat_leaf_SS	HSP90-09	32.1511	30.2741	32.6914	1.268754	31.70553	24.11908	7.586449	2.23919	5.347259	0.024565
6hheat_stem_SS	HSP90-09	28.86041	28.52743	29.76272	0.639138	29.05018	24.97927	4.070917	6.543193	-2.47228	5.549184
1hheat_leaf_SS	sHSP-02	29.8001	30.22941	29.27845	0.476226	29.76932	24.12828	5.641041	2.631307	3.009734	0.124159
1hheat_stem_SS	sHSP-02	31.8446	31.19189	31.33409	0.343236	31.45686	25.93535	5.521508	6.923013	-1.40151	2.64177
24hheat_leaf_SS	sHSP-02	32.34459	31.50163	32.53014	0.548156	32.12545	25.10597	7.019489	2.631307	4.388182	0.047756
24hheat_stem_SS	sHSP-02	33.01296	#VALUE!	36.42967	#VALUE!	34.72131	29.9759	4.745416	6.923013	-2.1776	4.523993
6hheat_leaf_SS	sHSP-02	30.43379	30.58361	30.62356	0.100045	30.54699	24.11908	6.427904	2.631307	3.796597	0.071963
6hheat_stem_SS	sHSP-02	29.55477	31.19079	29.53524	0.950248	30.0936	24.97927	5.114332	6.923013	-1.80868	3.503219
1hheat_leaf_SS	sHSP-07	34.95469	34.9197	35.90403	0.499516	35.25947	31.86798	3.391495	2.979305	0.41219	0.751482
1hheat_stem_SS	sHSP-07	27.7945	27.72374	28.42882	0.347276	27.98235	28.67466	-0.69231	2.00304	-2.69535	6.477103
24hheat_leaf_SS	sHSP-07	32.43135	32.31139	33.42099	0.544669	32.72124	30.38865	2.332595	2.979305	-0.64671	1.565594
24hheat_stem_SS	sHSP-07	31.6191	32.21506	33.17901	0.704056	32.33772	31.86798	0.469745	3.274928	-2.80518	6.989468
6hheat_leaf_SS	sHSP-07	29.35702	30.4772	30.10041	0.509823	29.97821	28.67466	1.303546	0.788017	0.515529	0.699536
6hheat_stem_SS	sHSP-07	31.97957	32.11514	32.6424	0.313203	32.2457	30.38865	1.857049	3.274928	-1.41788	2.671923
1hheat_leaf_SS	sHSP-08	34.18128	34.34284	34.57661	0.177777	34.36691	30.98837	3.37854	0.788017	2.590523	0.166026

1hheat_stem_SS	sHSP-08	31.63097	32.11416	31.79771	0.219519	31.84761	32.58468	-0.73707	-0.66351	-0.07356	1.052307
24hheat_leaf_SS	sHSP-08	32.67087	33.16291	32.64088	0.262176	32.82489	28.67466	4.150224	2.31406	1.836165	0.280065
24hheat_stem_SS	sHSP-08	25.81419	25.66905	25.67532	0.082049	25.71952	22.16385	3.555671	-2.74485	6.300523	0.012687
6hheat_leaf_SS	sHSP-08	33.19505	34.90294	33.55143	0.900972	33.88314	25.6533	8.229834	7.276778	0.953056	0.516537
6hheat_stem_SS	sHSP-08	27.93754	28.39068	28.5812	0.330636	28.30314	23.71978	4.583364	2.00304	2.580324	0.167203
1hheat_leaf_SS	sHSP-11	26.03889	26.62094	26.44941	0.299092	26.36975	24.12828	2.241469	0.714292	1.527177	0.346956
1hheat_stem_SS	sHSP-11	24.34914	22.91846	22.92559	0.823953	23.39773	25.93535	-2.53762	0.249301	-2.78693	6.901577
24hheat_leaf_SS	sHSP-11	32.86461	32.47826	33.01849	0.27833	32.78712	25.10597	7.681155	0.714292	6.966863	0.007994
24hheat_stem_SS	sHSP-11	26.15119	27.05481	27.42851	0.656724	26.87817	29.9759	-3.09773	0.249301	-3.34703	10.17552
6hheat_leaf_SS	sHSP-11	26.24301	27.39009	27.70928	0.771109	27.11413	24.11908	2.995043	0.714292	2.280751	0.205791
6hheat_stem_SS	sHSP-11	22.12395	22.98197	22.26468	0.460164	22.45687	24.97927	-2.5224	0.249301	-2.7717	6.829133
1hheat_leaf_SS	sHSP-15	27.43306	28.7705	27.48696	0.757092	27.89684	24.12828	3.768558	1.426268	2.34229	0.197197
1hheat_stem_SS	sHSP-15	29.61857	27.62618	29.49533	1.116436	28.91336	25.93535	2.978006	0.859893	2.118113	0.230348
24hheat_leaf_SS	sHSP-15	32.17983	32.67381	32.40403	0.247339	32.41922	25.10597	7.313257	1.426268	5.886989	0.016898
24hheat_stem_SS	sHSP-15	33.24169	32.21975	32.27196	0.575537	32.5778	29.9759	2.601903	0.859893	1.74201	0.298953
6hheat_leaf_SS	sHSP-15	29.61114	29.40585	31.07355	0.909399	30.03018	24.11908	5.911097	1.426268	4.484829	0.044661
6hheat_stem_SS	sHSP-15	28.17186	28.53721	28.32546	0.183444	28.34484	24.97927	3.365575	0.859893	2.505682	0.176082
1hheat_leaf_SS	sHSP-25	28.66511	29.5991	30.82402	1.082717	29.69608	24.12828	5.5678	2.602725	2.965075	0.128063
1hheat_stem_SS	sHSP-25	30.27051	29.38398	29.48323	0.48573	29.71257	25.93535	3.77722	5.437723	-1.6605	3.161267
24hheat_leaf_SS	sHSP-25	32.65819	32.41228	32.08281	0.288698	32.38443	25.10597	7.278461	2.602725	4.675736	0.039126
24hheat_stem_SS	sHSP-25	31.2482	32.05554	30.87726	0.602459	31.39367	29.9759	1.417769	5.437723	-4.01995	16.22283
6hheat_leaf_SS	sHSP-25	31.6191	32.21506	33.17901	0.787158	32.33772	24.11908	8.218638	2.602725	5.615913	0.020391
6hheat_stem_SS	sHSP-25	29.35702	30.4772	30.10041	0.569999	29.97821	24.97927	4.998941	5.437723	-0.43878	1.35546
1hheat_leaf_SS	sHSP-26	31.63097	32.11416	31.79771	0.24543	31.84761	24.12828	7.719334	3.953186	3.766148	0.073498
1hheat_stem_SS	sHSP-26	32.67087	33.16291	32.64088	0.293122	32.82489	25.93535	6.889531	5.258543	1.630988	0.322867
24hheat_leaf_SS	sHSP-26	31.79455	31.55073	32.92556	0.733573	32.09028	25.10597	6.984313	3.953186	3.031126	0.122332
24hheat_stem_SS	sHSP-26	#VALUE!	35.41071	35.42814	#VALUE!	35.41942	29.9759	5.443525	5.258543	0.184983	0.87966
6hheat_leaf_SS	sHSP-26	33.39578	35.22473	33.52942	1.019558	34.04998	24.11908	9.930895	3.953186	5.977708	0.015868
6hheat_stem_SS	sHSP-26	32.15436	34.38535	32.81613	1.145843	33.11861	24.97927	8.139347	5.258543	2.880805	0.135766
1hheat_leaf_SS	sHSP-27	28.36953	29.3157	29.80355	0.729117	29.16293	24.12828	5.034647	0.60927	4.425377	0.04654
1hheat_stem_SS	sHSP-27	27.70894	27.78297	28.38883	0.373005	27.96025	25.93535	2.024893	1.253735	0.771158	0.585947

24hheat_leaf_SS	sHSP-27	33.93436	33.28621	33.42141	0.341932	33.54733	25.10597	8.44136	0.60927	7.832089	0.004388
24hheat_stem_SS	sHSP-27	30.5948	30.09433	31.12007	0.512923	30.60307	29.9759	0.627168	1.253735	-0.62657	1.543887
6hheat_leaf_SS	sHSP-27	30.46845	30.55067	30.77137	0.156645	30.59683	24.11908	6.477746	0.60927	5.868475	0.017116
6hheat_stem_SS	sHSP-27	25.85951	26.10044	26.21724	0.18242	26.05906	24.97927	1.079797	1.253735	-0.17394	1.128134
1hheat_leaf_SS	sHSP-30	26.44326	26.51584	26.40086	0.052008	26.45332	27.11683	-0.66351	0.658062	-1.32157	2.499384
1hheat_stem_SS	sHSP-30	Undetermined	35.31896	33.04511	1.312808	34.18204	31.86798	2.31406	3.365561	-1.0515	2.072686
24hheat_leaf_SS	sHSP-30	28.24348	28.31382	28.23427	0.038921	28.26386	28.67466	-0.4108	0.658062	-1.06887	2.097783
24hheat_stem_SS	sHSP-30	32.63204	32.75892	32.79751	0.077433	32.72949	30.38865	2.340841	3.365561	-1.02472	2.034564
6hheat_leaf_SS	sHSP-30	32.41454	31.7491	31.58443	0.393107	31.91603	30.98837	0.927655	0.788017	0.139638	0.907747
6hheat_stem_SS	sHSP-30	26.31472	27.16257	26.77635	0.379669	26.75122	27.11683	-0.36561	0.788017	-1.15363	2.224731

Supplementary Table 7. Distribution of methylation percentase for TSS and gene region of *SiHSP* genes

	ID	Chr.	Start	End	Strand	IC04			IC41		
						CpG	CHG	CHH	CpG	CHG	CHH
Gene Body	SiHSP60-01	1	179729	184974	+	74	4	6	62	4	6
	SisHSP-01	1	3123887	3125282	-	508	92	171	830	240	413
	SisHSP-02	1	4474502	4475416	+	139	46	117	359	135	224
	SiHSP60-02	1	7299768	7304886	-	89	10	7	33	3	6
	SisHSP-03	1	8792291	8793129	-	80	37	20	128	45	51
	SiHSP70-01	1	10246401	10250296	-	126	3	19	107	6	3
	SiHSP70-02	1	36152254	36158358	+	305	57	124	501	131	273
	SisHSP-04	1	36169044	36170129	+	53	28	14	86	29	61
	SisHSP-05	1	38572788	38573986	+	4	0	7	4	0	2
	SisHSP-06	1	39834413	39835503	+	84	37	36	196	25	78
	SiHSP70-03	2	227828	229641	-	66	20	43	129	38	52
	SiHSP60-03	2	589555	594286	-	75	2	12	50	0	2
	SiHSP60-04	2	14956816	14960659	+	247	2	10	47	0	2
	SiHSP70-04	2	32025020	32027826	+	106	3	6	104	3	7
	SiHSP70-05	2	35804609	35808035	-	114	3	28	63	3	8
	SisHSP-07	2	41831224	41832119	+	0	1	0	0	0	0
	SiHSP70-06	3	650939	652060	-	191	77	214	405	176	305
	SisHSP-08	3	2125364	2127024	+	52	14	33	95	20	47
	SiHSP60-05	3	2403233	2409946	+	103	21	36	78	10	10
	SiHSP70-07	3	3180564	3183107	+	2	2	9	5	1	4
	SiHSP70-08	3	5573789	5578979	-	144	19	95	150	40	157
	SiHSP70-09	3	6988813	6994353	-	221	2	7	225	8	6
	SiHSP70-10	3	7000063	7005788	+	129	2	9	150	1	9
	SisHSP-09	3	8276444	8277820	-	112	48	37	172	70	50
	SiHSP60-06	3	11971877	11977268	+	87	1	4	81	1	2
	SisHSP-10	3	14350334	14352367	-	49	17	15	59	25	47
	SiHSP70-11	3	17574222	17577356	+	4	1	13	3	3	6
	SiHSP70-12	3	21467161	21473122	-	238	36	116	299	52	186
	SiHSP60-07	3	26620668	26625685	+	2301	29	75	2018	39	102
	SiHSP70-13	3	41234666	41238851	-	63	5	16	40	7	1
	SiHSP60-08	4	699908	704765	-	275	2	21	195	7	18
	SiHSP60-09	4	3925434	3931187	-	72	1	6	72	1	3
	SiHSP70-14	4	4474303	4477299	-	43	14	37	11	2	22
	SisHSP-11	4	6236824	6238049	-	107	32	57	307	66	87
	SisHSP-12	4	7456674	7457892	-	43	20	29	42	14	15
	SisHSP-13	4	7475921	7476915	-	10	1	5	4	7	0
	SisHSP-14	4	10433864	10434817	-	32	1	8	44	4	1
	SiHSP60-10	4	27834664	27840602	+	109	29	48	48	38	78
	SiHSP60-11	4	36307046	36312007	-	164	4	7	58	1	10
	SiHSP60-12	4	37441166	37446206	+	112	4	7	81	4	4
	SiHSP70-15	4	37951108	37956799	-	120	3	11	40	1	6
	SiHSP60-13	5	5416922	5421635	-	126	1	9	101	2	11
	SisHSP-15	5	7897990	7898792	+	74	27	47	116	48	84
	SisHSP-16	5	7908007	7908850	+	0	0	3	3	3	3
	SisHSP-17	5	7909852	7910635	-	39	19	30	32	13	14
	SisHSP-18	5	7912355	7913513	-	32	12	16	28	8	26
	SisHSP-19	5	7923272	7924004	-	1	0	0	4	0	1
	SisHSP-20	5	7924595	7925730	+	45	12	21	17	12	7
	SisHSP-21	5	11220808	11221302	-	40	19	23	122	19	81
	SisHSP-22	5	11223563	11224171	-	2	0	1	5	0	3
	SiHSP70-16	5	23768496	23770499	+	36	4	7	49	1	25
	SiHSP70-17	5	23871912	23873915	+	16	0	3	42	1	18
	SisHSP-23	5	31718600	31720000	+	7	3	7	3	4	3
	SisHSP-24	5	31740859	31741583	+	41	21	23	61	29	10
	SiHSP70-18	5	41050516	41053751	+	5	4	15	1	9	6

	SisHSP-25	6	20972495	20973792	-	23	11	15	12	4	10
	SiHSP70-19	6	34467838	34469692	-	29	16	10	26	9	3
	SiHSP60-14	7	21074009	21077334	-	42	2	2	14	1	1
	SisHSP-26	7	21161295	21162303	+	99	27	64	80	21	74
	SiHSP60-15	7	26596337	26601494	+	66	2	10	36	2	10
	SiHSP70-20	7	35472298	35473748	-	142	18	22	93	8	14
	SisHSP-27	8	8605725	8606738	+	82	43	36	161	49	97
	SiHSP60-16	8	30233760	30238633	-	99	0	7	55	1	6
	SiHSP70-21	8	37819280	37822074	+	1	0	0	0	3	8
	SiHSP60-17	9	187201	190487	+	178	5	15	146	11	7
	SiHSP70-22	9	1843820	1847776	-	1158	23	44	334	7	29
	SiHSP70-23	9	7468266	7470511	-	122	48	78	174	51	137
	SisHSP-28	9	9816080	9819828	+	37	24	28	18	21	19
	SisHSP-29	9	9820171	9821928	+	20	25	35	17	5	0
	SisHSP-30	9	9826133	9826973	+	35	18	42	29	7	25
	SiHSP70-24	9	13463310	13467080	+	204	4	8	89	5	8
	SiHSP60-18	9	17442926	17447951	-	109	8	12	64	14	6
	SisHSP-31	9	19017777	19018715	-	50	14	42	36	14	29
	SisHSP-32	9	19020419	19021508	-	30	5	39	52	12	20
	SisHSP-33	9	31685191	31686227	+	115	58	115	100	37	49
	SiHSP70-25	9	49893761	49896547	+	76	26	116	83	24	100
	SiHSP70-26	9	49939979	49942366	-	73	22	62	35	21	48
	SisHSP-34	9	50454063	50454638	+	91	53	96	169	120	199
	SisHSP-35	9	50484374	50485172	-	67	34	58	107	57	128
	SisHSP-36	9	50485555	50487142	+	40	28	25	108	104	69
	SisHSP-37	9	51325204	51326419	-	23	6	13	7	2	8
	SiHSP70-27	9	52763610	52767974	+	57	13	14	178	49	19
	SiHSP60-19	9	56420402	56425609	+	61	1	5	39	0	5
	SiHSP60-20	9	58126455	58130544	+	170	24	109	399	48	124
	SiHSP90-01	2	6007915	6010199	+	4	0	5	0	0	0
	SiHSP90-02	2	34600248	34605291	-	65	0	10	25	0	0
	SiHSP90-03	3	45081885	45088781	+	133	1	35	27	2	31
	SiHSP90-04	4	39628388	39633588	-	47	4	4	41	0	1
	SiHSP90-05	6	31466879	31471644	-	40	1	4	49	3	8
	SiHSP90-06	6	32063209	32065743	-	63	17	78	73	23	83
	SiHSP90-07	6	32076788	32080613	-	63	5	5	53	2	2
	SiHSP90-08	6	32083265	32087190	-	127	10	43	77	34	66
	SiHSP90-09	7	1556544	1559316	+	120	48	96	99	21	75
	SiHSP100-01	1	5797867	5798067	+	0	0	1	3	0	2
	SiHSP100-02	1	12407707	12407907	-	0	0	0	0	0	0
	SiHSP100-03	1	21562257	21562457	+	0	0	2	0	0	0
	SiHSP100-04	1	25559389	25559589	+	1	0	0	0	0	0
	SiHSP100-05	1	26386750	26386950	+	18	5	1	31	1	0
	SiHSP100-06	1	26480704	26480904	+	0	0	1	0	0	0
	SiHSP100-07	1	28004999	28005199	+	0	0	0	0	0	0
	SiHSP100-08	3	5977599	5977799	-	0	0	1	0	0	1
	SiHSP100-09	3	12275728	12275928	-	1	0	1	2	0	1
	SiHSP100-10	3	13015867	13016067	+	0	0	1	1	1	1
	SiHSP100-11	5	36658482	36658682	+	0	0	0	0	0	0
	SiHSP100-12	6	6767802	6768002	+	0	0	0	0	0	0
	SiHSP100-13	7	13430527	13430727	-	0	0	0	0	0	0
	SiHSP100-14	7	18352831	18353031	-	0	0	0	0	0	0
	SiHSP100-15	7	18362846	18363046	-	0	0	0	0	0	0
	SiHSP100-16	7	19133252	19133452	+	0	0	2	0	0	1
	SiHSP100-17	7	19676097	19676297	+	24	15	9	16	14	12
	SiHSP100-18	8	2488788	2488988	+	0	0	0	0	0	0
	SiHSP100-19	9	8489362	8489562	+	381	43	11	511	60	38
	SiHSP100-20	9	42170586	42170786	-	0	0	0	0	0	0

**Promoter**	SiHSP60-01	1	179528	179728	+	1	0	5	0	0	1
	SisHSP-01	1	3123686	3123886	-	0	0	0	3	0	0
	SisHSP-02	1	4474301	4474501	+	0	1	0	0	0	0
	SiHSP60-02	1	7299567	7299767	-	0	1	0	0	0	0
	SisHSP-03	1	8792090	8792290	-	0	0	0	0	0	0
	SiHSP70-01	1	10246200	10246400	-	0	0	0	0	0	0
	SiHSP70-02	1	36152053	36152253	+	0	0	1	0	0	0
	SisHSP-04	1	36168843	36169043	+	1	0	0	2	1	1
	SisHSP-05	1	38572587	38572787	+	0	0	0	0	0	0
	SisHSP-06	1	39834212	39834412	+	10	6	39	7	0	8
	SiHSP70-03	2	227627	227827	-	0	0	0	0	0	0
	SiHSP60-03	2	589354	589554	-	0	0	0	0	0	0
	SiHSP60-04	2	14956615	14956815	+	0	1	1	0	1	1
	SiHSP70-04	2	32024819	32025019	+	0	0	0	0	0	0
	SiHSP70-05	2	35804408	35804608	-	0	0	0	0	0	0
	SisHSP-07	2	41831023	41831223	+	1	0	0	0	0	1
	SiHSP70-06	3	650738	650938	-	0	0	0	0	0	0
	SisHSP-08	3	2125163	2125363	+	0	0	0	0	0	1
	SiHSP60-05	3	2403032	2403232	+	6	0	1	4	0	6
	SiHSP70-07	3	3180363	3180563	+	4	2	8	0	0	2
	SiHSP70-08	3	5573588	5573788	-	0	0	0	0	0	0
	SiHSP70-09	3	6988612	6988812	-	0	0	0	0	0	0
	SiHSP70-10	3	6999862	7000062	+	0	0	0	0	0	0
	SisHSP-09	3	8276243	8276443	-	0	0	0	0	0	0
	SiHSP60-06	3	11971676	11971876	+	0	0	0	0	0	0
	SisHSP-10	3	14350133	14350333	-	1	1	0	0	1	5
	SiHSP70-11	3	17574021	17574221	+	2	2	0	0	0	0
	SiHSP70-12	3	21466960	21467160	-	0	0	0	0	0	0
	SiHSP60-07	3	26620467	26620667	+	0	0	0	0	0	0
	SiHSP70-13	3	41234465	41234665	-	0	1	0	1	0	3
	SiHSP60-08	4	699707	699907	-	0	0	0	0	0	1
	SiHSP60-09	4	3925233	3925433	-	0	0	0	0	0	1
	SiHSP70-14	4	4474102	4474302	-	1	0	4	0	1	8
	SisHSP-11	4	6236623	6236823	-	0	0	0	0	0	0
	SisHSP-12	4	7456473	7456673	-	0	0	0	0	1	0
	SisHSP-13	4	7475720	7475920	-	1	1	1	0	0	0
	SisHSP-14	4	10433663	10433863	-	0	0	0	0	0	0
	SiHSP60-10	4	27834463	27834663	+	18	8	15	21	7	2
	SiHSP60-11	4	36306845	36307045	-	0	0	0	0	0	0
	SiHSP60-12	4	37440965	37441165	+	1	0	0	0	2	0
	SiHSP70-15	4	37950907	37951107	-	0	1	0	1	0	0
	SiHSP60-13	5	5416721	5416921	-	0	1	0	0	0	1
	SisHSP-15	5	7897789	7897989	+	0	0	0	0	0	0
	Si003160m	5	7907806	7908006	+	35	30	43	21	9	8
	SisHSP-17	5	7909651	7909851	-	0	0	0	0	1	0
	SisHSP-18	5	7912154	7912354	-	0	0	0	2	0	0
	SisHSP-19	5	7923071	7923271	-	42	28	35	34	17	35
	SisHSP-20	5	7924394	7924594	+	0	0	0	0	0	0
	SisHSP-21	5	11220607	11220807	-	0	2	0	0	0	1
	SisHSP-22	5	11223362	11223562	-	0	0	0	0	0	0
	SiHSP70-16	5	23768295	23768495	+	16	14	14	39	32	21
	SiHSP70-17	5	23871711	23871911	+	18	22	16	58	58	23
	SisHSP-23	5	31718399	31718599	+	0	1	1	0	0	0
	SisHSP-24	5	31740658	31740858	+	0	0	0	5	0	0
	SiHSP70-18	5	41050315	41050515	+	21	17	39	40	7	48
	SisHSP-25	6	20972294	20972494	-	0	1	1	0	0	0
	SiHSP70-19	6	34467637	34467837	-	0	0	0	0	0	0
	SiHSP60-14	7	21073808	21074008	-	1	0	1	0	0	0

	SisHSP-26	7	21161094	21161294	+	0	0	2	0	0	0
	SiHSP60-15	7	26596136	26596336	+	1	0	0	3	0	0
	SiHSP70-20	7	35472097	35472297	-	0	0	0	0	0	0
	SisHSP-27	8	8605524	8605724	+	1	2	0	1	0	0
	SiHSP60-16	8	30233559	30233759	-	0	0	0	0	0	0
	SiHSP70-21	8	37819079	37819279	+	0	0	0	1	0	0
	SiHSP60-17	9	187000	187200	+	0	0	0	0	0	0
	SiHSP70-22	9	1843619	1843819	-	1	0	0	0	0	0
	SiHSP70-23	9	7468065	7468265	-	0	0	0	0	0	1
	SisHSP-28	9	9815879	9816079	+	0	0	0	1	0	0
	SisHSP-29	9	9819970	9820170	+	0	0	7	0	0	0
	SisHSP-30	9	9825932	9826132	+	0	0	0	0	0	0
	SiHSP70-24	9	13463109	13463309	+	1	0	0	0	0	0
	SiHSP60-18	9	17442725	17442925	-	0	0	0	0	0	0
	SisHSP-31	9	19017576	19017776	-	0	1	0	0	0	0
	SisHSP-32	9	19020218	19020418	-	8	6	0	0	0	0
	SisHSP-33	9	31684990	31685190	+	0	0	0	2	0	0
	SiHSP70-25	9	49893560	49893760	+	0	0	0	3	1	0
	SiHSP70-26	9	49939778	49939978	-	2	2	35	0	1	16
	SisHSP-34	9	50453862	50454062	+	5	6	8	3	2	6
	SisHSP-35	9	50484173	50484373	-	5	1	1	3	0	4
	SisHSP-36	9	50485354	50485554	+	0	0	0	0	0	0
	SisHSP-37	9	51325003	51325203	-	0	0	0	0	0	0
	SiHSP70-27	9	52763409	52763609	+	0	0	1	0	0	1
	SiHSP60-19	9	56420201	56420401	+	0	0	0	0	0	1
	SiHSP60-20	9	58126254	58126454	+	0	0	1	0	0	0
	SiHSP90-01	2	6007714	6007914	+	1	0	0	0	0	0
	SiHSP90-02	2	34600047	34600247	-	0	0	0	0	1	0
	SiHSP90-03	3	45081684	45081884	+	1	0	0	0	0	0
	SiHSP90-04	4	39628187	39628387	-	0	0	0	0	0	0
	SiHSP90-05	6	31466678	31466878	-	0	0	0	0	0	0
	SiHSP90-06	6	32063008	32063208	-	0	0	0	0	0	0
	SiHSP90-07	6	32076587	32076787	-	0	0	0	0	0	0
	SiHSP90-08	6	32083064	32083264	-	0	0	0	0	0	0
	SiHSP90-09	7	1556343	1556543	+	67	28	54	65	10	39
	SiHSP100-01	1	5797867	5798067	+	0	0	1	3	0	2
	SiHSP100-02	1	12407707	12407907	-	0	0	0	0	0	0
	SiHSP100-03	1	21562257	21562457	+	0	0	2	0	0	0
	SiHSP100-04	1	25559389	25559589	+	1	0	0	0	0	0
	SiHSP100-05	1	26386750	26386950	+	18	5	1	31	1	0
	SiHSP100-06	1	26480704	26480904	+	0	0	1	0	0	0
	SiHSP100-07	1	28004999	28005199	+	0	0	0	0	0	0
	SiHSP100-08	3	5977599	5977799	-	0	0	1	0	0	1
	SiHSP100-09	3	12275728	12275928	-	1	0	1	2	0	1
	SiHSP100-10	3	13015867	13016067	+	0	0	1	1	1	1
	SiHSP100-11	5	36658482	36658682	+	0	0	0	0	0	0
	SiHSP100-12	6	6767802	6768002	+	0	0	0	0	0	0
	SiHSP100-13	7	13430527	13430727	-	0	0	0	0	0	0
	SiHSP100-14	7	18352831	18353031	-	0	0	0	0	0	0
	SiHSP100-15	7	18362846	18363046	-	0	0	0	0	0	0
	SiHSP100-16	7	19133252	19133452	+	0	0	2	0	0	1
	SiHSP100-17	7	19676097	19676297	+	24	15	9	16	14	12
	SiHSP100-18	8	2488788	2488988	+	0	0	0	0	0	0
	SiHSP100-19	9	8489362	8489562	+	381	43	11	511	60	38
	SiHSP100-20	9	42170586	42170786	-	0	0	0	0	0	0

Supplementary Table 8. List of primers used in quantitative real time-PCR expression analysis of *SiHSP* genes.

GENE ID	FORWARD PRIMERS (5′- 3′)	REVERSE PRIMERS (5′- 3′)
SiHSP100-07	GCAGCAGGATTTGGAAGAGT	CACCCAGCAGCCATTACA
SiHSP100-08	GGGTGACTCTGCCATTGT	TCTAAACCGTGACTGCCG
SiHSP100-11	TGGGGCTGTCAAGTGCTAA	GGGTAATCAACACACTCCGA
SiHSP100-12	ACGAACACGCTCATCATCA	TCTCCTTCACCTCCACCTTG
SiHSP100-16	TCACCATAGAAGGCATACATT	TACGGACATAGACGGCTG
SiHSP100-18	TCTAATGCTTCCTCCCCTCG	CTCACTCCGTCCTCTTCTCC
SiHSP100-20	TTCAAGGACGAGGACAGCAT	TCATACTCCATCAGACGGC
SiHSP60-02	CGTGTGATGTGAGTGATTC	GTGGCGGCTACATTCATAA
SiHSP60-03	AGTTTTCACGGCTTTTGT	CGCTTTTGGATTGTAACGC
SiHSP60-05	ATGGGTTTTGTTTGGCTATTG	TGTATCTATCACTCAGGTCACG
SiHSP60-09	GCAGCCATTACTATCCTCAG	ACAACCATCACGAAACAGC
SiHSP60-13	GGGAACTGTTTTTTGTGC	CATTAACACCAGGAGCAGCA
SiHSP60-14	CCACTTCCATAATCCTCGC	GTTCAGACACCAAAGCAGCA
SiHSP60-16	TCCTGGTGTATTGCGATGC	TCAGATGAACAAACCCCG
SiHSP60-20	GTCACCGTAAGATAAGGGC	CAATCCAGACAACAGGGC
SiHSP70-01	CATCATCTCGGCGGTGTA	CCCAAGAGTCATTCATAAGC
SiHSP70-04	AGTCAACATCTGCTGCCAA	GACACAAGAGTTAGTAGTTCCCA
SiHSP70-06	ATGTCAATGGCATCCTCC	TTCTTGTCTTCCTCGGCG
SiHSP70-15	CCCACCGTCATCTTCTTG	TTGGCTTCTGTTCATTCATCA
SiHSP70-16	AAGATGACCGACAAGATGGAC	TACACCGCCGAGATGATAG
SiHSP70-19	GGGACACGGCTATGATGG	GACGGCAACTGAAATGGG
SiHSP70-21	CCAGGTTTACGAGGGTGA	TCCTTGGCAGAGACTTCC
SiHSP70-24	CCACTTGTTCCTCCTCAGCA	TGTCACCAAAGCATCACC
SiHSP90-02	GGTTGTGCTGCGAAGATAA	CCTTGTGTGCGTGAATGTA
SiHSP90-03	TGAGATTTTGTTCCAGCGGC	GGGCTTCCAATGTCCAACTAC
SiHSP90-04	TTGGTTCTCAGGGACGCC	GAAGACACGAAGCACACG
SiHSP90-05	GAAGGCACTGTAACGGAGGT	ACAAAGGATGCCCAAGAGAT
SiHSP90-09	AGTTCTACGACGCCTTCTCC	GATGTCCTTCTGCCCTTCCT
SisHSP-02	CCTTGCGTGTTGTCCTCCT	GGTTACTCTGCTCTGGGTGA
SisHSP-08	TAAGCCACCATAAACGG	AGGAGAGAGCAGGGAGAAGA
SisHSP-09	CCTCTGCCTCCTTATCGC	ACTGCCGTCTTCCTCTGGTA
SisHSP-12	ATGGAGAGAGATTGCGAAC	AGACATCAGGCACGAGACA
SisHSP-16	CGTGTCCATCGTTTGTGTT	CTCAGTTTATTCGGCTCC
SisHSP-27	AGGACAAAACTACCCAAGG	GCCGAAACTCAGAAAAGG
SisHSP-28	CGACGCCAAGAAGAGCAT	CGTAAAGCCAAGTCACCA
SisHSP-29	CAAAGCCGAGATGTGAGC	ATAATAGGGCGGACAAGATGA
SisHSP-32	ATGCTGCCAAGAACGATGA	CAACACCCACCAATGCGAG
